# Gluebodies Offer a Route To Improve Crystal Reliability and Diversity through Transferable Nanobody Mutations That Introduce Constitutive Close Contacts

**DOI:** 10.1021/acscentsci.5c00937

**Published:** 2025-10-27

**Authors:** Mingda Ye, Mpho Makola, Mark W. Richards, Joseph A. Newman, Michael Fairhead, Selena G. Burgess, Zhihuang Wu, Elizabeth Maclean, Nathan D. Wright, Lizbé Koekemoer, Andrew Thompson, Gustavo Arruda Bezerra, Gangshun Yi, Huanyu Li, Victor L. Rangel, Dimitrios Mamalis, Hazel Aitkenhead, Benjamin G. Davis, Robert J.C. Gilbert, Katharina L. Duerr, Richard Bayliss, Opher Gileadi, Frank von Delft

**Affiliations:** † Centre for Medicines Discovery, Nuffield Department of Medicine, 6396 University of Oxford, Oxford OX3 7FZ, U.K.; ‡ Astbury Centre for Structural Molecular Biology, School of Molecular and Cellular Biology, Faculty of Biological Sciences, 4468 University of Leeds, Leeds LS2 9JT, U.K.; § Division of Structural Biology, Wellcome Centre for Human Genetics, University of Oxford, Roosevelt Drive, Oxford OX3 7BN, U.K.; ∥ Laboratory of Protein Crystallography, School of Pharmaceutical Sciences of Ribeirão Preto, University of São Paulo, Ribeirão Preto, São Paulo 05508-000, Brazil; ⊥ Department of Chemistry, 6396 University of Oxford, Oxford OX1 3TA, U.K.; # The Rosalind Franklin Institute, Oxfordshire, Oxford OX11 0QS, U.K.; ◆ Department of Pharmacology, University of Oxford, Oxford OX1 3QT, U.K.; ¶ Calleva Research Centre for Evolution and Human Sciences, Magdalen College, University of Oxford, Oxford OX1 4 AU, U.K.; □ Kavli Institute for Nanoscience Discovery, Department of Chemistry, University of Oxford, Oxford OX1 3QU, U.K.; ■ SGC Karolinska Center for Molecular Medicine, Karolinska University Hospital, 171 76 Stockholm, Sweden; ◇ Diamond Light Source, Harwell Science and Innovation Campus, Didcot OX11 0DE, U.K.; ⊖ Research Complex at Harwell, Harwell Science and Innovation Campus, Didcot OX11 0FA, U.K.; ⦶ Department of Biochemistry, University of Johannesburg, Auckland Park 2006, South Africa

## Abstract

Design of modular, transferable protein assemblies has broad applicability and in structural biology could help with the ever-troublesome crystallization bottleneck, including finding robustly behaved protein crystals for rapidly characterizing ligands or drug candidates or generating multiple polymorphs to illuminate diverse conformations. Nanobodies as crystallization chaperones are well-established but still unreliable, as we show here. Instead, we show an exemplar of how robust crystallization behavior can be engineered by exploring many combinations (>200) of nanobody surface mutations over several iterations. Critically, what needed testing was crystallization and diffraction quality, since target–nanobody binding affinity is decoupled from crystallizability enhancement. Our study yielded multiple polymorphs, all mediated by the same interface, with dramatically improved resolution and diffraction reliability for some mutants; we thus name them ‘Gluebodies’ (Gbs). We further demonstrate that these Gb mutations do transfer to some other targets, both for achieving robust crystallization in alternative packing forms and for establishing the ability to crystallize a key early stage readout. Since the Gb interface is evidently a favored interaction, it may be broadly applicable for modular assembly; more specifically, this work suggests that Gbs should be routinely attempted for crystallization whenever nanobodies are available.

## Graphical abstract

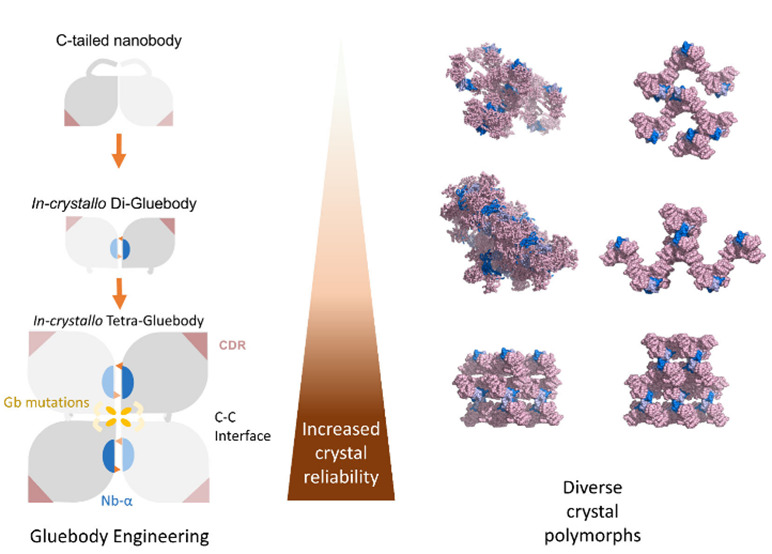

## Introduction

Protein X-ray crystallography has long been a routinely used method to elucidate the near-atomic structures of proteins of interest. However, enabling the protein to consistently crystallize can prove to be highly troublesome, with no substantial methodological breakthroughs for many decades. The mechanistic specifics remain poorly understood, so there is no systematic route to coax a protein to pack in an ordered lattice.^1–5^ Nevertheless, numerous techniques have emerged throughout the past decade to aid crystallization, and they generally follow one of four general strategies.

The first is to vary the protein environment by exploring crystallization solution, temperature, or physical format.^6,7^ This approach has been thoroughly commercialized for over two decades, with a large repertoire of crystallization screens purchasable from vendors, and many laboratories equipped with various systems of automation.^8^

The second strategy is to modify the protein itself to favor crystallization, either by introducing major changes to the protein through varying the expression construct^9^ or by subtle changes on the protein surface, such as by surface entropy reduction,^10^ chemical modifications,^11^ or the ‘crystal epitope’ approach.^12,13^ Using protein orthologues that crystallize more easily is also an alternative.^14^

The third approach is to introduce a natural partner that forms a complex with the target of interest. The natural binding partner, including ligands and proteins, could potentially stabilize the target or introduce additional surface for forming crystal contacts.^15–18^

The fourth approach is to use a protein ‘chaperone’ engineered to provide the target with additional or alternative potential crystal contacts. Reported chaperones include fusion tags such as lysozyme^19^ or BRIL;^20^ and protein binders such as Fabs,^21^ scFvs,^22^ nanobodies/sybodies,^23,24^ or DARPins.^25^ The required use of fusion tags can be challenging since their addition disrupts the native structure, decreases protein yields, or otherwise impedes biochemical behavior.^9^ As a result, this strategy often requires extensive testing of a combinatorial matrix of constructs to identify the insertion point that optimizes protein packing and thus improves diffraction.^26^ Consequently, binder-assisted protein crystallization has tended to be used as a last resort for the most challenging targets, such as membrane proteins,^27^ because these tend to be long-term projects where the necessarily extended timescales required for obtaining binders are not seen as rate-limiting. Nevertheless, thanks to the improvement and diversification of binder selection methods, the generation of binders to assist structural studies is now increasingly attempted in many laboratories.^24,28,29^

Beyond these, more speculative suggestions to aid crystallization have also been reported, but have either not been well-developed (e.g., embedding the protein of interest into a highly porous crystal lattice^30–32^ ), or have not yet, despite their ingenuity, achieved widespread usage and thus validation (e.g., imprinted polymers,^33^ microgravity,^34^ racemic protein crystallography^35^).

Therefore, while none of these methods routinely allow enhancement of crystal reliability and diversity yet, binder-assisted crystallization perhaps holds the greatest promise to address several key crystallization challenges beyond simply obtaining a first crystal structure.^27^ First, such binders mask out surface heterogeneity and reduce the entropy for forming stable crystal lattices.^36^ Second, they can lock the target protein into conformations that cannot be otherwise isolated.^37,38^ Third, they can help find considerably more robust crystallization systems that are required for structure-based lead discovery, including crystal-based fragment screening, since the introduction of new or additional crystal–crystal contacts mediated by the chaperone can systematically increase polymorphism.^39,40^ Finally, by engineering binder scaffolds, symmetry can be introduced to the binder:target complex to further facilitate crystallization processes.^41,42^

Significant challenges prevent the current realization of this promise. Although benefits have been suggested in human Fabs (e.g., by shortening the FG loop by two residues)^43^ or diabody scaffolds (e.g., at the V_H_-V_H_ interface)^44^ these remain bespoke and nonmodular. Moreover, binders such as Fabs and diabodies are relatively difficult to express and purify, rendering the engineering process arduous.

Here, we demonstrate a workflow in the readily manipulated nanobody scaffold where, first, a wholly crystallization-ineffective nanobody can be made effective by a limited number of mutations on the nanobody scaffold and, second, enhanced with mutational screening of more than 200 constructs designed through data mining. This identifies four key mutations on the nanobody scaffold far from the CDR surfaces that lead to extensive crystal polymorphism and robust diffraction (Figure 1). Finally, we describe the ability of these engineered nanobodies to help enhance the crystallizability of proteins reluctant to crystallize. This engineerable process, driven by an interface trapped under kinetic (here crystallization) control, should prove transferable not only to other targets but indeed to other widely used chaperone scaffolds and even to other kinetically trapped processes.

**Figure 1:**
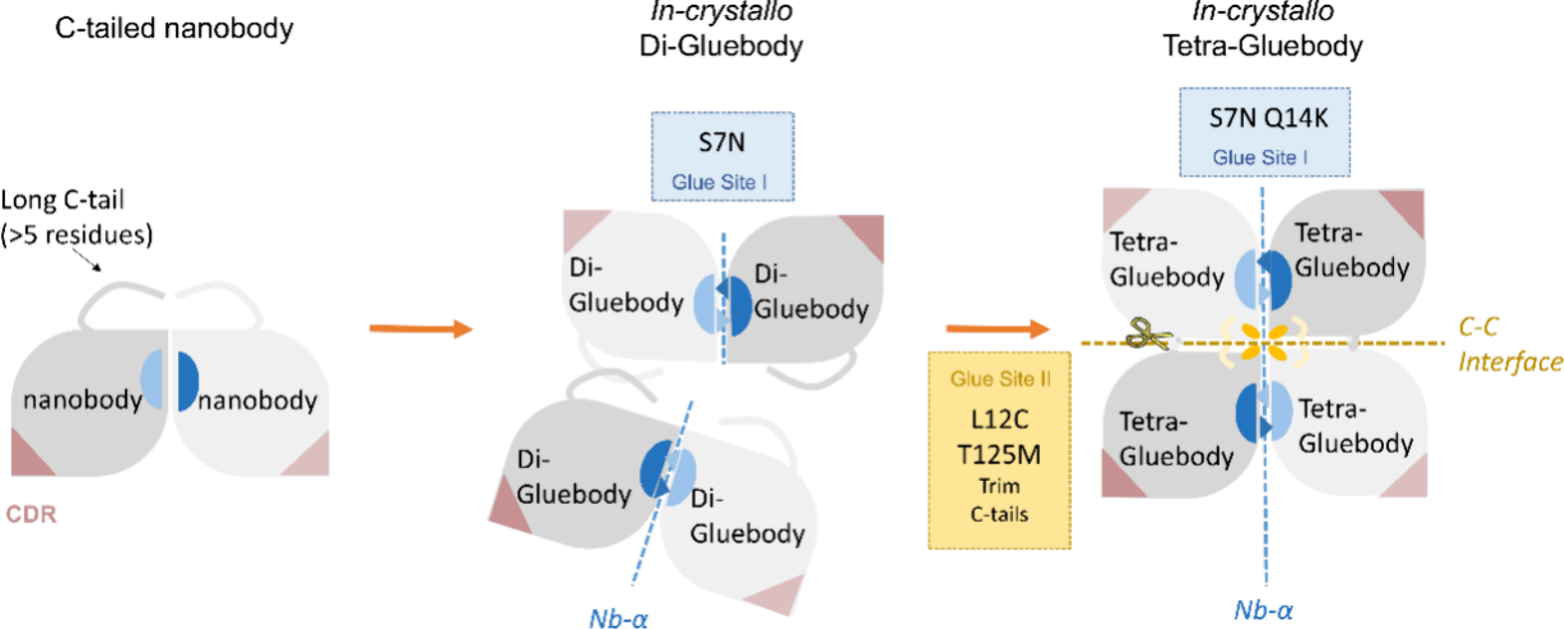
The evolution of the nanobody scaffold to Gluebodies. Progressive sets of mutations that engineer the Gluebody interface lead to diverse polymorphs with improved crystallizability and diffraction quality. The putative crystallization patterns from the three scaffolds are presented from left to right with the scaffold type at the top of the sketches. The leaf shapes colored in gray and light gray represent nanobody molecules. CDRs are indicated in light brown. The Nb-α contacts are shown in blue and light blue patches. The long or short overhangs on the leaf shapes represent the C-terminal tails of the nanobody molecules. The orange ovals, blue triangles, and pale hooks represent the key mutations on the nanobody scaffold (C12, N7 & K14, and M125, respectively). The dashed lines represent nanobody–nanobody interfaces occurring in the putative crystallization patterns.

## Results

### Naïve Nanobodies Are Unsatisfactory Crystallization Chaperones

We selected 17 nanobody–target pairs across five different target proteins (Table S1 and Figure S1). Among the targets, solved crystal structures (of apo RECQL5, RECQL5 bound with ADP/Mg^45^ and apo WRN^46^) were included as benchmark comparators. We performed standard crystallization trials of the 10 protein complexes (seven failed during purification) against two commercially available coarse screens (192 conditions and two temperatures) for each protein complex. This yielded crystals for only one protein complex (MAGEB1), which diffracted well and could be readily solved (PDB ID 6R7T). This initial screen suggested that the naïve effectiveness of appropriately binding nanobodies as crystallization chaperones without engineering is very low, in our experimental set less than 10%, demonstrating the need for dramatic improvement of the approach.

### A Single Nanobody Surface Mutation by the Crystal Epitope Approach Yields a First Effective Chaperone for RECQL5

The strategy of Crystal Epitope^13,47^ entails identifying short sequence motifs (3–6 residues) that frequently appear in crystal contacts in the Protein Data Bank (PDB). We have previously successfully used this strategy to obtain crystals of several different target proteins (e.g., BRD1A: 5AMF, GALT: 6GQD, glycogenin-2 catalytic domain: 4UEG), and similarly, we applied it to the non-CDRs of a specific nanobody targeting RECQL5.

Seventy-four crystal epitope mutations (variant class G0 and variant G1-001) were designed through three iterations on the scaffold (non-CDR, nonvariable region) of a RECQL5 nanobody (Figures S3A and S3B, Table S2). Purified nanobodies all comigrated with RECQL5 on size exclusion chromatography, indicating adequate affinity of the nanobodies. The purified protein complexes were put into crystallization trials using the Hampton Index coarse screen, and five of the 74 yielded crystals (Figures S3C, S3D, and S3E). These variants shared one common mutation, D69Y, the single mutation of variant G1-001 (Figure S2 and Table S4), and crystals of the G1-001 complex diffracted to the highest resolution (2.76 Å). Consistent with this initial design, in the mutant structure, Y69 on the nanobody forms a hydrogen bond with D400 of RECQL5, which most likely stabilizes the crystal formation. The striking effect that this emergent single mutation has on its crystallizability gave the first proof-of-principle that the nanobody scaffold could be engineered for improved crystallization—a general screening approach can be applied to nanobodies targeting different proteins, thereby bootstrapping the chances to obtain a first crystal.

### A Single Nanobody–Nanobody Interface Predominates in PDB Structures

This demonstrated that the utility of non-CDR surface mutations increased crystallization propensity while preserving nanobody affinity for its target protein. Nonetheless, the effective mutations were not mediating nanobody–nanobody crystal contacts likely to build a modular lattice and thus were only useful for their specific target RECQL5. To optimize a nanobody interface independent of target proteins, we next applied a general strategy applicable to all nanobody–protein complexes by engineering only nanobody–nanobody interactions (Figure 2).

**Figure 2:**
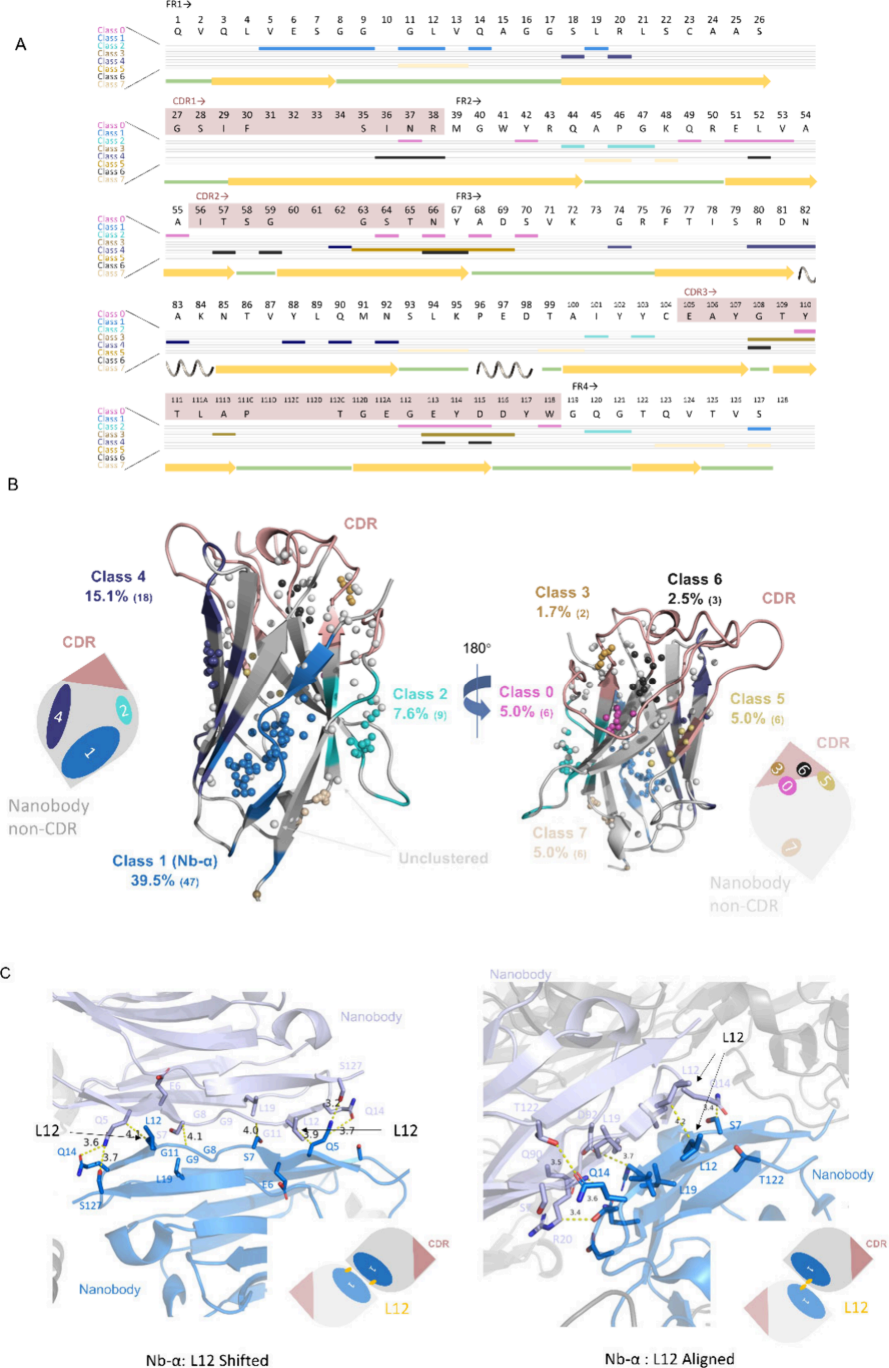
Nanobody–nanobody crystal contacts fall into limited classes. (A) Standard nanobody residue numbering of C-terminally tagged wild type RECQL5 nanobody (C-tag:WT) using an IMGT scheme provided by ANARCI.^49^ CDRs are in a light brown background, and non-CDRs are in a white background. Participating residues of each category are indicated by a bar below the one-letter code. (B) DBSCAN clustering result: global view of major nanobody–nanobody crystal interfaces on a model drawn from chain E of 5O0W in the PDB. Each colored sphere on the structure represents the average location of participating residues of an interface analyzed. Gray spheres represent ungrouped nanobody interfaces in the analysis. The three largest interface patterns are colored on the cartoon of the nanobody structure with the respective class colors. Simplified nanobody sketches are shown at the corners with CDRs (light brown) and numbered contact classes, indicating their relative locations on a nanobody. The areas of contact color patches are proportional to their respective percentages. Colors are consistent in A and B. (C) Typical close views of Class 1 (Nb-α) nanobody–nanobody crystal contacts. L12 Shifted type is shown in the left panel, and the L12 Aligned type is shown in the right panel. Structure cartoons in marine and light blue represent two nanobody molecules participating in the interface. Residues represented as sticks are interacting residues. The simplified nanobody sketches are shown at the bottom corners (see Figure S4 and Table S2 for detailed information of each class of nanobody–nanobody crystal contacts).

To understand how nanobodies commonly pack with each other in crystals, we examined the 335 nanobody-containing crystal structures in the PDB. We defined two molecules as forming crystal contacts if their nearest atoms are below 4 Å apart^48^ and thus found 273 structures with nanobody–nanobody contacts. Contacts with more than five pairs of interacting residues were deemed suitable for engineering. In this way, interactions were refined to 356 large contacts across 119 structures for further analyses.

Through the use of a bespoke Python script (using the Python interface in Pymol 2.7), we were able to extract all interface information in the 335 crystal structures and focused on the interfaces that only involved nanobodies. The nanobody:nanobody interfaces were represented by index arrays of the residues involved. The index array was then renumbered (using an IMGT scheme on the online server ANARCI,^49^ Figure 2A), enabling comparisons across the nanobodies appearing in different deposited structures. The renumbered arrays were further converted into coordinate matrices, which then immediately shrank into a four-dimensional array (n*, μ_*x*_, μ_*y*_, μ_*z*_) (Figure S4). The four items in the array represent 1/3 of the number of residues in the interface, and the mean values of the *x*, *y*, *z* coordinates of the residues in the interface, respectively. Selected nanobody interfaces were then mapped onto a single nanobody structure (PDB ID 5O0W) using their coordinate information (Figure S4). As numeric representations of the interfaces, four-dimensional vectors were used, and we further performed density-based spatial clustering (DBSCAN) on the 356 vectors (Figure S4).

Clustering revealed eight classes of nanobody-nanobody crystal contacts. Four of them (classes 1, 2, 4, and 7), away from the CDRs, account for 39.5%, 7.6%, 15.1%, and 5.0% of the total (Figure 2B). The most prevalent, termed ‘Nb-α’ (39.5%), is an edge-to-edge class 1 interaction of the two beta sheets at the N terminus (Figure 2C). The Nb-α contact typically has 10 residues interacting in this type of contact, creating a plethora of options for engineering an interface that is more likely to crystallize.

The other contact classes 0, 3, 5, and 6 are located in or very close to CDR regions and therefore are not likely to mediate crystal packing and target binding at the same time. Contact classes 2 and 7 have relatively rare appearances in the PDB. Finally, class 4 contact also presented a potentially good surface for engineering; however, it appeared with half the frequency and is closer to CDRs. We therefore chose to focus on surface engineering within the Nb-α contact family, which is also a very conserved N-terminal region across all nanobodies.

### Iterations of Nanobody Scaffold Mutagenesis Yield Consistently Diffracting Crystals Despite Loss of Affinity

Based on these mining results and within our RECQL5:nanobody system, we designed five generations of nanobody scaffolds (G1–G5) of more than 200 constructs with different combinations of Nb-α mutations and crystal epitope mutations (Figure S2 and Figure 3A). Mutations were designed that might enhance affinity within the interface by variously introducing salt bridges, hydrogen bonds, and disulfide bonds.

**Figure 3:**
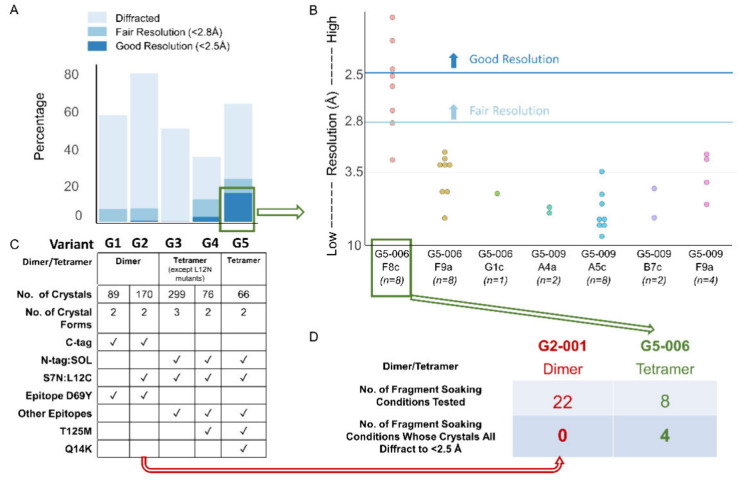
Consistently well-diffracting crystals emerge only after several iterations of mutagenesis around class 1 contact Nb-α. (A) The diffraction quality of different groups of variants is measured by the percentage of crystals that diffract to high resolution. The numbers of crystals and mutations present in each group of variants are indicated in the table below each bar. (B) Diffraction quality of different nanobody variant and condition pairs of RECQL5:nanobody variant complexes presented as a dotplot. (C) Summary of characters of G1–G5 variants, number of crystals tested and number of crystal forms observed. (D) A table summary of diffraction after testing cryoprotectants against crystals of G2-001 and G5-006 (details in Figure S8).

This yielded highly effective reagents; retrospective analysis allowed extraction of the mutational origins underpinning these successful iterations. In brief, G1 and G2 mutants, derived from the original RECQL5 nanobody scaffold, bore a C-terminal 6xHis tag that is cleaved during purification (resulting in six-residue tail ENLYFQ after TEV protease-mediated cleavage). G2 crystals with the S7N:L12C mutations showed a higher diffraction rate; however, the high-resolution fraction did not significantly increase. Next, in G3, solubility mutations (G40T:Q49E:L52W:I101V, abbreviated SOL, that mimic the well-expressing GFP-enhancer nanobody from PDB:3K1K) were combined with both movement of the C-terminal 6xHis tag to an N-terminal MBP-6xHis tag (which does not yield an ENLYFQ) as well as epitope mutations beyond D69Y. Those epitope mutations were a subset of G0 variants (Figure S3) with little overlap in the mutational sites. Although G3 crystals showed initially worsened diffraction quality, two mutations (T125 M and Q14K) based on G3 scaffolds drove significantly improved diffraction quality in G5.

We explored in further detail the crystals derived from G5. There were several crystallization conditions yielding crystals within just 2 days; these were selected. Ten to twelve crystals from each variant–condition pair were harvested for diffraction analysis. The pair G5-006-F8c had a significantly larger fraction of crystals diffracting better than 2.5 Å (Figure 3B). Improvements were also importantly obtained in processes employing optimization of cryo-protectant and DMSO concentrations (i.e., under potential fragment-soaking conditions). Out of the eight conditions we tested, 100% crystals from four conditions diffracted better than 2.5 Å (Figure 3C and Figure S8); this is a critically desirable resolution threshold to reliably determine the ligand-binding conformations.^50^ Notably, when comparing G5-006 with G2-001, the diffraction quality of G5-006 generation-5 crystals was significantly improved over generation-2, consistent with successful iterative engineering.

In summary, against the target RECQL5, we first obtained crystals with G1 constructs (a few crystal hits) and then optimized the Nb-α interface with S7N and L12C in G2 constructs, resulting in many more hits in PEG conditions. Both G1 and G2 crystals are driven by D69Y in the target:Nb interface and in a dimeric form. From G3 onward, we eliminated D69Y, but kept S7N:L12C, to increase the driving force of the Nb:Nb interface mediating crystal formation. Therefore, G3–G5 crystals all take a different tetrameric crystal form (except the L12N controls), which also would form under high salt conditions. While G3 crystal diffract poorly, we mutated the closest residue T125 to test a number of side chains (G3* constructs, Figure S2). Finally, we concluded that T125M is the most optimal for the interface as G4 constructs. The final mutation Q14K resulted in fast-growing and large crystals, which also yielded the best diffractions the thus named G5 crystals (Figure 3C and Figure S2). Notably, although the binding affinity of the G5-006 variant is somewhat weaker (1 log-unit),^51^ it is still sufficient for protein complexation and shows much-improved crystallizability.

### Constitutive Nanobody Interfaces Were Present in Six Different Crystal Forms

In the five generations of nanobody scaffolds, we looked for constitutive nanobody–nanobody interfaces that might be independent of the target protein, consistent with our design hypothesis.

Starting with Nb-α in G2, upon combining S7N:L12C with prior G1, the crystallization propensity of the RECQL5:nanobody complex increased dramatically for both N-terminally (N-tag) and C-terminally (C-tag) tagged variants (Figures 4A and 4B). Comparison of the structures of G1-001 with those of G2-001 reveals an additional hydrogen bond in Nb-α introduced by S7N between the side chain N7 and the carbonyl oxygen of A15 (Figure 4C). These S7N:L12C mutations were retained in the following iterations of mutagenesis.

**Figure 4:**
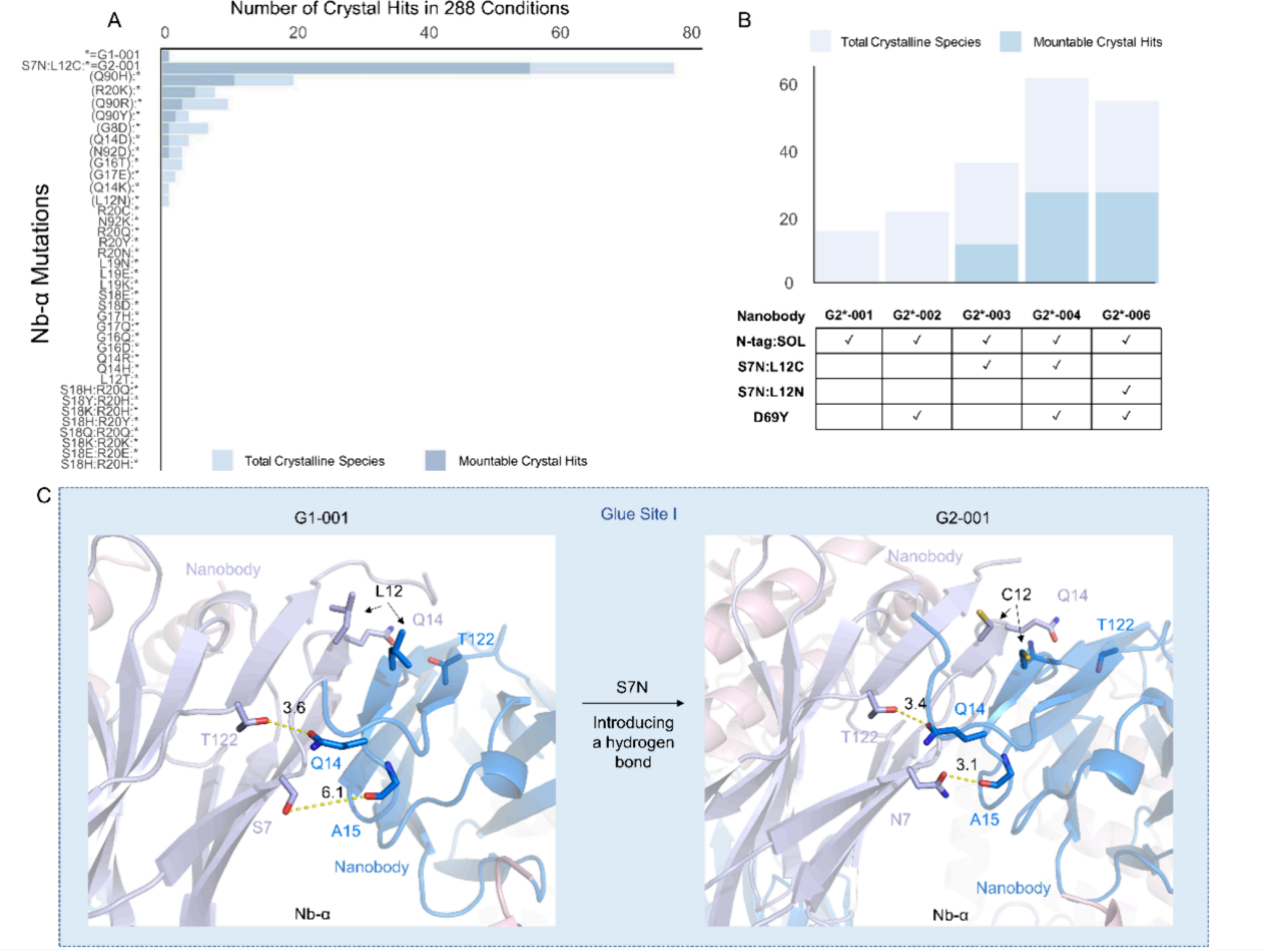
Mutation S7N enhances Nb-α by introducing an additional pair of hydrogen bonds. (A) Hampton Index 3 (HIN3) triplicate drop coarse crystallization screen experiments show that S7N:L12C stands out among Nb-α mutations in significantly increasing the crystallization propensity of the RECQL5:nanobody complex. The asterisk represents variant complex G1-001. (B) HIN3 triplicate drop coarse crystallization screen experiments show that S7N:L12C significantly increases the crystallization propensity of the N-tag:SOL variant of the RECQL5:nanobody complex with or without D69Y as the crystal epitope mutation. (C) Close views of the Glue Site I before and after the mutation. S7N (stick representation) introduces an additional hydrogen bond with the carbonyl oxygen of A15 (stick representation) on the opposite nanobody molecule. Oxygen atoms are colored red, and nitrogen atoms are colored blue. The distances between the atoms connected by dashed yellow lines are shown in Å.

Different combinations of crystal epitope mutations and Nb-α mutations were further explored in G3. We replaced D69Y with nine other crystal epitope mutations in G3 and changed the terminal tag position (see above). Crystallization patterns changed dramatically with most G3 crystals requiring high salt conditions, while most G2 crystals required PEG conditions (Figure 5A). Notably, Cys12 appeared important in the crystallization of G3—introduction of the C12N mutation decreased the average number of observed hits significantly (Figure 5A). Moreover, mutation of Cys12 to other residues (including via chemical mutation to dehydroalanine Dha,^52^ Figure S7) resulted in even ‘good crystallizers’ immediately losing crystallizing ability (Figure 5B).

**Figure 5:**
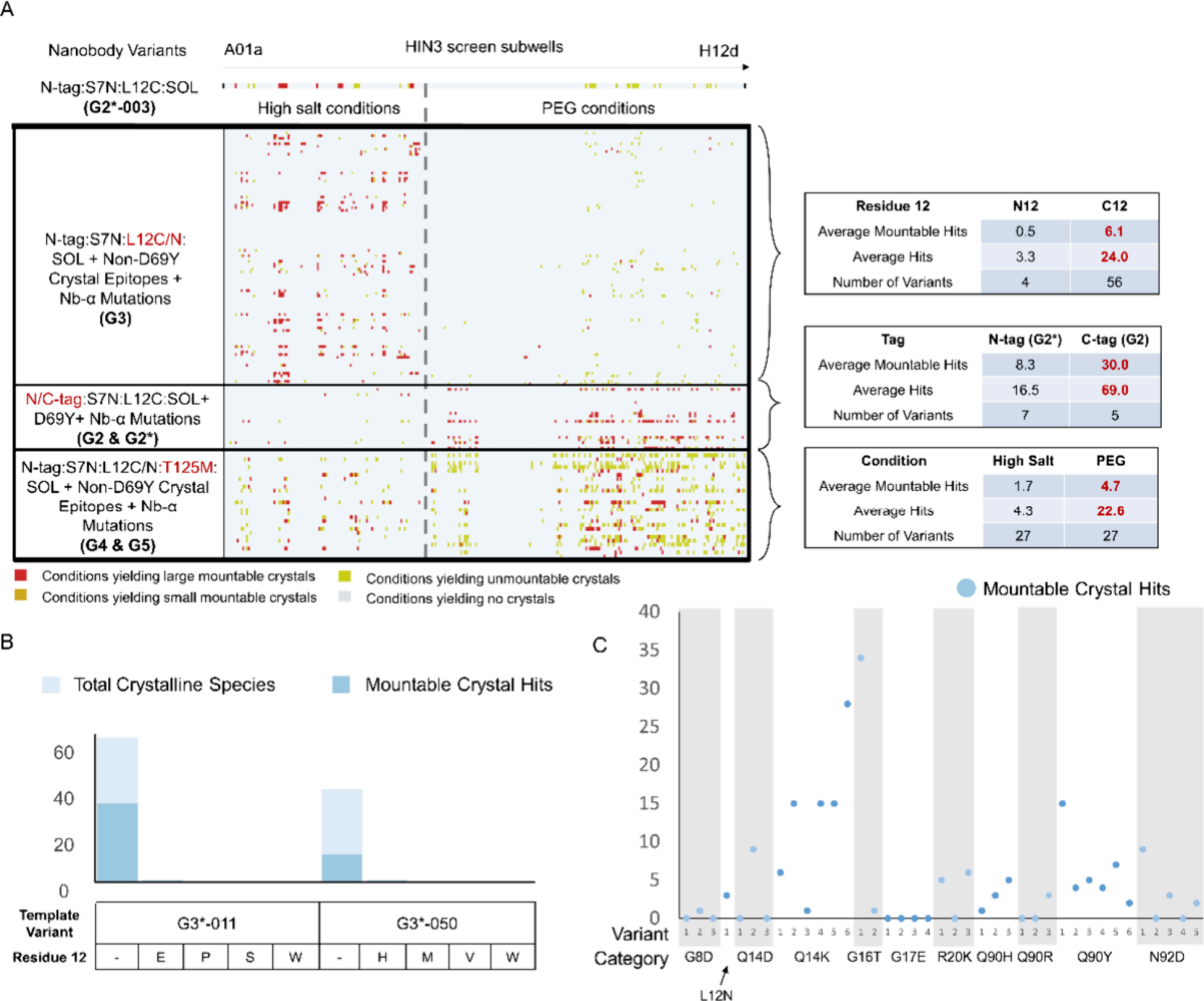
G2, G3, and G4&G5 have different crystallization patterns in the Hampton Index coarse screen. (A) Hampton Index 3 coarse screen triplicate crystal trials of the RECQL5:nanobody complex with various mutations on the nanobody scaffold. Each row represents a nanobody construct, and each column in the chart represents a crystallization condition in the Hampton Index 3 coarse screen. The conditions are linearized sequentially from A01a on the left to H12d on the right. To the left of the gray dashed line are high salt conditions and to the right are PEG conditions. The color of each cell in the row represents whether there are crystals growing in that condition and the quality of the crystals. Some statistics of each group of nanobody variants are generalized on the right of the main plot. See Table S5 for detailed information on what each row and column represents. (B) The RECQL5 nanobody loses crystallization propensity when C12 is mutated to a selection of other residues. (C) HIN3 triplicate drop coarse crystallization screen experiments show Q14K led to higher mountable crystal hits among a set of Nb-α mutations in G4&G5. The graph shows mountable crystal counts against each variant in respective Nb-α mutation categories.

Since Cys12 was demonstrated to be important in improving crystallization, the area around this striking focal point lynchpin residue was further explored by mutagenesis of neighboring residues. In particular, when T125 M was introduced in G4 and G5, the crystallization pattern again changed, expanding hits under both high salt and PEG conditions (Figure 5A). Furthermore, with the Nb-α mutation Q14K in G5, the crystallization propensity was further improved (Figure 5C) with even noticeable growth of large crystals. It should be noted that ambiguity in the electron densities captured from the K14 side chains means that the mechanistic origin of this improvement remains unknown, further highlighting the value in the discovery of our combined, iterative computation-plus-empirical approach over that based on structural analyses alone.

The significant change in crystallization observed on moving from G2 to G3 could also be explained. Consistent with a stepped evolutionary approach to these hierarchical changes, an entirely different nanobody arrangement emerges *in crystallo*. Instead of a dimer nanobody core (Figure 6A, left panel) mediated by Nb-α in G1 and G2, we observed a tetrameric nanobody core (Figure 6A, right panel) in G3. In this tetrameric nanobody arrangement, the Cys12 residues are in the core of the interface and in contact with each other. We named this emergent interface, which is perpendicular to Nb-α, the ‘C-C interface’. The emergence of a novel C-C interface at G3 can be further understood. Due to the geometric arrangement of the four nanobody molecules, the tetrameric core in G3 would not form with the six-residue C-terminal tail ENLYFQ still present; this tail can be observed, for example, in the G2-001 structure ‘hanging over’ the critical Cys12 residues (Figure 6A, left panel). The critical role of Cys12 can also be understood in forming a tetrameric nanobody arrangement. When mutated to Asn12, the C-C interface dissociates and only the dimeric nanobody arrangement is observed (Figure 6B), resulting in a much lower resolution (Table S4).

**Figure 6:**
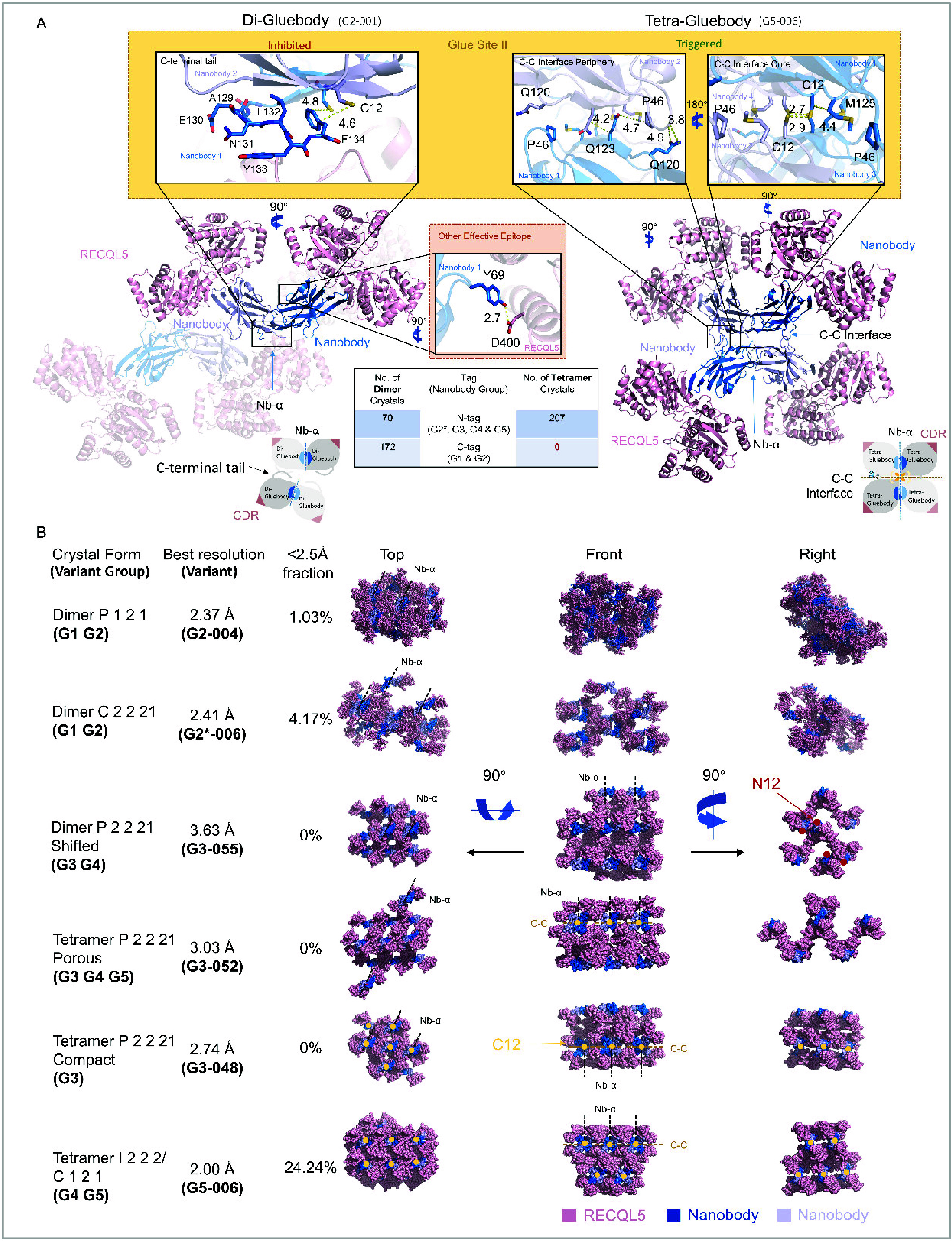
Two constitutive nanobody interfaces are found in six different crystal forms in Gluebodies. (A) *In crystallo* dimeric (Di-Gluebody) and *in crystallo* tetrameric (Tetra-Gluebody) crystallization pattern of RECQL5:nanobody with close views of Glue Site II and the crystal epitope site of Y69. The left panel shows RECQL5:nanobody complex crystallizes in a dimeric pattern when mutation D69Y and the C-terminal tail exist on the nanobody scaffold. The cartoon structure with high transparency is a symmetry mate of the dimer. The schematic of the dimer pattern is shown on the bottom left. The right panel shows the tetrameric crystallization. The M125 has a longer side chain than the T125 and occupies the cavity around the cysteine core within the C-C interface. The schematic of the tetramer pattern is shown on the bottom right. Oxygen atoms are colored red and nitrogen atoms are colored blue. The distances between the atoms connected by dashed yellow lines are shown in Å. (B) Six different crystal forms of the RECQL5:nanobody complex with respective mutations on the nanobody scaffold are presented in three perspectives (top, front, and right views). RECQL5 molecules are in light-pink, and nanobody molecules are in marine and light-blue. C12 residues are presented as yellow dots on the structures and N12 residues are presented as red dots. Nb-α and C-C interfaces are indicated by dashed lines in the lattices. Resolution indicated here uses the criteria CC 1/2 > 0.3 from the ISPYB autoprocessing pipeline without any further data truncation.

From G1–G3 we already observed five different crystal forms (Figure 6B). Dimer P 1 2 1 and Dimer C 2 2 21 existed mostly under PEG conditions. Tetramer P 2 2 21 Porous/Compact and Dimer P 2 2 21 shifted mostly existed under high salt condition. In G4 and G5, we observed crystals in both high salt and PEG conditions. The high salt crystals of G4 and G5 were Tetramer P 2 2 21 Porous form, which we already observed in G3. The reason why G4 and G5 crystallized under drastically different conditions might be that it unlocked an additional crystal form Tetramer I 2 2 2/C1 2 1. Notably, this crystal form led to our best-diffracting data (Figure S5), indicating that crystal packing was also improved by the mutation T125M.

Among all six crystal forms, the Nb-α interface was present, and among all tetramer crystal forms, the C-C interface was present (Figure 6B). This indicated that these two interfaces are key, mediating crystal packing under certain conditions like the glueing of surfaces. We therefore termed the nanobody scaffolds with our modifications that enhance these two interfaces as ‘Gluebodies’ (Gbs). We also termed interacting residues in Nb-α as ‘Glue site I’ (Figures 4C and 6A) and interacting residues in the C-C interface as ‘Glue site II’ (Figure 6A).

### Transferable Gluebody Mutations Can Re-Establish Nanobody Crystallizability

Gluebody mutations have also yielded crystals for noncrystallizing proteins. For six proteins for which native nanobodies failed as crystallization chaperones, Gluebodies yielded a new, well-diffracting crystal form for one case that was known to crystallize (MPP8 protein) and informative crystallization outcomes for the others, ranging from no crystals to poorly diffracting or otherwise problematic crystals (Table 1, Figures S6 and S11, and Table S6).

**Table 1:** Experimental Details of the Crystallization Trials of Several Targets in Complex with Respective Gluebodies^a^

target	no. of Gluebodies tested	no. of crystal plates with Gluebodies	crystals with Gluebody	crystals with original nanobody	crystals without chaperone	comments
TUT4	5	20	6–8 Å	none	none	
WRN	8	18	**2 Å**	none	**2.2 Å** ^46^	single crystal, unreproducible, phasing failed
PRNP (PrP^C^)	1	2	6–8 Å	none	none	
MFS protein	2	12	no diffraction	7–10 Å	none	in lipidic cubic phase
MAGEC2	3	6	none	none	none	
MPP8	1	3	**2 Å**	none	**1.6 Å** ^53^	solvable data, albeit with pseudotranslation

a The WRN:Gluebody crystal diffracted to 2 Å, but the phasing failed, resulting in an unsolvable dataset.

The successful case, the MPP8:Gluebody complex, diffracted to 2 Å and the structure was readily determined. The Gluebody mutations M125 and K14 mediated the inter-Gluebody crystal contact and revealed yet another Gb-Gb interface that is not observed in any of the RECQL5:Gluebody crystal forms. This interface only involves three pairs of residues, too small to have been included in the Nb interface classification discussed earlier; notably, two of the three pairs involve modified residues, meaning they have a prominent role in the crystal contact. This interface would not have been compatible with a C-terminal His-tag, as it involves the extreme C-terminus of the nanobody (Figure S11).

The MPP8 chromodomain is a best-case scenario, where the Gluebody assists in finding new packing forms: the protein crystallizes well on its own but not with the wild-type nanobody; it requires the Gluebody mutations to generate the alternative crystal form that may be useful for screening purposes.

For the other targets tested, the associating interfaces of Gluebodies promote crystallogenesis in half of the challenging targets. In those cases, this was insufficient for structure solution, as the resolution and diffraction quality were too poor for phasing so that more target construct or crystallization screening is required. However, the important readout of those experiments is that the proteins are likely to take alternative packing patterns, which presumably provide more chances of crystallization than the original nanobody scaffold.

In summary, although Gluebody mutations are not a one-for-all solution for noncrystallizing proteins, we recommend that two Gluebody constructs (S7N:Q14K:T125 M and S7N:L12C:Q14K:T125M) are routinely tested along with the original nanobody scaffold, whether to increase the likelihood of identifying alternative crystal forms or to provide initial crystals and conditions for those reluctant to crystallize. Where feasible, combinatorial mutations based on the two Gluebody scaffolds may also be helpful (see the list of mutations in Figure S3B).

## Discussion

In this study, we show that the Gluebody method provides a route to novel and better-behaving crystal polymorphs by applying iterative engineering of the nanobody scaffold (Figure S5) to a model (RECQL5) protein target:nanobody system. The polymorphs are strikingly diverse, including a novel interface-driven tetramer lattice, despite involving only a single target protein. This contrasts strongly with the polymorphic behaviors of naïve nanobody scaffolds, as inferred from the PDB, where, for instance, the tetrameric nanobody pattern is present only twice (PDB IDs 6OS1 and 6OS2), notably with fewer interacting residues than in our Gluebody.

We demonstrated that transplanting all or some the recommended Gluebody modifications (Table 2) onto nanobodies with different CDRs, made crystallogenesis more likely, and was overall informative of the crystallizability of the target protein. While Gluebodies led to high-resolution structures for only crystallizing proteins and showed limited capability to rescue noncrystallizing proteins, especially intrinsically highly flexible or dynamic assemblies, they should provide better opportunities to crystallization via more crystallogenic target-independent interfaces. Guidelines for using Gluebodies on different targets are listed in Table 3.

**Table 2:** Recommended Gluebody Modifications^a^

recommended modifications	In Glue site I (Nb-α interface)?	In Glue site II (C-C interface)?	comment
L12C		√	This could lead to increased potential of forming crystal contacts (C-C interface) on this Gluebody surface by introducing polar interactions or disulfides.
T125M		√	This could improve the packing of the C-C interface, forming larger and better-diffracting crystals.
S7N	√		This could create more chances for an enhanced Nb-α interface as crystal contacts by creating more hydrogen bonds.
Q14K	√		This could create more chances for an enhanced Nb-α interface as crystal contacts by creating more hydrogen bonds or salt bridges.
C-terminal truncation		√	This could potentially expose Glue Site II for the formation of crystal contacts that are otherwise inapproachable.

a All numbering is in the IMGT format.

**Table 3:** Gluebody Usage Guidelines for Different Targets

target type	Gluebodies likely to help?	notes
small (<60 kDa), monomeric proteins	yes	Especially for rigid domains without oligomerization interfaces.
intrinsically disordered protein	no	The target itself is too flexible to form crystal lattice, and a single crystallogenic Gluebody interface is not sufficient to rescue.
membrane protein	maybe	Optimal binding pose of the Gluebody might lead to successful crystal packing while others cause difficulty.
oligomeric proteins	maybe	The Gluebody interface might be introducing a different oligomerizing symmetry than the target itself and interfere with crystal packing.

What was crucial in this engineering workflow was to monitor the ‘phenotypic’ properties salient to the problem, namely, diffraction quality and crystal packing, rather than the far more readily measured binding affinity that is typically used in binder selection studies. This approach was crucially enabled by state-of-the-art technologies in high-throughput protein purification, crystal harvesting, and X-ray diffraction in large synchrotrons that vitally streamlined iterative analyses of the hundreds of mutant crystal growth and diffractions that were required. It thus demonstrated that X-ray crystallography is sufficiently powerful to be used even as the sole methodology for iterative protein engineering.

Much of the experimental versatility needed in this workflow (high-throughput protein production, crystal harvesting and diffraction) can be achieved by reducing the protein consumption and fully exploiting the facilities of modern synchrotrons.^54,55^ For sufficient protein production, nanobodies are naturally high-yielding and only require 1–100 mL expression volumes for crystallization purposes. Thus, with commercially available high-throughput gene synthesis services, hundreds of mutants can be obtained quickly and conveniently. For the target protein, however, the yield can vary and might be very low. In this case, new technologies using microfluidics could minimize the protein consumption while maximizing the number of conditions or nanobody/Gluebody constructs for trials.^56^ While this is still not a widespread method, this may provide an approach to address the protein bottleneck. For diffraction experiments, there exists facilities such as Diamond Light Source with publicly accessible high throughput beamline I04-1, which can collect >1000 diffraction data within a week, and the synchrotron is currently constructing a new line increasing its capability by 10-fold. Therefore, more availability will be provided for high-throughput crystal screening and crystal engineering.

Nevertheless, Gluebodies can also feasibly be assessed in standard structural biology experiments, without a large-scale high-throughput engineering effort, applying just 1–3 key variants to available nanobodies (e.g., S7N:Q14K:T125M or S7N:L12C:Q14K:T125M).

Other biophysical methods (such as BLI, MST, and ITC) that may measure binding affinities and so provide insight into thermodynamics are neither direct nor necessarily relevant outputs. Here this is vitally relevant when preferred interfaces for better crystal packing involve transient and dynamic interactions within one type of protein molecule. In this way, X-ray crystallography has allowed the unique discovery of the preferred interface (see below). It also suggests that striving for affinity alone (i.e., thermodynamic measures) in chaperones is not necessarily the correct design approach.

A key mechanistic conclusion arises from the evolution of this engineering workflow, namely, that it is the “gluey” interfaces that drive the oligomerization, here provided by crystallization. First, by introducing S7N Glue Site I mutations (Figures 1, 4C, and 6A) crystallization is driven by the *in situ* formation of Gb dimers. Second, our data reveal a key role for the Gb C-terminus as an emergent interface (Glue site II) that drives polymorphism. Analyses reveal that our discovery is consistent with missing interfaces in current structures; the PDB reveals a trend of C-terminal tags attached (63% of 335 crystal structures in the PDB have more than five residues at their C-terminal sequence). As we explicitly show here, such ‘overhang’ residues hinder both the formation of the Gb C-C interface and impede the tetrameric assembly form by occluding C12. The combination of free C-terminal tail, Q14K, and Glue Site II mutations (L12C, T125M) (Figures 1 and 6A) trigger tetrameric crystallization polymorphs. This arises through a combination of glue sites as preferred interfaces: dimerization of diGb gives tetraGb *in crystallo* (Figure 6B).

Clearly other favored interfaces are feasible, given that not only dimers but also tetramers were formed *in crystallo*. In particular, this *in crystallo* oligomerization could be achieved in solution through chemically controlled covalent bond formation. One such constellation is observed in the tetrameric crystallization pattern with four Cys residues at the C-C interface core, where there is clear electron density between the two sulfur atoms in each pair of Cys residues (Figure S10). Understanding this requires further exploratory work: for instance, we were unable to fully model the atomic constellation, since the distances between the pairs of sulfur atoms were 2.8 Å, which is much longer than a standard disulfide bond (∼2.05 Å^57^). Nevertheless, it indicates that covalent kinetic trapping in solution is feasible because that may well also have occurred here prior to crystallization or as part of the process.

The rigidity supplied by such covalency can further be exploited in scaffolds that combine the advantages of Gbs but with symmetry also introduced. Indeed, where sufficient rigidity can be achieved, it provides a modular ‘plug-and-play’ tool that achieves protein assemblies without protein fusions via molecular biology, an application we demonstrated in cryo-EM,^58^ where we named them Gembodies to reflect these additional functionalities of deliberate covalency and symmetry.

## Methods

### Crystal Contact Analysis of Structures Containing Nanobodies in PDB

Crystal structures were fetched and analyzed using the open-source PYMOL package in Python.^59^ The workflow of analysis is described in the results and shown in detail in Figure S4. Finally, a few interfaces that fell into the noise category were manually inspected and added to respective categories resulting in the final classification.

### Expression and Purification of RECQL5

The truncated RECQL5 protein (11–453) was subcloned into the pNIC-Bsa4 vector with a TEV cleavable 6xHIS tag at the N terminus. The protein was expressed using BL21-DE3-pRARE strain in autoinduction TB media (Formedia) containing Kanamycin and 0.01% antifoam 204 at 37 °C for 5.5 h followed by 40–44 h at 18 °C. The base buffer we used for purification contained 5% glycerol, 10 mM HEPES (pH 7.5), 500 mM NaCl, and 0.5 mM TCEP. Bacteria were harvested by centrifugation at 4000 g and resuspended in 3 times volume of base buffer + 30 mM Imidazole, 1% Triton, 0.5 mg/mL lysozyme, 10 μg/mL homemade benzonase, followed by storage in −80 °C freezer overnight for complete cell lysis. The purification started the next day with thawing the frozen pellets in a room-temperature water bath, followed by centrifugation at 5000 g for 1 h to obtain clear supernatant. The supernatant was then applied to Ni-NTA prepacked columns (GE Healthcare) pre-equilibrated with base buffer + 30 mM imidazole. After thoroughly washing the Ni-NTA columns with base buffer, protein was eluted using 2.5 mL of base buffer + 500 mM imidazole directly into base buffer equilibrated PD-10 columns (GE Healthcare). Then, 3.5 mL of base buffer was then applied to PD10 columns to elute the RECQL5 protein in base buffer. Then TEV protease was added to the protein solution with a 1:10 mass ratio for overnight incubation, and 20 mM imidazole was also added in the solution. The next day, twice the quantity of Ni-NTA columns were pre-equilibrated with base buffer + 20 mM imidazole, and the RECQL5 solution with TEV was applied to the columns to get rid of RECQL5 with uncleaved 6xHIS tag, TEV protease, and contaminants. Flow-through fractions were collected and flash frozen for making nanobody complexes.

### High-Throughput Cloning, Expression, and Purification of Nanobody Variants

Wild-type anti-RECQL5 nanobody was discovered in the Instruct-ERIC infrastructure following established protocols described previously.^38^ The immunizations of alpaca for the RECQL5 wild-type nanobody were conducted in the Instruct-ERIC (PID6873), part of the European Strategy Forum on Research Infrastructure (ESFRI), and the Research Foundation – Flanders (FWO), and strictly followed the EU animals health legislation (ELI: http://data.europa.eu/eli/reg/2016/429/oj). All experiments were carried out locally.

The nanobody constructs of G0-002 to G0-074 and G1-001 were generated by the site-directed mutagenesis method based on G0-001 using pNIC-CTH0 as the vector plasmid. The nanobody construct of G2-001 was generated by the mutagenesis method based on G1-001 using pNIC-CTH0 as the vector plasmid. The nanobody constructs of G1-002 to G1-091 were generated by the mutagenesis method based on G1-001 using pNIC-CTBH as the vector plasmid. The nanobody constructs of G2*-005 to G2*-016 were generated by mutagenesis method based on G2*-004 using pNIC-MBP2-LIC as the vector plasmid and G2-002 to G2-013 using pNIC-CTBH as the vector plasmid with the same method and template DNA. G3*-001 to G3*-019, G3*-020 to G3*-038, G3*-039 to G3*-057, G3*-058 to G3*-076, and G3*-077 to G3*-095 were generated using mutagenesis based on templates G3-048, G3-055, G3-052, G3*-011, and G3*-050, respectively. G3-001 to G3-096 and G2*-001 to G2*-004 were directly synthesized as DNA fragments and subcloned into pNIC-MBP2-LIC vectors. G4-001 to G4-087 and G5-001 to G5-009 were also based on the synthesized DNA fragments using a different set of primers in the PCR step. All cloning procedures followed ligation independent cloning described previously.^9,60^

Constructs of nanobody variants were transformed into a single-step (KRX) *E. coli* strain (Promega) at the end of molecular cloning. Single colony was inoculated into 1 mL of LB medium (Merck) and incubated overnight. Starter culture of each nanobody was then inoculated into 100 mL of autoinduction media (Formedia) containing 0.1% Rhamnose (Sigma), 0.01% antifoam 204 (Sigma), and 0.05 mg/ml Kanamycin (Sigma) at 37 °C for 5.5 h followed by 40–44 h at 18 °C. The base buffer we used for purification contained 5% glycerol, 10 mM HEPES (pH 7.5), 500 mM NaCl, and 0.5 mM TCEP. Bacteria for each nanobody were harvested at 4000 g and resuspended in 10 mL of base buffer + 30 mM imidazole, 1% Triton, 0.5 mg/mL lysozyme, and 10 μg/mL homemade benzonase, followed by storage in −80 °C freezer overnight for complete cell lysis. The purification started the next day with thawing the frozen pellets in a room temperature water bath, followed by centrifugation at 5000 g for 1 h to obtain clear supernatant. The supernatant for each nanobody was then applied to one 1 mL Ni-NTA prepacked column (GE Healthcare) pre-equilibrated with base buffer + 30 mM imidazole. After thoroughly washing the Ni-NTA columns with base buffer, the nanobody was eluted using 2.5 mL of base buffer + 500 mM imidazole directly into base buffer equilibrated PD-10 columns (GE Healthcare). Then, 3.5 mL of base buffer was then applied to PD10 columns to elute the nanobody protein base buffer without imidazole. Then TEV protease was added to the protein solution with a 1:10 mass ratio for overnight incubation, and 10 mM imidazole was also added in the solution. The next day, two Ni-NTA columns were pre-equilibrated for each nanobody with base buffer + 10 mM imidazole, and the nanobody solution with TEV was applied to the columns to get rid of nanobody with uncleaved tags, TEV protease, and contaminants. Flow-through fractions were collected, and 2 mL of base buffer was applied to the Ni-NTA columns to flush all nanobody through. The collected fractions were ready for complex preparation.

### Dehydroalanine Formation

To a solution of G5-007 nanobody (1 mL, 1 mg/mL, 72 μM) in Na_2_HPO_4_ buffer (50 mM, pH 8.0), 0.28 mg (25 equiv) of DTT were added. The solution was shaken at room temperature for 20 min and then the protein was treated with 22 μL of 0.5 M DBHDA (2,5-dibromohexanediamide) in DMSO (500 eq.) and heated to 37 °C for 4 h, at which point analysis by mass spectrometry showed reaction completion. The protein was purified using a PD Miditrap G-25 column (Cytiva no. 28918008) pre-equilibrated with Na_2_HPO_4_ buffer (50 mM, pH 8.0).

### Crystallization of RECQL5:Nanobody Complex Variants and X-ray Diffraction

Purified RECQL5 and nanobody were mixed at a ratio of 1:1.5 and concentrated using a 10 kDa concentrator (Amicon and Corning), followed by Sepax SRT SEC-300 HPLC or Superdex-200 SEC (Cytiva). The peak fractions containing both RECQL5 and nanobody were pooled and concentrated to certain extent (Table S3) for crystallization trials. Crystal plates were set up by Mosquito (model no. TC1100-1100, TTP Labtech) using Swiss-CI 3- drop plates with precipitants from the Hampton Index screen (HIN3 HT-96, Molecular Dimensions). Plates were sealed and sent into Formulatrix imager (model no. R1-1000) and imaged at 12 h, 1 day, 4 days, 7 days, 14 days, 28 days, and 56 days. Crystals were harvested using Shifter^61^ with 17% glycerol for high salt conditions or ethylene glycol for PEG conditions added as cryo-protectant, then snap frozen and shipped to multiple beamlines at Diamond Light Source for X-ray diffraction screening and data collection.

### Structure Determination of RECQL5:Nanobody Complex Variants

Diffraction data were processed by multiple autoprocessing (including Xia2 and Autoproc) pipelines on ISPYB.^62–64^ The high-resolution cutoff was determined by a CC 1/2 above 0.3. The resolution values used in the analysis were the results of the Xia2 processing pipeline. Data sets were truncated using Aimless in CCP4-i suite.^65^ Phasing of RECQL5 was done by Phaser,^66^ using the D1 and D2 domains of RECQCL5 (PDB ID 5LB8) as search models. The search model for phasing the nanobody was chain A of 2X1O. Further refinement was done by Coot and Refmac5 in CCP4-i suite.^67,68^

### Purification and Structural Determination of MPP8:Gluebody Complex

The region of MPP8 corresponding to the chromodomain (residues 53–117) was expressed as an N-terminally His-tagged protein in *E. coli* BL21­(DE3)­RIL cells using a modified pET30 vector. Cells were lysed by sonication, and the protein purified by nickel affinity chromatography from clarified lysate prior to cleavage of the His-tag using TEV protease overnight. The cleaved protein was repurified by nickel affinity chromatography and subjected to size exclusion chromatography. A construct of the Nb 3A02 nanobody incorporating the Gluebody mutations S7N, Q14K, and T125 M (S8N, Q14K, and T117 M in the original non-IMGT sequence) and carrying an N-terminal TEV cleavable His-tag following the pelB leader sequence was made in the phagemid vector pBLIP1. This 3A02 gluebody was expressed in the periplasm of *E. coli* SS320 cells, purified from periplasm extract by nickel affinity chromatography, then cleaved, and repurified as described for the MPP8 protein. For isolation of the MPP8/3A02 gluebody complex for crystallization, the proteins were combined at a 1:1.2 molar ratio and the mix subjected to gel filtration on a Superdex 75 16/600 column (Cytiva) into 20 mM Hepes pH 7.5, 80 mM NaCl. An upshifted peak was observed for the complex (Figure S11), and the fractions corresponding to this peak were concentrated to ∼12 mg/mL. The crystals used for structure determination were obtained within a few days by vapor diffusion at 18 °C using a 1:1 mix of the protein with a reservoir solution of 100 mM sodium acetate and 25% PEG 3000 and were frozen in reservoir solution supplemented with 22% glycerol.

Two diffraction data sets at a resolution of 2.01 Å were collected from a single crystal at Diamond I24 and were combined and processed with xia2.multiplex.^69^ In these data, and all other data sets obtained, pseudotranslation was identified between molecules in the lattice related by noncrystallographic symmetry; this was mitigated by application of a pseudotranslation vector (0.000, 0.265, 0.500) in Molrep.^70^ The data were initially phased by molecular replacement using Molrep with a nanobody scaffold model, then iterative cycles of refinement in Refmac, model building in Coot,^71^ and molecular replacement in Molrep were carried out until the MPP8 molecule and the CDR regions of the nanobody were complete. Further rounds of model optimization and refinement were carried out using Coot and Phenix.refine.^72^

## Supplementary Material





## Data Availability

The structures of RECQL5 in complex with engineered nanobodies are deposited in the Protein Data Bank (PDB), with PDB IDs listed as follows: G1-001, 7ZML; G2-001, 7ZMM; G3-048, 7ZMN; G3-052, 7ZMO; G3-055, 7ZMP; G2*-006, 7ZMQ; G2*-011, 7ZMR; G4-043, 7ZMS; G5-006, 7ZMT; G5-006, 7ZMV. The structure of MPP8 in complex with engineered nanobodies is deposited in the PDB under the ID 9H77. The PDB IDs of structures used in the nanobody interface analysis are listed as follows: 2X1O, 2X1P, 2X1Q, 2X6M, 2X89, 2XT1, 2XV6, 2XXC, 2XXM, 3CFI, 3DWT, 3EAK, 3EBA, 3EZJ, 3G9A, 3K1K, 3K74, 3OGO, 3P0G, 3SN6, 3STB, 3ZKQ, 3ZKS, 4AQ1, 4BEL, 4BFB, 4C57, 4C58, 4C59, 4CDG, 4EIG, 4EIZ, 4EJ1, 4FHB, 4GFT, 4GRW, 4HEM, 4HEP, 4I0C, 4I13, 4I1N, 4IOS, 4JVP, 4KML, 4KRL, 4KRM, 4KRN, 4KRO, 4KRP, 4LDE, 4LDL, 4LDO, 4LGP, 4LGR, 4LGS, 4LHJ, 4LHQ, 4MQS, 4MQT, 4N9O, 4OCL, 4OCM, 4OCN, 4P2C, 4QGY, 4QKX, 4QLR, 4QO1, 4S10, 4S11, 4W6W, 4W6X, 4W6Y, 4WEM, 4WEN, 4WEU, 4WGV, 4WGW, 4X7C, 4X7D, 4X7E, 4X7F, 4XT1, 4Y8D, 4Z9K, 4ZG1, 5BOP, 5BOZ, 5C1M, 5C2U, 5C3L, 5DA0, 5DA4, 5DFZ, 5DXW, 5000, 5E0Q, 5E5M, 5E7B, 5E7F, 5F1K, 5F1O, 5F21, 5F7K, 5F7L, 5F7M, 5F7N, 5F7W, 5F7Y, 5F8Q, 5F8R, 5F93, 5F97, 5F9A, 5F9D, 5FWO, 5G5R, 5G5X, 5GXB, 5H8D, 5H8O, 5HDO, 5HGG, 5HVF, 5HVG, 5HVH, 5IMK, 5IML, 5IMM, 5IMO, 5IP4, 5IVN, 5IVO, 5JA8, 5JA9, 5JDS, 5JQH, 5LHN, 5LHP, 5LHQ, 5LHR, 5LMJ, 5LMW, 5LZ0, 5M13, 5M14, 5M15, 5M2M, 5M2W, 5M30, 5M7Q, 5M94, 5M95, 5MJE, 5MP2, 5MWN, 5MY6, 5MZV, 5NBD, 5NBL, 5NBM, 5NLU, 5NLW, 5NM0, 5NML, 5NQW, 5O02, 5O03, 5O04, 5O05, 5O0W, 5O2U, 5O8F, 5OCL, 5OJM, 5OMM, 5OMN, 5OVW, 5TD8, 5TJW, 5TOJ, 5TOK, 5TP3, 5U64, 5U65, 5UK4, 5UKB, 5USF, 5VAK, 5VAN, 5VL2, 5VLV, 5VM0, 5VM4, 5VM6, 5VNV, 5VNW, 5WB1, 5WB2, 5Y7Z, 5Y80, 6B20, 6B73, 6C5W, 6C9W, 6DBA, 6DBD, 6DBE, 6DBF, 6DBG, 6EHG, 6EQI, 6EY0, 6EY6, 6EZW, 6F0D, 6FE4, 6FUZ, 6FV0, 6GZP, 6H02, 6H15, 6H16, 6H1F, 6H6Y, 6H6Z, 6H70, 6H71, 6H72, 6H7J, 6H7L, 6H7M, 6H7N, 6H7O, 6I6J, 6IBL, 6MXT, 6QTL, 6R7T, 6O3C, 6OS0, 6OS1, 6OS2, 6OYH, 6OYZ, 6OZ6, 6Q6Z, 6QD6, 6QGW, 6QGX, 6QGY, 6QPG, 6QUZ, 6QV0, 6QV1, 6QV2, 6QX4, 6RNK, 6RTW, 6RTY, 6RU3, 6RU5, 6RUL, 6RUM, 6RUV, 6RVC, 6S0Y, 6SGE, 6SSI, 6SSP, 6TEJ, 6TYL, 6SWR, 6U12, 6U14, 6U50, 6U51, 6U52, 6U53, 6U54, 6U55, 6VI4, 6WAQ, 6WAR, 4DK3, 4DK6, 4DKA, 4KDT, 4KSD, 4PIR, 5J1S, 5J1T, 5M2I, 5M2J, 6GJQ, 6GJS, 6GJU, 6GK4, 6GKD, 6GWN, 6GWP, 6GWQ, 6HD8, 6HD9, 6HDA, 6HDB, 6HDC, 6HEQ, 6HER, 6HHD, 6HHU, 6HJX, 6HJY, 6I2G, 6I8G, 6I8H, 6IBB, 6ITP, 6ITQ, 6JB2, 6JB5, 6JB8, 6JB9, 6DO1, 6DYX, 6F2G, 6F2W, 6FPV, 6GCI, 6GS1, 6GS4, 6GS7, 6N4Y, 6N50, 6NFJ.

## Notes

Figure S1, size exclusion chromatography profiles of each target protein; Figure S2, the roadmap of the several iterations of mutagenesis on RECQL5 nanobody scaffold; Figure S3, the crystal epitope mutation D69Y on the scaffold that rescues the RECQL5 nanobody to be effective again as a crystal chaperone; Figure S4, the computational workflow of nanobody–nanobody crystal contact analysis and classification results; Figure S5, resolution comparison of different crystal forms from RECQL5:nanobody complexes; Figure S6, crystals from PRNP, WRN, TUT4, and MFS protein in complex with respective Tetra-Gluebodies; Figure S7, chemical conversion of the Cys12 on G5-007 nanobody to dehydroalanine (Dha); Figure S8; diffraction quality of G5-006 and G5-009 soaked in different cryo-protectants; Figure S9, tilted back-to-back packing of nanobody dimers from PDB ID 6QGY; Figure S10, electron density implying possible bonds between the sulfur atoms in the C-C interface core; Figure S11, successful crystallization of MPP8:Gb complex; Table S1, the crystallization experiment on a selection of target:nanobody pairs with naïve nanobody scaffolds; Table S2, detailed DBSCAN result with full list of PDB codes of each nanobody–nanobody interface category (in the ‘Pattern name’ column, the first four letters represent PDB and the digit after ‘_’ represents the no. of the pattern in the structure, and in the ‘Pattern’ column, each number represents the IMGT normalized index of a residue of the nanobody scaffold involved in the pattern); Table S3, full list and the crystallization result of nanobody variants tested in the RECQL5:nanobody system; Table S4, full list of diffraction data collected from crystals of all nanobody variants tested in the RECQL5:nanobody system (resolution indicated here uses the criteria CC 1/2 > 0.3 from the ISPYB autoprocessing pipeline without any further data truncation); Table S5, detailed information with numeric values of each cell in Figure 5A; Table S6, data collection and refinement statistics of MPP8 in complex with the gluebody (PDF)
Transparent Peer Review report available (PDF)

## Notes

All related codes for nanobody crystal contact analysis are accessible on Zenodo (https://zenodo.org/records/12782755).

## ∇

The Walter and Eliza Hall Institute of Medical Research, 1G, Royal Parade, Parkville, Victoria 3052, Australia

## ○

Bicycle Therapeutics Plc, Cambridge CB21 6GS, U.K.

## ●

Evotec, Ltd., Oxford OX14 4RY, U.K.
