# Supplementary material for: Gluebodies Offer a Route To Improve Crystal Reliability and Diversity through Transferable Nanobody Mutations That Introduce Constitutive Close Contacts
Source: ACS Cent Sci. 2025 Oct 27;11(12):2385–99. doi: 10.1021/acscentsci.5c00937 (PMC12746149; doi:10.1021/acscentsci.5c00937)
Supplement: Supplementary file 1 [file oc5c00937_si_001.pdf]

Supporting Information

Gluebodies offer a route to improve crystal reliability and diversity through transferable nanobody mutations that introduce constitutive close contacts

Mingda Ye[1,*], Mpho Makola[1], Mark W. Richards[2], Joseph A. Newman[1], Michael Fairhead[1], Selena G. Burgess[2], Zhihuang Wu[2], Elizabeth Maclean[1,3], Nathan D. Wright[1], Lizbé Koekemoer[1], Andrew Thompson[1,4], Gustavo A. Bezerra[1,5], Gangshun Yi[3,14], Huanyu Li[1], Victor L. Rangel[6,7], Dimitrios Mamalis[8,9], Hazel Aitkenhead[1], Benjamin G. Davis[8,9,10], Robert J.C. Gilbert[3,11], Katharina L. Duerr[12], Richard Bayliss[2], Opher Gileadi[13] and Frank von Delft[1,14,15,16*]

1. Centre for Medicines Discovery, Nuffield Department of Medicine, University of Oxford, Oxford, OX3 7FZ, UK,
2. Astbury Centre for Structural Molecular Biology, School of Molecular and Cellular Biology, Faculty of Biological Sciences, University of Leeds, Leeds LS2 9JT, UK.
3. Division of Structural Biology, Wellcome Centre for Human Genetics, University of Oxford, Roosevelt Drive, Oxford OX3 7BN, UK
4. Present address: The Walter and Eliza Hall Institute of Medical Research, 1G, Royal Parade, Parkville, Victoria, 3052, Australia
5. Present address: Bicycle Therapeutics Plc, Cambridge, CB21 6GS, UK
6. Laboratory of Protein Crystallography, School of Pharmaceutical Sciences of Ribeirão Preto, University of São Paulo, Ribeirão Preto, São Paulo, 05508-000, Brazil
7. Present address: Evotec Ltd, Oxford, OX14 4RY, UK
8. Department of Chemistry, University of Oxford, Oxford, OX1 3TA, UK
9. The Rosalind Franklin Institute, Oxfordshire, Oxford, OX11 0QS, UK
10. Department of Pharmacology, University of Oxford, Oxford, OX1 3QT, UK
11. Calleva Research Centre for Evolution and Human Sciences, Magdalen College, University of Oxford, Oxford OX1 4AU, UK
12. Kavli Institute for Nanoscience Discovery, Department of Chemistry, University of Oxford, Oxford, OX1 3QU UK
13. SGC Karolinska Center for Molecular Medicine, Karolinska University Hospital, 171 76 Stockholm, Sweden
14. Diamond Light Source, Harwell Science and Innovation Campus, Didcot, OX11 0DE, UK
15. Research Complex at Harwell, Harwell Science and Innovation Campus, Didcot, OX11 0FA, UK.
16. Department of Biochemistry, University of Johannesburg, Auckland Park, 2006, South Africa

*Correspondence to M.Y. (martin.ye@cmd.ox.ac.uk), F.V.D. (frank.vondelft@cmd.ox.ac.uk)

Supplementary figures

Figure S1 Size exclusion chromatography profiles (left) and SDS-PAGE images (right) for each target protein. The red arrows show the protein peak fractions collected for crystallization trials. '*' indicates contaminants in the protein samples.

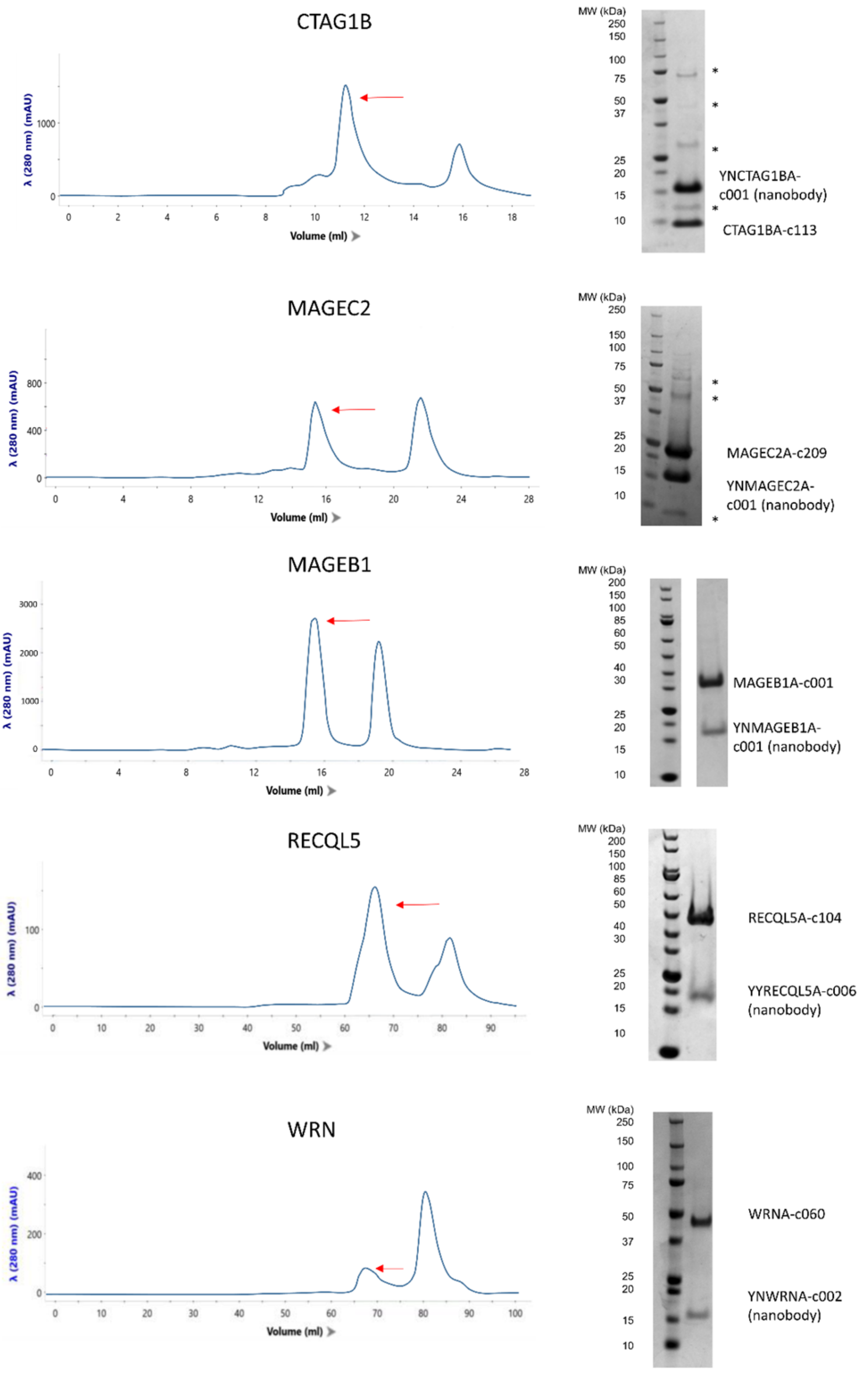

Figure S2 The road map of the several iterations of mutagenesis on RECQL5 nanobody scaffold. Variants in '[]' are selected crystal epitope mutants and in '()' are selected Nb-α mutants for subsequent iterations of mutagenesis.

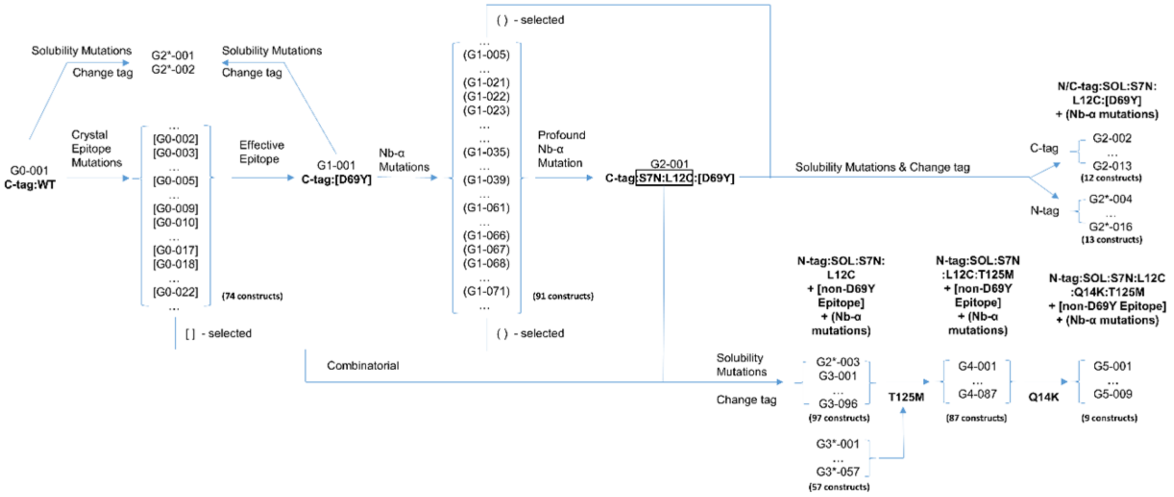

Figure S3 The crystal epitope mutation D69Y on the scaffold rescues the RECQL5 nanobody to be effective again as a crystal chaperone. A) The crystal epitope sites on the surface of a RECQL5 nanobody (C-tag:WT) are coloured in green, yellow and cyan. CDR regions are coloured in light brown. B) The combinatorial mutation designs. 22 single site mutations were chosen from the list of mutations provided by the crystal epitope server. 11 of the single site mutations were then selected to generate double-site and triple-site mutations thereafter, which resulted in 74 nanobody variants for crystallization trials. Colours are consistent with the sub-figure A. C) The crystal epitope variants that yielded crystals in the Hampton Index 3 Screen. All effective nanobody variants shared the D69Y mutation, highlighted in red. D) G1-001 crystal. E) G0-057 crystals.

A

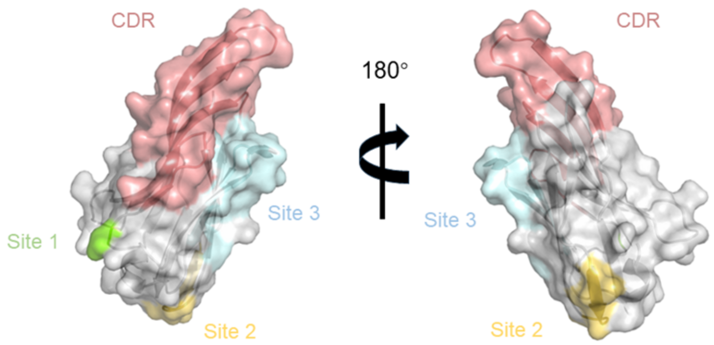

B

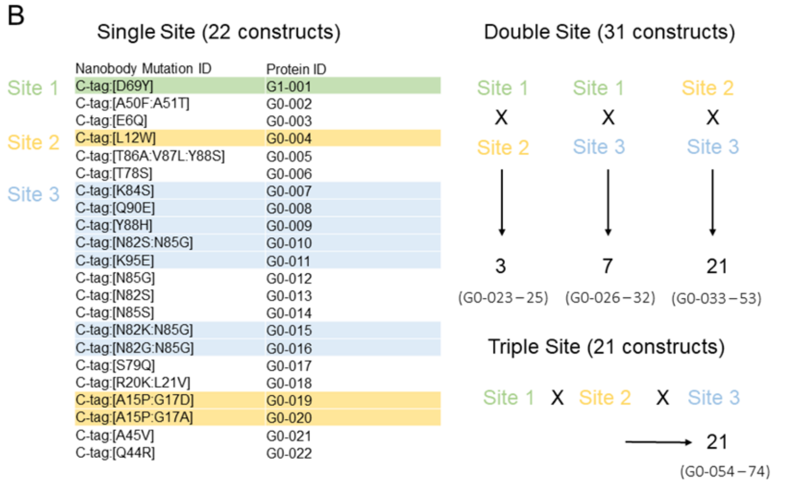

C

Nanobody mutation ID	Nanobody Grouped ID	Concentration (mg/ml)	Drops with mountable crystals	Drop with crystalline species
C-tag:[D69Y]	G1-001	11.46	1	1
C-tag:L12W:[D69Y:K84S]	G0-054	10.43	6	6
C-tag:L12W:[D69Y:Y88H]	G0-056	10.15	2	19
C-tag:L12W:[D69Y:N82S:N85G]	G0-057	14.05	4	10
C-tag:L12W:[D69Y:N82G:N85G]	G0-060	16.70	0	4

D

E

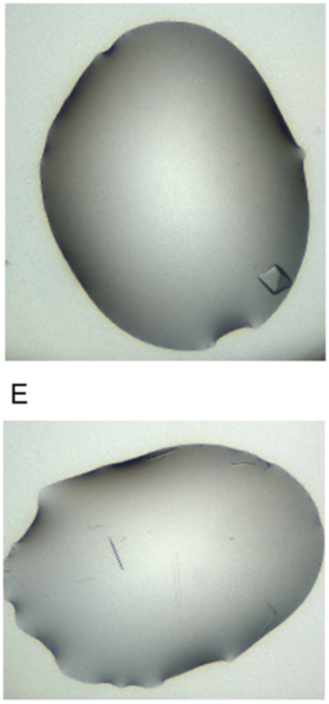

Figure S4 The computational workflow of nanobody-nanobody crystal contact analysis and classification results. The texts in red represent actions taken in each step. Structure cartoons in marine and light blue represent two nanobody molecules participating in the interface. Residues represented as sticks are interacting residues of the current nanobody molecules. Simplified nanobody sketches are shown at the bottom corners, and are consistent with Figure 2. Class 8 interfaces are between fusion parts of the nanobody and therefore are not counted as nanobody-nanobody interfaces.

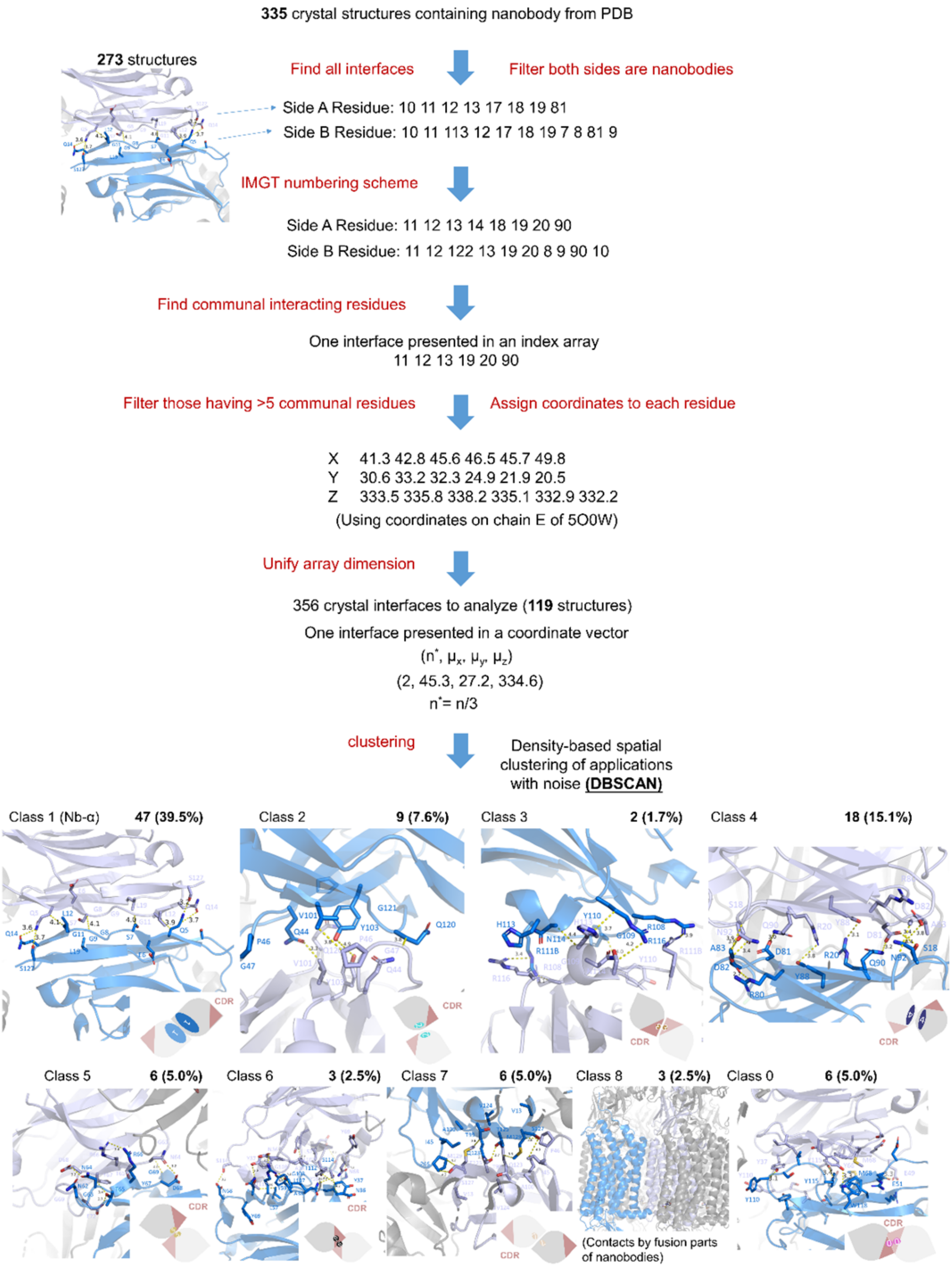

Figure S5 Resolution comparison of different crystal forms from RECQL5:nanobody complexes. Each combination of crystal forms and crystallization condition categories are grouped and indicated in different colors. The crystals are not in fragment-soaking conditions. Resolution indicated here uses the criteria CC ½ > 0.3 from the ISPYB auto-processing pipeline without any further data truncation.

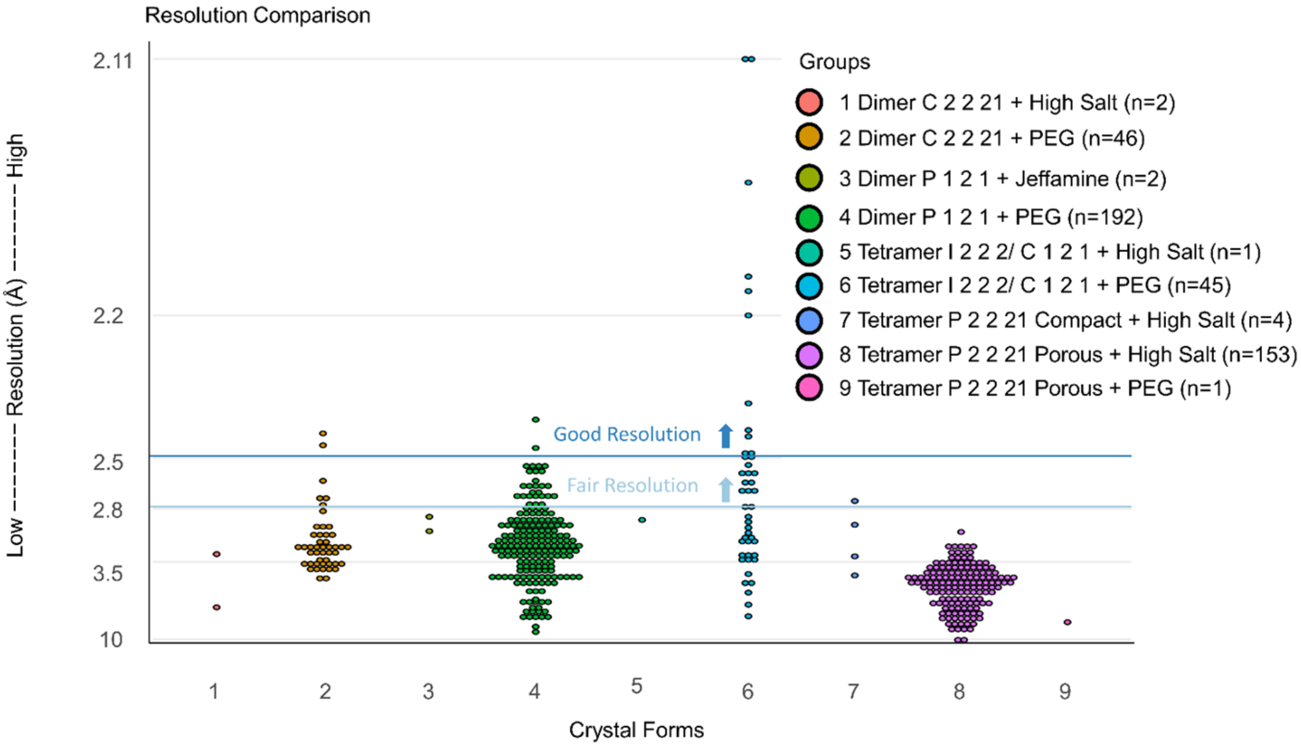

Figure S6 Crystals from PRNP, WRN, TUT4 and MFS protein in complex with respective Tetra-Gluebodies. There are visible and UV images for crystals and SDS-PAGE, SEC profiles showing the quality of the protein. The protein peaks in SEC collected for crystallization trials are indicated by the red arrows. Crystals are indicated by orange arrows. A) PRNP:Gluebody complex crystals. Crystallization happened at 14 mg/ml, 20 °C in the condition of 0.2M Magnesium chloride hexahydrate, 0.1M tris pH 8.5 25% w/v PEG3350, with a protein:precipitant ratio of 1:2. B) WRN:Gluebody complex crystals. Crystallization happened at 27.3 mg/ml, 20 °C in the condition of 0.15 M potassium bromide, 30% w/v polyethylene glycol monomethyl ether 2,000, with a protein:precipitant ratio of 1:2. C) TUT4:Gluebody complex crystals. There are several conditions yielding crystals. In the visible image on the left, the condition is 0.2 M magnesium chloride hexahydrate, 0.1 M BIS-TRIS pH 6.5, 25% w/v polyethylene glycol 3,350, with a protein:precipitant ratio of 1:2. In the visible image on the right, the condition is 0.1 M HEPES pH 7.5, 2.0 M ammonium sulfate, with a protein:precipitant ratio of 1:2. All crystallization happened at 14 mg/ml, 20 °C. D) The LCP crystals formed in the condition of 0.1M NaCl 0.1M Li_2SO_4 40%v/v PEG200 0.1M MES pH 6 at 40 mg/ml, 20 °C.

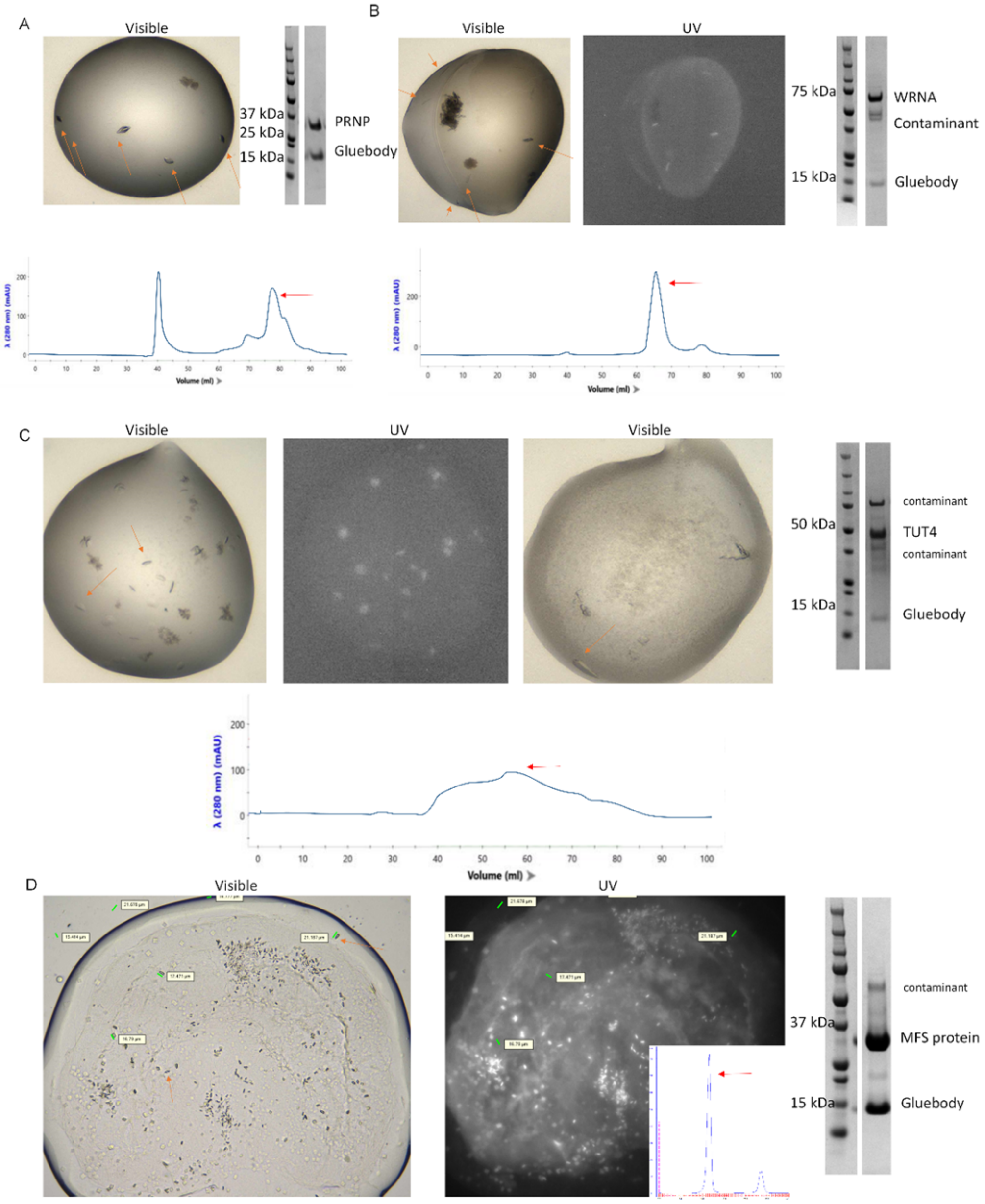

Figure S7 Chemical conversion of the Cys12 on G5-007 nanobody to dehydroalanine (Dha). To a solution of G5-007 nanobody (1 mL, 1 mg/mL, 72 µM) in Na_2HPO_4 buffer (50 mM, pH 8.0), 0.28 mg (25 eq.) of DTT were added. The solution was shaken at room temperature for 20 min and then the protein was treated with 22 µL of 0.5M DBHDA (2,5-dibromohexanediamide) in DMSO (500 eq.) and heated to 37 °C for 4 h, at which point analysis by mass spectrometry showed reaction completion. The protein was purified using a PD Miditrap™ G-25 column (Cytiva #28918008) pre-equilibrated with Na_2HPO_4 buffer (50 mM, pH 8.0).

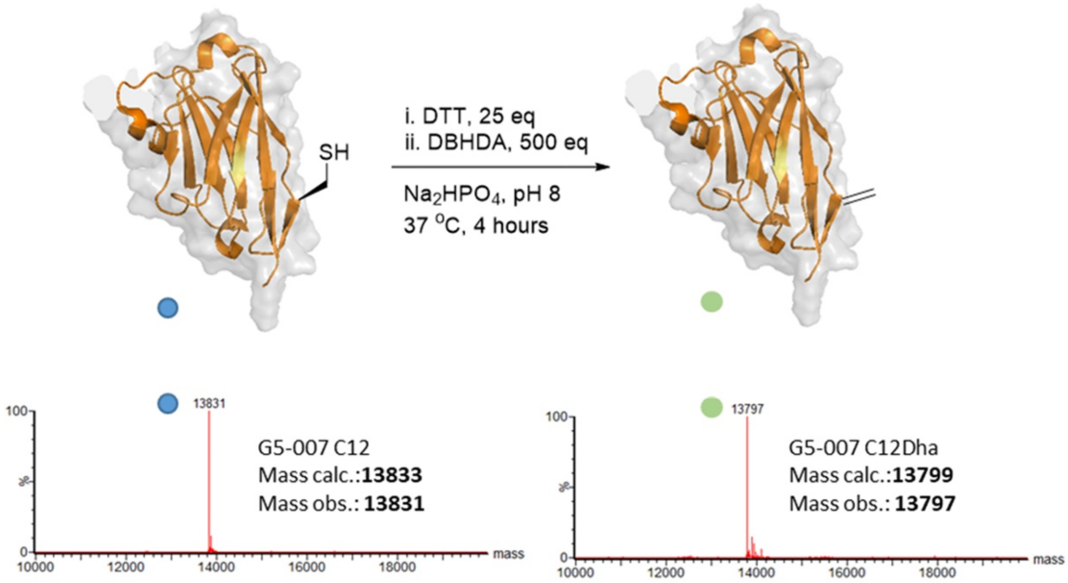

Figure S8 Diffraction quality of G5-006 (A) and G5-009 (B) soaked in different cryo-protectants, shaded according to diffraction resolution. In the bar plot, deep blue fractions represent good resolution (<2.5 Å) crystal percentage, blue bar heights represent fair resolution (<2.8 Å) crystal percentage and the total heights of the bars represent percentage of crystals that diffracted. The numbers of crystals, nanobody variants and soaking conditions in each group are indicated in the table below each bar.

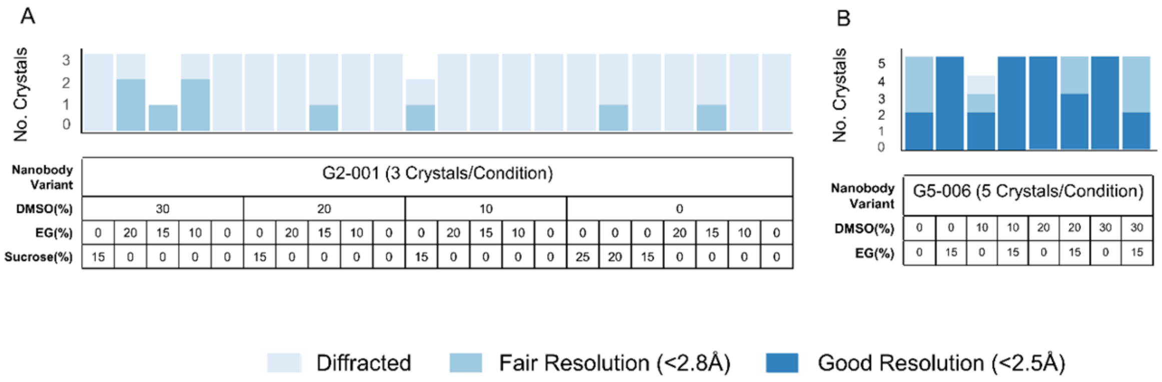

Figure S9 Tilted back-to-back packing of nanobody dimers from PDB id 6QGY. The C12 is replaced by M12, resulting in the tilted packing. Nb-α is also present in this crystal lattice. Molecules colored in marine and light blue are nanobody molecules. Nb-α is colored pink. The target protein molecules of the nanobodies are colored grey.

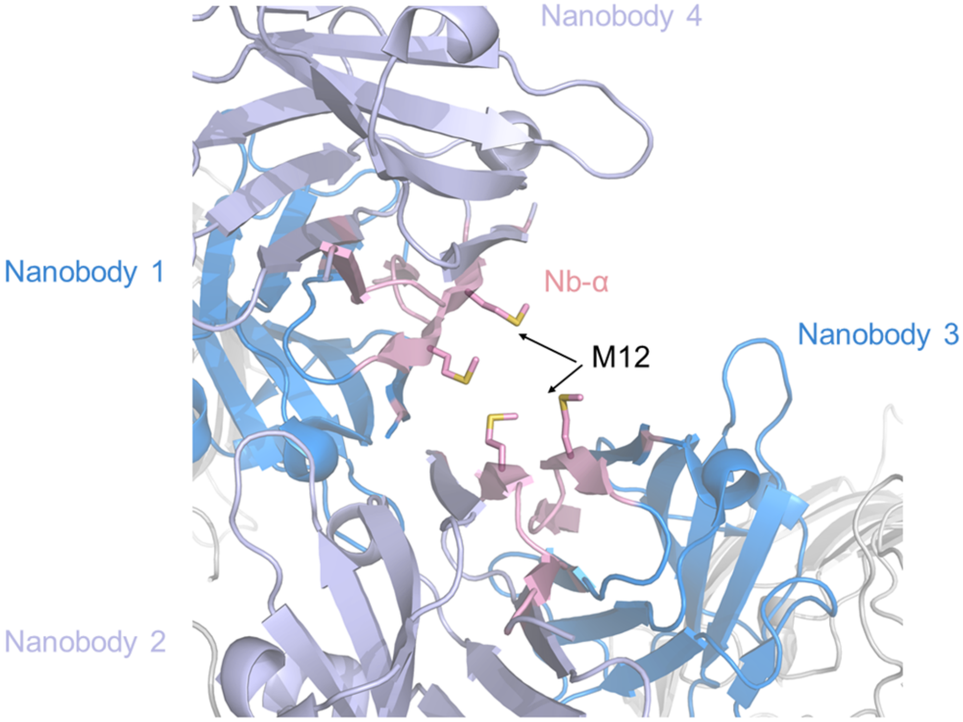

Figure S10 Electron density (pointed to by yellow arrows) implies possible bonds between the sulfur atoms in the C-C interface core. C12 and M125 are presented in stick and the sulfur atoms are colored yellow. The electron density around C12 is indicated in light blue mesh with the contour levels at 1σ, 2σ and 3σ, viewed from the top and the side.

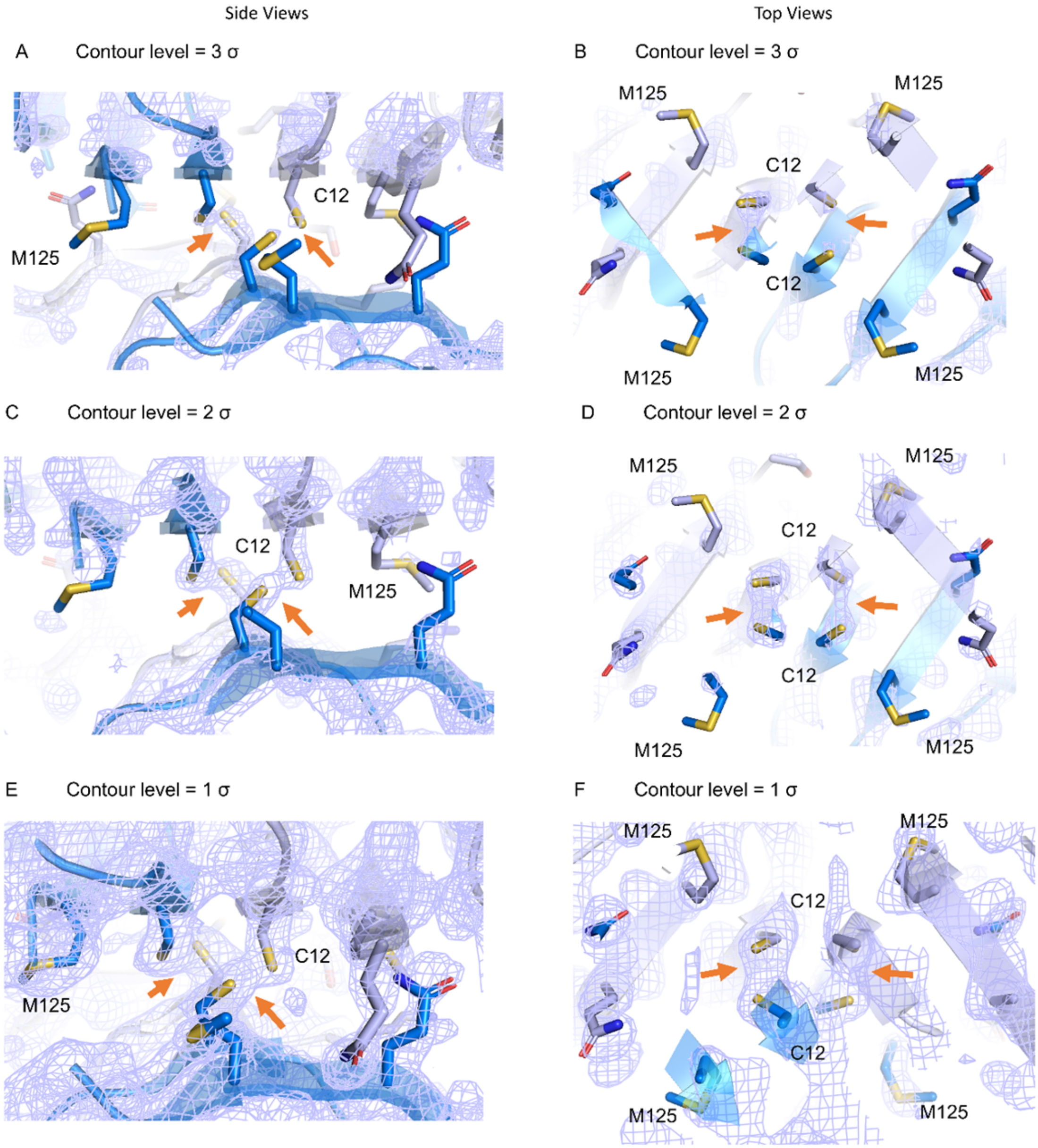

Figure S11 Successful crystallization of MPP8:Gb complex. (A) Size exclusion chromatography trace and SDS-PAGE gel of MPP8 chromodomain in complex with wild type Nb 3A02 and Gb 3A02 harbouring S7N, Q14K and T125M Gluebody mutations (S8N, Q14K and T117M in the original non-IMGT sequence) and without a C-terminal his-tag appended, the complex having been isolated by size exclusion chromatography. (B) Image of a crystal of the MPP8 chromodomain/3A02 gluebody complex. (C) Representation of the asymmetric unit containing two copies of the 3A02 gluebody/MPP8 chromodomain complex: chain A (blue)/chain C (pale blue) and chain B (green)/chain D (pale green). The sites of the gluebody mutations S7N, Q14K and T125M (S8N, Q14K and T117M in the original non-IMGT sequence) in Nb 3A04 are indicated by side chains in stick representation and the location of the C-terminus is also indicated. (D) Representation of three adjacent asymmetric units in one plane of the crystal lattice, with inset close-ups of crystal contacts between chain B of AU1 and chain B of AU2, and between chain A of AU3 and chain A of AU2, which in both cases involve K14 and M125 (K14, M117 in the original non-IMGT sequence) and the nanobody C-terminus.

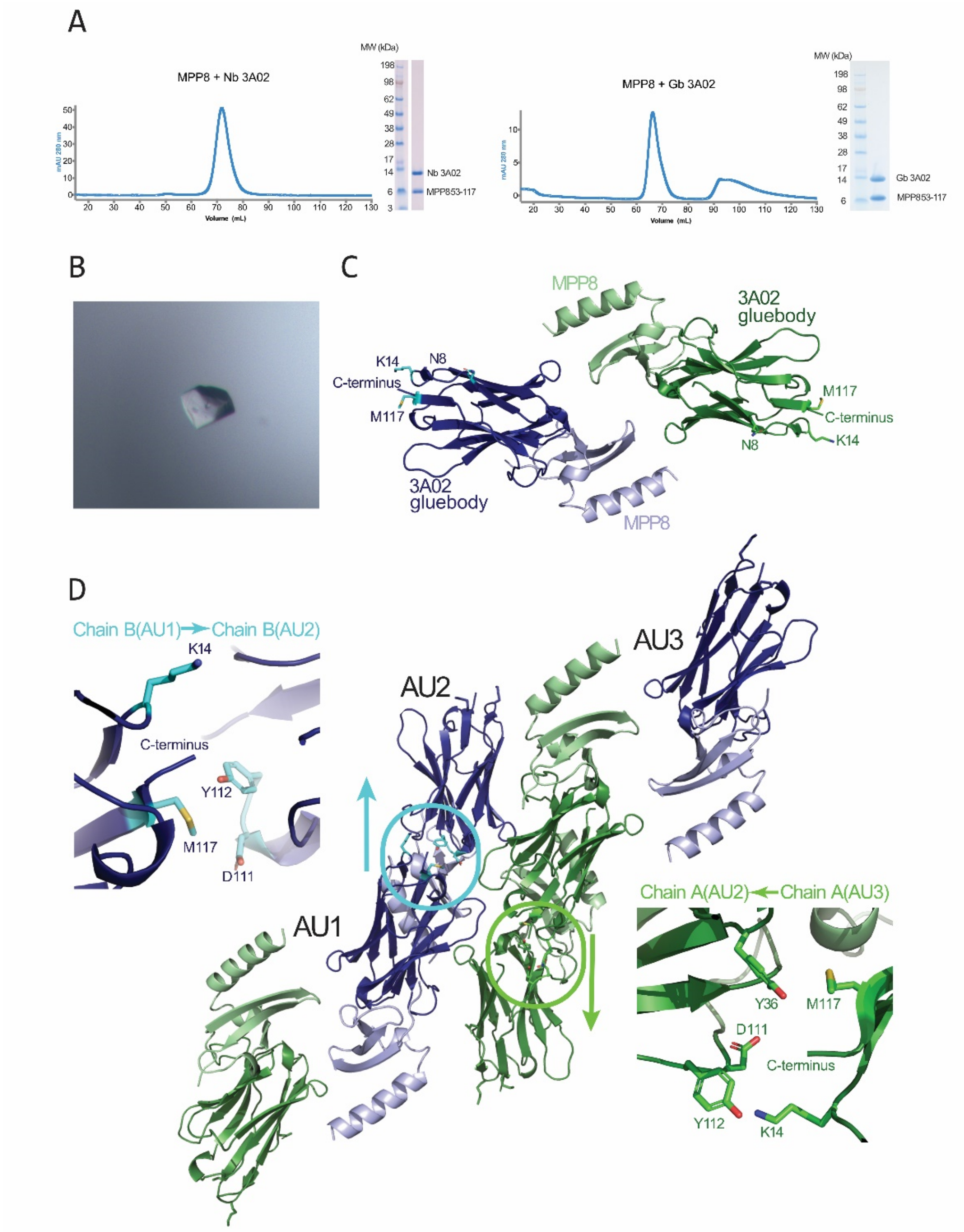

Supplementary tables

<u>Table S1</u> The crystallization experiment on a selection of target:nanobody pairs with naïve nanobody scaffolds.

Table S1 Extended Data table 1 Trials of co-crystallization of targets and nanobodies with naive scaffolds

Target-Nanobody PAIR	STRUCTURE SOLVED?	Target ID	Vector Name	Target protein mass	Construct Protein Sequence	Corresponding Nanobody ID	Nanobody Protein Sequence
CTAG1BA:YNCTAG1BA-c001	no	CTAG1BA-c113	pNIC-CTHF	10582.5	MLEFYLAMPFATPMEAELARRSLAQDAPPLP<br>VPGVLLKEFTVSGNILTIRLTAADHRQLQLSISS<br>CLQQLSLLMWITQCFLPVFLAQAENLYFQ	YNCTAG1BA-c001	QVQLQESGGGLVQAGGSLRLSCAAS<br>GRTFSSYSMGWFRQAPGKEREFVA<br>GISWSGGSTDYVDSVKGRFTISRDN<br>AKNTAYLQMNSLKPEDTAVYYCAA<br>DRGDGGSNWRLETYYDYWGQGTQ<br>VTVS
CTAG1BA:YNCTAG1BA-c002	no	CTAG1BA-c113	pNIC-CTHF	10582.5	MLEFYLAMPFATPMEAELARRSLAQDAPPLP<br>VPGVLLKEFTVSGNILTIRLTAADHRQLQLSISS<br>CLQQLSLLMWITQCFLPVFLAQAENLYFQ	YNCTAG1BA-c002	QVQLQESGGGLVQAGGSLRLSCATS<br>GRTVYTLAMGWFRQAPGKEREFVA<br>AVSTSGGSTFYADSVEDRFTISKDNA<br>KSTVNLHMNSLNPEDTAVYYCAAK<br>MGYTSSRWTNYRLTDVYDYWGQG<br>TQVTVS
CTAG1BA:YNCTAG1BA-c003	no	CTAG1BA-c113	pNIC-CTHF	10582.5	MLEFYLAMPFATPMEAELARRSLAQDAPPLP<br>VPGVLLKEFTVSGNILTIRLTAADHRQLQLSISS<br>CLQQLSLLMWITQCFLPVFLAQAENLYFQ	YNCTAG1BA-c003	QVQLQESGGGLVQPGGSLRLSCAAS<br>GFSSDYYEIGWFRQAPGKEREAVSCI<br>SSGDNTYYKDSVKGRFTISRDNAKKT<br>VYLQMNSLKSEDTAVYFCAAGLRTV<br>AGSQCPMSRYFYSSWGQGTQVTVS
MAGEB1A:YNMAGEB1A-c001	yes PDB id 6R7T	MAGEB1A-c001	pNIC28-Bsa4	29161.3	SMSTESSVKDPVAWEAGMLMHFILRKYKMR<br>EPIMKADMLKVVDEKYKEYFTEILNGASRRLE<br>LVFGLDLKEDNPSGHTYTLVSKLNLTNDGNLS<br>NDWDFPRNGLLMPLLGVIFLKGNSATEEEIW<br>KFMNVLGAYDGEEHLIYGEPRKFITQDLVQE<br>KYLKYEQVPNSDPPRYQFLWGPRAYAETTK<br>MKVLEFLAKMNGATPRDFPSHYEEALRDEEE<br>RAQVRSSVRARRRTTATTFRARSRAPFSRSSH<br>PM	YNMAGEB1A-c001	QVQLQESGGGLVQAGGSLRLSCAAS<br>GFTFDDYAIGWFRQVPGKEREGVSC<br>TSSSVGSTYYADSVKGRFTITSDNAK<br>NTVYLQMNSLKPEDTAVYYCAADSY<br>PYGSTWCRPPFFASWGQGTQVTVS
MAGEC2A:YNMAGEC2A-c001	no	MAGEC2A-c209	pNH-TrxT	24626.3	SMESSFTYTLDEKVAELVEFLLLKYEAEEPVTE<br>AEMLMIVIKYKDYFPVILKRAREFMELLFGLAL<br>IEVGPDHFCVFANTVGLTDEGSDDEGMPENS<br>LLIIILSVIFIKGNCASEEVIWEVLNAVGVYAGR<br>EHFVYGEPRELLTKVWVQGHYLEYREVPHSS<br>PPYYEFLWGPRAHSESIKKKVLEFLAKLNNTV<br>PSSFPSWYKDALKDVEERVQ	YNMAGEC2A-c001	QVQLQESGGGLVQAGGSLRLSCKAS<br>ISLFNRFDVAWYRQAPGKQRDMVA<br>RITSGGSVEYSDDVKGRFTIARDNAK<br>NMVLLQMNSLKLEDTAVYYCNLKR<br>RWSWGSSNEFWGQGTQVTVS
RECQL5A:YYRECQL5A-c004	no	RECQL5A-c104	pNIC28-Bsa4	49466.3	SMDPERRVRSTLKKVFGFDSFKTPLQESATM<br>AVVKGNKDVFVCMPTGAGKSLCYQLPALLAK<br>GITIVVSPLIALIQDQVDHLLTLKVRVSSLNSKL<br>SAQERKELLADLEREKPQTKILYITPEMAASSS<br>FQPTLNSLVSRHLLSYLVVDEAHCVSQWGHD<br>FRPDYLRLGALRSRLGHAPCVALTATATPQV<br>QEDVFAALHLKKPVAIFKTPCFRANLFYDVQF<br>KELISDPYGNLKDFCLKALGQEADKGLSGCGI<br>VYCRTREACEQLAIELSCRGVNAKAYHAGLKA<br>SERTLVQNDWMEEKVPVIVATISFGMGVDK<br>ANVRFVAHWNIAKSMAGYYQESGRAGRDG<br>KPSWCRLYYSRNDRDQVSFLIRKEVAKLQEKR<br>GNKASDKATIMAFDALVTFCEELGCRHAAIAK<br>YFGDALPACAKGCDHCQNPTAVRRRLEALER<br>SSSW	YYRECQL5A-c004	QVQLQESGGGLVQAGGSLRLSCAAS<br>GFTFDDYAIGWFRQAPGKEREGVSS<br>IKSSDGSTYYADSVKGRFTISSDNAK<br>NTVYLQMNSLKPEDTAVYYCAATET<br>RPTMDPLDFGYWDQGTQVTVSAE<br>NLYFQ
RECQL5A:YYRECQL5A-c005	no	RECQL5A-c104	pNIC28-Bsa4	49466.3	SMDPERRVRSTLKKVFGFDSFKTPLQESATM<br>AVVKGNKDVFVCMPTGAGKSLCYQLPALLAK<br>GITIVVSPLIALIQDQVDHLLTLKVRVSSLNSKL<br>SAQERKELLADLEREKPQTKILYITPEMAASSS<br>FQPTLNSLVSRHLLSYLVVDEAHCVSQWGHD<br>FRPDYLRLGALRSRLGHAPCVALTATATPQV<br>QEDVFAALHLKKPVAIFKTPCFRANLFYDVQF<br>KELISDPYGNLKDFCLKALGQEADKGLSGCGI<br>VYCRTREACEQLAIELSCRGVNAKAYHAGLKA<br>SERTLVQNDWMEEKVPVIVATISFGMGVDK<br>ANVRFVAHWNIAKSMAGYYQESGRAGRDG<br>KPSWCRLYYSRNDRDQVSFLIRKEVAKLQEKR<br>GNKASDKATIMAFDALVTFCEELGCRHAAIAK<br>YFGDALPACAKGCDHCQNPTAVRRRLEALER<br>SSSW	YYRECQL5A-c005	QVQLQESGGGLVQAGGSLRLSCAAS<br>GNIFNINAMGWYRQVPGKQREMV<br>AAITSGGSTNYADSVKGRFTISRDNA<br>KNTVYLQMNSLNPEDTAIYYCEAYG<br>TYTLAPTGEGEYDDYWGQGTQVTV<br>SAENLYFQ
RECQL5A:YYRECQL5A-c006	no	RECQL5A-c104	pNIC28-Bsa4	49466.3	SMDPERRVRSTLKKVFGFDSFKTPLQESATM<br>AVVKGNKDVFVCMPTGAGKSLCYQLPALLAK<br>GITIVVSPLIALIQDQVDHLLTLKVRVSSLNSKL<br>SAQERKELLADLEREKPQTKILYITPEMAASSS<br>FQPTLNSLVSRHLLSYLVVDEAHCVSQWGHD<br>FRPDYLRLGALRSRLGHAPCVALTATATPQV<br>QEDVFAALHLKKPVAIFKTPCFRANLFYDVQF<br>KELISDPYGNLKDFCLKALGQEADKGLSGCGI<br>VYCRTREACEQLAIELSCRGVNAKAYHAGLKA<br>SERTLVQNDWMEEKVPVIVATISFGMGVDK<br>ANVRFVAHWNIAKSMAGYYQESGRAGRDG<br>KPSWCRLYYSRNDRDQVSFLIRKEVAKLQEKR<br>GNKASDKATIMAFDALVTFCEELGCRHAAIAK<br>YFGDALPACAKGCDHCQNPTAVRRRLEALER<br>SSSW	YYRECQL5A-c006	MQVQLQESGGGLVQAGGSLRLSCA<br>ASGSIFSINRMGWYRQAPGKQRELV<br>AAITSGGSTNYADSVKGRFTISRDNA<br>KNTVYLQMNSLKPEDTAIYYCEAYG<br>TYTLAPTGEGEYDDYWGQGTQVTV<br>SAAENLYFQ
WRNA:YNWRNA-c001	no	WRNA-c060	pFB-6HZB	47992.8485	SMAPNEEQVTCLKMYFGHSSFKPVQWKVIH<br>SVLEERRDNVAVMATGYGKSLCFQYPPVYVG<br>KIGLVISPLISLMEDQVLQLKMSNIPACFLGSA<br>QSENVLTDIKLGKYRIVYVTPEYCSGNMGLLQ<br>QLEADIGITLIAVDEAHCISEWGHDFRDSFRKL<br>GSLKTALPMVPIVALTATASSSIREDIVRCLNL<br>RNPQITCTGFDRPNLYLEVRRKTGNILQDLQP<br>FLVKTSSHWEFEGPTIIYCPSRKMTQQVTGEL<br>RKLNLSCGTYHAGMSFSTRKDIHHRFVRDEIQ<br>CVIATIAFGMGINKADIRQVIHYGAPKDMESY<br>YQEIGRAGRDGLQSSCHVLWAPADINLNRHL<br>LTEIRNEKFRLYKLKMMAKMEKYLHSSRCRR<br>QIILSHFEDKQVQKASLGIMGTEKCCDNCRSR<br>LDHCYSMD	YNWRNA-c001	QVQLVESGGGLVQAGNSLRLSCLAS<br>GRTLASYGMGWFRQAPGKEREFVA<br>AISRSFSNTYYADSVKGRFTISRDNAK<br>NAVYLQMNILQPEDTAAYYCAATSR<br>FAAATDSQYYDYWGQGTQVTVSSH<br>HHHHHEPEA*
WRNA:YNWRNA-c002	no	WRNA-c060	pFB-6HZB	47992.8485	SMAPNEEQVTCLKMYFGHSSFKPVQWKVIH<br>SVLEERRDNVAVMATGYGKSLCFQYPPVYVG<br>KIGLVISPLISLMEDQVLQLKMSNIPACFLGSA<br>QSENVLTDIKLGKYRIVYVTPEYCSGNMGLLQ<br>QLEADIGITLIAVDEAHCISEWGHDFRDSFRKL<br>GSLKTALPMVPIVALTATASSSIREDIVRCLNL<br>RNPQITCTGFDRPNLYLEVRRKTGNILQDLQP<br>FLVKTSSHWEFEGPTIIYCPSRKMTQQVTGEL<br>RKLNLSCGTYHAGMSFSTRKDIHHRFVRDEIQ<br>CVIATIAFGMGINKADIRQVIHYGAPKDMESY<br>YQEIGRAGRDGLQSSCHVLWAPADINLNRHL<br>LTEIRNEKFRLYKLKMMAKMEKYLHSSRCRR<br>QIILSHFEDKQVQKASLGIMGTEKCCDNCRSR<br>LDHCYSMD	YNWRNA-c002	QVQLVESGGGLVQPGGSLRLSCLAS<br>GRTLASYGMGWFRQAPGKEREFVA<br>AISRSFSNTYYADSVKGRFTISRDNAK<br>NAVYLQMNILQPEDTAAYYCAATSR<br>FAAATDSQYYDYWGQGTQVTVSSH<br>HHHHHEPEA*

Table S1 Extended Data table 1 Trials of co-crystallization of targets and nanobodies with naive scaffolds

WRNA:YNWRNA-c005	no	WRNA-c060	pFB-6HZB	47992.8485	SMAPNEEQVTCLKMYFGHSSFKPVQWKVIH<br>SVLEERRDNVAVMATGYGKSLCFQYPPVYVG<br>KIGLVISPLISLMEDQVLQLKMSNIPACFLGSA<br>QSENVLTDIKLGKYRIVYVTPEYCSGNMGLLQ<br>QLEADIGITLIAVDEAHCISEWGHDFRDSFRKL<br>GSLKTALPMVPIVALTATASSSIREDIVRCLNL<br>RNPQITCTGFDRPNLYLEVRRKTGNILQDLQP<br>FLVKTSSHWEFEGPTIIYCPSRKMTQQVTGEL<br>RKLNLSCGTYHAGMSFSTRKDIHHRFVRDEIQ<br>CVIATIAFGMGINKADIRQVIHYGAPKDMESY<br>YQEIGRAGRDGLQSSCHVLWAPADINLNRHL<br>LTEIRNEKFRLYKLKMMAKMEKYLHSSRCRR<br>QIILSHFEDKQVQKASLGIMGTEKCCDNCRSR<br>LDHCYSMD	YNWRNA-c005	QVQLVESGGGLVQAGNSLSLSCLAS<br>GRTLASYGMGWFRQAPGKEREFVA<br>AISRSFSNTYYADSVKGRFTISRDNAK<br>NAVYLQMNILQPEDTAAYYCAATSR<br>FAAATDSQYYDYWGQGTQVTVSSH<br>HHHHHEPEA*
WRNA:YNWRNA-c006	no	WRNA-c060	pFB-6HZB	47992.8485	SMAPNEEQVTCLKMYFGHSSFKPVQWKVIH<br>SVLEERRDNVAVMATGYGKSLCFQYPPVYVG<br>KIGLVISPLISLMEDQVLQLKMSNIPACFLGSA<br>QSENVLTDIKLGKYRIVYVTPEYCSGNMGLLQ<br>QLEADIGITLIAVDEAHCISEWGHDFRDSFRKL<br>GSLKTALPMVPIVALTATASSSIREDIVRCLNL<br>RNPQITCTGFDRPNLYLEVRRKTGNILQDLQP<br>FLVKTSSHWEFEGPTIIYCPSRKMTQQVTGEL<br>RKLNLSCGTYHAGMSFSTRKDIHHRFVRDEIQ<br>CVIATIAFGMGINKADIRQVIHYGAPKDMESY<br>YQEIGRAGRDGLQSSCHVLWAPADINLNRHL<br>LTEIRNEKFRLYKLKMMAKMEKYLHSSRCRR<br>QIILSHFEDKQVQKASLGIMGTEKCCDNCRSR<br>LDHCYSMD	YNWRNA-c006	QVQLVESGGGLVQTGGSLRLSCAAS<br>ESITSTMSWYRQAPGKQRELVASITY<br>FGSTDYADFVKGRFSISRNGARDML<br>FLQMNKMKPEDTAVYYCKVHTLYG<br>RDYWGQGTQVTVSSHHHHHHEPE<br>A*
WRNA:YNWRNA-c013	no	WRNA-c060	pFB-6HZB	47992.8485	SMAPNEEQVTCLKMYFGHSSFKPVQWKVIH<br>SVLEERRDNVAVMATGYGKSLCFQYPPVYVG<br>KIGLVISPLISLMEDQVLQLKMSNIPACFLGSA<br>QSENVLTDIKLGKYRIVYVTPEYCSGNMGLLQ<br>QLEADIGITLIAVDEAHCISEWGHDFRDSFRKL<br>GSLKTALPMVPIVALTATASSSIREDIVRCLNL<br>RNPQITCTGFDRPNLYLEVRRKTGNILQDLQP<br>FLVKTSSHWEFEGPTIIYCPSRKMTQQVTGEL<br>RKLNLSCGTYHAGMSFSTRKDIHHRFVRDEIQ<br>CVIATIAFGMGINKADIRQVIHYGAPKDMESY<br>YQEIGRAGRDGLQSSCHVLWAPADINLNRHL<br>LTEIRNEKFRLYKLKMMAKMEKYLHSSRCRR<br>QIILSHFEDKQVQKASLGIMGTEKCCDNCRSR<br>LDHCYSMD	YNWRNA-c013	QVQLVESGGGLVQPGGSLRLSCAAS<br>GFTFGTYFMNWFRQAPGKGPEWV<br>SGIDPGGDRTWYVESVKGRFTISRD<br>NVKNTLYLQMNSLKPADTAEYYCSP<br>QGRPERGQGTQVTVSSHHHHHHE<br>PEA*
WRNA:YNWRNA-c014	no	WRNA-c060	pFB-6HZB	47992.8485	SMAPNEEQVTCLKMYFGHSSFKPVQWKVIH<br>SVLEERRDNVAVMATGYGKSLCFQYPPVYVG<br>KIGLVISPLISLMEDQVLQLKMSNIPACFLGSA<br>QSENVLTDIKLGKYRIVYVTPEYCSGNMGLLQ<br>QLEADIGITLIAVDEAHCISEWGHDFRDSFRKL<br>GSLKTALPMVPIVALTATASSSIREDIVRCLNL<br>RNPQITCTGFDRPNLYLEVRRKTGNILQDLQP<br>FLVKTSSHWEFEGPTIIYCPSRKMTQQVTGEL<br>RKLNLSCGTYHAGMSFSTRKDIHHRFVRDEIQ<br>CVIATIAFGMGINKADIRQVIHYGAPKDMESY<br>YQEIGRAGRDGLQSSCHVLWAPADINLNRHL<br>LTEIRNEKFRLYKLKMMAKMEKYLHSSRCRR<br>QIILSHFEDKQVQKASLGIMGTEKCCDNCRSR<br>LDHCYSMD	YNWRNA-c014	QVQLVESGGGLVQPGGSLRLSCAAS<br>GFTFGTYFMNWFRQAPGKGPEWV<br>SGINPGGDRTSYVESVKGRFTISRDN<br>VKNTLYLQMNSLKPADTAEYYCSPQ<br>GRPERGQGTQVTVSSHHHHHHEPE<br>A*
WRNA:YNWRNA-c015	no	WRNA-c060	pFB-6HZB	47992.8485	SMAPNEEQVTCLKMYFGHSSFKPVQWKVIH<br>SVLEERRDNVAVMATGYGKSLCFQYPPVYVG<br>KIGLVISPLISLMEDQVLQLKMSNIPACFLGSA<br>QSENVLTDIKLGKYRIVYVTPEYCSGNMGLLQ<br>QLEADIGITLIAVDEAHCISEWGHDFRDSFRKL<br>GSLKTALPMVPIVALTATASSSIREDIVRCLNL<br>RNPQITCTGFDRPNLYLEVRRKTGNILQDLQP<br>FLVKTSSHWEFEGPTIIYCPSRKMTQQVTGEL<br>RKLNLSCGTYHAGMSFSTRKDIHHRFVRDEIQ<br>CVIATIAFGMGINKADIRQVIHYGAPKDMESY<br>YQEIGRAGRDGLQSSCHVLWAPADINLNRHL<br>LTEIRNEKFRLYKLKMMAKMEKYLHSSRCRR<br>QIILSHFEDKQVQKASLGIMGTEKCCDNCRSR<br>LDHCYSMD	YNWRNA-c015	QVQLVESGGGLVQAGGSLRLSCAAS<br>GSIFSSNIMGWYRQGPGNTRRFVAS<br>IAPGGTTNYERSVKGRFTISRDNAKN<br>TVHLQMNSLKPDDTADYLCNALPA<br>DYWGQGTQVTVSSHHHHHHEPEA<br>*
WRNA:YNWRNA-c017	no	WRNA-c060	pFB-6HZB	47992.8485	SMAPNEEQVTCLKMYFGHSSFKPVQWKVIH<br>SVLEERRDNVAVMATGYGKSLCFQYPPVYVG<br>KIGLVISPLISLMEDQVLQLKMSNIPACFLGSA<br>QSENVLTDIKLGKYRIVYVTPEYCSGNMGLLQ<br>QLEADIGITLIAVDEAHCISEWGHDFRDSFRKL<br>GSLKTALPMVPIVALTATASSSIREDIVRCLNL<br>RNPQITCTGFDRPNLYLEVRRKTGNILQDLQP<br>FLVKTSSHWEFEGPTIIYCPSRKMTQQVTGEL<br>RKLNLSCGTYHAGMSFSTRKDIHHRFVRDEIQ<br>CVIATIAFGMGINKADIRQVIHYGAPKDMESY<br>YQEIGRAGRDGLQSSCHVLWAPADINLNRHL<br>LTEIRNEKFRLYKLKMMAKMEKYLHSSRCRR<br>QIILSHFEDKQVQKASLGIMGTEKCCDNCRSR<br>LDHCYSMD	YNWRNA-c017	QVQLVESGGGLVQAGGSLRLSCAAS<br>GRTSSIYGMGWFRQAPGKEREFVA<br>AITWILGTAYYGDSVKGRFTISRDNA<br>KNTYLQMNSLKPEDTAVYYCAGQE<br>APFAPRDPGRVNYWGQGTQVTVSS<br>HHHHHHEPEA*
WRNA:YNWRNA-c018	no	WRNA-c060	pFB-6HZB	47992.8485	SMAPNEEQVTCLKMYFGHSSFKPVQWKVIH<br>SVLEERRDNVAVMATGYGKSLCFQYPPVYVG<br>KIGLVISPLISLMEDQVLQLKMSNIPACFLGSA<br>QSENVLTDIKLGKYRIVYVTPEYCSGNMGLLQ<br>QLEADIGITLIAVDEAHCISEWGHDFRDSFRKL<br>GSLKTALPMVPIVALTATASSSIREDIVRCLNL<br>RNPQITCTGFDRPNLYLEVRRKTGNILQDLQP<br>FLVKTSSHWEFEGPTIIYCPSRKMTQQVTGEL<br>RKLNLSCGTYHAGMSFSTRKDIHHRFVRDEIQ<br>CVIATIAFGMGINKADIRQVIHYGAPKDMESY<br>YQEIGRAGRDGLQSSCHVLWAPADINLNRHL<br>LTEIRNEKFRLYKLKMMAKMEKYLHSSRCRR<br>QIILSHFEDKQVQKASLGIMGTEKCCDNCRSR<br>LDHCYSMD	YNWRNA-c018	QVQLVESGGGLVQPGGSLRLSCAAS<br>RSTVSLNVIAWYRQAQGKQREWVA<br>NIGSGGSTNYAVSVKGRFTISRDNAK<br>NTAYLQMNNLKPEDTAVYYCQAFA<br>HTTAESPYTDYWGQGTQVTVSSHH<br>HHHHEPEA*

Table S2 Detailed DBSCAN result with full list of PDB codes of each nanobody-nanobody interface category. In the 'Pattern name' column, first four letters represent PDB and the digit after '_'
represents the No. of the pattern in the structure. In the ‘Pattern’ column, each number represents the IMGT normalized index of a residue of the nanobody scaffold involved in the pattern.

Table S2 Cluster Result Coordinate Log file

Pattern name	Pattern	Reference pattern	Class
2X1O_2	16 63 65 67 72 74 75 77 92 93	none	-1
2X1O_4	16 63 65 67 72 74 75 77 92 93	none	-1
2X1P_2	16 18 20 77 81 90 92	none	-1
2X1P_3	14 16 18 20 77 81 84 90 92	none	-1
2X1P_5	14 16 18 20 77 81 84 90 92	none	-1
2X1P_6	16 18 20 77 81 90 92	none	-1
2XXC_1	126 38 39 40 45 53 54 55 56 63 69 71 72 74 75 78 120 122	none	-1
3DWT_2	24 25 26 27 5 7	none	-1
3K1K_1	11 24 26 3 5 6 7 8	none	-1
4EJ1_1	48 49 69 70 95 96 97	none	-1
4I0C_1	16 17 18 20 91 93 94	none	-1
4I0C_2	16 17 18 20 91 93 94	none	-1
4JVP_1	119 120 121 122 123 124 125 52	none	-1
4JVP_2	119 120 121 122 123 124 125 52	none	-1
4KML_1	35 59 62 63 82 83	none	-1
4N9O_1	36 62 63 64 84 85	none	-1
4QGY_3	113 114 119 44 48 49 50	none	-1
4QGY_4	27 28 29 30 84 85 86	none	-1
4QGY_5	27 28 29 30 84 85 86	none	-1
4Z9K_1	24 25 26 27 5 7	none	-1
4ZG1_1	1 117 118 119 2 29 36 114	none	-1
4ZG1_2	113 115 53 54 55 56 69	none	-1
4ZG1_5	50 53 54 55 56 69 113 114	none	-1
4ZG1_7	1 118 119 2 29 36 114	none	-1
5BOZ_1	128 128 1 121 24 25 27 28 3 86 87	none	-1
5BOZ_2	24 25 26 28 3 85	none	-1
5BOZ_3	24 25 27 28 3 85 86	none	-1
5C1M_1	124 11 126 128 12 46 97	none	-1
5C1M_2	119 121 122 1 44 2 3 4 102 104	none	-1
5DXW_1	115 117 119 37 39 43 50 51 56 58 59 67 74	none	-1
5E7B_1	35 37 39 41 2 43 44 6 94 95 96 98	none	-1
5FWO_1	1 117 118 119 120 121 122 123 29	none	-1
5G5R_2	1 118 119 120 28 3 50	none	-1
5H8D_1	16 17 18 74 75 90 92 93	none	-1
5H8O_1	15 69 70 96 97 98	none	-1
5HDO_2	1 113 114 115 116 2 27 28 29 36 37	none	-1
5HDO_3	123 125 44 99 100 101	none	-1
5HDO_5	1 113 114 115 116 2 27 28 29 36 37	none	-1
5HDO_6	123 125 44 99 100 101	none	-1
5HDO_7	1 113 114 115 116 2 27 28 29 36 37	none	-1
5HDO_8	20 62 63 64 65 77 79	none	-1
5HDO_9	1 113 114 115 116 2 27 28 29 36 37	none	-1
5HDO_10	123 125 44 46 99 100 101	none	-1
5HDO_11	20 62 63 64 65 77 79	none	-1
5HDO_12	123 125 44 46 99 100 101	none	-1
5HGG_1	62 63 64 65 66 67	none	-1
5HGG_2	62 63 64 65 66 67	none	-1
5IMO_1	17 18 20 56 59 62 63 64 65 74 75 76 77 78 79 80 81 82 83 84 88 90 92	none	-1
5IP4_1	120 24 25 26 3 4 5 6	none	-1
5IP4_2	120 24 25 26 3 4 5 6	none	-1
5IVN_1	119 120 122 127 40 42 66 68 117	none	-1
5JA9_1	27 28 29 30 3 5 87	none	-1
5JA9_4	27 28 29 30 3 5 87	none	-1
5LMJ_1	112B 112A 56 57 65 66	none	-1
5LMJ_2	111A 112A 56 57 65 66	none	-1
5LMJ_3	1 122 123 3 45 5	none	-1
5LMJ_4	1 122 123 3 45 5	none	-1
5LZ0_1	111B 111C 112B 112A 114 111	none	-1
5LZ0_2	111B 111C 112B 112A 114 111	none	-1

Table S2 Cluster Result Coordinate Log file

Pattern name	Pattern	Reference pattern	Class
5M2M_1	120 122 24 26 4 5 6 7 8 9	none	-1
5M2M_2	120 122 24 26 4 5 6 7 8 9	none	-1
5NLU_1	114 115 117 118 120 121 35 36 2 40 55 110 112 113	none	-1
5NLW_1	113 115 116 117 118 36 2 40 63 64 110 112	none	-1
5NM0_2	110 114 115 116 37 38 39 50 52 53 54 55 56 58 59 63 65 67 68 69 108 109	none	-1
5NM0_4	110 114 115 116 37 38 39 50 52 53 54 55 56 58 59 63 65 67 68 69 108 109	none	-1
5NML_1	118 120 24 25 26 27 3 4 5 6 85	none	-1
5NML_14	118 120 24 25 26 27 3 4 5 6 85	none	-1
5NQW_1	14 16 18 20 90 92 93	none	-1
5TP3_1	1 25 27 28 29 82 83 84 85	none	-1
5TP3_2	1 25 27 28 29 82 83 84 85	none	-1
5U65_1	26 29 36 37 85 111A	none	-1
5UK4_3	69 70 72 93 95 97	none	-1
5UK4_21	69 70 72 93 95 97	none	-1
5USF_1	1 115 116 117 118 119 120 44	none	-1
5USF_2	1 115 116 117 118 119 120 44	none	-1
5VL2_2	111 111A 111B 112B 112A 52 65 66 67 68 69	none	-1
5VL2_3	111 111A 111B 112B 112A 65 66 67 68 69	none	-1
5VL2_8	111 111A 111B 112B 112A 65 66 67 68 69	none	-1
5VL2_10	111 111A 111B 112B 112A 52 65 66 67 68 69	none	-1
5VM0_1	64 65 66 83 84 85	none	-1
5VM0_2	64 65 66 83 84 85	none	-1
5VM4_4	62 63 64 65 67 77	none	-1
5VM4_6	59 62 63 80 81 82	none	-1
5WB2_3	128 3 118 128 128 128 128	none	-1
6DBD_8	24 25 26 27 5 7 82 83 85 86	none	-1
6DBE_4	116 118 53 67 68 69	none	-1
6EY0_1	123 12 125 127 13 14	none	-1
6F0D_2	128 108 110 113 114 115 116 1	none	-1
6F0D_4	128 108 111 113 114 115 116 1 28 36	none	-1
6QD6_13	58 64 65 66 111A 111B 111C	none	-1
6QD6_14	58 64 65 66 111A 111B 111C	none	-1
6RU3_1	9 123 124 125 126 127 128 1 24 25 26 27 28 29 2 3 4 5 6 7 8 104 106	none	-1
6U51_2	18 20 65 76 77 79 80 90 92 93	none	-1
6U53_2	66 67 68 69 70 72	none	-1
6WAR_2	120 26 3 5 6 7 8	none	-1
6WAR_4	120 26 3 5 6 7 8	none	-1
4DK3_2	112 113 49 50 51 52	none	-1
4KDT_3	1 119 120 121 2 48 104	none	-1
4KDT_4	1 119 120 121 2 48 104	none	-1
6GJU_1	1 115 117 119 120 3 4	none	-1
6HDA_1	118 119 120 44 48 49 3	none	-1
6HHD_1	114 45 48 49 50 51 105	none	-1
6HHD_2	114 45 48 49 50 51 105	none	-1
6I2G_1	66 67 76 77 80 82 83	none	-1
6ITP_2	1 108 28 30 35 36 60	none	-1
6ITP_4	1 108 28 30 35 36 60	none	-1
6F2G_1	1 118 119 120 24 25 26 3 4 5	none	-1
6F2W_1	1 118 119 120 24 25 26 3 4 5	none	-1
6GCI_1	113 114 118 120 44 47 48 49	none	-1
2X1O_1	108 111A 112A 112 114 115 116 117 37 42 49 50 51 52 66 68 69	6QTL_5:6QTL E:6QTL F:110 112 113 114 115 118 37 42 49 51 52 55 64 66 68 70:0	0
2X1O_3	108 111A 112A 112 114 115 116 117 37 42 49 50 51 52 66 68 69	6QTL_5:6QTL E:6QTL F:110 112 113 114 115 118 37 42 49 51 52 55 64 66 68 70:0	0
5DA4_1	112A 112 113 114 115 116 118 49 50 52 59 64 65 66 67	6QTL_5:6QTL E:6QTL F:110 112 113 114 115 118 37 42 49 51 52 55 64 66 68 70:0	0
5DA4_5	112A 112 113 114 115 116 118 49 50 52 59 64 65 66 67	6QTL_5:6QTL E:6QTL F:110 112 113 114 115 118 37 42 49 51 52 55 64 66 68 70:0	0

Table S2 Cluster Result Coordinate Log file

Pattern name	Pattern	Reference pattern	Class
5DA4_6	111 112A 112 113 114 115 116 118 42 49 50 52 59 64 65 66 67	6QTL_5:6QTL E:6QTL F:110 112 113 114 115 118 37 42 49 51 52 55 64 66 68 70:0	0
6QTL_1	112 113 114 115 118 37 42 43 49 51 52 55 57 64 66 68 70	6QTL_5:6QTL E:6QTL F:110 112 113 114 115 118 37 42 49 51 52 55 64 66 68 70:0	0
6QTL_2	112 113 114 115 118 37 42 43 49 51 52 55 57 64 66 68 70	6QTL_5:6QTL E:6QTL F:110 112 113 114 115 118 37 42 49 51 52 55 64 66 68 70:0	0
6QTL_3	112 113 114 115 118 37 42 43 49 51 52 55 57 64 66 68 70	6QTL_5:6QTL E:6QTL F:110 112 113 114 115 118 37 42 49 51 52 55 64 66 68 70:0	0
6QTL_4	110 112 113 114 115 118 37 42 43 49 50 51 52 55 64 66 68 70	6QTL_5:6QTL E:6QTL F:110 112 113 114 115 118 37 42 49 51 52 55 64 66 68 70:0	0
6QTL_5	110 112 113 114 115 118 37 42 49 51 52 55 64 66 68 70	6QTL_5:6QTL E:6QTL F:110 112 113 114 115 118 37 42 49 51 52 55 64 66 68 70:0	0
6QTL_6	112 113 114 115 118 37 42 43 49 51 52 55 57 64 66 68 70	6QTL_5:6QTL E:6QTL F:110 112 113 114 115 118 37 42 49 51 52 55 64 66 68 70:0	0
6QTL_7	112 113 114 115 118 37 42 43 49 50 51 52 55 64 66 68 70	6QTL_5:6QTL E:6QTL F:110 112 113 114 115 118 37 42 49 51 52 55 64 66 68 70:0	0
6QTL_8	112 113 114 115 118 37 42 49 51 52 55 66 68 70	6QTL_5:6QTL E:6QTL F:110 112 113 114 115 118 37 42 49 51 52 55 64 66 68 70:0	0
6U50_1	115 117 119 38 49 50 52 57 58 66 67 103 108	6QTL_5:6QTL E:6QTL F:110 112 113 114 115 118 37 42 49 51 52 55 64 66 68 70:0	0
6DYX_1	113 114 115 116 117 38 39 40 54 59 66 68 70 71 72 109	6QTL_5:6QTL E:6QTL F:110 112 113 114 115 118 37 42 49 51 52 55 64 66 68 70:0	0
2X1P_1	11 12 120 121 122 127 5 6 7 8 9	6H7J_1:SYM00010000 D:6H7J C:11 12 127 14 19 5 6 7 8 9:1	1
2X1P_4	11 12 120 121 122 127 5 6 7 8 9	6H7J_1:SYM00010000 D:6H7J C:11 12 127 14 19 5 6 7 8 9:1	1
2X1P_7	11 12 120 121 122 127 5 6 7 8 9	6H7J_1:SYM00010000 D:6H7J C:11 12 127 14 19 5 6 7 8 9:1	1
2X1P_8	11 12 120 121 122 127 5 6 7 8 9	6H7J_1:SYM00010000 D:6H7J C:11 12 127 14 19 5 6 7 8 9:1	1
2XV6_1	11 12 13 18 19 93	6H7J_1:SYM00010000 D:6H7J C:11 12 127 14 19 5 6 7 8 9:1	1
2XV6_2	11 12 13 18 19 93	6H7J_1:SYM00010000 D:6H7J C:11 12 127 14 19 5 6 7 8 9:1	1
3P0G_1	11 12 127 14 19 26 3 5 6 7 8 9	6H7J_1:SYM00010000 D:6H7J C:11 12 127 14 19 5 6 7 8 9:1	1
3STB_1	8 9 11 122 12 13 14 18 19 20 7	6H7J_1:SYM00010000 D:6H7J C:11 12 127 14 19 5 6 7 8 9:1	1
4QGY_1	11 12 13 123 14 18 19 20 7 8 9	6H7J_1:SYM00010000 D:6H7J C:11 12 127 14 19 5 6 7 8 9:1	1
4QGY_2	11 12 13 123 14 18 19 20 7 8 9	6H7J_1:SYM00010000 D:6H7J C:11 12 127 14 19 5 6 7 8 9:1	1
4W6W_1	5 6 7 8 9 11 12 13 14 19 120 122 127	6H7J_1:SYM00010000 D:6H7J C:11 12 127 14 19 5 6 7 8 9:1	1
4WEU_1	7 8 9 11 12 13 14 18 19 20 90 92 122	6H7J_1:SYM00010000 D:6H7J C:11 12 127 14 19 5 6 7 8 9:1	1
4WEU_2	7 8 9 11 12 13 14 18 19 20 90 92 122	6H7J_1:SYM00010000 D:6H7J C:11 12 127 14 19 5 6 7 8 9:1	1
4WGV_1	11 12 13 127 14 19 26 3 5 6 7 8 9	6H7J_1:SYM00010000 D:6H7J C:11 12 127 14 19 5 6 7 8 9:1	1
4WGV_2	11 12 13 127 14 19 26 3 5 6 7 8 9	6H7J_1:SYM00010000 D:6H7J C:11 12 127 14 19 5 6 7 8 9:1	1
4WGW_1	11 12 13 127 14 19 26 3 5 6 7 8 9	6H7J_1:SYM00010000 D:6H7J C:11 12 127 14 19 5 6 7 8 9:1	1
4WGW_2	11 12 13 127 14 19 26 3 5 6 7 8 9	6H7J_1:SYM00010000 D:6H7J C:11 12 127 14 19 5 6 7 8 9:1	1
4ZG1_3	11 12 13 14 5 6 7 8 9	6H7J_1:SYM00010000 D:6H7J C:11 12 127 14 19 5 6 7 8 9:1	1
4ZG1_4	11 12 14 15 20 28 5 6 7 8 9	6H7J_1:SYM00010000 D:6H7J C:11 12 127 14 19 5 6 7 8 9:1	1
4ZG1_6	12 13 14 6 7 8 9 11	6H7J_1:SYM00010000 D:6H7J C:11 12 127 14 19 5 6 7 8 9:1	1
4ZG1_8	11 12 13 14 6 7 8 9	6H7J_1:SYM00010000 D:6H7J C:11 12 127 14 19 5 6 7 8 9:1	1

Table S2 Cluster Result Coordinate Log file

Pattern name	Pattern	Reference pattern	Class
4ZG1_9	11 12 13 14 5 6 7 8 9	6H7J_1:SYM00010000 D:6H7J C:11 12 127 14 19 5 6 7 8 9:1	1
4ZG1_10	11 12 14 19 27 4 5 6 7 8 9	6H7J_1:SYM00010000 D:6H7J C:11 12 127 14 19 5 6 7 8 9:1	1
5DA4_2	7 8 9 127 11 12 13 14 19 26 5 6	6H7J_1:SYM00010000 D:6H7J C:11 12 127 14 19 5 6 7 8 9:1	1
5DA4_3	7 8 9 127 11 12 13 14 19 26 5 6	6H7J_1:SYM00010000 D:6H7J C:11 12 127 14 19 5 6 7 8 9:1	1
5DA4_4	7 8 9 127 11 12 13 14 19 26 5 6	6H7J_1:SYM00010000 D:6H7J C:11 12 127 14 19 5 6 7 8 9:1	1
5HDO_1	11 12 18 19 20 90 92	6H7J_1:SYM00010000 D:6H7J C:11 12 127 14 19 5 6 7 8 9:1	1
5HDO_4	11 12 18 19 20 90 92	6H7J_1:SYM00010000 D:6H7J C:11 12 127 14 19 5 6 7 8 9:1	1
5JA9_2	9 11 128 12 13 14 19 20 8	6H7J_1:SYM00010000 D:6H7J C:11 12 127 14 19 5 6 7 8 9:1	1
5JA9_3	11 12 128 13 14 19 20 8 9	6H7J_1:SYM00010000 D:6H7J C:11 12 127 14 19 5 6 7 8 9:1	1
5M2W_1	11 12 13 14 19 20	6H7J_1:SYM00010000 D:6H7J C:11 12 127 14 19 5 6 7 8 9:1	1
5M2W_2	12 13 14 19 20 11	6H7J_1:SYM00010000 D:6H7J C:11 12 127 14 19 5 6 7 8 9:1	1
5M7Q_1	11 12 13 14 18 19 20 7 8 9	6H7J_1:SYM00010000 D:6H7J C:11 12 127 14 19 5 6 7 8 9:1	1
5M7Q_2	13 14 18 19 20 7 8 9	6H7J_1:SYM00010000 D:6H7J C:11 12 127 14 19 5 6 7 8 9:1	1
5M94_1	12 13 127 14 19 26 3 5 6 7 8 9	6H7J_1:SYM00010000 D:6H7J C:11 12 127 14 19 5 6 7 8 9:1	1
5M94_2	12 13 127 14 19 26 3 5 6 7 8 9	6H7J_1:SYM00010000 D:6H7J C:11 12 127 14 19 5 6 7 8 9:1	1
5M95_1	11 12 13 127 14 19 26 3 5 6 7 8 9	6H7J_1:SYM00010000 D:6H7J C:11 12 127 14 19 5 6 7 8 9:1	1
5M95_2	11 12 13 127 14 19 26 3 5 6 7 8 9	6H7J_1:SYM00010000 D:6H7J C:11 12 127 14 19 5 6 7 8 9:1	1
5O0W_1	11 12 13 14 18 19 20 92 9	6H7J_1:SYM00010000 D:6H7J C:11 12 127 14 19 5 6 7 8 9:1	1
5O0W_2	11 12 13 14 18 19 20 92 9	6H7J_1:SYM00010000 D:6H7J C:11 12 127 14 19 5 6 7 8 9:1	1
5U64_1	14 16 17 22 23 24 26 11 12 108 13	6H7J_1:SYM00010000 D:6H7J C:11 12 127 14 19 5 6 7 8 9:1	1
5UK4_1	11 12 13 14 18 19 20 9	6H7J_1:SYM00010000 D:6H7J C:11 12 127 14 19 5 6 7 8 9:1	1
5UK4_2	11 12 13 14 18 19 20 9	6H7J_1:SYM00010000 D:6H7J C:11 12 127 14 19 5 6 7 8 9:1	1
5UK4_4	11 12 13 14 18 19 20 9	6H7J_1:SYM00010000 D:6H7J C:11 12 127 14 19 5 6 7 8 9:1	1
5UK4_5	11 12 13 14 18 19 20 9	6H7J_1:SYM00010000 D:6H7J C:11 12 127 14 19 5 6 7 8 9:1	1
5UK4_6	11 12 13 14 18 19 20 9	6H7J_1:SYM00010000 D:6H7J C:11 12 127 14 19 5 6 7 8 9:1	1
5UK4_7	11 12 13 14 18 19 20 9	6H7J_1:SYM00010000 D:6H7J C:11 12 127 14 19 5 6 7 8 9:1	1
5UK4_8	11 12 13 14 18 19 20 9	6H7J_1:SYM00010000 D:6H7J C:11 12 127 14 19 5 6 7 8 9:1	1
5UK4_9	11 12 13 14 18 19 20 9	6H7J_1:SYM00010000 D:6H7J C:11 12 127 14 19 5 6 7 8 9:1	1
5UK4_10	11 12 13 14 18 19 20 9	6H7J_1:SYM00010000 D:6H7J C:11 12 127 14 19 5 6 7 8 9:1	1
5UK4_11	11 12 13 14 18 19 20 9	6H7J_1:SYM00010000 D:6H7J C:11 12 127 14 19 5 6 7 8 9:1	1
5UK4_12	11 12 13 14 18 19 20 9	6H7J_1:SYM00010000 D:6H7J C:11 12 127 14 19 5 6 7 8 9:1	1
5UK4_13	11 12 13 14 18 19 20 9	6H7J_1:SYM00010000 D:6H7J C:11 12 127 14 19 5 6 7 8 9:1	1

Table S2 Cluster Result Coordinate Log file

Pattern name	Pattern	Reference pattern	Class
5UK4_14	11 12 13 14 18 19 20 9	6H7J_1:SYM00010000 D:6H7J C:11 12 127 14 19 5 6 7 8 9:1	1
5UK4_15	11 12 13 14 18 19 20 9	6H7J_1:SYM00010000 D:6H7J C:11 12 127 14 19 5 6 7 8 9:1	1
5UK4_16	11 12 13 14 18 19 20 9	6H7J_1:SYM00010000 D:6H7J C:11 12 127 14 19 5 6 7 8 9:1	1
5UK4_17	11 12 13 14 18 19 20 9	6H7J_1:SYM00010000 D:6H7J C:11 12 127 14 19 5 6 7 8 9:1	1
5UK4_18	11 12 13 14 18 19 20 9	6H7J_1:SYM00010000 D:6H7J C:11 12 127 14 19 5 6 7 8 9:1	1
5UK4_19	11 12 13 14 18 19 20 9	6H7J_1:SYM00010000 D:6H7J C:11 12 127 14 19 5 6 7 8 9:1	1
5UK4_20	11 12 13 14 18 19 20 9	6H7J_1:SYM00010000 D:6H7J C:11 12 127 14 19 5 6 7 8 9:1	1
5UK4_22	11 12 13 14 18 19 20 9	6H7J_1:SYM00010000 D:6H7J C:11 12 127 14 19 5 6 7 8 9:1	1
6DBA_1	11 13 19 20 7 8 93 9	6H7J_1:SYM00010000 D:6H7J C:11 12 127 14 19 5 6 7 8 9:1	1
6DBA_2	11 13 19 20 7 8 93 9	6H7J_1:SYM00010000 D:6H7J C:11 12 127 14 19 5 6 7 8 9:1	1
6DBD_3	11 12 13 14 3 5 6 7 8 9	6H7J_1:SYM00010000 D:6H7J C:11 12 127 14 19 5 6 7 8 9:1	1
6DBD_9	11 12 13 14 3 5 6 7 8 9	6H7J_1:SYM00010000 D:6H7J C:11 12 127 14 19 5 6 7 8 9:1	1
6F0D_1	9 11 13 14 18 19 20 90 92 8	6H7J_1:SYM00010000 D:6H7J C:11 12 127 14 19 5 6 7 8 9:1	1
6F0D_3	9 13 14 18 19 20 90 92 8	6H7J_1:SYM00010000 D:6H7J C:11 12 127 14 19 5 6 7 8 9:1	1
6FUZ_1	11 12 13 18 19 20 90 92 9	6H7J_1:SYM00010000 D:6H7J C:11 12 127 14 19 5 6 7 8 9:1	1
6FV0_1	11 12 13 14 18 19 20 90 92 9	6H7J_1:SYM00010000 D:6H7J C:11 12 127 14 19 5 6 7 8 9:1	1
6H7J_1	11 12 127 14 19 5 6 7 8 9	6H7J_1:SYM00010000 D:6H7J C:11 12 127 14 19 5 6 7 8 9:1	1
6H7J_2	11 12 127 14 19 5 6 7 8 9	6H7J_1:SYM00010000 D:6H7J C:11 12 127 14 19 5 6 7 8 9:1	1
6H7L_1	11 12 127 13 14 19 5 6 7 8 9	6H7J_1:SYM00010000 D:6H7J C:11 12 127 14 19 5 6 7 8 9:1	1
6H7L_2	11 12 127 13 14 19 5 6 7 8 9	6H7J_1:SYM00010000 D:6H7J C:11 12 127 14 19 5 6 7 8 9:1	1
6H7M_1	11 12 127 13 14 19 5 6 7 8 9	6H7J_1:SYM00010000 D:6H7J C:11 12 127 14 19 5 6 7 8 9:1	1
6H7M_2	11 12 127 13 14 19 5 6 7 8 9	6H7J_1:SYM00010000 D:6H7J C:11 12 127 14 19 5 6 7 8 9:1	1
6H7N_1	11 12 127 14 19 5 6 7 8 9	6H7J_1:SYM00010000 D:6H7J C:11 12 127 14 19 5 6 7 8 9:1	1
6H7N_2	11 12 127 14 19 5 6 7 8 9	6H7J_1:SYM00010000 D:6H7J C:11 12 127 14 19 5 6 7 8 9:1	1
6H7O_1	11 12 127 13 14 19 26 5 6 7 8 9	6H7J_1:SYM00010000 D:6H7J C:11 12 127 14 19 5 6 7 8 9:1	1
6H7O_2	11 12 127 13 14 19 26 5 6 7 8 9	6H7J_1:SYM00010000 D:6H7J C:11 12 127 14 19 5 6 7 8 9:1	1
6IBL_1	11 12 127 14 19 5 6 7 8 9	6H7J_1:SYM00010000 D:6H7J C:11 12 127 14 19 5 6 7 8 9:1	1
6IBL_2	11 12 127 14 19 5 6 7 8 9	6H7J_1:SYM00010000 D:6H7J C:11 12 127 14 19 5 6 7 8 9:1	1
6OS1_1	11 12 13 14 18 19 20 8 9	6H7J_1:SYM00010000 D:6H7J C:11 12 127 14 19 5 6 7 8 9:1	1
6OS2_1	11 12 13 14 18 19 20 8 9	6H7J_1:SYM00010000 D:6H7J C:11 12 127 14 19 5 6 7 8 9:1	1
6Q6Z_1	1 11 12 14 26 3 5 6 7 8 9	6H7J_1:SYM00010000 D:6H7J C:11 12 127 14 19 5 6 7 8 9:1	1
6QGY_1	9 11 122 12 13 14 17 18 19 20 7 90 8	6H7J_1:SYM00010000 D:6H7J C:11 12 127 14 19 5 6 7 8 9:1	1

Table S2 Cluster Result Coordinate Log file

Pattern name	Pattern	Reference pattern	Class
6QGY_3	9 11 122 12 13 14 17 18 19 20 7 90 8	6H7J_1:SYM00010000 D:6H7J C:11 12 127 14 19 5 6 7 8 9:1	1
6U52_1	11 12 13 5 6 7 8 9	6H7J_1:SYM00010000 D:6H7J C:11 12 127 14 19 5 6 7 8 9:1	1
6U52_2	11 12 13 5 6 7 8 9	6H7J_1:SYM00010000 D:6H7J C:11 12 127 14 19 5 6 7 8 9:1	1
6U53_1	11 12 13 14 18 19 20 7 8 98 100 9 107	6H7J_1:SYM00010000 D:6H7J C:11 12 127 14 19 5 6 7 8 9:1	1
6U54_1	11 12 13 14 20 2 5 6 7 8 9	6H7J_1:SYM00010000 D:6H7J C:11 12 127 14 19 5 6 7 8 9:1	1
6U55_1	11 120 12 127 128 13 14 19 26 27 3 5 6 7 8 9	6H7J_1:SYM00010000 D:6H7J C:11 12 127 14 19 5 6 7 8 9:1	1
6VI4_1	8 9 11 12 14 17 18 19 20 5 84 6 88 7	6H7J_1:SYM00010000 D:6H7J C:11 12 127 14 19 5 6 7 8 9:1	1
6VI4_2	8 9 11 12 14 17 18 19 20 5 84 6 88 7	6H7J_1:SYM00010000 D:6H7J C:11 12 127 14 19 5 6 7 8 9:1	1
6WAR_1	11 120 123 12 26 5 9	6H7J_1:SYM00010000 D:6H7J C:11 12 127 14 19 5 6 7 8 9:1	1
6WAR_3	11 120 123 12 26 5 9	6H7J_1:SYM00010000 D:6H7J C:11 12 127 14 19 5 6 7 8 9:1	1
4DK3_1	14 19 26 7 8 9	6H7J_1:SYM00010000 D:6H7J C:11 12 127 14 19 5 6 7 8 9:1	1
4DK3_3	11 12 120 13 127 14 19 26 5 6 7 8 9	6H7J_1:SYM00010000 D:6H7J C:11 12 127 14 19 5 6 7 8 9:1	1
4KDT_1	11 12 13 123 15 20 21 27 4 5 6 7 8 9	6H7J_1:SYM00010000 D:6H7J C:11 12 127 14 19 5 6 7 8 9:1	1
4KDT_2	12 13 14 16 21 22 28 4 5 6 7 8 9 11	6H7J_1:SYM00010000 D:6H7J C:11 12 127 14 19 5 6 7 8 9:1	1
6GK4_1	11 12 13 14 19 5 6 7 8 9	6H7J_1:SYM00010000 D:6H7J C:11 12 127 14 19 5 6 7 8 9:1	1
6GK4_2	11 12 13 14 19 5 6 7 8 9	6H7J_1:SYM00010000 D:6H7J C:11 12 127 14 19 5 6 7 8 9:1	1
6ITQ_2	11 12 24 5 7 8 9	6H7J_1:SYM00010000 D:6H7J C:11 12 127 14 19 5 6 7 8 9:1	1
6ITQ_3	11 12 24 5 7 8 9	6H7J_1:SYM00010000 D:6H7J C:11 12 127 14 19 5 6 7 8 9:1	1
2X89_1	118 120 123 44 47 50 103	5BOP_1:SYM00-10000 A:5BOP C:120 121 44 46 47 101 103:2	2
2X89_2	118 120 44 47 50 103	5BOP_1:SYM00-10000 A:5BOP C:120 121 44 46 47 101 103:2	2
4GRW_1	120 121 123 125 44 46 47 101 103	5BOP_1:SYM00-10000 A:5BOP C:120 121 44 46 47 101 103:2	2
4GRW_2	120 121 123 125 44 46 47 101 103	5BOP_1:SYM00-10000 A:5BOP C:120 121 44 46 47 101 103:2	2
5BOP_1	120 121 44 46 47 101 103	5BOP_1:SYM00-10000 A:5BOP C:120 121 44 46 47 101 103:2	2
5BOP_2	120 121 44 46 47 101 103	5BOP_1:SYM00-10000 A:5BOP C:120 121 44 46 47 101 103:2	2
5O8F_1	118 120 123 125 44 45 46 47 48 50 99 101 103	5BOP_1:SYM00-10000 A:5BOP C:120 121 44 46 47 101 103:2	2
5O8F_2	118 120 123 125 44 45 46 47 48 50 99 101 103	5BOP_1:SYM00-10000 A:5BOP C:120 121 44 46 47 101 103:2	2
5O8F_3	118 120 125 44 45 46 47 48 50 101 103	5BOP_1:SYM00-10000 A:5BOP C:120 121 44 46 47 101 103:2	2
5O8F_4	118 120 125 44 45 46 47 48 50 101 103	5BOP_1:SYM00-10000 A:5BOP C:120 121 44 46 47 101 103:2	2
5O8F_5	118 120 125 44 46 47 48 50 101 103	5BOP_1:SYM00-10000 A:5BOP C:120 121 44 46 47 101 103:2	2
5OCL_1	120 121 123 44 46 47 48 49 103	5BOP_1:SYM00-10000 A:5BOP C:120 121 44 46 47 101 103:2	2
5OCL_2	120 121 123 44 46 47 48 49 103	5BOP_1:SYM00-10000 A:5BOP C:120 121 44 46 47 101 103:2	2
6DBE_1	120 121 122 125 45 47 48 51 102 104	5BOP_1:SYM00-10000 A:5BOP C:120 121 44 46 47 101 103:2	2

Table S2 Cluster Result Coordinate Log file

Pattern name	Pattern	Reference pattern	Class
6DBE_2	120 121 122 125 45 47 48 51 102 104	5BOP_1:SYM00-10000 A:5BOP C:120 121 44 46 47 101 103:2	2
6GKD_1	120 121 123 46 47 101 103	5BOP_1:SYM00-10000 A:5BOP C:120 121 44 46 47 101 103:2	2
6GKD_2	120 121 123 46 47 101 103	5BOP_1:SYM00-10000 A:5BOP C:120 121 44 46 47 101 103:2	2
6HD8_1	118 119 120 44 48 49	5BOP_1:SYM00-10000 A:5BOP C:120 121 44 46 47 101 103:2	2
6HD9_1	118 119 120 44 48 49 4	5BOP_1:SYM00-10000 A:5BOP C:120 121 44 46 47 101 103:2	2
6HDB_2	118 119 120 44 48 49	5BOP_1:SYM00-10000 A:5BOP C:120 121 44 46 47 101 103:2	2
6N50_1	115 118 44 45 46 47 101	5BOP_1:SYM00-10000 A:5BOP C:120 121 44 46 47 101 103:2	2
3DWT_1	107 108 111B 113 114 115 116	3DWT_9:3DWT C:3DWT B:108 109 110 111B 113 114 115 116:3	3
3DWT_3	108 109 110 111B 113 114 116 36	3DWT_9:3DWT C:3DWT B:108 109 110 111B 113 114 115 116:3	3
3DWT_4	108 109 110 111B 113 114 116	3DWT_9:3DWT C:3DWT B:108 109 110 111B 113 114 115 116:3	3
3DWT_5	108 109 110 111B 113 114 115 116 36	3DWT_9:3DWT C:3DWT B:108 109 110 111B 113 114 115 116:3	3
3DWT_6	107 108 111B 113 114 116	3DWT_9:3DWT C:3DWT B:108 109 110 111B 113 114 115 116:3	3
3DWT_7	107 108 111B 113 114 116	3DWT_9:3DWT C:3DWT B:108 109 110 111B 113 114 115 116:3	3
3DWT_8	107 108 111B 113 114 115 116	3DWT_9:3DWT C:3DWT B:108 109 110 111B 113 114 115 116:3	3
3DWT_9	108 109 110 111B 113 114 115 116	3DWT_9:3DWT C:3DWT B:108 109 110 111B 113 114 115 116:3	3
3EAK_1	18 20 62 74 77 78 79 80 81 82 83 88 90 92	5VL2_11:5VL2 A:5VL2 C:18 20 62 74 80 81 82 83 88 90 92:4	4
3EAK_2	18 20 62 74 77 78 79 80 81 82 83 88 90 92	5VL2_11:5VL2 A:5VL2 C:18 20 62 74 80 81 82 83 88 90 92:4	4
3K1K_2	18 62 63 74 77 79 80 81 82 83 90 92	5VL2_11:5VL2 A:5VL2 C:18 20 62 74 80 81 82 83 88 90 92:4	4
3K1K_3	18 59 62 63 74 77 79 80 81 82 83 90 92	5VL2_11:5VL2 A:5VL2 C:18 20 62 74 80 81 82 83 88 90 92:4	4
3OGO_1	18 77 79 80 81 82 83 88 90 92	5VL2_11:5VL2 A:5VL2 C:18 20 62 74 80 81 82 83 88 90 92:4	4
3OGO_2	18 20 77 79 80 81 82 83 88 90 92	5VL2_11:5VL2 A:5VL2 C:18 20 62 74 80 81 82 83 88 90 92:4	4
3OGO_3	18 77 79 80 81 82 83 88 90 92	5VL2_11:5VL2 A:5VL2 C:18 20 62 74 80 81 82 83 88 90 92:4	4
3OGO_4	18 20 77 79 80 81 82 83 88 90 92	5VL2_11:5VL2 A:5VL2 C:18 20 62 74 80 81 82 83 88 90 92:4	4
5E03_1	18 20 77 78 79 80 81 82 90 92 93	5VL2_11:5VL2 A:5VL2 C:18 20 62 74 80 81 82 83 88 90 92:4	4
5G5R_1	18 20 63 74 77 78 79 80 81 82 83 90 92	5VL2_11:5VL2 A:5VL2 C:18 20 62 74 80 81 82 83 88 90 92:4	4
5G5X_1	18 20 77 79 80 81 82 83 88 90 92	5VL2_11:5VL2 A:5VL2 C:18 20 62 74 80 81 82 83 88 90 92:4	4
5VL2_1	18 20 62 74 80 81 82 83 90 92	5VL2_11:5VL2 A:5VL2 C:18 20 62 74 80 81 82 83 88 90 92:4	4
5VL2_4	18 20 62 74 80 81 82 90 92	5VL2_11:5VL2 A:5VL2 C:18 20 62 74 80 81 82 83 88 90 92:4	4
5VL2_5	18 20 74 80 81 82 83 90 92	5VL2_11:5VL2 A:5VL2 C:18 20 62 74 80 81 82 83 88 90 92:4	4
5VL2_6	18 80 81 82 83 90 92	5VL2_11:5VL2 A:5VL2 C:18 20 62 74 80 81 82 83 88 90 92:4	4
5VL2_7	18 20 80 81 82 83 88 90 92	5VL2_11:5VL2 A:5VL2 C:18 20 62 74 80 81 82 83 88 90 92:4	4
5VL2_9	18 20 80 81 82 83 90 92	5VL2_11:5VL2 A:5VL2 C:18 20 62 74 80 81 82 83 88 90 92:4	4

Table S2 Cluster Result Coordinate Log file

Pattern name	Pattern	Reference pattern	Class
5VL2_11	18 20 62 74 80 81 82 83 88 90 92	5VL2_11:5VL2 A:5VL2 C:18 20 62 74 80 81 82 83 88 90 92:4	4
5VL2_12	18 20 80 81 82 83 90 92	5VL2_11:5VL2 A:5VL2 C:18 20 62 74 80 81 82 83 88 90 92:4	4
5VNV_1	19 21 64 65 76 77 78 80 82 83 84 85 86 87 91 93 95	5VL2_11:5VL2 A:5VL2 C:18 20 62 74 80 81 82 83 88 90 92:4	4
6C9W_1	17 18 20 62 63 74 77 78 79 80 81 82 83 84 88 90 92	5VL2_11:5VL2 A:5VL2 C:18 20 62 74 80 81 82 83 88 90 92:4	4
6DBE_3	20 64 75 76 78 80 81 82 83 84 85 91 93	5VL2_11:5VL2 A:5VL2 C:18 20 62 74 80 81 82 83 88 90 92:4	4
6U51_1	18 20 59 65 72 74 76 77 79 80 84 90 92	5VL2_11:5VL2 A:5VL2 C:18 20 62 74 80 81 82 83 88 90 92:4	4
4DK3_4	20 59 62 63 74 77 79 80 81 92	5VL2_11:5VL2 A:5VL2 C:18 20 62 74 80 81 82 83 88 90 92:4	4
5E03_1	18 20 77 78 79 80 81 82 90 92 93	5VL2_11:5VL2 A:5VL2 C:18 20 62 74 80 81 82 83 88 90 92:4	4
6GJQ_1	18 20 77 78 79 80 81 82 83 88 90 92	5VL2_11:5VL2 A:5VL2 C:18 20 62 74 80 81 82 83 88 90 92:4	4
6GJQ_2	18 20 77 78 79 80 81 82 83 90 92	5VL2_11:5VL2 A:5VL2 C:18 20 62 74 80 81 82 83 88 90 92:4	4
6GJQ_3	18 20 77 78 79 80 81 82 83 90 92	5VL2_11:5VL2 A:5VL2 C:18 20 62 74 80 81 82 83 88 90 92:4	4
6GJQ_4	18 20 77 78 79 80 81 82 83 88 90 92	5VL2_11:5VL2 A:5VL2 C:18 20 62 74 80 81 82 83 88 90 92:4	4
6HHU_1	14 16 17 18 20 59 63 74 77 78 79 80 81 82 83 88 90 92	5VL2_11:5VL2 A:5VL2 C:18 20 62 74 80 81 82 83 88 90 92:4	4
6ITQ_1	18 20 62 63 74 75 77 78 79 80 81 82 83 90 92	5VL2_11:5VL2 A:5VL2 C:18 20 62 74 80 81 82 83 88 90 92:4	4
6ITQ_4	18 63 74 77 78 79 80 81 82 83 90 92	5VL2_11:5VL2 A:5VL2 C:18 20 62 74 80 81 82 83 88 90 92:4	4
6ITQ_5	18 20 62 63 74 75 77 78 79 80 81 82 83 90 92	5VL2_11:5VL2 A:5VL2 C:18 20 62 74 80 81 82 83 88 90 92:4	4
6ITQ_6	18 63 74 77 78 79 80 81 82 83 90 92	5VL2_11:5VL2 A:5VL2 C:18 20 62 74 80 81 82 83 88 90 92:4	4
3EZJ_1	56 59 63 64 65 66 67 69 72	6DBD_1:6DBD C:6DBD B:63 64 65 66 67 68 69:5	5
3EZJ_2	56 59 63 64 65 66 67 69 72	6DBD_1:6DBD C:6DBD B:63 64 65 66 67 68 69:5	5
3EZJ_3	56 59 63 64 65 66 67 69 72	6DBD_1:6DBD C:6DBD B:63 64 65 66 67 68 69:5	5
3EZJ_4	56 59 63 64 65 66 67 69 72	6DBD_1:6DBD C:6DBD B:63 64 65 66 67 68 69:5	5
5FWO_2	112B 63 64 65 66 67 68	6DBD_1:6DBD C:6DBD B:63 64 65 66 67 68 69:5	5
5VNV_2	40 59 63 66 67 68 71 75	6DBD_1:6DBD C:6DBD B:63 64 65 66 67 68 69:5	5
6DBD_1	63 64 65 66 67 68 69	6DBD_1:6DBD C:6DBD B:63 64 65 66 67 68 69:5	5
6DBD_2	63 64 65 66 67 68 69	6DBD_1:6DBD C:6DBD B:63 64 65 66 67 68 69:5	5
6DBD_6	59 63 64 65 67 68 69	6DBD_1:6DBD C:6DBD B:63 64 65 66 67 68 69:5	5
6DBD_7	63 64 65 66 67 68 69	6DBD_1:6DBD C:6DBD B:63 64 65 66 67 68 69:5	5
6HD8_2	52 64 65 66 67 68 69 72	6DBD_1:6DBD C:6DBD B:63 64 65 66 67 68 69:5	5
6HD9_2	52 64 65 66 67 68 69 72	6DBD_1:6DBD C:6DBD B:63 64 65 66 67 68 69:5	5
6HDA_2	52 64 65 66 67 68 69 72	6DBD_1:6DBD C:6DBD B:63 64 65 66 67 68 69:5	5
6HDB_1	52 64 65 66 67 68 69	6DBD_1:6DBD C:6DBD B:63 64 65 66 67 68 69:5	5
6HDC_1	52 64 65 66 67 68 69 72	6DBD_1:6DBD C:6DBD B:63 64 65 66 67 68 69:5	5

Table S2 Cluster Result Coordinate Log file

Pattern name	Pattern	Reference pattern	Class
6ITP_1	111A 111B 112B 49 68 69 72 74 97	6DBD_1:6DBD C:6DBD B:63 64 65 66 67 68 69:5	5
6ITP_3	111A 111B 112B 49 68 69 72 74 97	6DBD_1:6DBD C:6DBD B:63 64 65 66 67 68 69:5	5
5NM0_1	110 37 38 39 53 58 59 108 109	5NML_4:SYM0100-101 C:5NML D:108 113 115 36 37 38 52 57 59 66 67:6	6
5NM0_3	110 37 38 39 53 58 59 65 108 109	5NML_4:SYM0100-101 C:5NML D:108 113 115 36 37 38 52 57 59 66 67:6	6
5NML_2	113 115 36 37 38 52 57 58 59 64 66 67 108	5NML_4:SYM0100-101 C:5NML D:108 113 115 36 37 38 52 57 59 66 67:6	6
5NML_3	108 112 114 36 37 38 52 57 59 64 68 107	5NML_4:SYM0100-101 C:5NML D:108 113 115 36 37 38 52 57 59 66 67:6	6
5NML_4	108 113 115 36 37 38 52 57 59 66 67	5NML_4:SYM0100-101 C:5NML D:108 113 115 36 37 38 52 57 59 66 67:6	6
5NML_5	36 37 38 57 64 107	5NML_4:SYM0100-101 C:5NML D:108 113 115 36 37 38 52 57 59 66 67:6	6
5NML_6	108 112 114 36 37 38 52 57 59 64 68 69 107	5NML_4:SYM0100-101 C:5NML D:108 113 115 36 37 38 52 57 59 66 67:6	6
5NML_7	108 112 114 36 37 38 52 57 66 68 107	5NML_4:SYM0100-101 C:5NML D:108 113 115 36 37 38 52 57 59 66 67:6	6
5NML_8	108 112 114 36 37 38 52 57 58 66 68 69 107	5NML_4:SYM0100-101 C:5NML D:108 113 115 36 37 38 52 57 59 66 67:6	6
5NML_9	36 37 38 57 64 107	5NML_4:SYM0100-101 C:5NML D:108 113 115 36 37 38 52 57 59 66 67:6	6
5NML_10	112 36 37 38 52 57 59 64 66 68 69 107	5NML_4:SYM0100-101 C:5NML D:108 113 115 36 37 38 52 57 59 66 67:6	6
5NML_13	112 36 37 38 52 57 66 68 69 107	5NML_4:SYM0100-101 C:5NML D:108 113 115 36 37 38 52 57 59 66 67:6	6
6DBD_4	110 115 116 37 38 57 59 107 109	5NML_4:SYM0100-101 C:5NML D:108 113 115 36 37 38 52 57 59 66 67:6	6
6DBD_5	110 115 116 37 38 57 59 107 109	5NML_4:SYM0100-101 C:5NML D:108 113 115 36 37 38 52 57 59 66 67:6	6
5NML_11	123 125 126 127 128 15 46 96 99 101	5VM4_14:SYM0300-100 G:5VM4 H:11 123 124 125 12 127 13 45 46 48 99 100:7	7
5NML_12	123 125 126 127 128 12 15 46 96 99 101	5VM4_14:SYM0300-100 G:5VM4 H:11 123 124 125 12 127 13 45 46 48 99 100:7	7
5OCL_3	123 125 126 127 128 12 14 15 46 96 99 100 101	5VM4_14:SYM0300-100 G:5VM4 H:11 123 124 125 12 127 13 45 46 48 99 100:7	7
5VM4_1	11 123 124 125 12 127 45 46 48 99 100 101	5VM4_14:SYM0300-100 G:5VM4 H:11 123 124 125 12 127 13 45 46 48 99 100:7	7
5VM4_2	11 123 125 12 127 13 45 46 47 48 99 100	5VM4_14:SYM0300-100 G:5VM4 H:11 123 124 125 12 127 13 45 46 48 99 100:7	7
5VM4_3	11 123 124 125 12 127 13 45 46 47 48 99 100 101	5VM4_14:SYM0300-100 G:5VM4 H:11 123 124 125 12 127 13 45 46 48 99 100:7	7
5VM4_5	11 123 125 12 127 45 46 48 99 100 101	5VM4_14:SYM0300-100 G:5VM4 H:11 123 124 125 12 127 13 45 46 48 99 100:7	7
5VM4_7	11 123 124 125 12 127 45 46 48 99 100 101	5VM4_14:SYM0300-100 G:5VM4 H:11 123 124 125 12 127 13 45 46 48 99 100:7	7
5VM4_8	11 123 125 12 127 13 45 46 48 99 100 101	5VM4_14:SYM0300-100 G:5VM4 H:11 123 124 125 12 127 13 45 46 48 99 100:7	7
5VM4_9	11 123 125 12 127 45 46 48 99 100	5VM4_14:SYM0300-100 G:5VM4 H:11 123 124 125 12 127 13 45 46 48 99 100:7	7
5VM4_10	11 123 124 125 12 127 45 46 48 99 100	5VM4_14:SYM0300-100 G:5VM4 H:11 123 124 125 12 127 13 45 46 48 99 100:7	7
5VM4_11	11 123 124 125 12 127 13 45 46 47 48 99 100 101	5VM4_14:SYM0300-100 G:5VM4 H:11 123 124 125 12 127 13 45 46 48 99 100:7	7
5VM4_12	11 123 125 12 127 45 46 48 99 100	5VM4_14:SYM0300-100 G:5VM4 H:11 123 124 125 12 127 13 45 46 48 99 100:7	7
5VM4_13	11 123 125 12 127 13 45 46 47 48 99 100	5VM4_14:SYM0300-100 G:5VM4 H:11 123 124 125 12 127 13 45 46 48 99 100:7	7
5VM4_14	11 123 124 125 12 127 13 45 46 48 99 100	5VM4_14:SYM0300-100 G:5VM4 H:11 123 124 125 12 127 13 45 46 48 99 100:7	7
6QGY_2	123 124 125 126 127 128 15 45 46 96 97 99 100 101	5VM4_14:SYM0300-100 G:5VM4 H:11 123 124 125 12 127 13 45 46 48 99 100:7	7

Table S2 Cluster Result Coordinate Log file

Pattern name	Pattern	Reference pattern	Class
5WB1_1	128 128 128 128 128 128 128	5WB2_1:SYM02000000 A:5WB2 A:128 128 128 128 128 128 128 128 128 128 128 128 128 128:8	8
5WB1_2	128 128 128 128 128 128 128	5WB2_1:SYM02000000 A:5WB2 A:128 128 128 128 128 128 128 128 128 128 128 128 128 128:8	8
5WB2_1	128 128 128 128 128 128 128 128 128 128 128 128 128 128	5WB2_1:SYM02000000 A:5WB2 A:128 128 128 128 128 128 128 128 128 128 128 128 128 128:8	8
5WB2_2	128 128 128 128 128 128 128 128 128 128	5WB2_1:SYM02000000 A:5WB2 A:128 128 128 128 128 128 128 128 128 128 128 128 128 128:8	8
6QD6_1	128 128 128 128 128 128 128 128 128 128 128 128 128 128 128 128 128 128 128 128 128 128 128	5WB2_1:SYM02000000 A:5WB2 A:128 128 128 128 128 128 128 128 128 128 128 128 128 128:8	8
6QD6_2	128 128 128 128 128 128 128	5WB2_1:SYM02000000 A:5WB2 A:128 128 128 128 128 128 128 128 128 128 128 128 128 128:8	8
6QD6_3	128 128 128 128 128 128 128 128 128 128	5WB2_1:SYM02000000 A:5WB2 A:128 128 128 128 128 128 128 128 128 128 128 128 128 128:8	8
6QD6_4	128 128 128 128 128 128 128 128 128 128 128 128 128 128 128 128 128 128	5WB2_1:SYM02000000 A:5WB2 A:128 128 128 128 128 128 128 128 128 128 128 128 128 128:8	8
6QD6_5	128 128 128 128 128 128 128 128	5WB2_1:SYM02000000 A:5WB2 A:128 128 128 128 128 128 128 128 128 128 128 128 128 128:8	8
6QD6_6	128 128 128 128 128 128 128 128 128 128 128 128 128 128 128 128 128 128 128 128 128	5WB2_1:SYM02000000 A:5WB2 A:128 128 128 128 128 128 128 128 128 128 128 128 128 128:8	8
6QD6_7	128 128 128 128 128 128 128 128 128 128	5WB2_1:SYM02000000 A:5WB2 A:128 128 128 128 128 128 128 128 128 128 128 128 128 128:8	8
6QD6_8	128 128 128 128 128 128 128 128 128 128 128 128 128 128 128 128 128 128 128 128 128 128	5WB2_1:SYM02000000 A:5WB2 A:128 128 128 128 128 128 128 128 128 128 128 128 128 128:8	8
6QD6_9	128 128 128 128 128 128 128 128 128 128 128 128 128 128 128 128 128	5WB2_1:SYM02000000 A:5WB2 A:128 128 128 128 128 128 128 128 128 128 128 128 128 128:8	8
6QD6_10	128 128 128 128 128 128 128 128 128	5WB2_1:SYM02000000 A:5WB2 A:128 128 128 128 128 128 128 128 128 128 128 128 128 128:8	8
6QD6_11	128 128 128 128 128 128 128 128 128 128 128 128 128 128 128 128 128 128 128 128 128 128	5WB2_1:SYM02000000 A:5WB2 A:128 128 128 128 128 128 128 128 128 128 128 128 128 128:8	8
6QD6_12	128 128 128 128 128 128 128 128 128 128 128 128 128 128 128 128 128 128 128 128 128	5WB2_1:SYM02000000 A:5WB2 A:128 128 128 128 128 128 128 128 128 128 128 128 128 128:8	8
6QD6_15	128 128 128 128 128 128 128 128 128 128 128 128 128 128 128 128 128 128 128	5WB2_1:SYM02000000 A:5WB2 A:128 128 128 128 128 128 128 128 128 128 128 128 128 128:8	8
6QD6_16	128 128 128 128 128 128 128 128 128 128 128 128 128 128 128 128 128 128 128 128 128	5WB2_1:SYM02000000 A:5WB2 A:128 128 128 128 128 128 128 128 128 128 128 128 128 128:8	8
6QD6_17	128 128 128 128 128 128 128 128 128 128 128 128 128 128 128 128 128 128 128 128 128 128 128 128	5WB2_1:SYM02000000 A:5WB2 A:128 128 128 128 128 128 128 128 128 128 128 128 128 128:8	8

Table S3 Full list and the crystallization result of nanobody variants tested in the RECQL5:nanobody system

Table S3 Nanobody variant information

Nanobody mutation ID	Nanobody grouped ID	Plate barcode	Key position mutations (S7/L12/Q14/T125)	Other surface mutations around NbInter1	Epitope mutations	Solubility mutations	With C-terminal tail *AENLYFQ?	Concentration (mg/ml)	Drops with mountable crystals	Drop with crystalline species
Ctag:WT	G0-001	CI072191	-	-	-	-	yes	12.98	0	0
Ctag:[A54F:A55T]	G0-002	-	-	-	A54F:A55T	-	yes	-	-	-
Ctag:[E6Q]	G0-003	CI072048	-	-	E6Q	-	yes	10.76	0	0
Ctag:[L12W]	G0-004	CI072046	-	-	L12W	-	yes	22.68	0	0
Ctag:[T86A:V87L:Y88S]	G0-005	-	-	-	T86A:V87L:Y88S	-	yes	-	-	-
Ctag:[T78S]	G0-006	CI070268	-	-	T78S	-	yes	7.84	0	0
Ctag:[K84S]	G0-007	CI070239	-	-	K84S	-	yes	18.67	0	0
Ctag:[Q90E]	G0-008	CI072044	-	-	Q90E	-	yes	10.66	0	0
Ctag:[Y88H]	G0-009	-	-	-	Y88H	-	yes	-	-	-
Ctag:[N82S:N85G]	G0-010	-	-	-	N82S:N85G	-	yes	-	-	-
Ctag:[K95E]	G0-011	-	-	-	K95E	-	yes	-	-	-
Ctag:[N85G]	G0-012	CI072098	-	-	N85G	-	yes	11.61	0	0
Ctag:[N82S]	G0-013	CI072096	-	-	N82S	-	yes	23.93	0	0
Ctag:[N85S]	G0-014	-	-	-	N85S	-	yes	-	-	-
Ctag:[N82K:N85G]	G0-015	CI072094	-	-	N82K:N85G	-	yes	7.95	0	0
Ctag:[N82G:N85G]	G0-016	-	-	-	N82G:N85G	-	yes	-	-	-
Ctag:[S79Q]	G0-017	-	-	-	S79Q	-	yes	-	-	-
Ctag:[R20K:L21V]	G0-018	-	-	-	R20K:L21V	-	yes	-	-	-
Ctag:[A15P:G17D]	G0-019	CI072092	-	-	A15P:G17D	-	yes	10.76	0	0
Ctag:[A15P:G17A]	G0-020	CI072090	-	-	A15P:G17A	-	yes	10.65	0	0
Ctag:[A45V]	G0-021	-	-	-	A45V	-	yes	-	-	-
Ctag:[Q44R]	G0-022	-	-	-	Q44R	-	yes	-	-	-
Ctag:[L12W:D69Y]	G0-023	CI072078	-	-	L12W:D69Y	-	yes	12.32	0	0
Ctag:[A15P:G17A:D69Y]	G0-024	CI072003	-	-	A15P:G17A:D69Y	-	yes	22.17	0	0
Ctag:[A45V:D69Y]	G0-025	CI072087	-	-	A45V:D69Y	-	yes	9.07	0	0
Ctag:[D69Y:K84S]	G0-026	CI072070	-	-	D69Y:K84S	-	yes	8.23	0	0
Ctag:[D69Y:Q90E]	G0-027	-	-	-	D69Y:Q90E	-	yes	-	-	-
Ctag:[D69Y:Y88H]	G0-028	CI072086	-	-	D69Y:Y88H	-	yes	5.92	0	0
Ctag:[D69Y:N82S:N85G]	G0-029	-	-	-	D69Y:N82S:N85G	-	yes	-	-	-
Ctag:[D69Y:K95E]	G0-030	CI072075	-	-	D69Y:K95E	-	yes	18.84	0	0
Ctag:[D69Y:N82K:N85G]	G0-031	-	-	-	D69Y:N82K:N85G	-	yes	-	-	-
Ctag:[D69Y:N82G:N85G]	G0-032	CI072079	-	-	D69Y:N82G:N85G	-	yes	8.99	0	0
Ctag:L12W:[K84S]	G0-033	CI072073	L12W	-	K84S	-	yes	25.40	0	0
Ctag:L12W:[Q90E]	G0-034	CI072072	L12W	-	Q90E	-	yes	23.50	0	0
Ctag:L12W:[Y88H]	G0-035	CI072000	L12W	-	Y88H	-	yes	19.00	0	0
Ctag:L12W:[N82S:N85G]	G0-036	CI072001	L12W	-	N82S:N85G	-	yes	19.50	0	0
Ctag:L12W:[K95E]	G0-037	CI072008	L12W	-	K95E	-	yes	19.33	0	0
Ctag:L12W:[N82K:N85G]	G0-038	CI072007	L12W	-	N82K:N85G	-	yes	21.27	0	0
Ctag:L12W:[N82G:N85G]	G0-039	CI072071	L12W	-	N82G:N85G	-	yes	20.90	0	0
Ctag:[A15P:G17D:K84S]	G0-040	CI072002	-	-	A15P:G17D:K84S	-	yes	21.80	0	0
Ctag:[A15P:G17D:Q90E]	G0-041	CI072004	-	-	A15P:G17D:Q90E	-	yes	20.00	0	0
Ctag:[A15P:G17D:Y88H]	G0-042	CI072074	-	-	A15P:G17D:Y88H	-	yes	21.80	0	0
Ctag:[A15P:G17D:N82S:N85G]	G0-043	-	-	-	A15P:G17D:N82S:N85G	-	yes	-	-	-
Ctag:[A15P:G17D:K95E]	G0-044	-	-	-	A15P:G17D:K95E	-	yes	-	-	-
Ctag:[A15P:G17D:N82K:N85G]	G0-045	CI072076	-	-	A15P:G17D:N82K:N85G	-	yes	8.50	0	0
Ctag:[A15P:G17D:N82G:N85G]	G0-046	-	-	-	A15P:G17D:N82G:N85G	-	yes	-	-	-
Ctag:[A15P:G17A:K84S]	G0-047	CI072077	-	-	A15P:G17A:K84S	-	yes	20.60	0	0
Ctag:[A15P:G17A:Q90E]	G0-048	-	-	-	A15P:G17A:Q90E	-	yes	-	-	-
Ctag:[A15P:G17A:Y88H]	G0-049	-	-	-	A15P:G17A:Y88H	-	yes	-	-	-
Ctag:[A15P:G17A:N82S:N85G]	G0-050	-	-	-	A15P:G17A:N82S:N85G	-	yes	-	-	-
Ctag:[A15P:G17A:K95E]	G0-051	-	-	-	A15P:G17A:K95E	-	yes	-	-	-
Ctag:[A15P:G17A:N82K:N85G]	G0-052	-	-	-	A15P:G17A:N82K:N85G	-	yes	-	-	-

Table S3 Nanobody variant information

Nanobody mutation ID	Nanobody grouped ID	Plate barcode	Key position mutations (S7/L12/Q14/T125)	Other surface mutations around NbInter1	Epitope mutations	Solubility mutations	With C-terminal tail *AENLYFQ?	Concentration (mg/ml)	Drops with mountable crystals	Drop with crystalline species
Ctag:[A15P:G17A:N82G:N85G]	G0-053	-	-	-	A15P:G17A:N82G:N85G	-	yes	-	-	-
Ctag:L12W:[D69Y:K84S]	G0-054	CI072152	L12W	-	D69Y:K84S	-	yes	10.43	6	6
Ctag:L12W:[D69Y:Q90E]	G0-055	-	L12W	-	D69Y:Q90E	-	yes	-	-	-
Ctag:L12W:[D69Y:Y88H]	G0-056	CI072154	L12W	-	D69Y:Y88H	-	yes	10.15	2	19
Ctag:L12W:[D69Y:N82S:N85G]	G0-057	CI066052	L12W	-	D69Y:N82S:N85G	-	yes	14.05	4	10
Ctag:L12W:[D69Y:K95E]	G0-058	-	L12W	-	D69Y:K95E	-	yes	-	-	-
Ctag:L12W:[D69Y:N82K:N85G]	G0-059	-	L12W	-	D69Y:N82K:N85G	-	yes	-	-	-
Ctag:L12W:[D69Y:N82G:N85G]	G0-060	CI072017	L12W	-	D69Y:N82G:N85G	-	yes	16.70	0	4
Ctag:[D69Y:A15P:G17D:K84S]	G0-061	-	-	-	D69Y:A15P:G17D:K84S	-	yes	-	-	-
Ctag:[D69Y:A15P:G17D:Q90E]	G0-062	CI066051	-	-	D69Y:A15P:G17D:Q90E	-	yes	21.31	0	0
Ctag:[D69Y:A15P:G17D:Y88H]	G0-063	CI072015	-	-	D69Y:A15P:G17D:Y88H	-	yes	58.19	0	0
Ctag:[D69Y:A15P:G17D:N82S:N85G]	G0-064	CI072014	-	-	D69Y:A15P:G17D:N82S:N85G	-	yes	40.33	0	0
Ctag:[D69Y:A15P:G17D:K95E]	G0-065	CI072039	-	-	D69Y:A15P:G17D:K95E	-	yes	40.16	0	0
Ctag:[D69Y:A15P:G17D:N82K:N85G]	G0-066	CI072038	-	-	D69Y:A15P:G17D:N82K:N85G	-	yes	58.83	0	0
Ctag:[D69Y:A15P:G17D:N82G:N85G]	G0-067	-	-	-	D69Y:A15P:G17D:N82G:N85G	-	yes	-	-	-
Ctag:[D69Y:A15P:G17A:K84S]	G0-068	CI072037	-	-	D69Y:A15P:G17A:K84S	-	yes	42.82	0	0
Ctag:[D69Y:A15P:G17A:Q90E]	G0-069	CI072036	-	-	D69Y:A15P:G17A:Q90E	-	yes	33.30	0	0
Ctag:[D69Y:A15P:G17A:Y88H]	G0-070	-	-	-	D69Y:A15P:G17A:Y88H	-	yes	-	-	-
Ctag:[D69Y:A15P:G17A:N82S:N85G]	G0-071	CI072035	-	-	D69Y:A15P:G17A:N82S:N85G	-	yes	41.73	0	0
Ctag:[D69Y:A15P:G17A:K95E]	G0-072	-	-	-	D69Y:A15P:G17A:K95E	-	yes	-	-	-
Ctag:[D69Y:A15P:G17A:N82K:N85G]	G0-073	-	-	-	D69Y:A15P:G17A:N82K:N85G	-	yes	-	-	-
Ctag:[D69Y:A15P:G17A:N82G:N85G]	G0-074	-	-	-	D69Y:A15P:G17A:N82G:N85G	-	yes	-	-	-
Ctag:[D69Y]	G1-001	CI072150	-	-	D69Y	-	yes	11.46	1	1
Ctag:S7N:L12C:[D69Y]	G2-001	CI078699	S7N:L12C	-	D69Y	-	Yes	12.07	56	78
Ctag:(L12S):[D69Y]	G1-002	-	-	L12S	D69Y	-	yes	-	-	-
Ctag:L12T:[D69Y]	G1-003	CI079052	L12T	-	D69Y	-	Yes	12.09	0	0
Ctag:(L12H):[D69Y]	G1-004	-	-	L12H	D69Y	-	yes	-	-	-
Ctag:L12N:[D69Y]	G1-005	CI066014	L12N	-	D69Y	-	Yes	10.72	0	1
Ctag:(L12Q):[D69Y]	G1-006	-	-	L12Q	D69Y	-	yes	-	-	-
Ctag:(L12E):[D69Y]	G1-007	-	-	L12E	D69Y	-	yes	-	-	-
Ctag:(L12D):[D69Y]	G1-008	-	-	L12D	D69Y	-	yes	-	-	-
Ctag:(L12Y):[D69Y]	G1-009	-	-	L12Y	D69Y	-	yes	-	-	-
Ctag:(S7H):[D69Y]	G1-010	-	-	S7H	D69Y	-	yes	-	-	-
Ctag:(S7T):[D69Y]	G1-011	-	-	S7T	D69Y	-	yes	-	-	-
Ctag:(S7N):[D69Y]	G1-012	-	-	S7N	D69Y	-	yes	-	-	-
Ctag:(S7Q):[D69Y]	G1-013	-	-	S7Q	D69Y	-	yes	-	-	-
Ctag:(S7Y):[D69Y]	G1-014	-	-	S7Y	D69Y	-	yes	-	-	-
Ctag:(G8H):[D69Y]	G1-015	-	-	G8H	D69Y	-	yes	-	-	-
Ctag:(G8K):[D69Y]	G1-016	-	-	G8K	D69Y	-	yes	-	-	-
Ctag:(G8N):[D69Y]	G1-017	-	-	G8N	D69Y	-	yes	-	-	-
Ctag:(G8Q):[D69Y]	G1-018	-	-	G8Q	D69Y	-	yes	-	-	-
Ctag:(G8R):[D69Y]	G1-019	-	-	G8R	D69Y	-	yes	-	-	-
Ctag:(G8E):[D69Y]	G1-020	-	-	G8E	D69Y	-	yes	-	-	-
Ctag:(G8D):[D69Y]	G1-021	CI066089	-	G8D	D69Y	-	Yes	11.38	1	7
Ctag:Q14D:[D69Y]	G1-022	CI066088	Q14D	-	D69Y	-	Yes	14.39	1	4
Ctag:Q14K:[D69Y]	G1-023	CI066087	Q14K	-	D69Y	-	Yes	12.38	0	1
Ctag:Q14H:[D69Y]	G1-024	CI066086	Q14H	-	D69Y	-	Yes	10.90	0	0
Ctag:Q14R:[D69Y]	G1-025	CI066085	Q14R	-	D69Y	-	Yes	9.61	0	0
Ctag:(Q14N):[D69Y]	G1-026	-	Q14N	-	D69Y	-	yes	-	-	-
Ctag:(G16K):[D69Y]	G1-027	-	-	G16K	D69Y	-	yes	-	-	-
Ctag:(G16R):[D69Y]	G1-028	-	-	G16R	D69Y	-	yes	-	-	-
Ctag:(G16D):[D69Y]	G1-029	CI079081	-	G16D	D69Y	-	Yes	11.48	0	0
Ctag:(G16E):[D69Y]	G1-030	-	-	G16E	D69Y	-	yes	-	-	-

Table S3 Nanobody variant information

Nanobody mutation ID	Nanobody grouped ID	Plate barcode	Key position mutations (S7/L12/Q14/T125)	Other surface mutations around NbInter1	Epitope mutations	Solubility mutations	With C-terminal tail *AENLYFQ?	Concentration (mg/ml)	Drops with mountable crystals	Drop with crystalline species
Ctag:(G16N):[D69Y]	G1-031	-	-	G16N	D69Y	-	yes	-	-	-
Ctag:(G16Q):[D69Y]	G1-032	CI066084	-	G16Q	D69Y	-	Yes	9.21	0	0
Ctag:(G16Y):[D69Y]	G1-033	-	-	G16Y	D69Y	-	yes	-	-	-
Ctag:(G16H):[D69Y]	G1-034	-	-	G16H	D69Y	-	yes	-	-	-
Ctag:(G16T):[D69Y]	G1-035	CI066083	-	G16T	D69Y	-	Yes	12.90	0	3
Ctag:(G17K):[D69Y]	G1-036	-	-	G17K	D69Y	-	yes	-	-	-
Ctag:(G17S):[D69Y]	G1-037	-	-	G17S	D69Y	-	yes	-	-	-
Ctag:(G17D):[D69Y]	G1-038	-	-	G17D	D69Y	-	yes	-	-	-
Ctag:(G17E):[D69Y]	G1-039	CI066082	-	G17E	D69Y	-	Yes	9.25	0	2
Ctag:(G17N):[D69Y]	G1-040	-	-	G17N	D69Y	-	yes	-	-	-
Ctag:(G17Q):[D69Y]	G1-041	CI066081	-	G17Q	D69Y	-	Yes	11.37	0	0
Ctag:(G17H):[D69Y]	G1-042	CI079053	-	G17H	D69Y	-	Yes	11.88	0	0
Ctag:(G17T):[D69Y]	G1-043	-	-	G17T	D69Y	-	yes	-	-	-
Ctag:(S18K):[D69Y]	G1-044	-	-	S18K	D69Y	-	yes	-	-	-
Ctag:(S18R):[D69Y]	G1-045	-	-	S18R	D69Y	-	yes	-	-	-
Ctag:(S18D):[D69Y]	G1-046	CI079054	-	S18D	D69Y	-	Yes	13.05	0	0
Ctag:(S18E):[D69Y]	G1-047	CI079055	-	S18E	D69Y	-	Yes	12.16	0	0
Ctag:(S18N):[D69Y]	G1-048	-	-	S18N	D69Y	-	yes	-	-	-
Ctag:(S18Q):[D69Y]	G1-049	-	-	S18Q	D69Y	-	yes	-	-	-
Ctag:(S18T):[D69Y]	G1-050	-	-	S18T	D69Y	-	yes	-	-	-
Ctag:(L19K):[D69Y]	G1-051	CI066080	-	L19K	D69Y	-	Yes	15.19	0	0
Ctag:(L19R):[D69Y]	G1-052	-	-	L19R	D69Y	-	yes	-	-	-
Ctag:(L19E):[D69Y]	G1-053	CI066011	-	L19E	D69Y	-	Yes	11.09	0	0
Ctag:(L19Q):[D69Y]	G1-054	-	-	L19Q	D69Y	-	yes	-	-	-
Ctag:(L19N):[D69Y]	G1-055	CI066010	-	L19N	D69Y	-	Yes	10.72	0	0
Ctag:(L19C):[D69Y]	G1-056	-	-	L19C	D69Y	-	yes	-	-	-
Ctag:(S18C:R20C):[D69Y]	G1-057	-	-	S18C:R20C	D69Y	-	yes	-	-	-
Ctag:(R20E):[D69Y]	G1-058	-	-	R20E	D69Y	-	yes	-	-	-
Ctag:(R20D):[D69Y]	G1-059	-	-	R20D	D69Y	-	yes	-	-	-
Ctag:(R20N):[D69Y]	G1-060	CI066039	-	R20N	D69Y	-	Yes	11.38	0	0
Ctag:(R20K):[D69Y]	G1-061	CI066019	-	R20K	D69Y	-	Yes	12.00	5	8
Ctag:(R20K):[D69Y]	G1-061	CI079082	-	R20K	D69Y	-	Yes	13.10	4	18
Ctag:(R20Y):[D69Y]	G1-062	CI066037	-	R20Y	D69Y	-	Yes	11.92	0	0
Ctag:(R20H):[D69Y]	G1-063	-	-	R20H	D69Y	-	yes	-	-	-
Ctag:(R20Q):[D69Y]	G1-064	CI066036	-	R20Q	D69Y	-	Yes	11.39	0	0
Ctag:(Q90K):[D69Y]	G1-065	-	-	Q90K	D69Y	-	yes	-	-	-
Ctag:(Q90R):[D69Y]	G1-066	CI066035	-	Q90R	D69Y	-	Yes	14.12	3	10
Ctag:(Q90Y):[D69Y]	G1-067	CI066034	-	Q90Y	D69Y	-	Yes	10.47	2	4
Ctag:(Q90Y):[D69Y]	G1-067	CI079080	-	Q90Y	D69Y	-	Yes	12.78	8	19
Ctag:(Q90H):[D69Y]	G1-068	CI066033	-	Q90H	D69Y	-	Yes	14.35	11	20
Ctag:(Q90H):[D69Y]	G1-068	CI079094	-	Q90H	D69Y	-	Yes	11.74	13	19
Ctag:(N92K):[D69Y]	G1-069	CI066032	-	N92K	D69Y	-	Yes	13.25	0	0
Ctag:(N92R):[D69Y]	G1-070	-	-	N92R	D69Y	-	yes	-	-	-
Ctag:(N92D):[D69Y]	G1-071	CI066031	-	N92D	D69Y	-	Yes	13.27	1	3
Ctag:(N92D):[D69Y]	G1-071	CI079093	-	N92D	D69Y	-	Yes	12.15	4	6
Ctag:(N92E):[D69Y]	G1-072	-	-	N92E	D69Y	-	yes	-	-	-
Ctag:(N92Q):[D69Y]	G1-073	-	-	N92Q	D69Y	-	yes	-	-	-
Ctag:(N92Y):[D69Y]	G1-074	-	-	N92Y	D69Y	-	yes	-	-	-
Ctag:(N92H):[D69Y]	G1-075	-	-	N92H	D69Y	-	yes	-	-	-
Ctag:(Q90C:N92C):[D69Y]	G1-076	-	-	Q90C:N92C	D69Y	-	yes	-	-	-
Ctag:(Q90E:N92E):[D69Y]	G1-077	-	-	Q90E:N92E	D69Y	-	yes	-	-	-
Ctag:(R20C):[D69Y]	G1-078	CI066030	-	R20C	D69Y	-	Yes	14.76	0	0
Ctag:(S18H:R20H):[D69Y]	G1-079	CI066013	-	S18H:R20H	D69Y	-	Yes	12.56	0	0
Ctag:(S18E:R20E):[D69Y]	G1-080	CI066012	-	S18E:R20E	D69Y	-	Yes	13.29	0	0
Ctag:(S18Y:R20Y):[D69Y]	G1-081	-	-	S18Y:R20Y	D69Y	-	yes	-	-	-
Ctag:(S18K:R20K):[D69Y]	G1-082	CI066029	-	S18K:R20K	D69Y	-	Yes	13.82	0	0
Ctag:(S18Q:R20Q):[D69Y]	G1-083	CI066028	-	S18Q:R20Q	D69Y	-	Yes	9.52	0	0
Ctag:(S18C:R20C):[D69Y]	G1-084	-	-	S18C:R20C	D69Y	-	yes	-	-	-
Ctag:(S18H:R20Y):[D69Y]	G1-085	CI066027	-	S18H:R20Y	D69Y	-	Yes	11.02	0	0
Ctag:(S18H:R20K):[D69Y]	G1-086	-	-	S18H:R20K	D69Y	-	yes	-	-	-
Ctag:(S18K:R20H):[D69Y]	G1-087	CI066026	-	S18K:R20H	D69Y	-	Yes	12.12	0	0
Ctag:(S18Q:R20H):[D69Y]	G1-088	-	-	S18Q:R20H	D69Y	-	yes	-	-	-
Ctag:(S18Y:R20H):[D69Y]	G1-089	CI066025	-	S18Y:R20H	D69Y	-	Yes	9.62	0	0
Ctag:(S18H:R20Q):[D69Y]	G1-090	CI066024	-	S18H:R20Q	D69Y	-	Yes	14.34	0	0
Ctag:(S18N:R20Q):[D69Y]	G1-091	-	-	S18N:R20Q	D69Y	-	yes	-	-	-
Ntag:S7N:L12C:(G8D):[E6Q]:SOL	G3-001	CI079713	S7N:L12C	G8D	E6Q	G40T:Q49E:L52W:I101V	no	27.53	0	0
Ntag:S7N:L12C:Q14D:[E6Q]:SOL	G3-002	CI079714	S7N:L12C:Q14D	-	E6Q	G40T:Q49E:L52W:I101V	no	37.98	1	17
Ntag:S7N:L12C:Q14K:[E6Q]:SOL	G3-003	CI079715	S7N:L12C:Q14K	-	E6Q	G40T:Q49E:L52W:I101V	no	34.27	11	19
Ntag:S7N:L12C:(G16T):[E6Q]:SOL	G3-004	CI079716	S7N:L12C	G16T	E6Q	G40T:Q49E:L52W:I101V	no	29.70	6	22
Ntag:S7N:L12C:(G17E):[E6Q]:SOL	G3-005	-	S7N:L12C	G17E	E6Q	G40T:Q49E:L52W:I101V	no	-	-	-
Ntag:S7N:L12C:(R20K):[E6Q]:SOL	G3-006	CI079717	S7N:L12C	R20K	E6Q	G40T:Q49E:L52W:I101V	no	27.90	14	36
Ntag:S7N:L12C:(Q90R):[E6Q]:SOL	G3-007	CI079730	S7N:L12C	Q90R	E6Q	G40T:Q49E:L52W:I101V	no	33.27	2	11
Ntag:S7N:L12C:(Q90Y):[E6Q]:SOL	G3-008	CI079731	S7N:L12C	Q90Y	E6Q	G40T:Q49E:L52W:I101V	no	32.21	5	24
Ntag:S7N:L12C:(Q90H):[E6Q]:SOL	G3-009	CI079732	S7N:L12C	Q90H	E6Q	G40T:Q49E:L52W:I101V	no	35.10	6	25
Ntag:S7N:L12C:(N92D):[E6Q]:SOL	G3-010	CI079733	S7N:L12C	N92D	E6Q	G40T:Q49E:L52W:I101V	no	33.78	6	33
Ntag:S7N:L12N:[E6Q]:SOL	G3-011	CI079734	S7N:L12N	-	E6Q	G40T:Q49E:L52W:I101V	no	38.39	0	5
Ntag:S7N:L12C:(G8D):[T86A:V87L:Y88S]:SOL	G3-012	-	S7N:L12C	G8D	T86A:V87L:Y88S	G40T:Q49E:L52W:I101V	no	-	-	-

Table S3 Nanobody variant information

Nanobody mutation ID	Nanobody grouped ID	Plate barcode	Key position mutations (S7/L12/Q14/T125)	Other surface mutations around NbInter1	Epitope mutations	Solubility mutations	With C-terminal tail *AENLYFQ?	Concentration (mg/ml)	Drops with mountable crystals	Drop with crystalline species
Ntag:S7N:L12C:(Q14D):[T86A:V87L:Y88S]:SOL	G3-013	-	S7N:L12C:Q14D	-	T86A:V87L:Y88S	G40T:Q49E:L52W:I101V	no	-	-	-
Ntag:S7N:L12C:(Q14K):[T86A:V87L:Y88S]:SOL	G3-014	-	S7N:L12C:Q14K	-	T86A:V87L:Y88S	G40T:Q49E:L52W:I101V	no	-	-	-
Ntag:S7N:L12C:(G16T):[T86A:V87L:Y88S]:SOL	G3-015	-	S7N:L12C	G16T	T86A:V87L:Y88S	G40T:Q49E:L52W:I101V	no	-	-	-
Ntag:S7N:L12C:(G17E):[T86A:V87L:Y88S]:SOL	G3-016	-	S7N:L12C	G17E	T86A:V87L:Y88S	G40T:Q49E:L52W:I101V	no	-	-	-
Ntag:S7N:L12C:(R20K):[T86A:V87L:Y88S]:SOL	G3-017	CI079735	S7N:L12C	R20K	T86A:V87L:Y88S	G40T:Q49E:L52W:I101V	no	30.30	14	30
Ntag:S7N:L12C:(Q90R):[T86A:V87L:Y88S]:SOL	G3-018	-	S7N:L12C	Q90R	T86A:V87L:Y88S	G40T:Q49E:L52W:I101V	no	-	-	-
Ntag:S7N:L12C:(Q90Y):[T86A:V87L:Y88S]:SOL	G3-019	CI079736	S7N:L12C	Q90Y	T86A:V87L:Y88S	G40T:Q49E:L52W:I101V	no	26.53	7	15
Ntag:S7N:L12C:(Q90H):[T86A:V87L:Y88S]:SOL	G3-020	CI079737	S7N:L12C	Q90H	T86A:V87L:Y88S	G40T:Q49E:L52W:I101V	no	29.99	7	31
Ntag:S7N:L12C:(N92D):[T86A:V87L:Y88S]:SOL	G3-021	-	S7N:L12C	N92D	T86A:V87L:Y88S	G40T:Q49E:L52W:I101V	no	-	-	-
Ntag:S7N:L12N:[T86A:V87L:Y88S]:SOL	G3-022	CI079751	S7N:L12N	-	T86A:V87L:Y88S	G40T:Q49E:L52W:I101V	no	30.86	0	2
Ntag:S7N:L12C:(G8D):[Y88H]:SOL	G3-023	CI079752	S7N:L12C	G8D	Y88H	G40T:Q49E:L52W:I101V	no	33.51	0	0
Ntag:S7N:L12C:(Q14D):[Y88H]:SOL	G3-024	-	S7N:L12C:Q14D	-	Y88H	G40T:Q49E:L52W:I101V	no	-	-	-
Ntag:S7N:L12C:Q14K:[Y88H]:SOL	G3-025	CI079759	S7N:L12C:Q14K	-	Y88H	G40T:Q49E:L52W:I101V	no	35.00	2	5
Ntag:S7N:L12C:(G16T):[Y88H]:SOL	G3-026	CI079753	S7N:L12C	G16T	Y88H	G40T:Q49E:L52W:I101V	no	21.34	5	8
Ntag:S7N:L12C:(G17E):[Y88H]:SOL	G3-027	CI079754	S7N:L12C	G17E	Y88H	G40T:Q49E:L52W:I101V	no	29.49	0	0
Ntag:S7N:L12C:(R20K):[Y88H]:SOL	G3-028	CI079755	S7N:L12C	R20K	Y88H	G40T:Q49E:L52W:I101V	no	36.51	20	26
Ntag:S7N:L12C:(Q90R):[Y88H]:SOL	G3-029	CI079756	S7N:L12C	Q90R	Y88H	G40T:Q49E:L52W:I101V	no	34.25	12	36
Ntag:S7N:L12C:(Q90Y):[Y88H]:SOL	G3-030	CI079757	S7N:L12C	Q90Y	Y88H	G40T:Q49E:L52W:I101V	no	32.58	15	34
Ntag:S7N:L12C:(Q90H):[Y88H]:SOL	G3-031	CI079296	S7N:L12C	Q90H	Y88H	G40T:Q49E:L52W:I101V	no	30.89	8	33
Ntag:S7N:L12C:(N92D):[Y88H]:SOL	G3-032	-	S7N:L12C	N92D	Y88H	G40T:Q49E:L52W:I101V	no	-	-	-
Ntag:S7N:L12N:[Y88H]:SOL	G3-033	CI079297	S7N:L12N	-	Y88H	G40T:Q49E:L52W:I101V	no	34.02	0	1
Ntag:S7N:L12C:(G8D):[A54F:A55T]:SOL	G3-034	-	S7N:L12C	G8D	A54F:A55T	G40T:Q49E:L52W:I101V	no	-	-	-
Ntag:S7N:L12C:(Q14D):[A54F:A55T]:SOL	G3-035	-	S7N:L12C:Q14D	-	A54F:A55T	G40T:Q49E:L52W:I101V	no	-	-	-
Ntag:S7N:L12C:(Q14K):[A54F:A55T]:SOL	G3-036	-	S7N:L12C:Q14K	-	A54F:A55T	G40T:Q49E:L52W:I101V	no	-	-	-
Ntag:S7N:L12C:(G16T):[A54F:A55T]:SOL	G3-037	-	S7N:L12C	G16T	A54F:A55T	G40T:Q49E:L52W:I101V	no	-	-	-
Ntag:S7N:L12C:(G17E):[A54F:A55T]:SOL	G3-038	-	S7N:L12C	G17E	A54F:A55T	G40T:Q49E:L52W:I101V	no	-	-	-
Ntag:S7N:L12C:(R20K):[A54F:A55T]:SOL	G3-039	-	S7N:L12C	R20K	A54F:A55T	G40T:Q49E:L52W:I101V	no	-	-	-
Ntag:S7N:L12C:(Q90R):[A54F:A55T]:SOL	G3-040	-	S7N:L12C	Q90R	A54F:A55T	G40T:Q49E:L52W:I101V	no	-	-	-
Ntag:S7N:L12C:(Q90Y):[A54F:A55T]:SOL	G3-041	-	S7N:L12C	Q90Y	A54F:A55T	G40T:Q49E:L52W:I101V	no	-	-	-
Ntag:S7N:L12C:(Q90H):[A54F:A55T]:SOL	G3-042	-	S7N:L12C	Q90H	A54F:A55T	G40T:Q49E:L52W:I101V	no	-	-	-
Ntag:S7N:L12C:(N92D):[A54F:A55T]:SOL	G3-043	-	S7N:L12C	N92D	A54F:A55T	G40T:Q49E:L52W:I101V	no	-	-	-
Ntag:S7N:L12N:[A54F:A55T]:SOL	G3-044	-	S7N:L12N	-	A54F:A55T	G40T:Q49E:L52W:I101V	no	-	-	-
Ntag:S7N:L12C:(G8D):[N82S:N85G]:SOL	G3-045	-	S7N:L12C	G8D	N82S:N85G	G40T:Q49E:L52W:I101V	no	-	-	-
Ntag:S7N:L12C:Q14D:[N82S:N85G]:SOL	G3-046	CI079298	S7N:L12C:Q14D	-	N82S:N85G	G40T:Q49E:L52W:I101V	no	35.01	2	12
Ntag:S7N:L12C:Q14K:[N82S:N85G]:SOL	G3-047	CI079299	S7N:L12C:Q14K	-	N82S:N85G	G40T:Q49E:L52W:I101V	no	35.01	1	9
Ntag:S7N:L12C:(G16T):[N82S:N85G]:SOL	G3-048	CI079205	S7N:L12C	G16T	N82S:N85G	G40T:Q49E:L52W:I101V	no	21.87	8	32
Ntag:S7N:L12C:(G16T):[N82S:N85G]:SOL	G3-048	CI080119	S7N:L12C	G16T	N82S:N85G	G40T:Q49E:L52W:I101V	no	29.84	8	47
Ntag:S7N:L12C:(G16T):[N82S:N85G]:SOL	G3-048	CI080118	S7N:L12C	G16T	N82S:N85G	G40T:Q49E:L52W:I101V	no	29.70	6	33
Ntag:S7N:L12C:(G16T):[N82S:N85G]:SOL	G3-048	CI080117	S7N:L12C	G16T	N82S:N85G	G40T:Q49E:L52W:I101V	no	28.94	6	37
Ntag:S7N:L12C:(G17E):[N82S:N85G]:SOL	G3-049	CI079206	S7N:L12C	G17E	N82S:N85G	G40T:Q49E:L52W:I101V	no	34.74	0	0
Ntag:S7N:L12C:(R20K):[N82S:N85G]:SOL	G3-050	CI079290	S7N:L12C	R20K	N82S:N85G	G40T:Q49E:L52W:I101V	no	44.82	5	34
Ntag:S7N:L12C:(Q90R):[N82S:N85G]:SOL	G3-051	CI079291	S7N:L12C	Q90R	N82S:N85G	G40T:Q49E:L52W:I101V	no	29.52	2	33
Ntag:S7N:L12C:(Q90Y):[N82S:N85G]:SOL	G3-052	CI079292	S7N:L12C	Q90Y	N82S:N85G	G40T:Q49E:L52W:I101V	no	35.01	13	41
Ntag:S7N:L12C:(Q90H):[N82S:N85G]:SOL	G3-053	CI079293	S7N:L12C	Q90H	N82S:N85G	G40T:Q49E:L52W:I101V	no	33.03	3	48

Table S3 Nanobody variant information

Nanobody mutation ID	Nanobody grouped ID	Plate barcode	Key position mutations (S7/L12/Q14/T125)	Other surface mutations around NbInter1	Epitope mutations	Solubility mutations	With C-terminal tail *AENLYFQ?	Concentration (mg/ml)	Drops with mountable crystals	Drop with crystalline species
Ntag:S7N:L12C:(N92D):[N82S:N85G]:SOL	G3-054	CI079218	S7N:L12C	N92D	N82S:N85G	G40T:Q49E:L52W:I101V	no	34.29	7	28
Ntag:S7N:L12N:[N82S:N85G]:SOL	G3-055	CI079204	S7N:L12N	-	N82S:N85G	G40T:Q49E:L52W:I101V	no	36.63	2	5
Ntag:S7N:L12N:[N82S:N85G]:SOL	G3-055	CI080269	S7N:L12N	-	N82S:N85G	G40T:Q49E:L52W:I101V	no	33.67	3	8
Ntag:S7N:L12C:(G8D):[K84E]:SOL	G3-056	CI079246	S7N:L12C	G8D	K84E	G40T:Q49E:L52W:I101V	no	30.51	0	0
Ntag:S7N:L12C:Q14D:[K84E]:SOL	G3-057	CI079247	S7N:L12C:Q14D	-	K84E	G40T:Q49E:L52W:I101V	no	34.20	0	7
Ntag:S7N:L12C:(Q14K):[K84E]:SOL	G3-058	-	S7N:L12C:Q14K	-	K84E	G40T:Q49E:L52W:I101V	no	-	-	-
Ntag:S7N:L12C:(G16T):[K84E]:SOL	G3-059	CI079248	S7N:L12C	G16T	K84E	G40T:Q49E:L52W:I101V	no	12.60	0	1
Ntag:S7N:L12C:(G17E):[K84E]:SOL	G3-060	-	S7N:L12C	G17E	K84E	G40T:Q49E:L52W:I101V	no	-	-	-
Ntag:S7N:L12C:(R20K):[K84E]:SOL	G3-061	CI079240	S7N:L12C	R20K	K84E	G40T:Q49E:L52W:I101V	no	36.00	7	36
Ntag:S7N:L12C:(Q90R):[K84E]:SOL	G3-062	CI079241	S7N:L12C	Q90R	K84E	G40T:Q49E:L52W:I101V	no	30.60	6	32
Ntag:S7N:L12C:(Q90Y):[K84E]:SOL	G3-063	CI079242	S7N:L12C	Q90Y	K84E	G40T:Q49E:L52W:I101V	no	40.50	7	60
Ntag:S7N:L12C:(Q90H):[K84E]:SOL	G3-064	-	S7N:L12C	Q90H	K84E	G40T:Q49E:L52W:I101V	no	-	-	-
Ntag:S7N:L12C:(N92D):[K84E]:SOL	G3-065	-	S7N:L12C	N92D	K84E	G40T:Q49E:L52W:I101V	no	-	-	-
Ntag:S7N:L12N:[K84E]:SOL	G3-066	-	S7N:L12N	-	K84E	G40T:Q49E:L52W:I101V	no	-	-	-
Ntag:S7N:L12C:(G8D):[S79Q]:SOL	G3-067	CI079243	S7N:L12C	G8D	S79Q	G40T:Q49E:L52W:I101V	no	34.65	0	0
Ntag:S7N:L12C:Q14D:[S79Q]:SOL	G3-068	CI079750	S7N:L12C:Q14D	-	S79Q	G40T:Q49E:L52W:I101V	no	21.44	0	0
Ntag:S7N:L12C:Q14K:[S79Q]:SOL	G3-069	CI079244	S7N:L12C:Q14K	-	S79Q	G40T:Q49E:L52W:I101V	no	32.40	0	6
Ntag:S7N:L12C:(G16T):[S79Q]:SOL	G3-070	CI079703	S7N:L12C	G16T	S79Q	G40T:Q49E:L52W:I101V	no	21.60	13	24
Ntag:S7N:L12C:(G17E):[S79Q]:SOL	G3-071	CI079704	S7N:L12C	G17E	S79Q	G40T:Q49E:L52W:I101V	no	31.29	0	0
Ntag:S7N:L12C:(R20K):[S79Q]:SOL	G3-072	CI079705	S7N:L12C	R20K	S79Q	G40T:Q49E:L52W:I101V	no	29.15	11	40
Ntag:S7N:L12C:(Q90R):[S79Q]:SOL	G3-073	CI079706	S7N:L12C	Q90R	S79Q	G40T:Q49E:L52W:I101V	no	32.94	5	40
Ntag:S7N:L12C:(Q90Y):[S79Q]:SOL	G3-074	-	S7N:L12C	Q90Y	S79Q	G40T:Q49E:L52W:I101V	no	-	-	-
Ntag:S7N:L12C:(Q90H):[S79Q]:SOL	G3-075	CI079707	S7N:L12C	Q90H	S79Q	G40T:Q49E:L52W:I101V	no	30.61	10	29
Ntag:S7N:L12C:(N92D):[S79Q]:SOL	G3-076	CI079708	S7N:L12C	N92D	S79Q	G40T:Q49E:L52W:I101V	no	38.41	8	29
Ntag:S7N:L12C:(G8D):[R20K:L21V]:SOL	G3-077	CI079709	S7N:L12C	G8D	R20K:L21V	G40T:Q49E:L52W:I101V	no	36.35	1	1
Ntag:S7N:L12C:Q14D:[R20K:L21V]:SOL	G3-078	CI079700	S7N:L12C:Q14D	-	R20K:L21V	G40T:Q49E:L52W:I101V	no	36.30	0	4
Ntag:S7N:L12C:Q14K:[R20K:L21V]:SOL	G3-079	CI079701	S7N:L12C:Q14K	-	R20K:L21V	G40T:Q49E:L52W:I101V	no	33.59	1	6
Ntag:S7N:L12C:(G16T):[R20K:L21V]:SOL	G3-080	-	S7N:L12C	G16T	R20K:L21V	G40T:Q49E:L52W:I101V	no	-	-	-
Ntag:S7N:L12C:(G17E):[R20K:L21V]:SOL	G3-081	-	S7N:L12C	G17E	R20K:L21V	G40T:Q49E:L52W:I101V	no	-	-	-
Ntag:S7N:L12C:(R20K):[R20K:L21V]:SOL	G3-082	CI079702	S7N:L12C	R20K	R20K:L21V	G40T:Q49E:L52W:I101V	no	31.67	16	40
Ntag:S7N:L12C:(Q90R):[R20K:L21V]:SOL	G3-083	-	S7N:L12C	Q90R	R20K:L21V	G40T:Q49E:L52W:I101V	no	-	-	-
Ntag:S7N:L12C:(Q90Y):[R20K:L21V]:SOL	G3-084	CI079719	S7N:L12C	Q90Y	R20K:L21V	G40T:Q49E:L52W:I101V	no	28.17	6	21
Ntag:S7N:L12C:(Q90H):[R20K:L21V]:SOL	G3-085	CI079721	S7N:L12C	Q90H	R20K:L21V	G40T:Q49E:L52W:I101V	no	35.75	17	43
Ntag:S7N:L12C:(N92D):[R20K:L21V]:SOL	G3-086	CI079428	S7N:L12C	N92D	R20K:L21V	G40T:Q49E:L52W:I101V	no	35.22	10	14
Ntag:S7N:L12C:(G8D):[Q44R]:SOL	G3-087	-	S7N:L12C	G8D	Q44R	G40T:Q49E:L52W:I101V	no	-	-	-
Ntag:S7N:L12C:(Q14D):[Q44R]:SOL	G3-088	-	S7N:L12C:Q14D	-	Q44R	G40T:Q49E:L52W:I101V	no	-	-	-
Ntag:S7N:L12C:Q14K:[Q44R]:SOL	G3-089	CI079738	S7N:L12C:Q14K	-	Q44R	G40T:Q49E:L52W:I101V	no	35.65	4	16
Ntag:S7N:L12C:(G16T):[Q44R]:SOL	G3-090	-	S7N:L12C	G16T	Q44R	G40T:Q49E:L52W:I101V	no	-	-	-
Ntag:S7N:L12C:(G17E):[Q44R]:SOL	G3-091	-	S7N:L12C	G17E	Q44R	G40T:Q49E:L52W:I101V	no	-	-	-
Ntag:S7N:L12C:(R20K):[Q44R]:SOL	G3-092	CI079739	S7N:L12C	R20K	Q44R	G40T:Q49E:L52W:I101V	no	33.48	11	56
Ntag:S7N:L12C:(Q90R):[Q44R]:SOL	G3-093	CI079720	S7N:L12C	Q90R	Q44R	G40T:Q49E:L52W:I101V	no	34.57	5	54
Ntag:S7N:L12C:(Q90Y):[Q44R]:SOL	G3-094	-	S7N:L12C	Q90Y	Q44R	G40T:Q49E:L52W:I101V	no	-	-	-
Ntag:S7N:L12C:(Q90H):[Q44R]:SOL	G3-095	CI079427	S7N:L12C	Q90H	Q44R	G40T:Q49E:L52W:I101V	no	33.39	9	64
Ntag:S7N:L12C:(N92D):[Q44R]:SOL	G3-096	CI079758	S7N:L12C	N92D	Q44R	G40T:Q49E:L52W:I101V	no	28.29	3	73

Table S3 Nanobody variant information

Nanobody mutation ID	Nanobody grouped ID	Plate barcode	Key position mutations (S7/L12/Q14/T125)	Other surface mutations around NbInter1	Epitope mutations	Solubility mutations	With C-terminal tail *AENLYFQ?	Concentration (mg/ml)	Drops with mountable crystals	Drop with crystalline species
Ntag:SOL	G2*-001	CI078933	-	-	-	G40T:Q49E: L52W:I101V	no	30.38	0	16
Ntag:[D69Y]:SOL	G2*-002	CI078934	-	-	D69Y	G40T:Q49E: L52W:I101V	no	29.10	0	22
Ntag:S7N:L12C:SOL	G2*-003	CI078935	S7N:L12C	-	-	G40T:Q49E: L52W:I101V	no	29.70	12	37
Ntag:S7N:L12C:[D69Y]:SOL	G2*-004	CI078967	S7N:L12C	-	D69Y	G40T:Q49E: L52W:I101V	no	12.00	3	20
Ntag:S7N:L12C:[D69Y]:SOL	G2*-004	CI079019	S7N:L12C	-	D69Y	G40T:Q49E: L52W:I101V	no	29.70	9	17
Ntag:S7N:L12C:[D69Y]:SOL	G2*-004	CI078926	S7N:L12C	-	D69Y	G40T:Q49E: L52W:I101V	no	32.30	28	63
Ntag:S7N:L12C:[D69Y]:SOL	G2*-004	CI078925	S7N:L12C	-	D69Y	G40T:Q49E: L52W:I101V	no	21.30	7	58
Ntag:S7N:L12C:[D69Y]:SOL	G2*-004	CI078928	S7N:L12C	-	D69Y	G40T:Q49E: L52W:I101V	no	29.00	20	54
Ntag:S7N:L12C:[D69Y]:SOL	G2*-004	CI078927	S7N:L12C	-	D69Y	G40T:Q49E: L52W:I101V	no	19.37	3	27
Ntag:S7N:L12C:(G8D):[D69Y]:SOL	G2*-005	-	S7N:L12C	G8D	D69Y	G40T:Q49E: L52W:I101V	no	-	-	-
Ntag:S7N:L12N:[D69Y]:SOL	G2*-006	CI078938	S7N:L12N	-	D69Y	G40T:Q49E: L52W:I101V	no	30.60	28	56
Ntag:S7N:L12C:Q14D:[D69Y]:SOL	G2*-007	CI078939	S7N:L12C:Q14D	-	D69Y	G40T:Q49E: L52W:I101V	no	7.47	0	2
Ntag:S7N:L12C:Q14K:[D69Y]:SOL	G2*-008	CI078975	S7N:L12C:Q14K	-	D69Y	G40T:Q49E: L52W:I101V	no	28.50	3	7
Ntag:S7N:L12C:(G17E):[D69 Y]:SOL	G2*-009	-	S7N:L12C	G17E	D69Y	G40T:Q49E: L52W:I101V	no	-	-	-
Ntag:S7N:L12C:(R20K):[D69 Y]:SOL	G2*-010	-	S7N:L12C	R20K	D69Y	G40T:Q49E: L52W:I101V	no	-	-	-
Ntag:S7N:L12C:(R20K):[D69 Y]:SOL	G2*-011	CI078977	S7N:L12C	R20K	D69Y	G40T:Q49E: L52W:I101V	no	9.54	1	6
Ntag:S7N:L12C:(Q90Y):[D69 Y]:SOL	G2*-012	-	S7N:L12C	Q90Y	D69Y	G40T:Q49E: L52W:I101V	no	-	-	-
Ntag:S7N:L12C:(Q90H):[D69 Y]:SOL	G2*-013	-	S7N:L12C	Q90H	D69Y	G40T:Q49E: L52W:I101V	no	-	-	-
Ntag:S7N:L12C:(Q90H):[D69 Y]:SOL	G2*-014	CI078979	S7N:L12C	Q90H	D69Y	G40T:Q49E: L52W:I101V	no	9.00	1	5
Ntag:S7N:L12C:(N92D):[D69 Y]:SOL	G2*-015	CI078930	S7N:L12C	N92D	D69Y	G40T:Q49E: L52W:I101V	no	18.36	11	20
Ntag:S7N:L12C:[D69Y]:SOL	G2*-016	CI078931	S7N:L12C	-	D69Y	G40T:Q49E: L52W:I101V	no	22.50	14	20
Ctag:S7N:L12C:(G8D):[D69Y]:SOL	G2-002	-	S7N:L12C	G8D	D69Y	G40T:Q49E: L52W:I101V	yes	-	-	-
Ctag:S7N:L12N:[D69Y]:SOL	G2-003	CI078932	S7N:L12N	-	D69Y	G40T:Q49E: L52W:I101V	yes	30.90	48	97
Ctag:S7N:L12C:(Q14D):[D69 Y]:SOL	G2-004	-	S7N:L12C:Q14D	-	D69Y	G40T:Q49E: L52W:I101V	yes	-	-	-
Ctag:S7N:L12C:(Q14K):[D69 Y]:SOL	G2-005	-	S7N:L12C:Q14K		D69Y	G40T:Q49E: L52W:I101V	yes	-	-	-
Ctag:S7N:L12C:(G16T):[D69 Y]:SOL	G2-006	-	S7N:L12C	G16T	D69Y	G40T:Q49E: L52W:I101V	yes	-	-	-
Ctag:S7N:L12C:(G17E):[D69 Y]:SOL	G2-007	-	S7N:L12C	G17E	D69Y	G40T:Q49E: L52W:I101V	yes	-	-	-
Ctag:S7N:L12C:(R20K):[D69 Y]:SOL	G2-008	-	S7N:L12C	R20K	D69Y	G40T:Q49E: L52W:I101V	yes	-	-	-
Ctag:S7N:L12C:(Q90R):[D69 Y]:SOL	G2-009	CI079712	S7N:L12C	Q90R	D69Y	G40T:Q49E: L52W:I101V	yes	12.56	34	80
Ctag:S7N:L12C:(Q90Y):[D69 Y]:SOL	G2-010	CI079711	S7N:L12C	Q90Y	D69Y	G40T:Q49E: L52W:I101V	yes	12.50	17	48
Ctag:S7N:L12C:(Q90H):[D69 Y]:SOL	G2-011	CI079710	S7N:L12C	Q90H	D69Y	G40T:Q49E: L52W:I101V	yes	9.21	19	48
Ctag:S7N:L12C:(N92D):[D69 Y]:SOL	G2-012	-	S7N:L12C	N92D	D69Y	G40T:Q49E: L52W:I101V	yes	-	-	-
Ctag:S7N:L12C:[D69Y]:SOL	G2-013	CI078936	S7N:L12C	-	D69Y	G40T:Q49E: L52W:I101V	yes	12.60	32	72
Ntag:S7N:L12C:T125A:(G16 T):[N82S:N85G]:SOL	G3*-001	-	S7N:L12C:T125A	G16T	N82S:N85 G	G40T:Q49E: L52W:I101V	no	-	-	-
Ntag:S7N:L12C:T125C:(G16 T):[N82S:N85G]:SOL	G3*-002	CI080147	S7N:L12C:T125C	G16T	N82S:N85 G	G40T:Q49E: L52W:I101V	no	21.29	0	1
Ntag:S7N:L12C:T125D:(G16 T):[N82S:N85G]:SOL	G3*-003	CI080146	S7N:L12C:T125D	G16T	N82S:N85 G	G40T:Q49E: L52W:I101V	no	4.48	0	0
Ntag:S7N:L12C:T125E:(G16 T):[N82S:N85G]:SOL	G3*-004	CI080145	S7N:L12C:T125E	G16T	N82S:N85 G	G40T:Q49E: L52W:I101V	no	13.20	0	0
Ntag:S7N:L12C:T125F:(G16 T):[N82S:N85G]:SOL	G3*-005	-	S7N:L12C:T125F	G16T	N82S:N85 G	G40T:Q49E: L52W:I101V	no	-	-	-
Ntag:S7N:L12C:T125G:(G16 T):[N82S:N85G]:SOL	G3*-006	-	S7N:L12C:T125G	G16T	N82S:N85 G	G40T:Q49E: L52W:I101V	no	-	-	-
Ntag:S7N:L12C:T125H:(G16 T):[N82S:N85G]:SOL	G3*-007	-	S7N:L12C:T125H	G16T	N82S:N85 G	G40T:Q49E: L52W:I101V	no	-	-	-
Ntag:S7N:L12C:T125I:(G16T):[N82S:N85G]:SOL	G3*-008	-	S7N:L12C:T125I	G16T	N82S:N85 G	G40T:Q49E: L52W:I101V	no	-	-	-
Ntag:S7N:L12C:T125K:(G16 T):[N82S:N85G]:SOL	G3*-009	-	S7N:L12C:T125K	G16T	N82S:N85 G	G40T:Q49E: L52W:I101V	no	-	-	-
Ntag:S7N:L12C:T125L:(G16 T):[N82S:N85G]:SOL	G3*-010	-	S7N:L12C:T125L	G16T	N82S:N85 G	G40T:Q49E: L52W:I101V	no	-	-	-
Ntag:S7N:L12C:T125M:(G16 T):[N82S:N85G]:SOL	G3*-011=G4-043	CI080144	S7N:L12C:T125M	G16T	N82S:N85 G	G40T:Q49E: L52W:I101V	no	24.46	34	62

Table S3 Nanobody variant information

Nanobody mutation ID	Nanobody grouped ID	Plate barcode	Key position mutations (S7/L12/Q14/T125)	Other surface mutations around NbInter1	Epitope mutations	Solubility mutations	With C-terminal tail *AENLYFQ?	Concentration (mg/ml)	Drops with mountable crystals	Drop with crystalline species
Ntag:S7N:L12C:T125N:(G16T):[N82S:N85G]:SOL	G3*-012	CI080129	S7N:L12C:T125N	G16T	N82S:N85G	G40T:Q49E:L52W:I101V	no	26.04	10	42
Ntag:S7N:L12C:T125P:(G16T):[N82S:N85G]:SOL	G3*-013	-	S7N:L12C:T125P	G16T	N82S:N85G	G40T:Q49E:L52W:I101V	no	-	-	-
Ntag:S7N:L12C:T125Q:(G16T):[N82S:N85G]:SOL	G3*-014	-	S7N:L12C:T125Q	G16T	N82S:N85G	G40T:Q49E:L52W:I101V	no	-	-	-
Ntag:S7N:L12C:T125R:(G16T):[N82S:N85G]:SOL	G3*-015	CI080128	S7N:L12C:T125R	G16T	N82S:N85G	G40T:Q49E:L52W:I101V	no	24.99	5	19
Ntag:S7N:L12C:T125S:(G16T):[N82S:N85G]:SOL	G3*-016	CI080127	S7N:L12C:T125S	G16T	N82S:N85G	G40T:Q49E:L52W:I101V	no	26.28	3	25
Ntag:S7N:L12C:T125V:(G16T):[N82S:N85G]:SOL	G3*-017	CI080189	S7N:L12C:T125V	G16T	N82S:N85G	G40T:Q49E:L52W:I101V	no	26.17	7	18
Ntag:S7N:L12C:T125W:(G16T):[N82S:N85G]:SOL	G3*-018	CI080188	S7N:L12C:T125W	G16T	N82S:N85G	G40T:Q49E:L52W:I101V	no	27.00	7	26
Ntag:S7N:L12C:T125Y:(G16T):[N82S:N85G]:SOL	G3*-019	-	S7N:L12C:T125Y	G16T	N82S:N85G	G40T:Q49E:L52W:I101V	no	-	-	-
Ntag:S7N:L12N:T125A:[N82S:N85G]:SOL	G3*-020	-	S7N:L12N:T125A	-	N82S:N85G	G40T:Q49E:L52W:I101V	no	-	-	-
Ntag:S7N:L12N:T125C:[N82S:N85G]:SOL	G3*-021	-	S7N:L12N:T125C	-	N82S:N85G	G40T:Q49E:L52W:I101V	no	-	-	-
Ntag:S7N:L12N:T125D:[N82S:N85G]:SOL	G3*-022	-	S7N:L12N:T125D	-	N82S:N85G	G40T:Q49E:L52W:I101V	no	-	-	-
Ntag:S7N:L12N:T125E:[N82S:N85G]:SOL	G3*-023	-	S7N:L12N:T125E	-	N82S:N85G	G40T:Q49E:L52W:I101V	no	-	-	-
Ntag:S7N:L12N:T125F:[N82S:N85G]:SOL	G3*-024	-	S7N:L12N:T125F	-	N82S:N85G	G40T:Q49E:L52W:I101V	no	-	-	-
Ntag:S7N:L12N:T125G:[N82S:N85G]:SOL	G3*-025	-	S7N:L12N:T125G	-	N82S:N85G	G40T:Q49E:L52W:I101V	no	-	-	-
Ntag:S7N:L12N:T125H:[N82S:N85G]:SOL	G3*-026	-	S7N:L12N:T125H	-	N82S:N85G	G40T:Q49E:L52W:I101V	no	-	-	-
Ntag:S7N:L12N:T125I:[N82S:N85G]:SOL	G3*-027	-	S7N:L12N:T125I	-	N82S:N85G	G40T:Q49E:L52W:I101V	no	-	-	-
Ntag:S7N:L12N:T125K:[N82S:N85G]:SOL	G3*-028	-	S7N:L12N:T125K	-	N82S:N85G	G40T:Q49E:L52W:I101V	no	-	-	-
Ntag:S7N:L12N:T125L:[N82S:N85G]:SOL	G3*-029	-	S7N:L12N:T125L	-	N82S:N85G	G40T:Q49E:L52W:I101V	no	-	-	-
Ntag:S7N:L12N:T125M:[N82S:N85G]:SOL	G3*-030=G4-050	-	S7N:L12N:T125M	-	N82S:N85G	G40T:Q49E:L52W:I101V	no	-	-	-
Ntag:S7N:L12N:T125N:[N82S:N85G]:SOL	G3*-031	-	S7N:L12N:T125N	-	N82S:N85G	G40T:Q49E:L52W:I101V	no	-	-	-
Ntag:S7N:L12N:T125P:[N82S:N85G]:SOL	G3*-032	-	S7N:L12N:T125P	-	N82S:N85G	G40T:Q49E:L52W:I101V	no	-	-	-
Ntag:S7N:L12N:T125Q:[N82S:N85G]:SOL	G3*-033	-	S7N:L12N:T125Q	-	N82S:N85G	G40T:Q49E:L52W:I101V	no	-	-	-
Ntag:S7N:L12N:T125R:[N82S:N85G]:SOL	G3*-034	-	S7N:L12N:T125R	-	N82S:N85G	G40T:Q49E:L52W:I101V	no	-	-	-
Ntag:S7N:L12N:T125S:[N82S:N85G]:SOL	G3*-035	-	S7N:L12N:T125S	-	N82S:N85G	G40T:Q49E:L52W:I101V	no	-	-	-
Ntag:S7N:L12N:T125V:[N82S:N85G]:SOL	G3*-036	-	S7N:L12N:T125V	-	N82S:N85G	G40T:Q49E:L52W:I101V	no	-	-	-
Ntag:S7N:L12N:T125W:[N82S:N85G]:SOL	G3*-037	-	S7N:L12N:T125W	-	N82S:N85G	G40T:Q49E:L52W:I101V	no	-	-	-
Ntag:S7N:L12N:T125Y:[N82S:N85G]:SOL	G3*-038	-	S7N:L12N:T125Y	-	N82S:N85G	G40T:Q49E:L52W:I101V	no	-	-	-
Ntag:S7N:L12C:T125A:(Q90Y):[N82S:N85G]:SOL	G3*-039	CI080001	S7N:L12C:T125A	Q90Y	N82S:N85G	G40T:Q49E:L52W:I101V	no	27.56	1	20
Ntag:S7N:L12C:T125C:(Q90Y):[N82S:N85G]:SOL	G3*-040	CI080002	S7N:L12C:T125C	Q90Y	N82S:N85G	G40T:Q49E:L52W:I101V	no	34.83	1	1
Ntag:S7N:L12C:T125D:(Q90Y):[N82S:N85G]:SOL	G3*-041	-	S7N:L12C:T125D	Q90Y	N82S:N85G	G40T:Q49E:L52W:I101V	no	-	-	-
Ntag:S7N:L12C:T125E:(Q90Y):[N82S:N85G]:SOL	G3*-042	CI080003	S7N:L12C:T125E	Q90Y	N82S:N85G	G40T:Q49E:L52W:I101V	no	32.54	0	1
Ntag:S7N:L12C:T125F:(Q90Y):[N82S:N85G]:SOL	G3*-043	-	S7N:L12C:T125F	Q90Y	N82S:N85G	G40T:Q49E:L52W:I101V	no	-	-	-
Ntag:S7N:L12C:T125G:(Q90Y):[N82S:N85G]:SOL	G3*-044	-	S7N:L12C:T125G	Q90Y	N82S:N85G	G40T:Q49E:L52W:I101V	no	-	-	-
Ntag:S7N:L12C:T125H:(Q90Y):[N82S:N85G]:SOL	G3*-045	-	S7N:L12C:T125H	Q90Y	N82S:N85G	G40T:Q49E:L52W:I101V	no	-	-	-
Ntag:S7N:L12C:T125I:(Q90Y):[N82S:N85G]:SOL	G3*-046	-	S7N:L12C:T125I	Q90Y	N82S:N85G	G40T:Q49E:L52W:I101V	no	-	-	-
Ntag:S7N:L12C:T125K:(Q90Y):[N82S:N85G]:SOL	G3*-047	CI080004	S7N:L12C:T125K	Q90Y	N82S:N85G	G40T:Q49E:L52W:I101V	no	33.17	0	0
Ntag:S7N:L12C:T125L:(Q90Y):[N82S:N85G]:SOL	G3*-048	-	S7N:L12C:T125L	Q90Y	N82S:N85G	G40T:Q49E:L52W:I101V	no	-	-	-
Ntag:S7N:L12C:T125M:(Q90Y):[N82S:N85G]:SOL	G3*-049=G4-047	CI080838	S7N:L12C:T125M	Q90Y	N82S:N85G	G40T:Q49E:L52W:I101V	no	30.00	15	26
Ntag:S7N:L12C:T125M:(Q90Y):[N82S:N85G]:SOL	G3*-049=G4-047	CI080420	S7N:L12C:T125M	Q90Y	N82S:N85G	G40T:Q49E:L52W:I101V	no	22.40	15	23
Ntag:S7N:L12C:T125N:(Q90Y):[N82S:N85G]:SOL	G3*-050	CI080817	S7N:L12C:T125N	Q90Y	N82S:N85G	G40T:Q49E:L52W:I101V	no	35.73	12	40
Ntag:S7N:L12C:T125P:(Q90Y):[N82S:N85G]:SOL	G3*-051	-	S7N:L12C:T125P	Q90Y	N82S:N85G	G40T:Q49E:L52W:I101V	no	-	-	-
Ntag:S7N:L12C:T125Q:(Q90Y):[N82S:N85G]:SOL	G3*-052	CI080006	S7N:L12C:T125Q	Q90Y	N82S:N85G	G40T:Q49E:L52W:I101V	no	33.98	12	33
Ntag:S7N:L12C:T125R:(Q90Y):[N82S:N85G]:SOL	G3*-053	CI080028	S7N:L12C:T125R	Q90Y	N82S:N85G	G40T:Q49E:L52W:I101V	no	30.29	1	9
Ntag:S7N:L12C:T125S:(Q90Y):[N82S:N85G]:SOL	G3*-054	-	S7N:L12C:T125S	Q90Y	N82S:N85G	G40T:Q49E:L52W:I101V	no	-	-	-

Table S3 Nanobody variant information

Nanobody mutation ID	Nanobody grouped ID	Plate barcode	Key position mutations (S7/L12/Q14/T125)	Other surface mutations around NbInter1	Epitope mutations	Solubility mutations	With C-terminal tail *AENLYFQ?	Concentration (mg/ml)	Drops with mountable crystals	Drop with crystalline species
Ntag:S7N:L12C:T125V:(Q90Y):[N82S:N85G]:SOL	G3*-055	CI080029	S7N:L12C:T125V	Q90Y	N82S:N85G	G40T:Q49E:L52W:I101V	no	32.18	12	26
Ntag:S7N:L12C:T125W:(Q90Y):[N82S:N85G]:SOL	G3*-056	CI080027	S7N:L12C:T125W	Q90Y	N82S:N85G	G40T:Q49E:L52W:I101V	no	33.17	4	27
Ntag:S7N:L12C:T125Y:(Q90Y):[N82S:N85G]:SOL	G3*-057	-	S7N:L12C:T125Y	Q90Y	N82S:N85G	G40T:Q49E:L52W:I101V	no	-	-	-
Ntag:S7N:L12A:T125M:(G16T):[N82S:N85G]:SOL	G3*-058	-	S7N:L12A:T125M	G16T	N82S:N85G	G40T:Q49E:L52W:I101V	no	-	-	-
Ntag:S7N:L12D:T125M:(G16T):[N82S:N85G]:SOL	G3*-059	-	S7N:L12D:T125M	G16T	N82S:N85G	G40T:Q49E:L52W:I101V	no	-	-	-
Ntag:S7N:L12E:T125M:(G16T):[N82S:N85G]:SOL	G3*-060	CI080422	S7N:L12E:T125M	G16T	N82S:N85G	G40T:Q49E:L52W:I101V	no	27.45	1	1
Ntag:S7N:L12F:T125M:(G16T):[N82S:N85G]:SOL	G3*-061	-	S7N:L12F:T125M	G16T	N82S:N85G	G40T:Q49E:L52W:I101V	no	-	-	-
Ntag:S7N:L12G:T125M:(G16T):[N82S:N85G]:SOL	G3*-062	-	S7N:L12G:T125M	G16T	N82S:N85G	G40T:Q49E:L52W:I101V	no	-	-	-
Ntag:S7N:L12H:T125M:(G16T):[N82S:N85G]:SOL	G3*-063	-	S7N:L12H:T125M	G16T	N82S:N85G	G40T:Q49E:L52W:I101V	no	-	-	-
Ntag:S7N:L12I:T125M:(G16T):[N82S:N85G]:SOL	G3*-064	-	S7N:L12I:T125M	G16T	N82S:N85G	G40T:Q49E:L52W:I101V	no	-	-	-
Ntag:S7N:L12K:T125M:(G16T):[N82S:N85G]:SOL	G3*-065	-	S7N:L12K:T125M	G16T	N82S:N85G	G40T:Q49E:L52W:I101V	no	-	-	-
Ntag:S7N:T125M:(G16T):[N82S:N85G]:SOL	G3*-066	-	S7N:T125M	G16T	N82S:N85G	G40T:Q49E:L52W:I101V	no	-	-	-
Ntag:S7N:L12M:T125M:(G16T):[N82S:N85G]:SOL	G3*-067	-	S7N:L12M:T125M	G16T	N82S:N85G	G40T:Q49E:L52W:I101V	no	-	-	-
Ntag:S7N:L12N:T125M:(G16T):[N82S:N85G]:SOL	G3*-068	-	S7N:L12N:T125M	G16T	N82S:N85G	G40T:Q49E:L52W:I101V	no	-	-	-
Ntag:S7N:L12P:T125M:(G16T):[N82S:N85G]:SOL	G3*-069	CI080423	S7N:L12P:T125M	G16T	N82S:N85G	G40T:Q49E:L52W:I101V	no	27.00	0	0
Ntag:S7N:L12Q:T125M:(G16T):[N82S:N85G]:SOL	G3*-070	-	S7N:L12Q:T125M	G16T	N82S:N85G	G40T:Q49E:L52W:I101V	no	-	-	-
Ntag:S7N:L12R:T125M:(G16T):[N82S:N85G]:SOL	G3*-071	-	S7N:L12R:T125M	G16T	N82S:N85G	G40T:Q49E:L52W:I101V	no	-	-	-
Ntag:S7N:L12S:T125M:(G16T):[N82S:N85G]:SOL	G3*-072	CI080425	S7N:L12S:T125M	G16T	N82S:N85G	G40T:Q49E:L52W:I101V	no	32.85	0	0
Ntag:S7N:L12V:T125M:(G16T):[N82S:N85G]:SOL	G3*-073	-	S7N:L12V:T125M	G16T	N82S:N85G	G40T:Q49E:L52W:I101V	no	-	-	-
Ntag:S7N:L12W:T125M:(G16T):[N82S:N85G]:SOL	G3*-074	CI080424	S7N:L12W:T125M	G16T	N82S:N85G	G40T:Q49E:L52W:I101V	no	36.90	0	0
Ntag:S7N:L12Y:T125M:(G16T):[N82S:N85G]:SOL	G3*-075	-	S7N:L12Y:T125M	G16T	N82S:N85G	G40T:Q49E:L52W:I101V	no	-	-	-
Ntag:S7N:L12T:T125M:(G16T):[N82S:N85G]:SOL	G3*-076	-	S7N:L12T:T125M	G16T	N82S:N85G	G40T:Q49E:L52W:I101V	no	-	-	-
Ntag:S7N:L12A:T125N:(Q90Y):[N82S:N85G]:SOL	G3*-077	-	S7N:L12A:T125N	Q90Y	N82S:N85G	G40T:Q49E:L52W:I101V	no	-	-	-
Ntag:S7N:L12D:T125N:(Q90Y):[N82S:N85G]:SOL	G3*-078	-	S7N:L12D:T125N	Q90Y	N82S:N85G	G40T:Q49E:L52W:I101V	no	-	-	-
Ntag:S7N:L12E:T125N:(Q90Y):[N82S:N85G]:SOL	G3*-079	-	S7N:L12E:T125N	Q90Y	N82S:N85G	G40T:Q49E:L52W:I101V	no	-	-	-
Ntag:S7N:L12F:T125N:(Q90Y):[N82S:N85G]:SOL	G3*-080	-	S7N:L12F:T125N	Q90Y	N82S:N85G	G40T:Q49E:L52W:I101V	no	-	-	-
Ntag:S7N:L12G:T125N:(Q90Y):[N82S:N85G]:SOL	G3*-081	-	S7N:L12G:T125N	Q90Y	N82S:N85G	G40T:Q49E:L52W:I101V	no	-	-	-
Ntag:S7N:L12H:T125N:(Q90Y):[N82S:N85G]:SOL	G3*-082	CI080426	S7N:L12H:T125N	Q90Y	N82S:N85G	G40T:Q49E:L52W:I101V	no	24.30	1	1
Ntag:S7N:L12I:T125N:(Q90Y):[N82S:N85G]:SOL	G3*-083	-	S7N:L12I:T125N	Q90Y	N82S:N85G	G40T:Q49E:L52W:I101V	no	-	-	-
Ntag:S7N:L12K:T125N:(Q90Y):[N82S:N85G]:SOL	G3*-084	-	S7N:L12K:T125N	Q90Y	N82S:N85G	G40T:Q49E:L52W:I101V	no	-	-	-
Ntag:S7N:T125N:(Q90Y):[N82S:N85G]:SOL	G3*-085	-	S7N:T125N	Q90Y	N82S:N85G	G40T:Q49E:L52W:I101V	no	-	-	-
Ntag:S7N:L12M:T125N:(Q90Y):[N82S:N85G]:SOL	G3*-086	CI080427	S7N:L12M:T125N	Q90Y	N82S:N85G	G40T:Q49E:L52W:I101V	no	32.85	0	0
Ntag:S7N:L12N:T125N:(Q90Y):[N82S:N85G]:SOL	G3*-087	-	S7N:L12N:T125N	Q90Y	N82S:N85G	G40T:Q49E:L52W:I101V	no	-	-	-
Ntag:S7N:L12P:T125N:(Q90Y):[N82S:N85G]:SOL	G3*-088	-	S7N:L12P:T125N	Q90Y	N82S:N85G	G40T:Q49E:L52W:I101V	no	-	-	-
Ntag:S7N:L12Q:T125N:(Q90Y):[N82S:N85G]:SOL	G3*-089	-	S7N:L12Q:T125N	Q90Y	N82S:N85G	G40T:Q49E:L52W:I101V	no	-	-	-
Ntag:S7N:L12R:T125N:(Q90Y):[N82S:N85G]:SOL	G3*-090	-	S7N:L12R:T125N	Q90Y	N82S:N85G	G40T:Q49E:L52W:I101V	no	-	-	-
Ntag:S7N:L12S:T125N:(Q90Y):[N82S:N85G]:SOL	G3*-091	-	S7N:L12S:T125N	Q90Y	N82S:N85G	G40T:Q49E:L52W:I101V	no	-	-	-
Ntag:S7N:L12V:T125N:(Q90Y):[N82S:N85G]:SOL	G3*-092	CI080428	S7N:L12V:T125N	Q90Y	N82S:N85G	G40T:Q49E:L52W:I101V	no	31.05	0	0
Ntag:S7N:L12W:T125N:(Q90Y):[N82S:N85G]:SOL	G3*-093	CI080429	S7N:L12W:T125N	Q90Y	N82S:N85G	G40T:Q49E:L52W:I101V	no	27.90	0	0
Ntag:S7N:L12Y:T125N:(Q90Y):[N82S:N85G]:SOL	G3*-094	-	S7N:L12Y:T125N	Q90Y	N82S:N85G	G40T:Q49E:L52W:I101V	no	-	-	-
Ntag:S7N:L12T:T125N:(Q90Y):[N82S:N85G]:SOL	G3*-095	-	S7N:L12T:T125N	Q90Y	N82S:N85G	G40T:Q49E:L52W:I101V	no	-	-	-
Ntag:S7N:L12C:T125M:(G8D):[E6Q]:SOL	G4-001	-	S7N:L12C:T125M	G8D	E6Q	G40T:Q49E:L52W:I101V	no	-	-	-
Ntag:S7N:L12C:Q14D:T125M:[E6Q]:SOL	G4-002	CI080550	S7N:L12C:Q14D:T125M	-	E6Q	G40T:Q49E:L52W:I101V	no	18.81	0	57
Ntag:S7N:L12C:Q14K:T125M:[E6Q]:SOL	G5-001	CI080551	S7N:L12C:Q14K:T125M	-	E6Q	G40T:Q49E:L52W:I101V	no	22.89	6	20

Table S3 Nanobody variant information

Nanobody mutation ID	Nanobody grouped ID	Plate barcode	Key position mutations (S7/L12/Q14/T125)	Other surface mutations around NbInter1	Epitope mutations	Solubility mutations	With C-terminal tail *AENLYFQ?	Concentration (mg/ml)	Drops with mountable crystals	Drop with crystalline species
Ntag:S7N:L12C:T125M:(G16T):[E6Q]:SOL	G4-003	-	S7N:L12C:T125M	G16T	E6Q	G40T:Q49E:L52W:I101V	no	-	-	-
Ntag:S7N:L12C:T125M:(G17E):[E6Q]:SOL	G4-004	-	S7N:L12C:T125M	G17E	E6Q	G40T:Q49E:L52W:I101V	no	-	-	-
Ntag:S7N:L12C:T125M:(R20K):[E6Q]:SOL	G4-005	-	S7N:L12C:T125M	R20K	E6Q	G40T:Q49E:L52W:I101V	no	-	-	-
Ntag:S7N:L12C:T125M:(Q90R):[E6Q]:SOL	G4-006	CI080552	S7N:L12C:T125M	Q90R	E6Q	G40T:Q49E:L52W:I101V	no	22.08	0	35
Ntag:S7N:L12C:T125M:(Q90Y):[E6Q]:SOL	G4-007	-	S7N:L12C:T125M	Q90Y	E6Q	G40T:Q49E:L52W:I101V	no	-	-	-
Ntag:S7N:L12C:T125M:(Q90	G4-008	CI080553	S7N:L12C:T125M	Q90H	E6Q	G40T:Q49E:	no	23.66	1	63
Ntag:S7N:L12C:T125M:(N92D):[E6Q]:SOL	G4-009	-	S7N:L12C:T125M	N92D	E6Q	G40T:Q49E:L52W:I101V	no	-	-	-
Ntag:S7N:L12N:T125M:[E6Q]:SOL	G4-010	-	S7N:L12N:T125M	-	E6Q	G40T:Q49E:L52W:I101V	no	-	-	-
Ntag:S7N:L12C:T125M:(G8D):[T86A:V87L:Y88S]:SOL	G4-011	-	S7N:L12C:T125M	G8D	T86A:V87L:Y88S	G40T:Q49E:L52W:I101V	no	-	-	-
Ntag:S7N:L12C:T125M:(Q14D):[T86A:V87L:Y88S]:SOL	G4-012	-	S7N:L12C:Q14D:T125M	-	T86A:V87L:Y88S	G40T:Q49E:L52W:I101V	no	-	-	-
Ntag:S7N:L12C:T125M:(Q14K):[T86A:V87L:Y88S]:SOL	G5-002	-	S7N:L12C:Q14K:T125M	-	T86A:V87L:Y88S	G40T:Q49E:L52W:I101V	no	-	-	-
Ntag:S7N:L12C:T125M:(G16T):[T86A:V87L:Y88S]:SOL	G4-013	-	S7N:L12C:T125M	G16T	T86A:V87L:Y88S	G40T:Q49E:L52W:I101V	no	-	-	-
Ntag:S7N:L12C:T125M:(G17E):[T86A:V87L:Y88S]:SOL	G4-014	-	S7N:L12C:T125M	G17E	T86A:V87L:Y88S	G40T:Q49E:L52W:I101V	no	-	-	-
Ntag:S7N:L12C:T125M:(R20K):[T86A:V87L:Y88S]:SOL	G4-015	-	S7N:L12C:T125M	R20K	T86A:V87L:Y88S	G40T:Q49E:L52W:I101V	no	-	-	-
Ntag:S7N:L12C:T125M:(Q90R):[T86A:V87L:Y88S]:SOL	G4-016	-	S7N:L12C:T125M	Q90R	T86A:V87L:Y88S	G40T:Q49E:L52W:I101V	no	-	-	-
Ntag:S7N:L12C:T125M:(Q90Y):[T86A:V87L:Y88S]:SOL	G4-017	-	S7N:L12C:T125M	Q90Y	T86A:V87L:Y88S	G40T:Q49E:L52W:I101V	no	-	-	-
Ntag:S7N:L12C:T125M:(Q90H):[T86A:V87L:Y88S]:SOL	G4-018	-	S7N:L12C:T125M	Q90H	T86A:V87L:Y88S	G40T:Q49E:L52W:I101V	no	-	-	-
Ntag:S7N:L12C:T125M:(N92D):[T86A:V87L:Y88S]:SOL	G4-019	-	S7N:L12C:T125M	N92D	T86A:V87L:Y88S	G40T:Q49E:L52W:I101V	no	-	-	-
Ntag:S7N:L12N:T125M:[T86A:V87L:Y88S]:SOL	G4-020	-	S7N:L12N:T125M	-	T86A:V87L:Y88S	G40T:Q49E:L52W:I101V	no	-	-	-
Ntag:S7N:L12C:T125M:(G8D	G4-021	CI080554	S7N:L12C:T125M	G8D	Y88H	G40T:Q49E:	no	22.31	0	0
Ntag:S7N:L12C:Q14D:T125M:[Y88H]:SOL	G4-022	CI080555	S7N:L12C:Q14D:T125M	-	Y88H	G40T:Q49E:L52W:I101V	no	19.16	9	29
Ntag:S7N:L12C:Q14K:T125M:[Y88H]:SOL	G5-003	CI080556	S7N:L12C:Q14K:T125M	-	Y88H	G40T:Q49E:L52W:I101V	no	18.19	15	20
Ntag:S7N:L12C:T125M:(G16T):[Y88H]:SOL	G4-023	-	S7N:L12C:T125M	G16T	Y88H	G40T:Q49E:L52W:I101V	no	-	-	-
Ntag:S7N:L12C:T125M:(G17E):[Y88H]:SOL	G4-024	-	S7N:L12C:T125M	G17E	Y88H	G40T:Q49E:L52W:I101V	no	-	-	-
Ntag:S7N:L12C:T125M:(R20K):[Y88H]:SOL	G4-025	CI080557	S7N:L12C:T125M	R20K	Y88H	G40T:Q49E:L52W:I101V	no	22.02	5	20
Ntag:S7N:L12C:T125M:(Q90R):[Y88H]:SOL	G4-026	CI080558	S7N:L12C:T125M	Q90R	Y88H	G40T:Q49E:L52W:I101V	no	10.07	0	2
Ntag:S7N:L12C:T125M:(Q90Y):[Y88H]:SOL	G4-027	CI080559	S7N:L12C:T125M	Q90Y	Y88H	G40T:Q49E:L52W:I101V	no	19.88	4	21
Ntag:S7N:L12C:T125M:(Q90H):[Y88H]:SOL	G4-028	-	S7N:L12C:T125M	Q90H	Y88H	G40T:Q49E:L52W:I101V	no	-	-	-
Ntag:S7N:L12C:T125M:(N92D):[Y88H]:SOL	G4-029	CI080536	S7N:L12C:T125M	N92D	Y88H	G40T:Q49E:L52W:I101V	no	20.12	9	52
Ntag:S7N:L12N:T125M:[Y88H]:SOL	G4-030	-	S7N:L12N:T125M	-	Y88H	G40T:Q49E:L52W:I101V	no	-	-	-
Ntag:S7N:L12C:T125M:(G8D):[A54F:A55T]:SOL	G4-031	-	S7N:L12C:T125M	G8D	A54F:A55T	G40T:Q49E:L52W:I101V	no	-	-	-
Ntag:S7N:L12C:T125M:(Q14D):[A54F:A55T]:SOL	G4-032	-	S7N:L12C:Q14D:T125M	-	A54F:A55T	G40T:Q49E:L52W:I101V	no	-	-	-
Ntag:S7N:L12C:T125M:(Q14K):[A54F:A55T]:SOL	G5-004	-	S7N:L12C:Q14K:T125M	-	A54F:A55T	G40T:Q49E:L52W:I101V	no	-	-	-
Ntag:S7N:L12C:T125M:(G16T):[A54F:A55T]:SOL	G4-033	-	S7N:L12C:T125M	G16T	A54F:A55T	G40T:Q49E:L52W:I101V	no	-	-	-
Ntag:S7N:L12C:T125M:(G17E):[A54F:A55T]:SOL	G4-034	-	S7N:L12C:T125M	G17E	A54F:A55T	G40T:Q49E:L52W:I101V	no	-	-	-
Ntag:S7N:L12C:T125M:(R20K):[A54F:A55T]:SOL	G4-035	-	S7N:L12C:T125M	R20K	A54F:A55T	G40T:Q49E:L52W:I101V	no	-	-	-
Ntag:S7N:L12C:T125M:(Q90R):[A54F:A55T]:SOL	G4-036	-	S7N:L12C:T125M	Q90R	A54F:A55T	G40T:Q49E:L52W:I101V	no	-	-	-
Ntag:S7N:L12C:T125M:(Q90Y):[A54F:A55T]:SOL	G4-037	-	S7N:L12C:T125M	Q90Y	A54F:A55T	G40T:Q49E:L52W:I101V	no	-	-	-
Ntag:S7N:L12C:T125M:(Q90H):[A54F:A55T]:SOL	G4-038	-	S7N:L12C:T125M	Q90H	A54F:A55T	G40T:Q49E:L52W:I101V	no	-	-	-
Ntag:S7N:L12C:T125M:(N92D):[A54F:A55T]:SOL	G4-039	-	S7N:L12C:T125M	N92D	A54F:A55T	G40T:Q49E:L52W:I101V	no	-	-	-
Ntag:S7N:L12N:T125M:[A54F:A55T]:SOL	G4-040	-	S7N:L12N:T125M	-	A54F:A55T	G40T:Q49E:L52W:I101V	no	-	-	-
Ntag:S7N:L12C:T125M:(G8D):[N82S:N85G]:SOL	G4-041	CI080537	S7N:L12C:T125M	G8D	N82S:N85G	G40T:Q49E:L52W:I101V	no	20.40	1	1
Ntag:S7N:L12C:T125M:(Q14D):[N82S:N85G]:SOL	G4-042	-	S7N:L12C:Q14D:T125M	-	N82S:N85G	G40T:Q49E:L52W:I101V	no	-	-	-
Ntag:S7N:L12C:Q14K:T125M:[N82S:N85G]:SOL	G5-005	CI080560	S7N:L12C:Q14K:T125M	-	N82S:N85G	G40T:Q49E:L52W:I101V	no	14.05	1	1
Ntag:S7N:L12C:T125M:(G16T):[N82S:N85G]:SOL	G4-043	-	S7N:L12C:T125M	G16T	N82S:N85G	G40T:Q49E:L52W:I101V	no	-	-	-
Ntag:S7N:L12C:T125M:(G17E):[N82S:N85G]:SOL	G4-044	CI080561	S7N:L12C:T125M	G17E	N82S:N85G	G40T:Q49E:L52W:I101V	no	22.15	0	0

Table S3 Nanobody variant information

Nanobody mutation ID	Nanobody grouped ID	Plate barcode	Key position mutations (S7/L12/Q14/T125)	Other surface mutations around NbInter1	Epitope mutations	Solubility mutations	With C-terminal tail *AENLYFQ?	Concentration (mg/ml)	Drops with mountable crystals	Drop with crystalline species
Ntag:S7N:L12C:T125M:(R20K):[N82S:N85G]:SOL	G4-045	-	S7N:L12C:T125M	R20K	N82S:N85G	G40T:Q49E:L52W:I101V	no	-	-	-
Ntag:S7N:L12C:T125M:(Q90R):[N82S:N85G]:SOL	G4-046	CI080562	S7N:L12C:T125M	Q90R	N82S:N85G	G40T:Q49E:L52W:I101V	no	7.75	3	8
Ntag:S7N:L12C:T125M:(Q90Y):[N82S:N85G]:SOL	G4-047	CI080563	S7N:L12C:T125M	Q90Y	N82S:N85G	G40T:Q49E:L52W:I101V	no	22.28	5	39
Ntag:S7N:L12C:T125M:(Q90H):[N82S:N85G]:SOL	G4-048	CI080564	S7N:L12C:T125M	Q90H	N82S:N85G	G40T:Q49E:L52W:I101V	no	20.42	3	10
Ntag:S7N:L12C:T125M:(N92D):[N82S:N85G]:SOL	G4-049	CI080607	S7N:L12C:T125M	N92D	N82S:N85G	G40T:Q49E:L52W:I101V	no	20.61	0	0
Ntag:S7N:L12N:T125M:[N82S:N85G]:SOL	G4-050	CI080565	S7N:L12N:T125M	-	N82S:N85G	G40T:Q49E:L52W:I101V	no	22.01	3	14
Ntag:S7N:L12C:T125M:(G8D):[K84E]:SOL	G4-051	-	S7N:L12C:T125M	G8D	K84E	G40T:Q49E:L52W:I101V	no	-	-	-
Ntag:S7N:L12C:T125M:(Q14D):[K84E]:SOL	G4-052	-	S7N:L12C:Q14D:T125M	-	K84E	G40T:Q49E:L52W:I101V	no	-	-	-
Ntag:S7N:L12C:T125M:(Q14K):[K84E]:SOL	G5-006	CI080566	S7N:L12C:Q14K:T125M	-	K84E	G40T:Q49E:L52W:I101V	no	20.04	15	22
Ntag:S7N:L12C:T125M:(G16T):[K84E]:SOL	G4-053	-	S7N:L12C:T125M	G16T	K84E	G40T:Q49E:L52W:I101V	no	-	-	-
Ntag:S7N:L12C:T125M:(G17E):[K84E]:SOL	G4-054	CI080567	S7N:L12C:T125M	G17E	K84E	G40T:Q49E:L52W:I101V	no	20.61	0	0
Ntag:S7N:L12C:T125M:(R20K):[K84E]:SOL	G4-055	CI080568	S7N:L12C:T125M	R20K	K84E	G40T:Q49E:L52W:I101V	no	20.65	0	0
Ntag:S7N:L12C:T125M:(Q90R):[K84E]:SOL	G4-056	-	S7N:L12C:T125M	Q90R	K84E	G40T:Q49E:L52W:I101V	no	-	-	-
Ntag:S7N:L12C:T125M:(Q90Y):[K84E]:SOL	G4-057	CI080569	S7N:L12C:T125M	Q90Y	K84E	G40T:Q49E:L52W:I101V	no	20.44	4	41
Ntag:S7N:L12C:T125M:(Q90H):[K84E]:SOL	G4-058	-	S7N:L12C:T125M	Q90H	K84E	G40T:Q49E:L52W:I101V	no	-	-	-
Ntag:S7N:L12C:T125M:(N92D):[K84E]:SOL	G4-059	-	S7N:L12C:T125M	N92D	K84E	G40T:Q49E:L52W:I101V	no	-	-	-
Ntag:S7N:L12N:T125M:[K84E]:SOL	G4-060	-	S7N:L12N:T125M	-	K84E	G40T:Q49E:L52W:I101V	no	-	-	-
Ntag:S7N:L12C:T125M:(G8D):[S79Q]:SOL	G4-061	CI080534	S7N:L12C:T125M	G8D	S79Q	G40T:Q49E:L52W:I101V	no	21.68	0	0
Ntag:S7N:L12C:Q14D:T125M:[S79Q]:SOL	G4-062	CI080608	S7N:L12C:Q14D:T125M	-	S79Q	G40T:Q49E:L52W:I101V	no	20.53	0	0
Ntag:S7N:L12C:Q14K:T125M:[S79Q:A24V]:SOL	G5-007	CI080535	S7N:L12C:Q14K:T125M	-	S79Q:A24V	G40T:Q49E:L52W:I101V	no	21.61	15	35
Ntag:S7N:L12C:T125M:(G16T):[S79Q]:SOL	G4-063	CI080520	S7N:L12C:T125M	G16T	S79Q	G40T:Q49E:L52W:I101V	no	5.83	1	3
Ntag:S7N:L12C:T125M:(G17E):[S79Q]:SOL	G4-064	CI080609	S7N:L12C:T125M	G17E	S79Q	G40T:Q49E:L52W:I101V	no	37.40	0	0
Ntag:S7N:L12C:T125M:(R20K):[S79Q]:SOL	G4-065	CI080521	S7N:L12C:T125M	R20K	S79Q	G40T:Q49E:L52W:I101V	no	21.30	6	46
Ntag:S7N:L12C:T125M:(Q90R):[S79Q]:SOL	G4-066	-	S7N:L12C:T125M	Q90R	S79Q	G40T:Q49E:L52W:I101V	no	-	-	-
Ntag:S7N:L12C:T125M:(Q90Y):[S79Q]:SOL	G4-067	CI080522	S7N:L12C:T125M	Q90Y	S79Q	G40T:Q49E:L52W:I101V	no	18.97	7	45
Ntag:S7N:L12C:T125M:(Q90H):[S79Q]:SOL	G4-068	-	S7N:L12C:T125M	Q90H	S79Q	G40T:Q49E:L52W:I101V	no	-	-	-
Ntag:S7N:L12C:T125M:(N92D):[S79Q]:SOL	G4-069	CI080523	S7N:L12C:T125M	N92D	S79Q	G40T:Q49E:L52W:I101V	no	22.49	3	66
Ntag:S7N:L12C:T125M:(G8D):[R20K:L21V]:SOL	G4-070	-	S7N:L12C:T125M	G8D	R20K:L21V	G40T:Q49E:L52W:I101V	no	-	-	-
Ntag:S7N:L12C:T125M:(Q14D):[R20K:L21V]:SOL	G4-071	-	S7N:L12C:Q14D:T125M	-	R20K:L21V	G40T:Q49E:L52W:I101V	no	-	-	-
Ntag:S7N:L12C:T125M:(Q14K):[R20K:L21V]:SOL	G5-008	-	S7N:L12C:Q14K:T125M	-	R20K:L21V	G40T:Q49E:L52W:I101V	no	-	-	-
Ntag:S7N:L12C:T125M:(G16T):[R20K:L21V]:SOL	G4-072	-	S7N:L12C:T125M	G16T	R20K:L21V	G40T:Q49E:L52W:I101V	no	-	-	-
Ntag:S7N:L12C:T125M:(G17E):[R20K:L21V]:SOL	G4-073	-	S7N:L12C:T125M	G17E	R20K:L21V	G40T:Q49E:L52W:I101V	no	-	-	-
Ntag:S7N:L12C:T125M:(R20K):[R20K:L21V]:SOL	G4-074	-	S7N:L12C:T125M	R20K	R20K:L21V	G40T:Q49E:L52W:I101V	no	-	-	-
Ntag:S7N:L12C:T125M:(Q90R):[R20K:L21V]:SOL	G4-075	-	S7N:L12C:T125M	Q90R	R20K:L21V	G40T:Q49E:L52W:I101V	no	-	-	-
Ntag:S7N:L12C:T125M:(Q90Y):[R20K:L21V]:SOL	G4-076	-	S7N:L12C:T125M	Q90Y	R20K:L21V	G40T:Q49E:L52W:I101V	no	-	-	-
Ntag:S7N:L12C:T125M:(Q90H):[R20K:L21V]:SOL	G4-077	-	S7N:L12C:T125M	Q90H	R20K:L21V	G40T:Q49E:L52W:I101V	no	-	-	-
Ntag:S7N:L12C:T125M:(N92D):[R20K:L21V]:SOL	G4-078	CI080524	S7N:L12C:T125M	N92D	R20K:L21V	G40T:Q49E:L52W:I101V	no	7.74	0	0
Ntag:S7N:L12C:T125M:(G8D):[Q44R]:SOL	G4-079	-	S7N:L12C:T125M	G8D	Q44R	G40T:Q49E:L52W:I101V	no	-	-	-
Ntag:S7N:L12C:T125M:(Q14D):[Q44R]:SOL	G4-080	-	S7N:L12C:Q14D:T125M	-	Q44R	G40T:Q49E:L52W:I101V	no	-	-	-
Ntag:S7N:L12C:Q14K:T125M:[Q44R]:SOL	G5-009	CI080525	S7N:L12C:Q14K:T125M	-	Q44R	G40T:Q49E:L52W:I101V	no	22.66	28	44
Ntag:S7N:L12C:T125M:(G16T):[Q44R]:SOL	G4-081	-	S7N:L12C:T125M	G16T	Q44R	G40T:Q49E:L52W:I101V	no	-	-	-
Ntag:S7N:L12C:T125M:(G17E):[Q44R]:SOL	G4-082	CI080526	S7N:L12C:T125M	G17E	Q44R	G40T:Q49E:L52W:I101V	no	21.35	0	0
Ntag:S7N:L12C:T125M:(R20K):[Q44R]:SOL	G4-083	-	S7N:L12C:T125M	R20K	Q44R	G40T:Q49E:L52W:I101V	no	-	-	-
Ntag:S7N:L12C:T125M:(Q90R):[Q44R]:SOL	G4-084	-	S7N:L12C:T125M	Q90R	Q44R	G40T:Q49E:L52W:I101V	no	-	-	-

Table S3 Nanobody variant information

Nanobody mutation ID	Nanobody grouped ID	Plate barcode	Key position mutations (S7/L12/Q14/T125)	Other surface mutations around NbInter1	Epitope mutations	Solubility mutations	With C-terminal tail *AENLYFQ?	Concentration (mg/ml)	Drops with mountable crystals	Drop with crystalline species
Ntag:S7N:L12C:T125M:(Q90Y):[Q44R]:SOL	G4-085	CI080527	S7N:L12C:T125M	Q90Y	Q44R	G40T:Q49E:L52W:I101V	no	20.39	2	46
Ntag:S7N:L12C:T125M:(Q90H):[Q44R]:SOL	G4-086	CI080528	S7N:L12C:T125M	Q90H	Q44R	G40T:Q49E:L52W:I101V	no	13.61	5	55
Ntag:S7N:L12C:T125M:(N92D):[Q44R]:SOL	G4-087	CI080529	S7N:L12C:T125M	N92D	Q44R	G40T:Q49E:L52W:I101V	no	19.08	2	41

Table S4 Full list of diffraction data collected from crystals of all nanobody variants tested in the RECQL5:nanobody system. Resolution indicated here uses the criteria CC ½ > 0.3 from the ISPYB auto-processing pipeline without any further data truncation.

Table S4 Diffraction data

Nanobody grouped ID	Mounted Crystal ID	Xtal Plate Protein Frozen/Fresh?	Xtal Plate Protein Concentration (mg/ml)	Xtal Plate Temperature	Xtal Plate Barcode	Screen Batch Name	XTBM Row	XTBM Column	XTBM Subwell	XTBM Condition	Crystal Form Group	Cystal Form Annotation	Unit Cell Dimensions	Space group	Resolution	Number of Successful Autoprocessing Pipelines	Autoprocessing Pipeline Used	Experiment Time	Soaking condition	Xtal Group	Surface Mutations Around Nbinter-1	Key Mutations	Crystal Epitope Mutations	Condition Repitition?
G1-001	XX10RECQL5A-x008	Frozen	11.46	20	CI072193	MYE XX10RECQL5A-c001-z001-01	E	5	a	0.1M bis-tris pH 5.9 -- 0.1M lithium sulfate -- 30% PEG3350	0	Dimer P 1 2 1	75.76 185.71 101.02 (90.0 107.46 90.0)	P 1 21 1	2.76	8	Xia2-DIALS	21/07/2018 20:36	-	PEG	-	-	D69Y	no
G1-001	XX10RECQL5A-x016	Frozen	11.46	20	CI072193	MYE XX10RECQL5A-c001-z001-01	F	5	a	0.1M bis-tris pH 6.2 -- 0.1M lithium sulfate -- 30% PEG3350	0	Dimer P 1 2 1	75.55 184.08 100.6 (90.0 107.68 90.0)	P 1 21 1	3.1	3	Autoproc	21/07/2018 20:28	-	PEG	-	-	D69Y	no
G1-061	XX17RECQL5A-x008	Fresh	12.0038	20	CI066019	HIN3-170302-01	F	6	c	0.2M ammonium sulfate -- 25% PEG3350 -- 0.1M bis-tris pH 5.5	0	Dimer P 1 2 1	76.61 185.71 98.67 (90.0 107.69 90.0)	P 1 21 1	3.28	5	Xia2-DIALS	29/11/2019 13:52	-	PEG	R20K	-	D69Y	no
G1-061	XX17RECQL5A-x017	Fresh	12.0038	20	CI066019	HIN3-170302-01	F	9	d	0.2M ammonium sulfate -- 25% PEG3350 -- 0.1M tris pH 8.5	0	Dimer P 1 2 1	77.11 184.26 98.99 (90.0 108.1 90.0)	P 1 21 1	3.31	5	Xia2-DIALS	29/11/2019 13:59	-	PEG	R20K	-	D69Y	no
G1-061	XX17RECQL5A-x007	Fresh	12.0038	20	CI066019	HIN3-170302-01	F	6	c	0.2M ammonium sulfate -- 25% PEG3350 -- 0.1M bis-tris pH 5.5	0	Dimer P 1 2 1	76.08 183.85 98.74 (90.0 107.42 90.0)	P 1 21 1	3.38	5	Xia2-DIALS	29/11/2019 14:29	-	PEG	R20K	-	D69Y	no
G1-061	XX17RECQL5A-x015	Fresh	12.0038	20	CI066019	HIN3-170302-01	F	8	d	0.2M ammonium sulfate -- 25% PEG3350 -- 0.1M HEPES pH 7.5	0	Dimer P 1 2 1	76.37 182.95 98.97 (90.0 107.76 90.0)	P 1 21 1	3.59	5	Xia2-DIALS	29/11/2019 14:21	-	PEG	R20K	-	D69Y	no
G1-061	XX17RECQL5A-x114	Fresh	13.104	20	CI079082	HIN3-170302-01	D	6	a	25% PEG3350 -- 0.1M bis-tris pH 5.5	0	Dimer P 1 2 1	74.87 182.63 99.98 (90.0 107.16 90.0)	P 1 21 1	3.91	5	Xia2-DIALS	14/12/2019 07:37	-	PEG	R20K	-	D69Y	no
G1-061	XX17RECQL5A-x004	Fresh	12.0038	20	CI066019	HIN3-170302-01	F	6	c	0.2M ammonium sulfate -- 25% PEG3350 -- 0.1M bis-tris pH 5.5	0	Dimer P 1 2 1	75.81 181.71 98.52 (90.0 107.37 90.0)	P 1 21 1	4.75	4	Xia2-3dii	29/11/2019 14:41	-	PEG	R20K	-	D69Y	no
G1-061	XX17RECQL5A-x117	Fresh	13.104	20	CI079082	HIN3-170302-01	G	7	a	0.2M ammonium acetate -- 25% PEG3350 -- 0.1M bis-tris pH 6.5	NA		NA	NA	NA	0	NA	14/12/2019 07:44	-	PEG	R20K	-	D69Y	no
G1-061	XX17RECQL5A-x116	Fresh	13.104	20	CI079082	HIN3-170302-01	G	7	a	0.2M ammonium acetate -- 25% PEG3350 -- 0.1M bis-tris pH 6.5	NA		NA	NA	NA	0	NA	14/12/2019 07:42	-	PEG	R20K	-	D69Y	no
G1-061	XX17RECQL5A-x115	Fresh	13.104	20	CI079082	HIN3-170302-01	F	9	c	0.2M ammonium sulfate -- 25% PEG3350 -- 0.1M tris pH 8.5	NA		NA	NA	NA	0	NA	14/12/2019 07:40	-	PEG	R20K	-	D69Y	no
G1-061	XX17RECQL5A-x012	Fresh	12.0038	20	CI066019	HIN3-170302-01	F	7	d	0.2M ammonium sulfate -- 25% PEG3350 -- 0.1M bis-tris pH 6.5	NA		NA	NA	NA	0	NA	29/11/2019 13:47	-	PEG	R20K	-	D69Y	no
G1-061	XX17RECQL5A-x006	Fresh	12.0038	20	CI066019	HIN3-170302-01	F	6	c	0.2M ammonium sulfate -- 25% PEG3350 -- 0.1M bis-tris pH 5.5	NA		NA	NA	NA	0	NA	29/11/2019 14:31	-	PEG	R20K	-	D69Y	no
G1-061	XX17RECQL5A-x005	Fresh	12.0038	20	CI066019	HIN3-170302-01	F	6	c	0.2M ammonium sulfate -- 25% PEG3350 -- 0.1M bis-tris pH 5.5	NA		NA	NA	NA	0	NA	29/11/2019 14:33	-	PEG	R20K	-	D69Y	no
G1-066	XX17RECQL5A-x021	Fresh	14.1165	20	CI066035	HIN3-170302-01	F	7	d	0.2M ammonium sulfate -- 25% PEG3350 -- 0.1M bis-tris pH 6.5	0	Dimer P 1 2 1	77.39 186.92 99.71 (90.0 108.23 90.0)	P 1 21 1	3.05	5	Xia2-DIALS	29/11/2019 14:02	-	PEG	Q90R	-	D69Y	no

Table S4 Diffraction data

Nanobody grouped ID	Mounted Crystal ID	Xtal Plate Protein Frozen/Fresh?	Xtal Plate Protein Concentration (mg/ml)	Xtal Plate Temperature	Xtal Plate Barcode	Screen Batch Name	XTBM Row	XTBM Column	XTBM Subwell	XTBM Condition	Crystal Form Group	Cystal Form Annotation	Unit Cell Dimensions	Space group	Resolution	Number of Successful Autoprocessing Pipelines	Autoprocessing Pipeline Used	Experiment Time	Soaking condition	Xtal Group	Surface Mutations Around Nbinter-1	Key Mutations	Crystal Epitope Mutations	Condition Repitition?
G1-066	XX17RECQ L5A-x022	Fresh	14.1165	20	CI066035	HIN3-170302-01	F	7	d	0.2M ammonium sulfate -- 25% PEG3350 -- 0.1M bis-tris pH 6.5	0	Dimer P 1 2 1	77.35 186.3 99.39 (90.0 108.19 90.0)	P 1 21 1	3.21	5	Xia2-DIALS	29/11/2019 13:56	-	PEG	Q90R	-	D69Y	no
G1-066	XX17RECQ L5A-x028	Fresh	14.1165	20	CI066035	HIN3-170302-01	G	5	c	0.2M lithium sulfate -- 25% PEG3350 -- 0.1M tris pH 8.5	NA		NA	NA	NA	0	NA	29/11/2019 14:44	-	PEG	Q90R	-	D69Y	no
G1-066	XX17RECQ L5A-x026	Fresh	14.1165	20	CI066035	HIN3-170302-01	G	5	c	0.2M lithium sulfate -- 25% PEG3350 -- 0.1M tris pH 8.5	NA		NA	NA	NA	0	NA	29/11/2019 14:46	-	PEG	Q90R	-	D69Y	no
G1-067	XX17RECQ L5A-x029	Fresh	10.4681	20	CI066034	HIN3-170302-01	F	7	d	0.2M ammonium sulfate -- 25% PEG3350 -- 0.1M bis-tris pH 6.5	0	Dimer P 1 2 1	77.21 187.25 99.4 (90.0 108.06 90.0)	P 1 21 1	3.02	5	Xia2-DIALS	29/11/2019 13:54	-	PEG	Q90Y	-	D69Y	no
G1-067	XX17RECQ L5A-x030	Fresh	10.4681	20	CI066034	HIN3-170302-01	F	7	d	0.2M ammonium sulfate -- 25% PEG3350 -- 0.1M bis-tris pH 6.5	0	Dimer P 1 2 1	76.97 186.84 98.89 (90.0 108.01 90.0)	P 1 21 1	3.13	5	Xia2-DIALS	29/11/2019 13:50	-	PEG	Q90Y	-	D69Y	no
G1-067	XX17RECQ L5A-x031	Fresh	10.4681	20	CI066034	HIN3-170302-01	F	7	d	0.2M ammonium sulfate -- 25% PEG3350 -- 0.1M bis-tris pH 6.5	0	Dimer P 1 2 1	76.86 184.35 98.86 (90.0 107.95 90.0)	P 1 21 1	3.2	4	Xia2-DIALS	29/11/2019 14:18	-	PEG	Q90Y	-	D69Y	no
G1-067	XX17RECQ L5A-x084	Fresh	12.78	20	CI079080	HIN3-170302-01	F	6	c	0.2M ammonium sulfate -- 25% PEG3350 -- 0.1M bis-tris pH 5.5	0	Dimer P 1 2 1	77.29 174.52 93.76 (90.0 107.57 90.0)	P 1 21 1	3.31	3	Xia2-DIALS	14/12/2019 08:26	-	PEG	Q90Y	-	D69Y	no
G1-067	XX17RECQ L5A-x032	Fresh	10.4681	20	CI066034	HIN3-170302-01	F	7	d	0.2M ammonium sulfate -- 25% PEG3350 -- 0.1M bis-tris pH 6.5	0	Dimer P 1 2 1	76.61 184.75 98.79 (90.0 107.94 90.0)	P 1 21 1	3.33	5	Xia2-DIALS	29/11/2019 14:43	-	PEG	Q90Y	-	D69Y	no
G1-067	XX17RECQ L5A-x033	Fresh	10.4681	20	CI066034	HIN3-170302-01	F	7	d	0.2M ammonium sulfate -- 25% PEG3350 -- 0.1M bis-tris pH 6.5	0	Dimer P 1 2 1	76.59 183.42 98.43 (90.0 107.86 90.0)	P 1 21 1	3.86	5	Xia2-DIALS	29/11/2019 14:58	-	PEG	Q90Y	-	D69Y	no
G1-067	XX17RECQ L5A-x037	Fresh	10.4681	20	CI066034	HIN3-170302-01	G	5	c	0.2M lithium sulfate -- 25% PEG3350 -- 0.1M tris pH 8.5	-1	NA	116.16 116.31 175.26 (90.1 89.93 119.93)	P 1	6.45	1	Xia2-DIALS	29/11/2019 14:55	-	PEG	Q90Y	-	D69Y	no
G1-067	XX17RECQ L5A-x085	Fresh	12.78	20	CI079080	HIN3-170302-01	F	7	a	0.2M ammonium sulfate -- 25% PEG3350 -- 0.1M bis-tris pH 6.5	0	Dimer P 1 2 1	76.62 186.37 97.83 (90.0 107.78 90.0)	P 1 2 1	7.18	2	Autoproc	14/12/2019 08:28	-	PEG	Q90Y	-	D69Y	no
G1-067	XX17RECQ L5A-x091	Fresh	12.78	20	CI079080	HIN3-170302-01	G	10	a	0.2M magnesium chloride -- 25% PEG3350 -- 0.1M bis-tris pH 5.5	NA		NA	NA	NA	0	NA	14/12/2019 08:45	-	PEG	Q90Y	-	D69Y	no
G1-067	XX17RECQ L5A-x090	Fresh	12.78	20	CI079080	HIN3-170302-01	G	6	a	0.2M ammonium acetate -- 25% PEG3350 -- 0.1M bis-tris pH 5.5	NA		NA	NA	NA	0	NA	14/12/2019 08:43	-	PEG	Q90Y	-	D69Y	no
G1-067	XX17RECQ L5A-x089	Fresh	12.78	20	CI079080	HIN3-170302-01	G	6	a	0.2M ammonium acetate -- 25% PEG3350 -- 0.1M bis-tris pH 5.5	NA		NA	NA	NA	0	NA	14/12/2019 08:41	-	PEG	Q90Y	-	D69Y	no
G1-067	XX17RECQ L5A-x088	Fresh	12.78	20	CI079080	HIN3-170302-01	G	5	c	0.2M lithium sulfate -- 25% PEG3350 -- 0.1M tris pH 8.5	NA		NA	NA	NA	0	NA	14/12/2019 08:39	-	PEG	Q90Y	-	D69Y	no

Table S4 Diffraction data

Nanobody grouped ID	Mounted Crystal ID	Xtal Plate Protein Frozen/Fresh?	Xtal Plate Protein Concentration (mg/ml)	Xtal Plate Temperature	Xtal Plate Barcode	Screen Batch Name	XTBM Row	XTBM Column	XTBM Subwell	XTBM Condition	Crystal Form Group	Cystal Form Annotation	Unit Cell Dimensions	Space group	Resolution	Number of Successful Autoprocessing Pipelines	Autoprocessing Pipeline Used	Experiment Time	Soaking condition	Xtal Group	Surface Mutations Around Nbinter-1	Key Mutations	Crystal Epitope Mutations	Condition Repitition?
G1-067	XX17RECQ L5A-x087	Fresh	12.78	20	CI079080	HIN3-170302-01	G	3	a	0.2M lithium sulfate -- 25% PEG3350 -- 0.1M bis-tris pH 6.5	NA		NA	NA	NA	0	NA	14/12/2019 08:35	-	PEG	Q90Y	-	D69Y	no
G1-067	XX17RECQ L5A-x086	Fresh	12.78	20	CI079080	HIN3-170302-01	F	9	a	0.2M ammonium sulfate -- 25% PEG3350 -- 0.1M tris pH 8.5	NA		NA	NA	NA	0	NA	14/12/2019 08:32	-	PEG	Q90Y	-	D69Y	no
G1-067	XX17RECQ L5A-x036	Fresh	10.4681	20	CI066034	HIN3-170302-01	G	5	c	0.2M lithium sulfate -- 25% PEG3350 -- 0.1M tris pH 8.5	NA		NA	NA	NA	0	NA	29/11/2019 15:26	-	PEG	Q90Y	-	D69Y	no
G1-068	XX17RECQ L5A-x057	Fresh	14.3505	20	CI066033	HIN3-170302-01	F	12	c	0.2M sodium chloride -- 25% PEG3350 -- 0.1M HEPES pH 7.5	0	Dimer P 1 2 1	76.48 182.36 100.09 (90.0 107.92 90.0)	P 1 2 1	2.54	4	Xia2-DIALS	29/11/2019 14:35	-	PEG	Q90H	-	D69Y	no
G1-068	XX17RECQ L5A-x057	Fresh	14.3505	20	CI066033	HIN3-170302-01	F	12	c	0.2M sodium chloride -- 25% PEG3350 -- 0.1M HEPES pH 7.5	0	Dimer P 1 2 1	76.16 182.28 100.78 (90.0 108.12 90.0)	P 1 2 1	2.54	5	Xia2-DIALS	29/11/2019 14:25	-	PEG	Q90H	-	D69Y	no
G1-068	XX17RECQ L5A-x057	Fresh	14.3505	20	CI066033	HIN3-170302-01	F	12	c	0.2M sodium chloride -- 25% PEG3350 -- 0.1M HEPES pH 7.5	0	Dimer P 1 2 1	76.13 181.91 100.69 (90.0 108.09 90.0)	P 1 21 1	2.54	5	Xia2-DIALS	29/11/2019 13:30	-	PEG	Q90H	-	D69Y	no
G1-068	XX17RECQ L5A-x103	Fresh	11.7439	20	CI079094	HIN3-170302-01	F	11	c	0.2M sodium chloride -- 25% PEG3350 -- 0.1M bis-tris pH 6.5	0	Dimer P 1 2 1	75.58 181.89 100.09 (90.0 107.8 90.0)	P 1 21 1	2.65	5	Xia2-DIALS	14/12/2019 05:58	-	PEG	Q90H	-	D69Y	no
G1-068	XX17RECQ L5A-x053	Fresh	14.3505	20	CI066033	HIN3-170302-01	F	10	c	0.2M sodium chloride -- 25% PEG3350 -- 0.1M bis-tris pH 5.5	0	Dimer P 1 2 1	75.49 182.13 100.52 (90.0 107.78 90.0)	P 1 21 1	2.69	5	Xia2-DIALS	29/11/2019 13:32	-	PEG	Q90H	-	D69Y	no
G1-068	XX17RECQ L5A-x052	Fresh	14.3505	20	CI066033	HIN3-170302-01	F	10	c	0.2M sodium chloride -- 25% PEG3350 -- 0.1M bis-tris pH 5.5	0	Dimer P 1 2 1	75.46 181.97 100.42 (90.0 107.81 90.0)	P 1 21 1	2.82	5	Xia2-DIALS	29/11/2019 13:33	-	PEG	Q90H	-	D69Y	no
G1-068	XX17RECQ L5A-x055	Fresh	14.3505	20	CI066033	HIN3-170302-01	F	10	c	0.2M sodium chloride -- 25% PEG3350 -- 0.1M bis-tris pH 5.5	0	Dimer P 1 2 1	75.03 180.41 99.77 (90.0 107.49 90.0)	P 1 2 1	2.95	5	Xia2-DIALS	29/11/2019 15:07	-	PEG	Q90H	-	D69Y	no
G1-068	XX17RECQ L5A-x043	Fresh	14.3505	20	CI066033	HIN3-170302-01	F	6	c	0.2M ammonium sulfate -- 25% PEG3350 -- 0.1M bis-tris pH 5.5	0	Dimer P 1 2 1	75.98 184.24 99.36 (90.0 107.45 90.0)	P 1 21 1	3.07	5	Xia2-DIALS	29/11/2019 14:07	-	PEG	Q90H	-	D69Y	no
G1-068	XX17RECQ L5A-x067	Fresh	14.3505	20	CI066033	HIN3-170302-01	G	5	c	0.2M lithium sulfate -- 25% PEG3350 -- 0.1M tris pH 8.5	-1	NA	115.18 115.02 173.41 (89.99 89.95 60.07)	P 1	3.08	2	Xia2-DIALS	29/11/2019 15:33	-	PEG	Q90H	-	D69Y	no
G1-068	XX17RECQ L5A-x050	Fresh	14.3505	20	CI066033	HIN3-170302-01	F	10	c	0.2M sodium chloride -- 25% PEG3350 -- 0.1M bis-tris pH 5.5	0	Dimer P 1 2 1	75.63 182.35 100.59 (90.0 107.84 90.0)	P 1 21 1	3.1	5	Xia2-DIALS	29/11/2019 13:41	-	PEG	Q90H	-	D69Y	no
G1-068	XX17RECQ L5A-x054	Fresh	14.3505	20	CI066033	HIN3-170302-01	F	10	c	0.2M sodium chloride -- 25% PEG3350 -- 0.1M bis-tris pH 5.5	0	Dimer P 1 2 1	75.06 178.99 98.79 (90.0 107.3 90.0)	P 1 21 1	3.12	5	Xia2-DIALS	29/11/2019 13:28	-	PEG	Q90H	-	D69Y	no
G1-068	XX17RECQ L5A-x074	Fresh	14.3505	20	CI066033	HIN3-170302-01	G	6	c	0.2M ammonium acetate -- 25% PEG3350 -- 0.1M bis-tris pH 5.5	0	Dimer P 1 2 1	76.05 183.52 101.05 (90.0 107.88 90.0)	P 1 21 1	3.22	5	Xia2-DIALS	29/11/2019 14:16	-	PEG	Q90H	-	D69Y	no

Table S4 Diffraction data

Nanobody grouped ID	Mounted Crystal ID	Xtal Plate Protein Frozen/Fresh?	Xtal Plate Protein Concentration (mg/ml)	Xtal Plate Temperature	Xtal Plate Barcode	Screen Batch Name	XTBM Row	XTBM Column	XTBM Subwell	XTBM Condition	Crystal Form Group	Cystal Form Annotation	Unit Cell Dimensions	Space group	Resolution	Number of Successful Autoprocessing Pipelines	Autoprocessing Pipeline Used	Experiment Time	Soaking condition	Xtal Group	Surface Mutations Around Nbinter-1	Key Mutations	Crystal Epitope Mutations	Condition Repitition?
G1-068	XX17RECQ L5A-x051	Fresh	14.3505	20	CI066033	HIN3-170302-01	F	10	c	0.2M sodium chloride -- 25% PEG3350 -- 0.1M bis-tris pH 5.5	0	Dimer P 1 2 1	74.96 188.87 97.4 (90.0 107.77 90.0)	P 1 21 1	3.25	4	Xia2-DIALS	29/11/2019 13:35	-	PEG	Q90H	-	D69Y	no
G1-068	XX17RECQ L5A-x059	Fresh	14.3505	20	CI066033	HIN3-170302-01	G	2	c	0.2M lithium sulfate -- 25% PEG3350 -- 0.1M bis-tris pH 5.5	0	Dimer P 1 2 1	75.99 184.18 98.76 (90.0 107.56 90.0)	P 1 21 1	3.27	5	Xia2-DIALS	29/11/2019 15:05	-	PEG	Q90H	-	D69Y	no
G1-068	XX17RECQ L5A-x077	Fresh	14.3505	20	CI066033	HIN3-170302-01	G	7	c	0.2M ammonium acetate -- 25% PEG3350 -- 0.1M bis-tris pH 6.5	0	Dimer P 1 2 1	75.53 184.12 100.39 (90.0 107.78 90.0)	P 1 21 1	3.28	5	Xia2-DIALS	29/11/2019 13:39	-	PEG	Q90H	-	D69Y	no
G1-068	XX17RECQ L5A-x064	Fresh	14.3505	20	CI066033	HIN3-170302-01	G	5	a	0.2M lithium sulfate -- 25% PEG3350 -- 0.1M tris pH 8.5	0	Dimer P 1 2 1	76.88 99.2 184.32 (89.78 89.74 72.06)	P 1 2 1	3.28	5	Xia2-DIALS	29/11/2019 13:24	-	PEG	Q90H	-	D69Y	no
G1-068	XX17RECQ L5A-x073	Fresh	14.3505	20	CI066033	HIN3-170302-01	G	6	c	0.2M ammonium acetate -- 25% PEG3350 -- 0.1M bis-tris pH 5.5	0	Dimer P 1 2 1	75.32 182.72 99.91 (90.0 107.69 90.0)	P 1 21 1	3.31	5	Xia2-DIALS	29/11/2019 15:13	-	PEG	Q90H	-	D69Y	no
G1-068	XX17RECQ L5A-x041	Fresh	14.3505	20	CI066033	HIN3-170302-01	F	6	c	0.2M ammonium sulfate -- 25% PEG3350 -- 0.1M bis-tris pH 5.5	0	Dimer P 1 2 1	76.54 186.7 99.25 (90.0 107.76 90.0)	P 1 21 1	3.33	2	Xia2-DIALS	29/11/2019 14:11	-	PEG	Q90H	-	D69Y	no
G1-068	XX17RECQ L5A-x060	Fresh	14.3505	20	CI066033	HIN3-170302-01	G	2	c	0.2M lithium sulfate -- 25% PEG3350 -- 0.1M bis-tris pH 5.5	0	Dimer P 1 2 1	76.89 188.03 99.05 (90.0 107.88 90.0)	P 1 21 1	3.37	4	Xia2-DIALS	29/11/2019 13:26	-	PEG	Q90H	-	D69Y	no
G1-068	XX17RECQ L5A-x046	Fresh	14.3505	20	CI066033	HIN3-170302-01	F	7	c	0.2M ammonium sulfate -- 25% PEG3350 -- 0.1M bis-tris pH 6.5	0	Dimer P 1 2 1	76.56 186.0 98.64 (90.0 107.97 90.0)	P 1 21 1	3.37	2	Xia2-DIALS	29/11/2019 14:09	-	PEG	Q90H	-	D69Y	no
G1-068	XX17RECQ L5A-x076	Fresh	14.3505	20	CI066033	HIN3-170302-01	G	7	c	0.2M ammonium acetate -- 25% PEG3350 -- 0.1M bis-tris pH 6.5	0	Dimer P 1 2 1	75.67 100.67 184.68 (90.03 89.88 72.24)	P 1 2 1	3.38	5	Xia2-DIALS	29/11/2019 13:43	-	PEG	Q90H	-	D69Y	no
G1-068	XX17RECQ L5A-x066	Fresh	14.3505	20	CI066033	HIN3-170302-01	G	5	c	0.2M lithium sulfate -- 25% PEG3350 -- 0.1M tris pH 8.5	1	Dimer C 2 2 21	115.59 199.7 174.17 (90.0 90.0 90.0)	C 2 2 21	3.44	3	Xia2-DIALS	29/11/2019 15:20	-	PEG	Q90H	-	D69Y	no
G1-068	XX17RECQ L5A-x042	Fresh	14.3505	20	CI066033	HIN3-170302-01	F	6	c	0.2M ammonium sulfate -- 25% PEG3350 -- 0.1M bis-tris pH 5.5	0	Dimer P 1 2 1	75.88 181.16 98.09 (90.0 107.27 90.0)	P 1 21 1	3.52	5	Xia2-DIALS	29/11/2019 14:53	-	PEG	Q90H	-	D69Y	no
G1-068	XX17RECQ L5A-x102	Fresh	11.7439	20	CI079094	HIN3-170302-01	F	11	c	0.2M sodium chloride -- 25% PEG3350 -- 0.1M bis-tris pH 6.5	0	Dimer P 1 2 1	76.26 95.68 179.68 (89.46 89.86 72.46)	P 1 2 1	3.69	1	Xia2-DIALS	14/12/2019 05:56	-	PEG	Q90H	-	D69Y	no
G1-068	XX17RECQ L5A-x045	Fresh	14.3505	20	CI066033	HIN3-170302-01	F	6	c	0.2M ammonium sulfate -- 25% PEG3350 -- 0.1M bis-tris pH 5.5	0	Dimer P 1 2 1	75.84 182.59 98.43 (90.0 107.29 90.0)	P 1 21 1	3.71	4	Xia2-DIALS	29/11/2019 14:50	-	PEG	Q90H	-	D69Y	no
G1-068	XX17RECQ L5A-x070	Fresh	14.3505	20	CI066033	HIN3-170302-01	G	6	c	0.2M ammonium acetate -- 25% PEG3350 -- 0.1M bis-tris pH 5.5	0	Dimer P 1 2 1	75.4 183.51 100.23 (90.0 107.74 90.0)	P 1 21 1	3.8	2	Autoproc	29/11/2019 13:45	-	PEG	Q90H	-	D69Y	no
G1-068	XX17RECQ L5A-x069	Fresh	14.3505	20	CI066033	HIN3-170302-01	G	6	c	0.2M ammonium acetate -- 25% PEG3350 -- 0.1M bis-tris pH 5.5	0	Dimer P 1 2 1	75.11 181.74 99.5 (90.0 107.58 90.0)	P 1 2 1	3.85	3	Autoproc	29/11/2019 15:18	-	PEG	Q90H	-	D69Y	no

Table S4 Diffraction data

Nanobody grouped ID	Mounted Crystal ID	Xtal Plate Protein Frozen/Fresh?	Xtal Plate Protein Concentration (mg/ml)	Xtal Plate Temperature	Xtal Plate Barcode	Screen Batch Name	XTBM Row	XTBM Column	XTBM Subwell	XTBM Condition	Crystal Form Group	Cystal Form Annotation	Unit Cell Dimensions	Space group	Resolution	Number of Successful Autoprocessing Pipelines	Autoprocessing Pipeline Used	Experiment Time	Soaking condition	Xtal Group	Surface Mutations Around Nbinter-1	Key Mutations	Crystal Epitope Mutations	Condition Repitition?
G1-068	XX17RECQ L5A-x047	Fresh	14.3505	20	CI066033	HIN3-170302-01	F	7	c	0.2M ammonium sulfate -- 25% PEG3350 -- 0.1M bis-tris pH 6.5	0	Dimer P 1 2 1	76.61 185.29 99.04 (90.0 107.82 90.0)	P 1 21 1	3.87	4	Xia2-3dii	29/11/2019 14:04	-	PEG	Q90H	-	D69Y	no
G1-068	XX17RECQ L5A-x095	Fresh	11.7439	20	CI079094	HIN3-170302-01	F	6	c	0.2M ammonium sulfate -- 25% PEG3350 -- 0.1M bis-tris pH 5.5	0	Dimer P 1 2 1	76.63 96.55 183.76 (89.96 89.95 72.65)	P 1 2 1	5.78	1	Xia2-DIALS	14/12/2019 08:56	-	PEG	Q90H	-	D69Y	no
G1-068	XX17RECQ L5A-x075	Fresh	14.3505	20	CI066033	HIN3-170302-01	G	6	c	0.2M ammonium acetate -- 25% PEG3350 -- 0.1M bis-tris pH 5.5	0	Dimer P 1 2 1	75.39 182.04 100.06 (90.0 107.68 90.0)	P 1 21 1	8.1	4	Xia2-DIALS	29/11/2019 13:22	-	PEG	Q90H	-	D69Y	no
G1-068	XX17RECQ L5A-x097	Fresh	11.7439	20	CI079094	HIN3-170302-01	F	7	a	0.2M ammonium sulfate -- 25% PEG3350 -- 0.1M bis-tris pH 6.5	-1	NA	76.29 94.16 194.2 (82.98 66.81 72.44)	P 1	NA	1	NA	14/12/2019 09:02	-	PEG	Q90H	-	D69Y	no
G1-068	XX17RECQ L5A-x110	Fresh	11.7439	20	CI079094	HIN3-170302-01	G	11	c	0.2M magnesium chloride -- 25% PEG3350 -- 0.1M bis-tris pH 6.5	NA		NA	NA	NA	0	NA	14/12/2019 06:14	-	PEG	Q90H	-	D69Y	no
G1-068	XX17RECQ L5A-x109	Fresh	11.7439	20	CI079094	HIN3-170302-01	G	10	c	0.2M magnesium chloride -- 25% PEG3350 -- 0.1M bis-tris pH 5.5	NA		NA	NA	NA	0	NA	14/12/2019 06:11	-	PEG	Q90H	-	D69Y	no
G1-068	XX17RECQ L5A-x108	Fresh	11.7439	20	CI079094	HIN3-170302-01	G	10	c	0.2M magnesium chloride -- 25% PEG3350 -- 0.1M bis-tris pH 5.5	NA		NA	NA	NA	0	NA	14/12/2019 06:09	-	PEG	Q90H	-	D69Y	no
G1-068	XX17RECQ L5A-x107	Fresh	11.7439	20	CI079094	HIN3-170302-01	G	7	c	0.2M ammonium acetate -- 25% PEG3350 -- 0.1M bis-tris pH 6.5	NA		NA	NA	NA	0	NA	14/12/2019 06:07	-	PEG	Q90H	-	D69Y	no
G1-068	XX17RECQ L5A-x106	Fresh	11.7439	20	CI079094	HIN3-170302-01	G	6	c	0.2M ammonium acetate -- 25% PEG3350 -- 0.1M bis-tris pH 5.5	NA		NA	NA	NA	0	NA	14/12/2019 06:05	-	PEG	Q90H	-	D69Y	no
G1-068	XX17RECQ L5A-x105	Fresh	11.7439	20	CI079094	HIN3-170302-01	G	6	c	0.2M ammonium acetate -- 25% PEG3350 -- 0.1M bis-tris pH 5.5	NA		NA	NA	NA	0	NA	14/12/2019 06:02	-	PEG	Q90H	-	D69Y	no
G1-068	XX17RECQ L5A-x104	Fresh	11.7439	20	CI079094	HIN3-170302-01	G	5	c	0.2M lithium sulfate -- 25% PEG3350 -- 0.1M tris pH 8.5	NA		NA	NA	NA	0	NA	14/12/2019 06:00	-	PEG	Q90H	-	D69Y	no
G1-068	XX17RECQ L5A-x101	Fresh	11.7439	20	CI079094	HIN3-170302-01	F	10	c	0.2M sodium chloride -- 25% PEG3350 -- 0.1M bis-tris pH 5.5	NA		NA	NA	NA	0	NA	14/12/2019 05:53	-	PEG	Q90H	-	D69Y	no
G1-068	XX17RECQ L5A-x100	Fresh	11.7439	20	CI079094	HIN3-170302-01	F	10	c	0.2M sodium chloride -- 25% PEG3350 -- 0.1M bis-tris pH 5.5	NA		NA	NA	NA	0	NA	14/12/2019 05:51	-	PEG	Q90H	-	D69Y	no
G1-068	XX17RECQ L5A-x099	Fresh	11.7439	20	CI079094	HIN3-170302-01	F	7	d	0.2M ammonium sulfate -- 25% PEG3350 -- 0.1M bis-tris pH 6.5	NA		NA	NA	NA	0	NA	14/12/2019 09:06	-	PEG	Q90H	-	D69Y	no
G1-068	XX17RECQ L5A-x098	Fresh	11.7439	20	CI079094	HIN3-170302-01	F	7	a	0.2M ammonium sulfate -- 25% PEG3350 -- 0.1M bis-tris pH 6.5	NA		NA	NA	NA	0	NA	14/12/2019 09:04	-	PEG	Q90H	-	D69Y	no

Table S4 Diffraction data

Nanobody grouped ID	Mounted Crystal ID	Xtal Plate Protein Frozen/Fresh?	Xtal Plate Protein Concentration (mg/ml)	Xtal Plate Temperature	Xtal Plate Barcode	Screen Batch Name	XTBM Row	XTBM Column	XTBM Subwell	XTBM Condition	Crystal Form Group	Cystal Form Annotation	Unit Cell Dimensions	Space group	Resolution	Number of Successful Autoprocessing Pipelines	Autoprocessing Pipeline Used	Experiment Time	Soaking condition	Xtal Group	Surface Mutations Around Nbinter-1	Key Mutations	Crystal Epitope Mutations	Condition Repitition?
G1-068	XX17RECQ L5A-x096	Fresh	11.7439	20	CI079094	HIN3-170302-01	F	6	c	0.2M ammonium sulfate -- 25% PEG3350 -- 0.1M bis-tris pH 5.5	NA		NA	NA	NA	0	NA	14/12/2019 08:58	-	PEG	Q90H	-	D69Y	no
G1-068	XX17RECQ L5A-x094	Fresh	11.7439	20	CI079094	HIN3-170302-01	D	7	a	25% PEG3350 -- 0.1M bis-tris pH 6.5	NA		NA	NA	NA	0	NA	14/12/2019 08:53	-	PEG	Q90H	-	D69Y	no
G1-068	XX17RECQ L5A-x093	Fresh	11.7439	20	CI079094	HIN3-170302-01	D	6	c	25% PEG3350 -- 0.1M bis-tris pH 5.5	NA		NA	NA	NA	0	NA	14/12/2019 08:51	-	PEG	Q90H	-	D69Y	no
G1-068	XX17RECQ L5A-x092	Fresh	11.7439	20	CI079094	HIN3-170302-01	D	6	a	25% PEG3350 -- 0.1M bis-tris pH 5.5	NA		NA	NA	NA	0	NA	14/12/2019 08:48	-	PEG	Q90H	-	D69Y	no
G1-068	XX17RECQ L5A-x081	Fresh	14.3505	20	CI066033	HIN3-170302-01	H	2	d	0.2M sodium/potassium tartrate -- 20% PEG3350	NA		NA	NA	NA	0	NA	29/11/2019 15:24	-	PEG	Q90H	-	D69Y	no
G1-068	XX17RECQ L5A-x080	Fresh	14.3505	20	CI066033	HIN3-170302-01	G	7	c	0.2M ammonium acetate -- 25% PEG3350 -- 0.1M bis-tris pH 6.5	NA		NA	NA	NA	0	NA	29/11/2019 15:10	-	PEG	Q90H	-	D69Y	no
G1-068	XX17RECQ L5A-x072	Fresh	14.3505	20	CI066033	HIN3-170302-01	G	6	c	0.2M ammonium acetate -- 25% PEG3350 -- 0.1M bis-tris pH 5.5	NA		NA	NA	NA	0	NA	29/11/2019 15:15	-	PEG	Q90H	-	D69Y	no
G1-068	XX17RECQ L5A-x071	Fresh	14.3505	20	CI066033	HIN3-170302-01	G	6	c	0.2M ammonium acetate -- 25% PEG3350 -- 0.1M bis-tris pH 5.5	NA		NA	NA	NA	0	NA	29/11/2019 15:16	-	PEG	Q90H	-	D69Y	no
G1-068	XX17RECQ L5A-x062	Fresh	14.3505	20	CI066033	HIN3-170302-01	G	4	c	0.2M lithium sulfate -- 25% PEG3350 -- 0.1M HEPES pH 7.5	NA		NA	NA	NA	0	NA	29/11/2019 15:30	-	PEG	Q90H	-	D69Y	no
G1-071	XX17RECQ L5A-x038	Fresh	13.2694	20	CI066031	HIN3-170302-01	F	7	d	0.2M ammonium sulfate -- 25% PEG3350 -- 0.1M bis-tris pH 6.5	0	Dimer P 1 2 1	76.79 188.38 99.98 (90.0 107.84 90.0)	P 1 21 1	3.07	5	Xia2-DIALS	29/11/2019 14:14	-	PEG	N92D	-	D69Y	no
G1-071	XX17RECQ L5A-x039	Fresh	13.2694	20	CI066031	HIN3-170302-01	F	7	d	0.2M ammonium sulfate -- 25% PEG3350 -- 0.1M bis-tris pH 6.5	0	Dimer P 1 2 1	77.13 189.38 99.41 (90.0 108.0 90.0)	P 1 21 1	3.34	5	Xia2-DIALS	29/11/2019 14:12	-	PEG	N92D	-	D69Y	no
G1-071	XX17RECQ L5A-x113	Fresh	12.1523	20	CI079093	HIN3-170302-01	H	12	c	0.1M potassium bromide -- 30% PEG2000MME	NA		NA	NA	NA	0	NA	14/12/2019 06:22	-	PEG	N92D	-	D69Y	no
G1-071	XX17RECQ L5A-x112	Fresh	12.1523	20	CI079093	HIN3-170302-01	F	7	d	0.2M ammonium sulfate -- 25% PEG3350 -- 0.1M bis-tris pH 6.5	NA		NA	NA	NA	0	NA	14/12/2019 06:19	-	PEG	N92D	-	D69Y	no
G1-071	XX17RECQ L5A-x111	Fresh	12.1523	20	CI079093	HIN3-170302-01	F	7	d	0.2M ammonium sulfate -- 25% PEG3350 -- 0.1M bis-tris pH 6.5	NA		NA	NA	NA	0	NA	14/12/2019 06:17	-	PEG	N92D	-	D69Y	no
G2*-004	XX21RECQ L5A-x1035	Fresh	32.3	20	CI078926	HIN3-170302-01	D	7	c	25% PEG3350 -- 0.1M bis-tris pH 6.5	0	Dimer P 1 2 1	74.25 183.78 99.84 (90.0 107.41 90.0)	P 1 2 1	2.46	9	Xia2-DIALS	17/01/2020 19:31	-	PEG	-	S7N:L12C	D69Y	no

Table S4 Diffraction data

Nanobody grouped ID	Mounted Crystal ID	Xtal Plate Protein Frozen/Fresh?	Xtal Plate Protein Concentration (mg/ml)	Xtal Plate Temperature	Xtal Plate Barcode	Screen Batch Name	XTBM Row	XTBM Column	XTBM Subwell	XTBM Condition	Crystal Form Group	Cystal Form Annotation	Unit Cell Dimensions	Space group	Resolution	Number of Successful Autoprocessing Pipelines	Autoprocessing Pipeline Used	Experiment Time	Soaking condition	Xtal Group	Surface Mutations Around Nbinter-1	Key Mutations	Crystal Epitope Mutations	Condition Repitition?
G2*-004	XX21RECQ L5A-x1053	Fresh	29	20	CI078928	HIN3-170302-01	D	7	c	25% PEG3350 -- 0.1M bis-tris pH 6.5	0	Dimer P 1 2 1	74.93 186.63 100.3 (90.0 107.68 90.0)	P 1 2 1	2.53	9	Xia2-DIALS	17/01/2020 23:10	-	PEG	-	S7N:L12C	D69Y	no
G2*-004	XX21RECQ L5A-x1043	Fresh	29	20	CI078928	HIN3-170302-01	H	11	c	0.1M potassium thiocyanate -- 30% PEG2000MME	0	Dimer P 1 2 1	74.04 182.98 101.05 (90.0 107.52 90.0)	P 1 2 1	2.55	9	Xia2-DIALS	17/01/2020 22:40	-	PEG	-	S7N:L12C	D69Y	no
G2*-004	XX21RECQ L5A-x1006	Fresh	29.7	20	CI079019	HIN3-170302-01	G	12	c	0.2M magnesium chloride -- 25% PEG3350 -- 0.1M HEPES pH 7.5	0	Dimer P 1 2 1	75.48 183.54 99.9 (90.0 107.85 90.0)	P 1 2 1	2.61	9	Xia2-DIALS	17/01/2020 17:35	-	PEG	-	S7N:L12C	D69Y	no
G2*-004	XX21RECQ L5A-x1040	Fresh	19.37	20	CI078927	HIN3-170302-01	D	7	c	25% PEG3350 -- 0.1M bis-tris pH 6.5	0	Dimer P 1 2 1	74.46 185.0 99.64 (90.0 107.46 90.0)	P 1 2 1	2.63	9	Xia2-DIALS	17/01/2020 19:45	-	PEG	-	S7N:L12C	D69Y	no
G2*-004	XX21RECQ L5A-x1042	Fresh	29	20	CI078928	HIN3-170302-01	H	12	c	0.1M potassium bromide -- 30% PEG2000MME	0	Dimer P 1 2 1	74.22 183.32 100.2 (90.0 107.21 90.0)	P 1 2 1	2.68	9	Xia2-DIALS	17/01/2020 22:37	-	PEG	-	S7N:L12C	D69Y	no
G2*-004	XX21RECQ L5A-x1036	Fresh	32.3	20	CI078926	HIN3-170302-01	D	7	a	25% PEG3350 -- 0.1M bis-tris pH 6.5	0	Dimer P 1 2 1	74.81 184.88 99.69 (90.0 107.64 90.0)	P 1 2 1	2.68	9	Xia2-DIALS	17/01/2020 19:34	-	PEG	-	S7N:L12C	D69Y	no
G2*-004	XX21RECQ L5A-x1037	Fresh	32.3	20	CI078926	HIN3-170302-01	D	6	c	25% PEG3350 -- 0.1M bis-tris pH 5.5	0	Dimer P 1 2 1	74.38 182.95 99.37 (90.0 107.37 90.0)	P 1 2 1	2.72	9	Xia2-DIALS	17/01/2020 19:36	-	PEG	-	S7N:L12C	D69Y	no
G2*-004	XX21RECQ L5A-x1051	Fresh	29	20	CI078928	HIN3-170302-01	D	11	c	28% PEG2000MME -- 0.1M bis-tris pH 6.5	0	Dimer P 1 2 1	74.25 184.3 99.9 (90.0 107.44 90.0)	P 1 21 1	2.77	9	Xia2-DIALS	17/01/2020 23:04	-	PEG	-	S7N:L12C	D69Y	no
G2*-004	XX21RECQ L5A-x1008	Fresh	29.7	20	CI079019	HIN3-170302-01	G	11	c	0.2M magnesium chloride -- 25% PEG3350 -- 0.1M bis-tris pH 6.5	0	Dimer P 1 2 1	74.9 184.47 99.83 (90.0 107.6 90.0)	P 1 21 1	2.82	9	Xia2-DIALS	17/01/2020 15:56	-	PEG	-	S7N:L12C	D69Y	no
G2*-004	XX21RECQ L5A-x1034	Fresh	32.3	20	CI078926	HIN3-170302-01	D	11	c	28% PEG2000MME -- 0.1M bis-tris pH 6.5	0	Dimer P 1 2 1	74.24 184.7 100.08 (90.0 107.43 90.0)	P 1 21 1	2.83	9	Xia2-DIALS	17/01/2020 19:29	-	PEG	-	S7N:L12C	D69Y	no
G2*-004	XX21RECQ L5A-x1001	Fresh	29.7	20	CI079019	HIN3-170302-01	D	7	c	25% PEG3350 -- 0.1M bis-tris pH 6.5	0	Dimer P 1 2 1	75.16 185.9 100.42 (90.0 107.71 90.0)	P 1 21 1	2.9	9	Xia2-DIALS	17/01/2020 17:21	-	PEG	-	S7N:L12C	D69Y	no
G2*-004	XX21RECQ L5A-x1012	Fresh	21.3	20	CI078925	HIN3-170302-01	D	7	c	25% PEG3350 -- 0.1M bis-tris pH 6.5	0	Dimer P 1 2 1	74.73 185.79 99.79 (90.0 107.49 90.0)	P 1 21 1	2.96	9	Xia2-DIALS	17/01/2020 16:06	-	PEG	-	S7N:L12C	D69Y	no
G2*-004	XX21RECQ L5A-x1022	Fresh	32.3	20	CI078926	HIN3-170302-01	G	11	c	0.2M magnesium chloride -- 25% PEG3350 -- 0.1M bis-tris pH 6.5	0	Dimer P 1 2 1	74.85 185.09 99.6 (90.0 107.44 90.0)	P 1 21 1	3.01	9	Xia2-DIALS	17/01/2020 16:19	-	PEG	-	S7N:L12C	D69Y	no
G2*-004	XX21RECQ L5A-x1000	Fresh	29.7	20	CI079019	HIN3-170302-01	D	7	a	25% PEG3350 -- 0.1M bis-tris pH 6.5	0	Dimer P 1 2 1	75.19 186.01 100.23 (90.0 107.76 90.0)	P 1 21 1	3.01	9	Xia2-DIALS	17/01/2020 17:17	-	PEG	-	S7N:L12C	D69Y	no
G2*-004	XX21RECQ L5A-x1054	Fresh	29	20	CI078928	HIN3-170302-01	D	6	c	25% PEG3350 -- 0.1M bis-tris pH 5.5	0	Dimer P 1 2 1	74.83 186.29 100.44 (90.0 107.68 90.0)	P 1 2 1	3.1	9	Xia2-DIALS	17/01/2020 23:13	-	PEG	-	S7N:L12C	D69Y	no

Table S4 Diffraction data

Nanobody grouped ID	Mounted Crystal ID	Xtal Plate Protein Frozen/Fresh?	Xtal Plate Protein Concentration (mg/ml)	Xtal Plate Temperature	Xtal Plate Barcode	Screen Batch Name	XTBM Row	XTBM Column	XTBM Subwell	XTBM Condition	Crystal Form Group	Cystal Form Annotation	Unit Cell Dimensions	Space group	Resolution	Number of Successful Autoprocessing Pipelines	Autoprocessing Pipeline Used	Experiment Time	Soaking condition	Xtal Group	Surface Mutations Around Nbinter-1	Key Mutations	Crystal Epitope Mutations	Condition Repitition?
G2*-004	XX21RECQ L5A-x1025	Fresh	32.3	20	CI078926	HIN3-170302-01	G	8	a	0.2M ammonium acetate -- 25% PEG3350 -- 0.1M HEPES pH 7.5	-1	NA	113.91 114.31 173.2 (89.99 89.96 60.12)	P 1	3.13	3	Xia2-DIALS	17/01/2020 16:27	-	PEG	-	S7N:L12C	D69Y	no
G2*-004	XX21RECQ L5A-x1055	Fresh	29	20	CI078928	HIN3-170302-01	D	6	a	25% PEG3350 -- 0.1M bis-tris pH 5.5	0	Dimer P 1 2 1	74.93 185.17 99.72 (90.0 107.5 90.0)	P 1 21 1	3.26	9	Xia2-DIALS	17/01/2020 21:29	-	PEG	-	S7N:L12C	D69Y	no
G2*-004	XX21RECQ L5A-x1013	Fresh	21.3	20	CI078925	HIN3-170302-01	D	7	a	25% PEG3350 -- 0.1M bis-tris pH 6.5	0	Dimer P 1 2 1	75.16 186.28 100.18 (90.0 107.76 90.0)	P 1 21 1	3.27	9	Xia2-DIALS	17/01/2020 16:08	-	PEG	-	S7N:L12C	D69Y	no
G2*-004	XX21RECQ L5A-x1015	Fresh	24.8	20	CI078929	HIN3-170302-01	G	4	c	0.2M lithium sulfate -- 25% PEG3350 -- 0.1M HEPES pH 7.5	1	Dimer C 2 2 21	115.61 199.12 172.93 (90.0 90.0 90.0)	C 2 2 21	3.32	5	Xia2-DIALS	17/01/2020 22:24	-	PEG	-	S7N:L12C	D69Y	no
G2*-004	XX21RECQ L5A-x1031	Fresh	32.3	20	CI078926	HIN3-170302-01	F	7	c	0.2M ammonium sulfate -- 25% PEG3350 -- 0.1M bis-tris pH 6.5	1	Dimer C 2 2 21	115.05 196.92 174.09 (90.0 90.0 90.0)	C 2 2 21	3.35	4	Xia2-DIALS	17/01/2020 19:21	-	PEG	-	S7N:L12C	D69Y	no
G2*-004	XX21RECQ L5A-x1027	Fresh	32.3	20	CI078926	HIN3-170302-01	G	1	c	0.2M sodium chloride -- 25% PEG3350 -- 0.1M tris pH 8.5	-1	NA	112.29 172.97 112.47 (90.0 119.87 90.0)	P 1 2 1	3.38	3	Xia2-DIALS	17/01/2020 19:08	-	PEG	-	S7N:L12C	D69Y	no
G2*-004	XX21RECQ L5A-x1176	Fresh	12	20	CI078967	HIN3-170302-01	F	9	c	0.2M ammonium sulfate -- 25% PEG3350 -- 0.1M HEPES pH 7.5	1	Dimer C 2 2 21	116.48 199.32 174.41 (90.0 90.0 90.0)	C 2 2 21	3.49	4	Xia2-DIALS	17/01/2020 18:07	-	PEG	-	S7N:L12C	D69Y	no
G2*-004	XX21RECQ L5A-x1018	Fresh	32.3	20	CI078926	HIN3-170302-01	H	10	a	0.2M sodium citrate tribasic -- 20% PEG3350	1	Dimer C 2 2 21	115.56 198.52 173.94 (90.0 90.0 90.0)	C 2 2 21	3.5	7	Xia2-DIALS	17/01/2020 16:10	-	PEG	-	S7N:L12C	D69Y	no
G2*-004	XX21RECQ L5A-x1014	Fresh	24.8	20	CI078929	HIN3-170302-01	G	5	c	0.2M lithium sulfate -- 25% PEG3350 -- 0.1M tris pH 8.5	1	Dimer C 2 2 21	117.47 200.42 171.65 (90.0 90.0 90.0)	C 2 2 21	3.53	3	Xia2-DIALS	17/01/2020 19:53	-	PEG	-	S7N:L12C	D69Y	no
G2*-004	XX21RECQ L5A-x1002	Fresh	29.7	20	CI079019	HIN3-170302-01	G	4	a	0.2M lithium sulfate -- 25% PEG3350 -- 0.1M HEPES pH 7.5	1	Dimer C 2 2 21	115.33 199.28 172.29 (90.0 90.0 90.0)	C 2 2 21	3.56	3	Xia2-DIALS	17/01/2020 17:23	-	PEG	-	S7N:L12C	D69Y	no
G2*-004	XX21RECQ L5A-x1005	Fresh	29.7	20	CI079019	HIN3-170302-01	G	11	c	0.2M magnesium chloride -- 25% PEG3350 -- 0.1M bis-tris pH 6.5	0	Dimer P 1 2 1	74.88 182.41 98.99 (90.0 107.48 90.0)	P 1 21 1	3.61	9	Xia2-DIALS	17/01/2020 17:31	-	PEG	-	S7N:L12C	D69Y	no
G2*-004	XX21RECQ L5A-x1028	Fresh	32.3	20	CI078926	HIN3-170302-01	F	12	c	0.2M sodium chloride -- 25% PEG3350 -- 0.1M HEPES pH 7.5	1	Dimer C 2 2 21	113.53 195.87 174.12 (90.00 90.00 90.00)	C 2 2 21	3.71	3	Xia2-DIALS	17/01/2020 19:11	-	PEG	-	S7N:L12C	D69Y	no
G2*-004	XX21RECQ L5A-x1032	Fresh	32.3	20	CI078926	HIN3-170302-01	F	7	a	0.2M ammonium sulfate -- 25% PEG3350 -- 0.1M bis-tris pH 6.5	-1	NA	117.95 117.97 176.3 (90.01 89.92 60.66)	P 1	4.08	2	Xia2-DIALS	17/01/2020 19:24	-	PEG	-	S7N:L12C	D69Y	no
G2*-004	XX21RECQ L5A-x1023	Fresh	32.3	20	CI078926	HIN3-170302-01	G	10	c	0.2M magnesium chloride -- 25% PEG3350 -- 0.1M bis-tris pH 5.5	0	Dimer P 1 2 1	74.56 185.17 99.47 (90.0 107.76 90.0)	P 1 2 1	4.09	9	Xia2-DIALS	17/01/2020 16:22	-	PEG	-	S7N:L12C	D69Y	no
G2*-004	XX21RECQ L5A-x1033	Fresh	32.3	20	CI078926	HIN3-170302-01	F	2	a	0.2M trimethylamine N-oxide -- 20% PEG2000MME -- 0.1M tris pH 8.5	-1	NA	115.55 115.31 174.85 (89.98 89.97 59.92)	P 1	4.18	5	Xia2-DIALS	17/01/2020 19:26	-	PEG	-	S7N:L12C	D69Y	no

Table S4 Diffraction data

Nanobody grouped ID	Mounted Crystal ID	Xtal Plate Protein Frozen/Fresh?	Xtal Plate Protein Concentration (mg/ml)	Xtal Plate Temperature	Xtal Plate Barcode	Screen Batch Name	XTBM Row	XTBM Column	XTBM Subwell	XTBM Condition	Crystal Form Group	Cystal Form Annotation	Unit Cell Dimensions	Space group	Resolution	Number of Successful Autoprocessing Pipelines	Autoprocessing Pipeline Used	Experiment Time	Soaking condition	Xtal Group	Surface Mutations Around Nbinter-1	Key Mutations	Crystal Epitope Mutations	Condition Repitition?
G2*-004	XX21RECQ L5A-x1038	Fresh	32.3	20	CI078926	HIN3-170302-01	D	3	a	30% jeffamine ED-2003 -- 0.1M HEPES pH 7.0	-1	NA	114.03 114.17 176.67 (89.95 89.96 60.64)	P 1	4.39	3	Xia2-DIALS	17/01/2020 19:39	-	PEG	-	S7N:L12C	D69Y	no
G2*-004	XX21RECQ L5A-x1004	Fresh	29.7	20	CI079019	HIN3-170302-01	G	10	c	0.2M magnesium chloride -- 25% PEG3350 -- 0.1M bis-tris pH 5.5	0	Dimer P 1 2 1	74.68 180.86 94.6 (90.0 107.39 90.0)	P 1 21 1	4.94	3	Xia2-DIALS	17/01/2020 17:29	-	PEG	-	S7N:L12C	D69Y	no
G2*-004	XX21RECQ L5A-x1177	Fresh	12	20	CI078967	HIN3-170302-01	G	11	d	0.2M magnesium chloride -- 25% PEG3350 -- 0.1M bis-tris pH 6.5	0	Dimer P 1 2 1	74.82 181.51 96.11 (90.0 107.48 90.0)	P 1 21 1	6.19	9	Xia2-DIALS	17/01/2020 18:10	-	PEG	-	S7N:L12C	D69Y	no
G2*-004	XX21RECQ L5A-x1052	Fresh	29	20	CI078928	HIN3-170302-01	D	7	d	25% PEG3350 -- 0.1M bis-tris pH 6.5	0	Dimer P 1 2 1	74.7 180.77 94.08 (90.0 107.47 90.0)	P 1 2 1	6.21	4	Xia2-DIALS	17/01/2020 23:07	-	PEG	-	S7N:L12C	D69Y	no
G2*-004	XX21RECQ L5A-x1030	Fresh	32.3	20	CI078926	HIN3-170302-01	F	10	a	0.2M sodium chloride -- 25% PEG3350 -- 0.1M bis-tris pH 5.5	-1	NA	73.23 198.48 264.2 (90.0 90.0 90.0)	P 21 21 2	7.38	1	Xia2-3dii	17/01/2020 19:18	-	PEG	-	S7N:L12C	D69Y	no
G2*-004	XX21RECQ L5A-x1007	Fresh	29.7	20	CI079019	HIN3-170302-01	H	6	a	0.2M sodium formate -- 20% PEG3350	-1	NA	91.9 114.03 114.72 (62.52 67.78 81.93)	P 1	7.75	3	Xia2-DIALS	17/01/2020 15:54	-	PEG	-	S7N:L12C	D69Y	no
G2*-004	XX21RECQ L5A-x1044	Fresh	29	20	CI078928	HIN3-170302-01	H	10	a	0.2M sodium citrate tribasic -- 20% PEG3350	-1	NA	115.08 171.96 114.0 (90.0 120.42 90.0)	P 1 2 1	NA	1	NA	17/01/2020 22:43	-	PEG	-	S7N:L12C	D69Y	no
G2*-004	XX21RECQ L5A-x1010	Fresh	21.3	20	CI078925	HIN3-170302-01	F	12	a	0.2M sodium chloride -- 25% PEG3350 -- 0.1M HEPES pH 7.5	-1	NA	116.5 115.6 176.39 (89.9 90.07 60.15)	P 1	NA	1	NA	17/01/2020 16:01	-	PEG	-	S7N:L12C	D69Y	no
G2*-004	XX21RECQ L5A-x1175	Fresh	12	20	CI078967	HIN3-170302-01	F	8	c	0.2M ammonium sulfate -- 25% PEG3350 -- 0.1M tris pH 8.5	NA		NA	NA	NA	0	NA	17/01/2020 18:05	-	PEG	-	S7N:L12C	D69Y	no
G2*-004	XX21RECQ L5A-x1050	Fresh	29	20	CI078928	HIN3-170302-01	F	12	c	0.2M sodium chloride -- 25% PEG3350 -- 0.1M HEPES pH 7.5	NA		NA	NA	NA	0	NA	17/01/2020 23:00	-	PEG	-	S7N:L12C	D69Y	no
G2*-004	XX21RECQ L5A-x1049	Fresh	29	20	CI078928	HIN3-170302-01	G	3	c	0.2M lithium sulfate -- 25% PEG3350 -- 0.1M bis-tris pH 6.5	NA		NA	NA	NA	0	NA	17/01/2020 22:58	-	PEG	-	S7N:L12C	D69Y	no
G2*-004	XX21RECQ L5A-x1048	Fresh	29	20	CI078928	HIN3-170302-01	G	8	a	0.2M ammonium acetate -- 25% PEG3350 -- 0.1M HEPES pH 7.5	NA		NA	NA	NA	0	NA	17/01/2020 22:55	-	PEG	-	S7N:L12C	D69Y	no
G2*-004	XX21RECQ L5A-x1047	Fresh	29	20	CI078928	HIN3-170302-01	G	10	c	0.2M magnesium chloride -- 25% PEG3350 -- 0.1M bis-tris pH 5.5	NA		NA	NA	NA	0	NA	17/01/2020 22:51	-	PEG	-	S7N:L12C	D69Y	no
G2*-004	XX21RECQ L5A-x1046	Fresh	29	20	CI078928	HIN3-170302-01	G	12	c	0.2M magnesium chloride -- 25% PEG3350 -- 0.1M HEPES pH 7.5	NA		NA	NA	NA	0	NA	17/01/2020 22:48	-	PEG	-	S7N:L12C	D69Y	no
G2*-004	XX21RECQ L5A-x1045	Fresh	29	20	CI078928	HIN3-170302-01	H	6	a	0.2M sodium formate -- 20% PEG3350	NA		NA	NA	NA	0	NA	17/01/2020 22:45	-	PEG	-	S7N:L12C	D69Y	no

Table S4 Diffraction data

Nanobody grouped ID	Mounted Crystal ID	Xtal Plate Protein Frozen/Fresh?	Xtal Plate Protein Concentration (mg/ml)	Xtal Plate Temperature	Xtal Plate Barcode	Screen Batch Name	XTBM Row	XTBM Column	XTBM Subwell	XTBM Condition	Crystal Form Group	Cystal Form Annotation	Unit Cell Dimensions	Space group	Resolution	Number of Successful Autoprocessing Pipelines	Autoprocessing Pipeline Used	Experiment Time	Soaking condition	Xtal Group	Surface Mutations Around Nbinter-1	Key Mutations	Crystal Epitope Mutations	Condition Repitition?
G2*-004	XX21RECQ L5A-x1041	Fresh	19.37	20	CI078927	HIN3-170302-01	D	6	c	25% PEG3350 -- 0.1M bis-tris pH 5.5	NA		NA	NA	NA	0	NA	17/01/2020 19:48	-	PEG	-	S7N:L12 C	D69Y	no
G2*-004	XX21RECQ L5A-x1039	Fresh	32.3	20	CI078926	HIN3-170302-01	A	12	a	3M sodium chloride -- 0.1M tris pH 8.5	NA		NA	NA	NA	0	NA	17/01/2020 19:41	-	High Salt	-	S7N:L12 C	D69Y	no
G2*-004	XX21RECQ L5A-x1029	Fresh	32.3	20	CI078926	HIN3-170302-01	F	12	a	0.2M sodium chloride -- 25% PEG3350 -- 0.1M HEPES pH 7.5	NA		NA	NA	NA	0	NA	17/01/2020 19:15	-	PEG	-	S7N:L12 C	D69Y	no
G2*-004	XX21RECQ L5A-x1026	Fresh	32.3	20	CI078926	HIN3-170302-01	G	3	c	0.2M lithium sulfate -- 25% PEG3350 -- 0.1M bis-tris pH 6.5	NA		NA	NA	NA	0	NA	17/01/2020 16:29	-	PEG	-	S7N:L12 C	D69Y	no
G2*-004	XX21RECQ L5A-x1024	Fresh	32.3	20	CI078926	HIN3-170302-01	G	9	a	0.2M ammonium acetate -- 25% PEG3350 -- 0.1M tris pH 8.5	NA		NA	NA	NA	0	NA	17/01/2020 16:24	-	PEG	-	S7N:L12 C	D69Y	no
G2*-004	XX21RECQ L5A-x1021	Fresh	32.3	20	CI078926	HIN3-170302-01	G	11	d	0.2M magnesium chloride -- 25% PEG3350 -- 0.1M bis-tris pH 6.5	NA		NA	NA	NA	0	NA	17/01/2020 16:17	-	PEG	-	S7N:L12 C	D69Y	no
G2*-004	XX21RECQ L5A-x1020	Fresh	32.3	20	CI078926	HIN3-170302-01	H	3	d	0.2M sodium malonate -- 20% PEG3350	NA		NA	NA	NA	0	NA	17/01/2020 16:15	-	PEG	-	S7N:L12 C	D69Y	no
G2*-004	XX21RECQ L5A-x1019	Fresh	32.3	20	CI078926	HIN3-170302-01	H	7	c	0.15M DL- malic acid -- 20% PEG3350	NA		NA	NA	NA	0	NA	17/01/2020 16:12	-	PEG	-	S7N:L12 C	D69Y	no
G2*-004	XX21RECQ L5A-x1017	Fresh	24.8	20	CI078929	HIN3-170302-01	F	8	c	0.2M ammonium sulfate -- 25% PEG3350 -- 0.1M HEPES pH 7.5	NA		NA	NA	NA	0	NA	17/01/2020 22:34	-	PEG	-	S7N:L12 C	D69Y	no
G2*-004	XX21RECQ L5A-x1016	Fresh	24.8	20	CI078929	HIN3-170302-01	F	9	c	0.2M ammonium sulfate -- 25% PEG3350 -- 0.1M tris pH 8.5	NA		NA	NA	NA	0	NA	17/01/2020 22:31	-	PEG	-	S7N:L12 C	D69Y	no
G2*-004	XX21RECQ L5A-x1011	Fresh	21.3	20	CI078925	HIN3-170302-01	F	9	a	0.2M ammonium sulfate -- 25% PEG3350 -- 0.1M tris pH 8.5	NA		NA	NA	NA	0	NA	17/01/2020 16:03	-	PEG	-	S7N:L12 C	D69Y	no
G2*-004	XX21RECQ L5A-x1009	Fresh	29.7	20	CI079019	HIN3-170302-01	G	10	c	0.2M magnesium chloride -- 25% PEG3350 -- 0.1M bis-tris pH 5.5	NA		NA	NA	NA	0	NA	17/01/2020 15:59	-	PEG	-	S7N:L12 C	D69Y	no
G2*-004	XX21RECQ L5A-x1003	Fresh	29.7	20	CI079019	HIN3-170302-01	G	5	a	0.2M lithium sulfate -- 25% PEG3350 -- 0.1M tris pH 8.5	NA		NA	NA	NA	0	NA	17/01/2020 17:26	-	PEG	-	S7N:L12 C	D69Y	no
G2*-006	XX21RECQ L5A-x1163	Fresh	30.6	20	CI078938	HIN3-170302-01	G	5	c	0.2M lithium sulfate -- 25% PEG3350 -- 0.1M tris pH 8.5	1	Dimer C 2 2 21	115.13 199.09 174.16 (90.00 90.00 90.00)	C 2 2 21	2.41	7	Xia2-DIALS	17/01/2020 18:56	-	PEG	L12N	S7N	D69Y	no
G2*-006	XX21RECQ L5A-x1151	Fresh	30.6	20	CI078938	HIN3-170302-01	D	6	c	25% PEG3350 -- 0.1M bis-tris pH 5.5	0	Dimer P 1 2 1	74.42 185.13 100.52 (90.0 107.68 90.0)	P 1 21 1	2.57	9	Xia2-DIALS	17/01/2020 18:22	-	PEG	L12N	S7N	D69Y	no

Table S4 Diffraction data

Nanobody grouped ID	Mounted Crystal ID	Xtal Plate Protein Frozen/Fresh?	Xtal Plate Protein Concentration (mg/ml)	Xtal Plate Temperature	Xtal Plate Barcode	Screen Batch Name	XTBM Row	XTBM Column	XTBM Subwell	XTBM Condition	Crystal Form Group	Cystal Form Annotation	Unit Cell Dimensions	Space group	Resolution	Number of Successful Autoprocessing Pipelines	Autoprocessing Pipeline Used	Experiment Time	Soaking condition	Xtal Group	Surface Mutations Around Nbinter-1	Key Mutations	Crystal Epitope Mutations	Condition Repitition?
G2*-006	XX21RECQ L5A-x1155	Fresh	30.6	20	CI078938	HIN3-170302-01	F	6	c	0.2M ammonium sulfate -- 25% PEG3350 -- 0.1M bis-tris pH 5.5	-1	NA	98.48 185.89 151.66 (90.0 107.96 90.0)	P 1 2 1	2.59	7	Xia2-DIALS	17/01/2020 18:34	-	PEG	L12N	S7N	D69Y	no
G2*-006	XX21RECQ L5A-x1171	Fresh	30.6	20	CI078938	HIN3-170302-01	H	3	d	0.2M sodium malonate -- 20% PEG3350	1	Dimer C 2 2 21	115.40 198.40 174.35 (90.00 90.00 90.00)	C 2 2 21	2.61	7	Xia2-DIALS	17/01/2020 17:52	-	PEG	L12N	S7N	D69Y	no
G2*-006	XX21RECQ L5A-x1165	Fresh	30.6	20	CI078938	HIN3-170302-01	G	7	c	0.2M ammonium acetate -- 25% PEG3350 -- 0.1M bis-tris pH 6.5	0	Dimer P 1 2 1	74.7 186.67 99.4 (90.0 107.54 90.0)	P 1 21 1	2.7	9	Xia2-DIALS	17/01/2020 19:03	-	PEG	L12N	S7N	D69Y	no
G2*-006	XX21RECQ L5A-x1164	Fresh	30.6	20	CI078938	HIN3-170302-01	G	5	d	0.2M lithium sulfate -- 25% PEG3350 -- 0.1M tris pH 8.5	1	Dimer C 2 2 21	115.64 199.79 174.37 (90.00 90.00 90.00)	C 2 2 21	2.71	7	Xia2-DIALS	17/01/2020 19:00	-	PEG	L12N	S7N	D69Y	no
G2*-006	XX21RECQ L5A-x1173	Fresh	30.6	20	CI078938	HIN3-170302-01	H	10	a	0.2M sodium citrate tribasic -- 20% PEG3350	1	Dimer C 2 2 21	115.02 198.80 172.99 (90.00 90.00 90.00)	C 2 2 21	2.73	5	Xia2-DIALS	17/01/2020 17:59	-	PEG	L12N	S7N	D69Y	no
G2*-006	XX21RECQ L5A-x1157	Fresh	30.6	20	CI078938	HIN3-170302-01	F	9	c	0.2M ammonium sulfate -- 25% PEG3350 -- 0.1M tris pH 8.5	1	Dimer C 2 2 21	115.15 198.22 174.02 (90.00 90.00 90.00)	C 2 2 21	2.77	7	Xia2-DIALS	17/01/2020 18:39	-	PEG	L12N	S7N	D69Y	no
G2*-006	XX21RECQ L5A-x1168	Fresh	30.6	20	CI078938	HIN3-170302-01	G	11	c	0.2M magnesium chloride -- 25% PEG3350 -- 0.1M bis-tris pH 6.5	0	Dimer P 1 2 1	74.83 185.1 100.24 (90.0 107.84 90.0)	P 1 21 1	2.81	9	Xia2-DIALS	17/01/2020 17:42	-	PEG	L12N	S7N	D69Y	no
G2*-006	XX21RECQ L5A-x1150	Fresh	30.6	20	CI078938	HIN3-170302-01	D	6	a	25% PEG3350 -- 0.1M bis-tris pH 5.5	0	Dimer P 1 2 1	74.98 187.41 100.38 (90.0 107.79 90.0)	P 1 21 1	2.81	9	Xia2-DIALS	17/01/2020 15:51	-	PEG	L12N	S7N	D69Y	no
G2*-006	XX21RECQ L5A-x1158	Fresh	30.6	20	CI078938	HIN3-170302-01	F	10	c	0.2M sodium chloride -- 25% PEG3350 -- 0.1M bis-tris pH 5.5	0	Dimer P 1 2 1	75.17 185.63 99.37 (90.0 107.97 90.0)	P 1 21 1	2.82	9	Xia2-DIALS	17/01/2020 18:41	-	PEG	L12N	S7N	D69Y	no
G2*-006	XX21RECQ L5A-x1160	Fresh	30.6	20	CI078938	HIN3-170302-01	G	3	a	0.2M lithium sulfate -- 25% PEG3350 -- 0.1M bis-tris pH 6.5	0	Dimer P 1 2 1	76.23 173.69 101.26 (90.0 100.18 90.0)	P 1 2 1	2.95	7	Xia2-DIALS	17/01/2020 18:48	-	PEG	L12N	S7N	D69Y	no
G2*-006	XX21RECQ L5A-x1167	Fresh	30.6	20	CI078938	HIN3-170302-01	G	10	c	0.2M magnesium chloride -- 25% PEG3350 -- 0.1M bis-tris pH 5.5	0	Dimer P 1 2 1	74.65 184.45 99.5 (90.0 107.71 90.0)	P 1 21 1	2.97	9	Xia2-DIALS	17/01/2020 17:40	-	PEG	L12N	S7N	D69Y	no
G2*-006	XX21RECQ L5A-x1162	Fresh	30.6	20	CI078938	HIN3-170302-01	G	4	c	0.2M lithium sulfate -- 25% PEG3350 -- 0.1M HEPES pH 7.5	1	Dimer C 2 2 21	114.97 198.61 174.1 (90.0 90.0 90.0)	C 2 2 2	3.09	4	Xia2-DIALS	17/01/2020 18:54	-	PEG	L12N	S7N	D69Y	no
G2*-006	XX21RECQ L5A-x1170	Fresh	30.6	20	CI078938	HIN3-170302-01	H	2	d	0.2M sodium/potassium tartrate -- 20% PEG3350	1	Dimer C 2 2 21	115.72 199.26 173.65 (90.00 90.00 90.00)	C 2 2 21	3.14	7	Xia2-DIALS	17/01/2020 17:47	-	PEG	L12N	S7N	D69Y	no
G2*-006	XX21RECQ L5A-x1172	Fresh	30.6	20	CI078938	HIN3-170302-01	H	6	c	0.2M sodium formate -- 20% PEG3350	1	Dimer C 2 2 21	114.93 198.71 175.39 (90.00 90.00 90.00)	C 2 2 21	3.18	4	Xia2-DIALS	17/01/2020 17:57	-	PEG	L12N	S7N	D69Y	no
G2*-006	XX21RECQ L5A-x1161	Fresh	30.6	20	CI078938	HIN3-170302-01	G	3	c	0.2M lithium sulfate -- 25% PEG3350 -- 0.1M bis-tris pH 6.5	1	Dimer C 2 2 21	115.05 198.34 173.95 (90.0 90.0 90.0)	C 2 2 21	3.21	5	Xia2-DIALS	17/01/2020 18:51	-	PEG	L12N	S7N	D69Y	no

Table S4 Diffraction data

Nanobody grouped ID	Mounted Crystal ID	Xtal Plate Protein Frozen/Fresh?	Xtal Plate Protein Concentration (mg/ml)	Xtal Plate Temperature	Xtal Plate Barcode	Screen Batch Name	XTBM Row	XTBM Column	XTBM Subwell	XTBM Condition	Crystal Form Group	Cystal Form Annotation	Unit Cell Dimensions	Space group	Resolution	Number of Successful Autoprocessing Pipelines	Autoprocessing Pipeline Used	Experiment Time	Soaking condition	Xtal Group	Surface Mutations Around Nbinter-1	Key Mutations	Crystal Epitope Mutations	Condition Repitition?
G2*-006	XX21RECQ L5A-x0387	Fresh	30.6	20	CI078938	HIN3-170302-01	F	12	a	0.2M sodium chloride -- 25% PEG3350 -- 0.1M HEPES pH 7.5	1	Dimer C 2 2 21	114.32 196.9 175.23 (90.0 90.0 90.0)	C 2 2 21	3.23	5	Xia2-DIALS	06/03/2020 08:25	-	PEG	L12N	S7N	D69Y	no
G2*-006	XX21RECQ L5A-x1156	Fresh	30.6	20	CI078938	HIN3-170302-01	F	7	c	0.2M ammonium sulfate -- 25% PEG3350 -- 0.1M bis-tris pH 6.5	1	Dimer C 2 2 21	114.61 197.04 173.11 (90.00 90.00 90.00)	C 2 2 21	3.24	7	Xia2-DIALS	17/01/2020 18:37	-	PEG	L12N	S7N	D69Y	no
G2*-006	XX21RECQ L5A-x1154	Fresh	30.6	20	CI078938	HIN3-170302-01	D	9	c	25% PEG3350 -- 0.1M tris pH 8.5	-1	NA	167.44 315.59 167.4 (90.0 120.08 90.0)	P 1 2 1	3.55	5	Xia2-DIALS	17/01/2020 18:32	-	PEG	L12N	S7N	D69Y	no
G2*-006	XX21RECQ L5A-x1159	Fresh	30.6	20	CI078938	HIN3-170302-01	F	12	c	0.2M sodium chloride -- 25% PEG3350 -- 0.1M HEPES pH 7.5	1	Dimer C 2 2 21	114.39 196.74 173.8 (90.0 90.0 90.0)	C 2 2 21	3.59	1	Xia2-DIALS	17/01/2020 18:44	-	PEG	L12N	S7N	D69Y	no
G2*-006	XX21RECQ L5A-x1169	Fresh	30.6	20	CI078938	HIN3-170302-01	G	12	c	0.2M magnesium chloride -- 25% PEG3350 -- 0.1M HEPES pH 7.5	-1	NA	288.6 166.66 318.81 (90.0 89.98 90.0)	C 1 2 1	3.83	4	Xia2-DIALS	17/01/2020 17:45	-	PEG	L12N	S7N	D69Y	no
G2*-006	XX21RECQ L5A-x1174	Fresh	30.6	20	CI078938	HIN3-170302-01	H	10	c	0.2M sodium citrate tribasic -- 20% PEG3350	1	Dimer C 2 2 21	116.44 200.24 174.08 (90.0 90.0 90.0)	C 2 2 21	3.89	3	Autoproc	17/01/2020 18:03	-	PEG	L12N	S7N	D69Y	no
G2*-006	XX21RECQ L5A-x1166	Fresh	30.6	20	CI078938	HIN3-170302-01	G	8	c	0.2M ammonium acetate -- 25% PEG3350 -- 0.1M HEPES pH 7.5	-1	NA	168.73 168.73 320.26 (90.0 90.0 120.0)	P 6 2 2	4.08	3	Xia2-DIALS	17/01/2020 19:06	-	PEG	L12N	S7N	D69Y	no
G2*-006	XX21RECQ L5A-x0386	Fresh	30.6	20	CI078938	HIN3-170302-01	H	11	c	0.1M potassium thiocyanate -- 30% PEG2000MME	0	Dimer P 1 2 1	73.55 182.29 99.2 (90.0 107.52 90.0)	P 1 2 1	4.1	1	Xia2-DIALS	06/03/2020 08:23	-	PEG	L12N	S7N	D69Y	no
G2*-006	XX21RECQ L5A-x1153	Fresh	30.6	20	CI078938	HIN3-170302-01	D	8	c	25% PEG3350 -- 0.1M HEPES pH 7.5	-1	NA	167.8 167.87 315.77 (90.04 89.96 60.03)	P 1	5.31	3	Xia2-DIALS	17/01/2020 18:30	-	PEG	L12N	S7N	D69Y	no
G2*-006	XX21RECQ L5A-x1152	Fresh	30.6	20	CI078938	HIN3-170302-01	D	7	c	25% PEG3350 -- 0.1M bis-tris pH 6.5	NA		NA	NA	NA	0	NA	17/01/2020 18:24	-	PEG	L12N	S7N	D69Y	no
G2*-006	XX21RECQ L5A-x1149	Fresh	30.6	20	CI078938	HIN3-170302-01	C	5	a	60% tacsimate	NA		NA	NA	NA	0	NA	17/01/2020 15:49	-	High Salt	L12N	S7N	D69Y	no
G2*-008	XX21RECQ L5A-x0390	Fresh	28.5	20	CI078975	HIN3-170302-01	C	9	c	1.1M sodium malonate -- 0.5% jeffamine ED-2003 -- 0.1M HEPES pH 7.0	2	Tetramer P 2 2 21 Porous	89.72 151.73 266.6 (90.0 90.0 90.0)	P 2 2 2	3.87	2	Xia2-DIALS	06/03/2020 08:32	-	High Salt	Q14K	S7N:L12C	D69Y	no
G2*-008	XX21RECQ L5A-x0389	Fresh	28.5	20	CI078975	HIN3-170302-01	C	9	c	1.1M sodium malonate -- 0.5% jeffamine ED-2003 -- 0.1M HEPES pH 7.0	-1	NA	89.72 145.38 270.02 (89.95 90.02 89.77)	P 1	3.9	1	Xia2-DIALS	06/03/2020 08:29	-	High Salt	Q14K	S7N:L12C	D69Y	no
G2*-008	XX21RECQ L5A-x1179	Fresh	28.5	20	CI078975	HIN3-170302-01	B	7	c	0.056M sodium phosphate monobasic -- 1.344M potassium phosphate dibasic	2	Tetramer P 2 2 21 Porous	90.24 152.4 271.2 (90.02 90.03 89.97)	P 21 21 2	5.07	5	Xia2-DIALS	17/01/2020 18:15	-	High Salt	Q14K	S7N:L12C	D69Y	no
G2*-008	XX21RECQ L5A-x0394	Fresh	28.5	20	CI078975	HIN3-170302-01	C	10	d	1M succinic acid -- 1% PEG2000MME -- 0.1M HEPES pH 7.0	-1	NA	89.82 147.68 270.5 (90.03 89.98 90.21)	P 1	5.35	1	Xia2-DIALS	06/03/2020 08:41	-	High Salt	Q14K	S7N:L12C	D69Y	no

Table S4 Diffraction data

Nanobody grouped ID	Mounted Crystal ID	Xtal Plate Protein Frozen/Fresh?	Xtal Plate Protein Concentration (mg/ml)	Xtal Plate Temperature	Xtal Plate Barcode	Screen Batch Name	XTBM Row	XTBM Column	XTBM Subwell	XTBM Condition	Crystal Form Group	Cystal Form Annotation	Unit Cell Dimensions	Space group	Resolution	Number of Successful Autoprocessing Pipelines	Autoprocessing Pipeline Used	Experiment Time	Soaking condition	Xtal Group	Surface Mutations Around Nbinter-1	Key Mutations	Crystal Epitope Mutations	Condition Repitition?
G2*-008	XX21RECQ L5A-x0392	Fresh	28.5	20	CI078975	HIN3-170302-01	C	10	d	1M succinic acid -- 1% PEG2000MME -- 0.1M HEPES pH 7.0	-1	NA	306.64 89.43 145.52 (90.0 118.33 90.0)	C 1 2 1	NA	1	NA	06/03/2020 08:36	-	High Salt	Q14K	S7N:L12C	D69Y	no
G2*-008	XX21RECQ L5A-x0393	Fresh	28.5	20	CI078975	HIN3-170302-01	C	10	d	1M succinic acid -- 1% PEG2000MME -- 0.1M HEPES pH 7.0	NA		NA	NA	NA	0	NA	06/03/2020 08:38	-	High Salt	Q14K	S7N:L12C	D69Y	no
G2*-008	XX21RECQ L5A-x0391	Fresh	28.5	20	CI078975	HIN3-170302-01	C	9	c	1.1M sodium malonate -- 0.5% jeffamine ED-2003 -- 0.1M HEPES pH 7.0	NA		NA	NA	NA	0	NA	06/03/2020 08:34	-	High Salt	Q14K	S7N:L12C	D69Y	no
G2*-008	XX21RECQ L5A-x0388	Fresh	28.5	20	CI078975	HIN3-170302-01	C	9	c	1.1M sodium malonate -- 0.5% jeffamine ED-2003 -- 0.1M HEPES pH 7.0	NA		NA	NA	NA	0	NA	06/03/2020 08:27	-	High Salt	Q14K	S7N:L12C	D69Y	no
G2*-011	XX21RECQ L5A-x1178	Fresh	9.54	20	CI078977	HIN3-170302-01	G	4	c	0.2M lithium sulfate -- 25% PEG3350 -- 0.1M HEPES pH 7.5	1	Dimer C 2 2 21	114.88 198.84 173.02 (90.0 90.0 90.0)	C 2 2 21	3.04	4	Xia2-DIALS	17/01/2020 18:12	-	PEG	R20K	S7N:L12C	D69Y	no
G2*-014	XX21RECQ L5A-x1180	Fresh	9	20	CI078979	HIN3-170302-01	G	5	c	0.2M lithium sulfate -- 25% PEG3350 -- 0.1M tris pH 8.5	NA		NA	NA	NA	0	NA	17/01/2020 18:18	-	PEG	Q90H	S7N:L12C	D69Y	no
G2*-015	XX21RECQ L5A-x1057	Fresh	18.36	20	CI078930	HIN3-170302-01	H	3	c	0.2M sodium malonate -- 20% PEG3350	1	Dimer C 2 2 21	115.90 199.69 173.86 (90.00 90.00 90.00)	C 2 2 21	3.05	5	Xia2-DIALS	17/01/2020 21:35	-	PEG	N92D	S7N:L12C	D69Y	no
G2*-015	XX21RECQ L5A-x1063	Fresh	18.36	20	CI078930	HIN3-170302-01	D	6	c	25% PEG3350 -- 0.1M bis-tris pH 5.5	0	Dimer P 1 2 1	74.81 186.63 100.11 (90.0 107.49 90.0)	P 1 21 1	3.15	7	Xia2-DIALS	17/01/2020 21:55	-	PEG	N92D	S7N:L12C	D69Y	no
G2*-015	XX21RECQ L5A-x1059	Fresh	18.36	20	CI078930	HIN3-170302-01	G	5	a	0.2M lithium sulfate -- 25% PEG3350 -- 0.1M tris pH 8.5	1	Dimer C 2 2 21	115.79 200.7 174.91 (90.0 90.0 90.0)	C 2 2 21	3.23	4	Xia2-DIALS	17/01/2020 21:42	-	PEG	N92D	S7N:L12C	D69Y	no
G2*-015	XX21RECQ L5A-x1061	Fresh	18.36	20	CI078930	HIN3-170302-01	F	9	c	0.2M ammonium sulfate -- 25% PEG3350 -- 0.1M tris pH 8.5	1	Dimer C 2 2 21	115.30 199.04 172.81 (90.00 90.00 90.00)	C 2 2 21	3.24	5	Xia2-DIALS	17/01/2020 21:48	-	PEG	N92D	S7N:L12C	D69Y	no
G2*-015	XX21RECQ L5A-x0395	Fresh	18.36	20	CI078930	HIN3-170302-01	D	7	c	25% PEG3350 -- 0.1M bis-tris pH 6.5	0	Dimer P 1 2 1	75.25 185.96 99.57 (90.0 107.93 90.0)	P 1 21 1	3.42	3	Xia2-DIALS	06/03/2020 08:43	-	PEG	N92D	S7N:L12C	D69Y	no
G2*-015	XX21RECQ L5A-x1060	Fresh	18.36	20	CI078930	HIN3-170302-01	G	4	c	0.2M lithium sulfate -- 25% PEG3350 -- 0.1M HEPES pH 7.5	1	Dimer C 2 2 21	114.77 198.83 172.75 (90.00 90.00 90.00)	C 2 2 21	3.69	3	Xia2-DIALS	17/01/2020 21:46	-	PEG	N92D	S7N:L12C	D69Y	no
G2*-015	XX21RECQ L5A-x1058	Fresh	18.36	20	CI078930	HIN3-170302-01	G	5	c	0.2M lithium sulfate -- 25% PEG3350 -- 0.1M tris pH 8.5	-1	NA	205.3 117.85 229.93 (90.0 125.36 90.0)	C 1 2 1	3.84	2	Xia2-DIALS	17/01/2020 21:39	-	PEG	N92D	S7N:L12C	D69Y	no
G2*-015	XX21RECQ L5A-x1062	Fresh	18.36	20	CI078930	HIN3-170302-01	F	8	c	0.2M ammonium sulfate -- 25% PEG3350 -- 0.1M HEPES pH 7.5	1	Dimer C 2 2 21	115.82 199.44 173.37 (90.00 90.00 90.00)	C 2 2 2	3.92	5	Xia2-DIALS	17/01/2020 21:52	-	PEG	N92D	S7N:L12C	D69Y	no
G2*-015	XX21RECQ L5A-x1056	Fresh	18.36	20	CI078930	HIN3-170302-01	H	10	c	0.2M sodium citrate tribasic -- 20% PEG3350	NA		NA	NA	NA	0	NA	17/01/2020 21:32	-	PEG	N92D	S7N:L12C	D69Y	no

Table S4 Diffraction data

Nanobody grouped ID	Mounted Crystal ID	Xtal Plate Protein Frozen/Fresh?	Xtal Plate Protein Concentration (mg/ml)	Xtal Plate Temperature	Xtal Plate Barcode	Screen Batch Name	XTBM Row	XTBM Column	XTBM Subwell	XTBM Condition	Crystal Form Group	Cystal Form Annotation	Unit Cell Dimensions	Space group	Resolution	Number of Successful Autoprocessing Pipelines	Autoprocessing Pipeline Used	Experiment Time	Soaking condition	Xtal Group	Surface Mutations Around Nbinter-1	Key Mutations	Crystal Epitope Mutations	Condition Repitition?
G2*-016	XX21RECQ L5A-x1072	Fresh	22.5	20	CI078931	HIN3-170302-01	F	8	c	0.2M ammonium sulfate -- 25% PEG3350 -- 0.1M HEPES pH 7.5	1	Dimer C 2 2 21	115.32 198.54 174.83 (90.00 90.00 90.00)	C 2 2 21	2.45	5	Xia2-DIALS	17/01/2020 20:43	-	PEG	-	S7N:L12C	D69Y	no
G2*-016	XX21RECQ L5A-x1071	Fresh	22.5	20	CI078931	HIN3-170302-01	F	9	a	0.2M ammonium sulfate -- 25% PEG3350 -- 0.1M tris pH 8.5	1	Dimer C 2 2 21	115.88 199.40 174.53 (90.00 90.00 90.00)	C 2 2 21	2.96	5	Xia2-DIALS	17/01/2020 20:40	-	PEG	-	S7N:L12C	D69Y	no
G2*-016	XX21RECQ L5A-x1068	Fresh	22.5	20	CI078931	HIN3-170302-01	G	10	c	0.2M magnesium chloride -- 25% PEG3350 -- 0.1M bis-tris pH 5.5	0	Dimer P 1 2 1	74.9 184.55 99.82 (90.0 107.63 90.0)	P 1 21 1	2.98	9	Xia2-DIALS	17/01/2020 22:12	-	PEG	-	S7N:L12C	D69Y	no
G2*-016	XX21RECQ L5A-x1064	Fresh	22.5	20	CI078931	HIN3-170302-01	H	10	c	0.2M sodium citrate tribasic -- 20% PEG3350	1	Dimer C 2 2 21	115.52 199.67 174.58 (90.00 90.00 90.00)	C 2 2 21	2.98	5	Xia2-DIALS	17/01/2020 22:01	-	PEG	-	S7N:L12C	D69Y	no
G2*-016	XX21RECQ L5A-x1069	Fresh	22.5	20	CI078931	HIN3-170302-01	G	5	c	0.2M lithium sulfate -- 25% PEG3350 -- 0.1M tris pH 8.5	1	Dimer C 2 2 21	114.93 198.55 173.91 (90.0 90.0 90.0)	C 2 2 21	3.05	5	Xia2-DIALS	17/01/2020 22:17	-	PEG	-	S7N:L12C	D69Y	no
G2*-016	XX21RECQ L5A-x1065	Fresh	22.5	20	CI078931	HIN3-170302-01	H	10	a	0.2M sodium citrate tribasic -- 20% PEG3350	1	Dimer C 2 2 21	115.08 198.75 173.09 (90.0 89.98 90.0)	C 2 2 21	3.2	5	Xia2-DIALS	17/01/2020 22:04	-	PEG	-	S7N:L12C	D69Y	no
G2*-016	XX21RECQ L5A-x1067	Fresh	22.5	20	CI078931	HIN3-170302-01	H	3	c	0.2M sodium malonate -- 20% PEG3350	1	Dimer C 2 2 21	115.51 198.91 174.55 (90.0 90.0 90.0)	C 2 2 21	3.27	4	Xia2-DIALS	17/01/2020 22:09	-	PEG	-	S7N:L12C	D69Y	no
G2*-016	XX21RECQ L5A-x1073	Fresh	22.5	20	CI078931	HIN3-170302-01	F	8	a	0.2M ammonium sulfate -- 25% PEG3350 -- 0.1M HEPES pH 7.5	1	Dimer C 2 2 21	115.53 198.88 173.25 (90.0 90.0 90.0)	C 2 2 21	3.35	5	Xia2-DIALS	17/01/2020 20:46	-	PEG	-	S7N:L12C	D69Y	no
G2*-016	XX21RECQ L5A-x1070	Fresh	22.5	20	CI078931	HIN3-170302-01	G	5	a	0.2M lithium sulfate -- 25% PEG3350 -- 0.1M tris pH 8.5	1	Dimer C 2 2 21	115.44 198.82 172.52 (90.0 90.0 90.0)	C 2 2 21	3.56	5	Xia2-DIALS	17/01/2020 22:22	-	PEG	-	S7N:L12C	D69Y	no
G2*-016	XX21RECQ L5A-x1066	Fresh	22.5	20	CI078931	HIN3-170302-01	H	6	a	0.2M sodium formate -- 20% PEG3350	1	Dimer C 2 2 21	115.04 199.29 174.86 (90.0 90.0 90.0)	C 2 2 21	3.67	4	Xia2-DIALS	17/01/2020 22:06	-	PEG	-	S7N:L12C	D69Y	no
G2-001	XX11RECQ L5A-x033	Fresh	10.63	20	CI072128	HIN3-170302-01	F	12	c	0.2M sodium chloride -- 25% PEG3350 -- 0.1M HEPES pH 7.5	0	Dimer P 1 2 1	75.9 183.33 101.08 (90.0 107.88 90.0)	P 1 21 1	2.56	5	Xia2-DIALS	28/02/2019 06:23	-	PEG	-	S7N:L12C	D69Y	no
G2-001	XX11RECQ L5A-x0782	Fresh	12.6	20	CI079584	HIN3-170302-01	F	12	c	0.2M sodium chloride -- 25% PEG3350 -- 0.1M HEPES pH 7.5	0	Dimer P 1 2 1	75.40 181.59 100.74(90.00 107.82 90.00)	P 1 2 1	2.61	7	Xia2-DIALS	07/10/2020 16:17	0% DMSO - 15% Glycerol	PEG	-	S7N:L12C	D69Y	no
G2-001	XX11RECQ L5A-x0720	Fresh	12.6	20	CI079584	HIN3-170302-01	F	12	c	0.2M sodium chloride -- 25% PEG3350 -- 0.1M HEPES pH 7.5	0	Dimer P 1 2 1	74.66 184.45 102.47(90.00 107.97 90.00)	P 1 21 1	2.62	9	Xia2-DIALS	07/10/2020 17:18	0% DMSO - 15% EG	PEG	-	S7N:L12C	D69Y	no
G2-001	XX11RECQ L5A-x0767	Fresh	12.6	20	CI079584	HIN3-170302-01	F	12	c	0.2M sodium chloride -- 25% PEG3350 -- 0.1M HEPES pH 7.5	0	Dimer P 1 2 1	75.12 183.72 101.72(90.00 107.92 90.00)	P 1 21 1	2.64	7	Xia2-DIALS	07/10/2020 19:43	30% DMSO - 20% EG	PEG	-	S7N:L12C	D69Y	no
G2-001	XX11RECQ L5A-x027	Fresh	10.63	20	CI072128	HIN3-170302-01	F	10	a	0.2M sodium chloride -- 25% PEG3350 -- 0.1M bis-tris pH 5.5	0	Dimer P 1 2 1	75.75 184.28 102.04 (90.0 107.97 90.0)	P 1 21 1	2.65	5	Xia2-DIALS	21/10/2019 18:44	-	PEG	-	S7N:L12C	D69Y	no

Table S4 Diffraction data

Nanobody grouped ID	Mounted Crystal ID	Xtal Plate Protein Frozen/Fresh?	Xtal Plate Protein Concentration (mg/ml)	Xtal Plate Temperature	Xtal Plate Barcode	Screen Batch Name	XTBM Row	XTBM Column	XTBM Subwell	XTBM Condition	Crystal Form Group	Cystal Form Annotation	Unit Cell Dimensions	Space group	Resolution	Number of Successful Autoprocessing Pipelines	Autoprocessing Pipeline Used	Experiment Time	Soaking condition	Xtal Group	Surface Mutations Around Nbinter-1	Key Mutations	Crystal Epitope Mutations	Condition Repitition?
G2-001	XX11RECQ L5A-x0762	Fresh	12.6	20	CI079584	HIN3-170302-01	F	12	c	0.2M sodium chloride -- 25% PEG3350 -- 0.1M HEPES pH 7.5	0	Dimer P 1 2 1	74.89 183.98 102.20(90.00 107.98 90.00)	P 1 2 1	2.66	9	Xia2-DIALS	07/10/2020 19:30	30% DMSO - 10% EG	PEG	-	S7N:L12 C	D69Y	no
G2-001	XX11RECQ L5A-x0741	Fresh	12.6	20	CI079584	HIN3-170302-01	F	12	c	0.2M sodium chloride -- 25% PEG3350 -- 0.1M HEPES pH 7.5	0	Dimer P 1 2 1	75.08 184.30 102.11(90.00 108.01 90.00)	P 1 21 1	2.67	9	Xia2-DIALS	07/10/2020 18:33	10% DMSO - 15% Sucrose	PEG	-	S7N:L12 C	D69Y	no
G2-001	XX11RECQ L5A-x0751	Fresh	12.6	20	CI079584	HIN3-170302-01	F	12	c	0.2M sodium chloride -- 25% PEG3350 -- 0.1M HEPES pH 7.5	0	Dimer P 1 2 1	75.65 188.23 102.01(90.00 107.86 90.00)	P 1 21 1	2.69	3	Xia2-DIALS	07/10/2020 18:59	20% DMSO - 15% EG	PEG	-	S7N:L12 C	D69Y	no
G2-001	XX11RECQ L5A-x0761	Fresh	12.6	20	CI079584	HIN3-170302-01	F	12	c	0.2M sodium chloride -- 25% PEG3350 -- 0.1M HEPES pH 7.5	0	Dimer P 1 2 1	75.55 185.82 102.27(90.00 108.07 90.00)	P 1 21 1	2.69	9	Xia2-DIALS	07/10/2020 19:28	30% DMSO - 10% EG	PEG	-	S7N:L12 C	D69Y	no
G2-001	XX11RECQ L5A-x0766	Fresh	12.6	20	CI079584	HIN3-170302-01	F	12	c	0.2M sodium chloride -- 25% PEG3350 -- 0.1M HEPES pH 7.5	0	Dimer P 1 2 1	75.28 183.71 101.60(90.00 107.90 90.00)	P 1 21 1	2.69	9	Xia2-DIALS	07/10/2020 19:40	30% DMSO - 15% EG	PEG	-	S7N:L12 C	D69Y	no
G2-001	XX11RECQ L5A-x0768	Fresh	12.6	20	CI079584	HIN3-170302-01	F	12	c	0.2M sodium chloride -- 25% PEG3350 -- 0.1M HEPES pH 7.5	0	Dimer P 1 2 1	75.31 182.97 101.28(90.00 108.01 90.00)	P 1 21 1	2.71	9	Xia2-DIALS	07/10/2020 19:45	30% DMSO - 20% EG	PEG	-	S7N:L12 C	D69Y	no
G2-001	XX11RECQ L5A-x0774	Fresh	12.6	20	CI079584	HIN3-170302-01	F	12	c	0.2M sodium chloride -- 25% PEG3350 -- 0.1M HEPES pH 7.5	0	Dimer P 1 2 1	76.06 181.55 100.47(90.00 108.00 90.00)	P 1 21 1	2.74	9	Xia2-DIALS	07/10/2020 20:01	0% DMSO - 20% Sucrose	PEG	-	S7N:L12 C	D69Y	no
G2-001	XX11RECQ L5A-x031	Fresh	10.63	20	CI072128	HIN3-170302-01	F	11	d	0.2M sodium chloride -- 25% PEG3350 -- 0.1M bis-tris pH 6.5	0	Dimer P 1 2 1	75.12 184.88 102.0 (90.0 107.89 90.0)	P 1 21 1	2.83	5	Xia2-DIALS	21/10/2019 18:57	-	PEG	-	S7N:L12 C	D69Y	no
G2-001	XX11RECQ L5A-x0759	Fresh	12.6	20	CI079584	HIN3-170302-01	F	12	c	0.2M sodium chloride -- 25% PEG3350 -- 0.1M HEPES pH 7.5	0	Dimer P 1 2 1	76.02 187.44 102.00(90.00 107.96 90.00)	P 1 21 1	2.86	7	Xia2-DIALS	07/10/2020 19:21	30% DMSO - 0% EG	PEG	-	S7N:L12 C	D69Y	no
G2-001	XX11RECQ L5A-x0771	Fresh	12.6	20	CI079584	HIN3-170302-01	F	12	c	0.2M sodium chloride -- 25% PEG3350 -- 0.1M HEPES pH 7.5	0	Dimer P 1 2 1	75.33 183.55 101.36(90.00 107.99 90.00)	P 1 2 1	2.86	9	Xia2-DIALS	07/10/2020 19:53	30% DMSO - 15% Sucrose	PEG	-	S7N:L12 C	D69Y	no
G2-001	XX11RECQ L5A-x0773	Fresh	12.6	20	CI079584	HIN3-170302-01	F	12	c	0.2M sodium chloride -- 25% PEG3350 -- 0.1M HEPES pH 7.5	0	Dimer P 1 2 1	75.61 182.42 101.16(90.00 107.92 90.00)	P 1 21 1	2.88	9	Xia2-DIALS	07/10/2020 19:59	0% DMSO - 20% Sucrose	PEG	-	S7N:L12 C	D69Y	no
G2-001	XX11RECQ L5A-x0721	Fresh	12.6	20	CI079584	HIN3-170302-01	F	12	c	0.2M sodium chloride -- 25% PEG3350 -- 0.1M HEPES pH 7.5	0	Dimer P 1 2 1	75.17 182.94 101.82(90.00 108.05 90.00)	P 1 21 1	2.89	9	Xia2-DIALS	07/10/2020 17:21	0% DMSO - 15% EG	PEG	-	S7N:L12 C	D69Y	no
G2-001	XX11RECQ L5A-x0783	Fresh	12.6	20	CI079584	HIN3-170302-01	F	12	c	0.2M sodium chloride -- 25% PEG3350 -- 0.1M HEPES pH 7.5	0	Dimer P 1 2 1	75.68 179.70 100.20(90.00 108.22 90.00)	P 1 2 1	2.91	11	Xia2-DIALS	07/10/2020 16:20	0% DMSO - 15% Glycerol	PEG	-	S7N:L12 C	D69Y	no
G2-001	XX11RECQ L5A-x0719	Fresh	12.6	20	CI079584	HIN3-170302-01	F	12	c	0.2M sodium chloride -- 25% PEG3350 -- 0.1M HEPES pH 7.5	0	Dimer P 1 2 1	75.13 184.65 102.09(90.00 107.97 90.00)	P 1 21 1	2.93	9	Xia2-DIALS	07/10/2020 17:16	0% DMSO - 15% EG	PEG	-	S7N:L12 C	D69Y	no
G2-001	XX11RECQ L5A-x0758	Fresh	12.6	20	CI079584	HIN3-170302-01	F	12	c	0.2M sodium chloride -- 25% PEG3350 -- 0.1M HEPES pH 7.5	0	Dimer P 1 2 1	75.22 183.58 101.24(90.00 108.05 90.00)	P 1 21 1	2.93	6	Xia2-DIALS	07/10/2020 19:19	30% DMSO - 0% EG	PEG	-	S7N:L12 C	D69Y	no

Table S4 Diffraction data

Nanobody grouped ID	Mounted Crystal ID	Xtal Plate Protein Frozen/Fresh?	Xtal Plate Protein Concentration (mg/ml)	Xtal Plate Temperature	Xtal Plate Barcode	Screen Batch Name	XTBM Row	XTBM Column	XTBM Subwell	XTBM Condition	Crystal Form Group	Cystal Form Annotation	Unit Cell Dimensions	Space group	Resolution	Number of Successful Autoprocessing Pipelines	Autoprocessing Pipeline Used	Experiment Time	Soaking condition	Xtal Group	Surface Mutations Around Nbinter-1	Key Mutations	Crystal Epitope Mutations	Condition Repitition?
G2-001	XX11RECQ L5A-x0743	Fresh	12.6	20	CI079584	HIN3-170302-01	F	12	c	0.2M sodium chloride -- 25% PEG3350 -- 0.1M HEPES pH 7.5	0	Dimer P 1 2 1	75.44 102.96 187.19(89.76 89.85 72.04)	P 1 21 1	2.95	9	Xia2-DIALS	07/10/2020 18:38	20% DMSO - 0% EG	PEG	-	S7N:L12 C	D69Y	no
G2-001	XX11RECQ L5A-x0755	Fresh	12.6	20	CI079584	HIN3-170302-01	F	12	c	0.2M sodium chloride -- 25% PEG3350 -- 0.1M HEPES pH 7.5	0	Dimer P 1 2 1	75.39 184.98 102.74(90.00 108.10 90.00)	P 1 2 1	2.97	9	Xia2-DIALS	07/10/2020 19:11	20% DMSO - 15% Sucrose	PEG	-	S7N:L12 C	D69Y	no
G2-001	XX11RECQ L5A-x0729	Fresh	12.6	20	CI079584	HIN3-170302-01	F	12	c	0.2M sodium chloride -- 25% PEG3350 -- 0.1M HEPES pH 7.5	0	Dimer P 1 2 1	75.60 102.58 184.72(90.00 89.92 108.11)	P 1 21 1	2.98	9	Xia2-DIALS	07/10/2020 18:01	10% DMSO - 0% EG	PEG	-	S7N:L12 C	D69Y	no
G2-001	XX11RECQ L5A-x0772	Fresh	12.6	20	CI079584	HIN3-170302-01	F	12	c	0.2M sodium chloride -- 25% PEG3350 -- 0.1M HEPES pH 7.5	0	Dimer P 1 2 1	75.16 183.01 101.20(90.00 107.96 90.00)	P 1 21 1	2.99	9	Xia2-DIALS	07/10/2020 19:56	30% DMSO - 15% Sucrose	PEG	-	S7N:L12 C	D69Y	no
G2-001	XX11RECQ L5A-x002	Fresh	10.63	20	CI072128	HIN3-170302-01	D	3	c	30% jeffamine ED-2003 -- 0.1M HEPES pH 7.0	0	Dimer P 1 2 1	75.5 184.89 101.77 (90.0 107.94 90.0)	P 1 21 1	3	5	Xia2-DIALS	28/02/2019 06:33	-	PEG	-	S7N:L12 C	D69Y	no
G2-001	XX11RECQ L5A-x0750	Fresh	12.6	20	CI079584	HIN3-170302-01	F	12	c	0.2M sodium chloride -- 25% PEG3350 -- 0.1M HEPES pH 7.5	0	Dimer P 1 2 1	77.59 193.18 104.67(90.00 108.17 90.00)	P 1 21 1	3.01	6	Xia2-DIALS	07/10/2020 18:57	20% DMSO - 15% EG	PEG	-	S7N:L12 C	D69Y	no
G2-001	XX11RECQ L5A-x0718	Fresh	12.6	20	CI079584	HIN3-170302-01	F	12	c	0.2M sodium chloride -- 25% PEG3350 -- 0.1M HEPES pH 7.5	0	Dimer P 1 2 1	74.98 184.97 102.73(90.00 107.98 90.00)	P 1 2 1	3.03	9	Xia2-DIALS	07/10/2020 17:13	0% DMSO - 10% EG	PEG	-	S7N:L12 C	D69Y	no
G2-001	XX11RECQ L5A-x0777	Fresh	12.6	20	CI079584	HIN3-170302-01	F	12	c	0.2M sodium chloride -- 25% PEG3350 -- 0.1M HEPES pH 7.5	0	Dimer P 1 2 1	75.57 181.12 100.18(90.00 107.75 90.00)	P 1 21 1	3.03	9	Xia2-DIALS	07/10/2020 16:04	0% DMSO - 25% Sucrose	PEG	-	S7N:L12 C	D69Y	no
G2-001	XX11RECQ L5A-x0728	Fresh	12.6	20	CI079584	HIN3-170302-01	F	12	c	0.2M sodium chloride -- 25% PEG3350 -- 0.1M HEPES pH 7.5	0	Dimer P 1 2 1	74.83 184.74 102.89(90.00 108.17 90.00)	P 1 21 1	3.04	9	Xia2-DIALS	07/10/2020 17:58	10% DMSO - 0% EG	PEG	-	S7N:L12 C	D69Y	no
G2-001	XX11RECQ L5A-x0757	Fresh	12.6	20	CI079584	HIN3-170302-01	F	12	c	0.2M sodium chloride -- 25% PEG3350 -- 0.1M HEPES pH 7.5	0	Dimer P 1 2 1	75.38 184.75 101.96(90.00 107.99 90.00)	P 1 2 1	3.07	8	Xia2-DIALS	07/10/2020 19:16	20% DMSO - 15% Sucrose	PEG	-	S7N:L12 C	D69Y	no
G2-001	XX11RECQ L5A-x0714	Fresh	12.6	20	CI079584	HIN3-170302-01	F	12	c	0.2M sodium chloride -- 25% PEG3350 -- 0.1M HEPES pH 7.5	0	Dimer P 1 2 1	75.16 185.32 102.71(90.00 108.20 90.00)	P 1 21 1	3.12	9	Xia2-DIALS	07/10/2020 17:03	0% DMSO - 0% EG	PEG	-	S7N:L12 C	D69Y	no
G2-001	XX11RECQ L5A-x0778	Fresh	12.6	20	CI079584	HIN3-170302-01	F	12	c	0.2M sodium chloride -- 25% PEG3350 -- 0.1M HEPES pH 7.5	0	Dimer P 1 2 1	75.56 183.90 101.47(90.00 107.94 90.00)	P 1 21 1	3.12	11	Xia2-DIALS	07/10/2020 16:06	0% DMSO - 25% Sucrose	PEG	-	S7N:L12 C	D69Y	no
G2-001	XX11RECQ L5A-x0730	Fresh	12.6	20	CI079584	HIN3-170302-01	F	12	c	0.2M sodium chloride -- 25% PEG3350 -- 0.1M HEPES pH 7.5	0	Dimer P 1 2 1	75.03 184.91 101.94(90.00 107.75 90.00)	P 1 21 1	3.12	9	Xia2-DIALS	07/10/2020 18:03	10% DMSO - 0% EG	PEG	-	S7N:L12 C	D69Y	no
G2-001	XX11RECQ L5A-x025	Fresh	10.63	20	CI072128	HIN3-170302-01	F	8	d	0.2M ammonium sulfate -- 25% PEG3350 -- 0.1M HEPES pH 7.5	0	Dimer P 1 2 1	75.62 180.84 100.49 (90.0 107.9 90.0)	P 1 21 1	3.13	5	Xia2-DIALS	21/10/2019 18:37	-	PEG	-	S7N:L12 C	D69Y	no
G2-001	XX11RECQ L5A-x0747	Fresh	12.6	20	CI079584	HIN3-170302-01	F	12	c	0.2M sodium chloride -- 25% PEG3350 -- 0.1M HEPES pH 7.5	0	Dimer P 1 2 1	75.00 184.68 102.76(90.00 108.09 90.00)	P 1 21 1	3.17	9	Xia2-DIALS	07/10/2020 18:49	20% DMSO - 10% EG	PEG	-	S7N:L12 C	D69Y	no

Table S4 Diffraction data

Nanobody grouped ID	Mounted Crystal ID	Xtal Plate Protein Frozen/Fresh?	Xtal Plate Protein Concentration (mg/ml)	Xtal Plate Temperature	Xtal Plate Barcode	Screen Batch Name	XTBM Row	XTBM Column	XTBM Subwell	XTBM Condition	Crystal Form Group	Cystal Form Annotation	Unit Cell Dimensions	Space group	Resolution	Number of Successful Autoprocessing Pipelines	Autoprocessing Pipeline Used	Experiment Time	Soaking condition	Xtal Group	Surface Mutations Around Nbinter-1	Key Mutations	Crystal Epitope Mutations	Condition Repitition?
G2-001	XX11RECQ L5A-x0715	Fresh	12.6	20	CI079584	HIN3-170302-01	F	12	c	0.2M sodium chloride -- 25% PEG3350 -- 0.1M HEPES pH 7.5	0	Dimer P 1 2 1	75.49 186.80 101.99(90.00 107.91 90.00)	P 1 21 1	3.2	9	Xia2-DIALS	07/10/2020 17:05	0% DMSO - 0% EG	PEG	-	S7N:L12 C	D69Y	no
G2-001	XX11RECQ L5A-x0716	Fresh	12.6	20	CI079584	HIN3-170302-01	F	12	c	0.2M sodium chloride -- 25% PEG3350 -- 0.1M HEPES pH 7.5	0	Dimer P 1 2 1	74.82 184.74 102.66(90.00 107.95 90.00)	P 1 21 1	3.2	9	Xia2-DIALS	07/10/2020 17:08	0% DMSO - 10% EG	PEG	-	S7N:L12 C	D69Y	no
G2-001	XX11RECQ L5A-x0781	Fresh	12.6	20	CI079584	HIN3-170302-01	F	12	c	0.2M sodium chloride -- 25% PEG3350 -- 0.1M HEPES pH 7.5	0	Dimer P 1 2 1	71.13 79.69 85.88(62.25 86.61 75.31)	P 1 21 1	3.2	9	Xia2-DIALS	07/10/2020 16:14	0% DMSO - 10% Glycerol	PEG	-	S7N:L12 C	D69Y	no
G2-001	XX11RECQ L5A-x026	Fresh	10.63	20	CI072128	HIN3-170302-01	F	9	a	0.2M ammonium sulfate -- 25% PEG3350 -- 0.1M tris pH 8.5	0	Dimer P 1 2 1	75.51 184.09 102.32 (90.0 108.04 90.0)	P 1 21 1	3.21	5	Xia2-DIALS	21/10/2019 18:41	-	PEG	-	S7N:L12 C	D69Y	no
G2-001	XX11RECQ L5A-x0744	Fresh	12.6	20	CI079584	HIN3-170302-01	F	12	c	0.2M sodium chloride -- 25% PEG3350 -- 0.1M HEPES pH 7.5	0	Dimer P 1 2 1	74.97 184.53 102.93(90.00 108.08 90.00)	P 1 21 1	3.23	9	Xia2-DIALS	07/10/2020 18:41	20% DMSO - 0% EG	PEG	-	S7N:L12 C	D69Y	no
G2-001	XX11RECQ L5A-x0763	Fresh	12.6	20	CI079584	HIN3-170302-01	F	12	c	0.2M sodium chloride -- 25% PEG3350 -- 0.1M HEPES pH 7.5	0	Dimer P 1 2 1	75.55 185.03 102.35(90.00 108.13 90.00)	P 1 2 1	3.26	9	Xia2-DIALS	07/10/2020 19:33	30% DMSO - 10% EG	PEG	-	S7N:L12 C	D69Y	no
G2-001	XX11RECQ L5A-x0738	Fresh	12.6	20	CI079584	HIN3-170302-01	F	12	c	0.2M sodium chloride -- 25% PEG3350 -- 0.1M HEPES pH 7.5	0	Dimer P 1 2 1	75.19 185.39 103.12(90.00 107.84 90.00)	P 1 21 1	3.27	9	Xia2-DIALS	07/10/2020 18:24	10% DMSO - 20% EG	PEG	-	S7N:L12 C	D69Y	no
G2-001	XX11RECQ L5A-x0756	Fresh	12.6	20	CI079584	HIN3-170302-01	F	12	c	0.2M sodium chloride -- 25% PEG3350 -- 0.1M HEPES pH 7.5	0	Dimer P 1 2 1	74.86 184.64 102.62(90.00 107.98 90.00)	P 1 21 1	3.27	9	Xia2-DIALS	07/10/2020 19:14	20% DMSO - 15% Sucrose	PEG	-	S7N:L12 C	D69Y	no
G2-001	XX11RECQ L5A-x0722	Fresh	12.6	20	CI079584	HIN3-170302-01	F	12	c	0.2M sodium chloride -- 25% PEG3350 -- 0.1M HEPES pH 7.5	0	Dimer P 1 2 1	75.52 183.32 101.80(90.00 108.07 90.00)	P 1 21 1	3.34	9	Xia2-DIALS	07/10/2020 17:23	0% DMSO - 20% EG	PEG	-	S7N:L12 C	D69Y	no
G2-001	XX11RECQ L5A-x023	Fresh	10.63	20	CI072128	HIN3-170302-01	F	8	c	0.2M ammonium sulfate -- 25% PEG3350 -- 0.1M HEPES pH 7.5	0	Dimer P 1 2 1	76.12 183.27 101.68 (90.0 108.12 90.0)	P 1 21 1	3.38	5	Xia2-DIALS	21/10/2019 18:31	-	PEG	-	S7N:L12 C	D69Y	no
G2-001	XX11RECQ L5A-x017	Fresh	10.63	20	CI072128	HIN3-170302-01	F	5	a	0.1M ammonium acetate -- 17%(w/v) PEG10000 -- 0.1M bis-tris pH 5.5	0	Dimer P 1 2 1	75.59 183.31 101.21 (90.0 107.75 90.0)	P 1 2 1	3.38	4	Xia2-DIALS	21/10/2019 18:06	-	PEG	-	S7N:L12 C	D69Y	no
G2-001	XX11RECQ L5A-x0779	Fresh	12.6	20	CI079584	HIN3-170302-01	F	12	c	0.2M sodium chloride -- 25% PEG3350 -- 0.1M HEPES pH 7.5	0	Dimer P 1 2 1	75.78 181.16 100.98(90.00 108.32 90.00)	P 1 21 1	3.41	8	Xia2-DIALS	07/10/2020 16:09	0% DMSO - 10% Glycerol	PEG	-	S7N:L12 C	D69Y	no
G2-001	XX11RECQ L5A-x032	Fresh	10.63	20	CI072128	HIN3-170302-01	F	12	a	0.2M sodium chloride -- 25% PEG3350 -- 0.1M HEPES pH 7.5	0	Dimer P 1 2 1	75.95 182.39 100.45 (90.0 107.87 90.0)	P 1 21 1	3.47	5	Xia2-DIALS	28/02/2019 06:21	-	PEG	-	S7N:L12 C	D69Y	no
G2-001	XX11RECQ L5A-x0717	Fresh	12.6	20	CI079584	HIN3-170302-01	F	12	c	0.2M sodium chloride -- 25% PEG3350 -- 0.1M HEPES pH 7.5	0	Dimer P 1 2 1	75.51 185.52 102.56(90.00 108.15 90.00)	P 1 21 1	3.47	8	Xia2-DIALS	07/10/2020 17:10	0% DMSO - 10% EG	PEG	-	S7N:L12 C	D69Y	no
G2-001	XX11RECQ L5A-x0769	Fresh	12.6	20	CI079584	HIN3-170302-01	F	12	c	0.2M sodium chloride -- 25% PEG3350 -- 0.1M HEPES pH 7.5	0	Dimer P 1 2 1	76.86 183.71 101.09(90.00 108.11 90.00)	P 1 21 1	3.49	3	Xia2-DIALS	07/10/2020 19:48	30% DMSO - 20% EG	PEG	-	S7N:L12 C	D69Y	no

Table S4 Diffraction data

Nanobody grouped ID	Mounted Crystal ID	Xtal Plate Protein Frozen/Fresh?	Xtal Plate Protein Concentration (mg/ml)	Xtal Plate Temperature	Xtal Plate Barcode	Screen Batch Name	XTBM Row	XTBM Column	XTBM Subwell	XTBM Condition	Crystal Form Group	Cystal Form Annotation	Unit Cell Dimensions	Space group	Resolution	Number of Successful Autoprocessing Pipelines	Autoprocessing Pipeline Used	Experiment Time	Soaking condition	Xtal Group	Surface Mutations Around Nbinter-1	Key Mutations	Crystal Epitope Mutations	Condition Repitition?
G2-001	XX11RECQ L5A-x0726	Fresh	12.6	20	CI079584	HIN3-170302-01	F	12	c	0.2M sodium chloride -- 25% PEG3350 -- 0.1M HEPES pH 7.5	0	Dimer P 1 2 1	75.17 185.13 102.90(90.00 108.19 90.00)	P 1 21 1	3.51	8	Xia2-DIALS	07/10/2020 17:34	0% DMSO - 15% Sucrose	PEG	-	S7N:L12C	D69Y	no
G2-001	XX11RECQ L5A-x0754	Fresh	12.6	20	CI079584	HIN3-170302-01	F	12	c	0.2M sodium chloride -- 25% PEG3350 -- 0.1M HEPES pH 7.5	0	Dimer P 1 2 1	74.88 185.34 102.55(90.00 107.96 90.00)	P 1 21 1	3.51	9	Xia2-DIALS	07/10/2020 19:08	20% DMSO - 20% EG	PEG	-	S7N:L12C	D69Y	no
G2-001	XX11RECQ L5A-x030	Fresh	10.63	20	CI072128	HIN3-170302-01	F	11	c	0.2M sodium chloride -- 25% PEG3350 -- 0.1M bis-tris pH 6.5	0	Dimer P 1 2 1	76.25 185.67 100.97 (90.0 107.82 90.0)	P 1 21 1	3.52	5	Xia2-DIALS	21/10/2019 18:54	-	PEG	-	S7N:L12C	D69Y	no
G2-001	XX11RECQ L5A-x0748	Fresh	12.6	20	CI079584	HIN3-170302-01	F	12	c	0.2M sodium chloride -- 25% PEG3350 -- 0.1M HEPES pH 7.5	0	Dimer P 1 2 1	75.23 102.90 184.54(90.01 90.02 71.82)	P 1 21 1	3.53	9	Xia2-DIALS	07/10/2020 18:52	20% DMSO - 10% EG	PEG	-	S7N:L12C	D69Y	no
G2-001	XX11RECQ L5A-x0740	Fresh	12.6	20	CI079584	HIN3-170302-01	F	12	c	0.2M sodium chloride -- 25% PEG3350 -- 0.1M HEPES pH 7.5	0	Dimer P 1 2 1	74.98 184.89 102.17(90.00 107.94 90.00)	P 1 21 1	3.54	9	Xia2-DIALS	07/10/2020 18:30	10% DMSO - 15% Sucrose	PEG	-	S7N:L12C	D69Y	no
G2-001	XX11RECQ L5A-x0725	Fresh	12.6	20	CI079584	HIN3-170302-01	F	12	c	0.2M sodium chloride -- 25% PEG3350 -- 0.1M HEPES pH 7.5	0	Dimer P 1 2 1	76.09 188.10 102.66(90.00 107.83 90.00)	P 1 2 1	3.55	9	Xia2-DIALS	07/10/2020 17:31	0% DMSO - 15% Sucrose	PEG	-	S7N:L12C	D69Y	no
G2-001	XX11RECQ L5A-x029	Fresh	10.63	20	CI072128	HIN3-170302-01	F	11	a	0.2M sodium chloride -- 25% PEG3350 -- 0.1M bis-tris pH 6.5	0	Dimer P 1 2 1	77.38 185.8 101.21 (90.0 108.04 90.0)	P 1 21 1	3.56	5	Xia2-DIALS	21/10/2019 18:50	-	PEG	-	S7N:L12C	D69Y	no
G2-001	XX11RECQ L5A-x0775	Fresh	12.6	20	CI079584	HIN3-170302-01	F	12	c	0.2M sodium chloride -- 25% PEG3350 -- 0.1M HEPES pH 7.5	0	Dimer P 1 2 1	75.71 181.54 100.25(90.00 108.23 90.00)	P 1 21 1	3.59	9	Xia2-DIALS	07/10/2020 20:04	0% DMSO - 20% Sucrose	PEG	-	S7N:L12C	D69Y	no
G2-001	XX11RECQ L5A-x0731	Fresh	12.6	20	CI079584	HIN3-170302-01	F	12	c	0.2M sodium chloride -- 25% PEG3350 -- 0.1M HEPES pH 7.5	0	Dimer P 1 2 1	75.30 183.90 102.35(90.00 108.20 90.00)	P 1 2 1	3.6	9	Xia2-DIALS	07/10/2020 18:06	10% DMSO - 10% EG	PEG	-	S7N:L12C	D69Y	no
G2-001	XX11RECQ L5A-x024	Fresh	10.63	20	CI072128	HIN3-170302-01	F	8	d	0.2M ammonium sulfate -- 25% PEG3350 -- 0.1M HEPES pH 7.5	0	Dimer P 1 2 1	75.84 183.98 101.47 (90.0 107.74 90.0)	P 1 21 1	3.63	3	Xia2-3dii	21/10/2019 18:34	-	PEG	-	S7N:L12C	D69Y	no
G2-001	XX11RECQ L5A-x0713	Fresh	12.6	20	CI079584	HIN3-170302-01	F	12	c	0.2M sodium chloride -- 25% PEG3350 -- 0.1M HEPES pH 7.5	0	Dimer P 1 2 1	74.64 184.37 102.41(90.00 108.17 90.00)	P 1 2 1	3.7	9	Xia2-DIALS	07/10/2020 17:00	0% DMSO - 0% EG	PEG	-	S7N:L12C	D69Y	no
G2-001	XX11RECQ L5A-x0753	Fresh	12.6	20	CI079584	HIN3-170302-01	F	12	c	0.2M sodium chloride -- 25% PEG3350 -- 0.1M HEPES pH 7.5	0	Dimer P 1 2 1	75.65 186.25 102.57(90.00 108.05 90.00)	P 1 21 1	3.76	9	Xia2-DIALS	07/10/2020 19:05	20% DMSO - 20% EG	PEG	-	S7N:L12C	D69Y	no
G2-001	XX11RECQ L5A-x0770	Fresh	12.6	20	CI079584	HIN3-170302-01	F	12	c	0.2M sodium chloride -- 25% PEG3350 -- 0.1M HEPES pH 7.5	0	Dimer P 1 2 1	76.45 185.43 101.55(90.00 108.00 90.00)	P 1 21 1	3.77	6	Xia2-3dii	07/10/2020 19:50	30% DMSO - 15% Sucrose	PEG	-	S7N:L12C	D69Y	no
G2-001	XX11RECQ L5A-x0776	Fresh	12.6	20	CI079584	HIN3-170302-01	F	12	c	0.2M sodium chloride -- 25% PEG3350 -- 0.1M HEPES pH 7.5	0	Dimer P 1 2 1	76.49 184.00 101.67(90.00 108.14 90.00)	P 1 21 1	3.78	5	Xia2-DIALS	07/10/2020 20:06	0% DMSO - 25% Sucrose	PEG	-	S7N:L12C	D69Y	no
G2-001	XX11RECQ L5A-x0780	Fresh	12.6	20	CI079584	HIN3-170302-01	F	12	c	0.2M sodium chloride -- 25% PEG3350 -- 0.1M HEPES pH 7.5	0	Dimer P 1 2 1	75.10 181.91 100.70(90.00 107.85 90.00)	P 1 21 1	3.79	7	Xia2-DIALS	07/10/2020 16:12	0% DMSO - 10% Glycerol	PEG	-	S7N:L12C	D69Y	no

Table S4 Diffraction data

Nanobody grouped ID	Mounted Crystal ID	Xtal Plate Protein Frozen/Fresh?	Xtal Plate Protein Concentration (mg/ml)	Xtal Plate Temperature	Xtal Plate Barcode	Screen Batch Name	XTBM Row	XTBM Column	XTBM Subwell	XTBM Condition	Crystal Form Group	Cystal Form Annotation	Unit Cell Dimensions	Space group	Resolution	Number of Successful Autoprocessing Pipelines	Autoprocessing Pipeline Used	Experiment Time	Soaking condition	Xtal Group	Surface Mutations Around Nbinter-1	Key Mutations	Crystal Epitope Mutations	Condition Repitition?
G2-001	XX11RECQ L5A-x0745	Fresh	12.6	20	CI079584	HIN3-170302-01	F	12	c	0.2M sodium chloride -- 25% PEG3350 -- 0.1M HEPES pH 7.5	0	Dimer P 1 2 1	74.73 184.24 102.87(90.00 108.23 90.00)	P 1 2 1	3.92	7	Xia2-DIALS	07/10/2020 18:44	20% DMSO - 0% EG	PEG	-	S7N:L12 C	D69Y	no
G2-001	XX11RECQ L5A-x0732	Fresh	12.6	20	CI079584	HIN3-170302-01	F	12	c	0.2M sodium chloride -- 25% PEG3350 -- 0.1M HEPES pH 7.5	0	Dimer P 1 2 1	75.19 183.90 101.83(90.00 107.94 90.00)	P 1 21 1	3.95	9	Xia2-DIALS	07/10/2020 18:09	10% DMSO - 10% EG	PEG	-	S7N:L12 C	D69Y	no
G2-001	XX11RECQ L5A-x0760	Fresh	12.6	20	CI079584	HIN3-170302-01	F	12	c	0.2M sodium chloride -- 25% PEG3350 -- 0.1M HEPES pH 7.5	0	Dimer P 1 2 1	75.24 185.12 102.66(90.00 107.98 90.00)	P 1 2 1	3.95	9	Xia2-DIALS	07/10/2020 19:25	30% DMSO - 0% EG	PEG	-	S7N:L12 C	D69Y	no
G2-001	XX11RECQ L5A-x0737	Fresh	12.6	20	CI079584	HIN3-170302-01	F	12	c	0.2M sodium chloride -- 25% PEG3350 -- 0.1M HEPES pH 7.5	0	Dimer P 1 2 1	75.07 185.02 102.94(90.00 108.05 90.00)	P 1 2 1	4.11	9	Xia2-DIALS	07/10/2020 18:22	10% DMSO - 20% EG	PEG	-	S7N:L12 C	D69Y	no
G2-001	XX11RECQ L5A-x0749	Fresh	12.6	20	CI079584	HIN3-170302-01	F	12	c	0.2M sodium chloride -- 25% PEG3350 -- 0.1M HEPES pH 7.5	0	Dimer P 1 2 1	75.24 182.99 101.61(90.00 108.10 90.00)	P 1 21 1	4.17	9	Xia2-DIALS	07/10/2020 18:54	20% DMSO - 15% EG	PEG	-	S7N:L12 C	D69Y	no
G2-001	XX11RECQ L5A-x032	Fresh	10.63	20	CI072128	HIN3-170302-01	F	12	a	0.2M sodium chloride -- 25% PEG3350 -- 0.1M HEPES pH 7.5	0	Dimer P 1 2 1	75.51 181.55 100.76 (90.0 107.82 90.0)	P 1 21 1	4.29	5	Xia2-DIALS	21/10/2019 19:00	-	PEG	-	S7N:L12 C	D69Y	no
G2-001	XX11RECQ L5A-x0739	Fresh	12.6	20	CI079584	HIN3-170302-01	F	12	c	0.2M sodium chloride -- 25% PEG3350 -- 0.1M HEPES pH 7.5	0	Dimer P 1 2 1	75.39 184.41 102.26(90.00 108.26 90.00)	P 1 21 1	4.29	9	Xia2-DIALS	07/10/2020 18:27	10% DMSO - 20% EG	PEG	-	S7N:L12 C	D69Y	no
G2-001	XX11RECQ L5A-x0735	Fresh	12.6	20	CI079584	HIN3-170302-01	F	12	c	0.2M sodium chloride -- 25% PEG3350 -- 0.1M HEPES pH 7.5	0	Dimer P 1 2 1	75.55 185.72 102.58(90.00 108.20 90.00)	P 1 21 1	4.36	9	Xia2-DIALS	07/10/2020 18:16	10% DMSO - 15% EG	PEG	-	S7N:L12 C	D69Y	no
G2-001	XX11RECQ L5A-x0752	Fresh	12.6	20	CI079584	HIN3-170302-01	F	12	c	0.2M sodium chloride -- 25% PEG3350 -- 0.1M HEPES pH 7.5	0	Dimer P 1 2 1	75.23 185.92 102.65(90.00 108.03 90.00)	P 1 2 1	4.49	9	Xia2-DIALS	07/10/2020 19:02	20% DMSO - 20% EG	PEG	-	S7N:L12 C	D69Y	no
G2-001	XX11RECQ L5A-x0736	Fresh	12.6	20	CI079584	HIN3-170302-01	F	12	c	0.2M sodium chloride -- 25% PEG3350 -- 0.1M HEPES pH 7.5	0	Dimer P 1 2 1	75.33 185.87 102.49(90.00 107.97 90.00)	P 1 21 1	4.71	7	Xia2-DIALS	07/10/2020 18:19	10% DMSO - 15% EG	PEG	-	S7N:L12 C	D69Y	no
G2-001	XX11RECQ L5A-x020	Fresh	10.63	20	CI072128	HIN3-170302-01	F	7	c	0.2M ammonium sulfate -- 25% PEG3350 -- 0.1M bis-tris pH 6.5	0	Dimer P 1 2 1	75.87 183.81 101.58 (90.0 107.65 90.0)	P 1 21 1	4.73	1	Xia2-DIALS	21/10/2019 18:16	-	PEG	-	S7N:L12 C	D69Y	no
G2-001	XX11RECQ L5A-x0746	Fresh	12.6	20	CI079584	HIN3-170302-01	F	12	c	0.2M sodium chloride -- 25% PEG3350 -- 0.1M HEPES pH 7.5	0	Dimer P 1 2 1	75.54 184.96 102.71(90.00 108.27 90.00)	P 1 21 1	4.78	9	Xia2-DIALS	07/10/2020 18:46	20% DMSO - 10% EG	PEG	-	S7N:L12 C	D69Y	no
G2-001	XX11RECQ L5A-x019	Fresh	10.63	20	CI072128	HIN3-170302-01	F	7	a	0.2M ammonium sulfate -- 25% PEG3350 -- 0.1M bis-tris pH 6.5	0	Dimer P 1 2 1	75.34 184.45 101.55 (90.0 107.87 90.0)	P 1 21 1	4.83	3	Xia2-DIALS	21/10/2019 18:13	-	PEG	-	S7N:L12 C	D69Y	no
G2-001	XX11RECQ L5A-x0724	Fresh	12.6	20	CI079584	HIN3-170302-01	F	12	c	0.2M sodium chloride -- 25% PEG3350 -- 0.1M HEPES pH 7.5	0	Dimer P 1 2 1	75.44 102.60 187.01(90.18 89.92 71.88)	P 1	4.89	9	Xia2-DIALS	07/10/2020 17:29	0% DMSO - 20% EG	PEG	-	S7N:L12 C	D69Y	no
G2-001	XX11RECQ L5A-x028	Fresh	10.63	20	CI072128	HIN3-170302-01	F	10	c	0.2M sodium chloride -- 25% PEG3350 -- 0.1M bis-tris pH 5.5	0	Dimer P 1 2 1	75.56 183.68 100.6 (90.0 107.51 90.0)	P 1 21 1	5.14	4	Xia2-DIALS	21/10/2019 18:47	-	PEG	-	S7N:L12 C	D69Y	no

Table S4 Diffraction data

Nanobody grouped ID	Mounted Crystal ID	Xtal Plate Protein Frozen/Fresh?	Xtal Plate Protein Concentration (mg/ml)	Xtal Plate Temperature	Xtal Plate Barcode	Screen Batch Name	XTBM Row	XTBM Column	XTBM Subwell	XTBM Condition	Crystal Form Group	Cystal Form Annotation	Unit Cell Dimensions	Space group	Resolution	Number of Successful Autoprocessing Pipelines	Autoprocessing Pipeline Used	Experiment Time	Soaking condition	Xtal Group	Surface Mutations Around Nbinter-1	Key Mutations	Crystal Epitope Mutations	Condition Repitition?
G2-001	XX11RECQ L5A-x0733	Fresh	12.6	20	CI079584	HIN3-170302-01	F	12	c	0.2M sodium chloride -- 25% PEG3350 -- 0.1M HEPES pH 7.5	0	Dimer P 1 2 1	76.58 186.12 102.01(90.00 108.04 90.00)	P 1 21 1	5.2	8	Xia2-DIALS	07/10/2020 18:11	10% DMSO - 10% EG	PEG	-	S7N:L12C	D69Y	no
G2-001	XX11RECQ L5A-x0723	Fresh	12.6	20	CI079584	HIN3-170302-01	F	12	c	0.2M sodium chloride -- 25% PEG3350 -- 0.1M HEPES pH 7.5	0	Dimer P 1 2 1	75.30 186.32 104.17(90.00 108.21 90.00)	P 1 21 1	5.37	3	Autoproc	07/10/2020 17:26	0% DMSO - 20% EG	PEG	-	S7N:L12C	D69Y	no
G2-001	XX11RECQ L5A-x0727	Fresh	12.6	20	CI079584	HIN3-170302-01	F	12	c	0.2M sodium chloride -- 25% PEG3350 -- 0.1M HEPES pH 7.5	0	Dimer P 1 2 1	75.73 185.74 103.03(90.00 108.23 90.00)	P 1 2 1	5.47	4	Fast_dp	07/10/2020 17:54	0% DMSO - 15% Sucrose	PEG	-	S7N:L12C	D69Y	no
G2-001	XX11RECQ L5A-x0734	Fresh	12.6	20	CI079584	HIN3-170302-01	F	12	c	0.2M sodium chloride -- 25% PEG3350 -- 0.1M HEPES pH 7.5	0	Dimer P 1 2 1	73.07 180.52 98.32(90.00 107.77 90.00)	P 1 2 1	6.61	4	Xia2-3dii	07/10/2020 18:14	10% DMSO - 15% EG	PEG	-	S7N:L12C	D69Y	no
G2-001	XX11RECQ L5A-x022	Fresh	10.63	20	CI072128	HIN3-170302-01	F	8	a	0.2M ammonium sulfate -- 25% PEG3350 -- 0.1M HEPES pH 7.5	NA		NA	NA	NA	0	NA	21/10/2019 18:22	-	PEG	-	S7N:L12C	D69Y	no
G2-001	XX11RECQ L5A-x021	Fresh	10.63	20	CI072128	HIN3-170302-01	F	7	d	0.2M ammonium sulfate -- 25% PEG3350 -- 0.1M bis-tris pH 6.5	NA		NA	NA	NA	0	NA	21/10/2019 18:19	-	PEG	-	S7N:L12C	D69Y	no
G2-001	XX11RECQ L5A-x018	Fresh	10.63	20	CI072128	HIN3-170302-01	F	6	a	0.2M ammonium sulfate -- 25% PEG3350 -- 0.1M bis-tris pH 5.5	NA		NA	NA	NA	0	NA	21/10/2019 18:10	-	PEG	-	S7N:L12C	D69Y	no
G2-001	XX11RECQ L5A-x0742	Fresh	12.6	20	CI079584	HIN3-170302-01	F	12	c	0.2M sodium chloride -- 25% PEG3350 -- 0.1M HEPES pH 7.5	NA	NA	NA	NA	NA	0	NA	07/10/2020 18:36	10% DMSO - 15% Sucrose	PEG	-	S7N:L12C	D69Y	no
G2-001	XX11RECQ L5A-x0765	Fresh	12.6	20	CI079584	HIN3-170302-01	F	12	c	0.2M sodium chloride -- 25% PEG3350 -- 0.1M HEPES pH 7.5	NA	NA	NA	NA	NA	0	NA	07/10/2020 19:38	30% DMSO - 15% EG	PEG	-	S7N:L12C	D69Y	no
G2-001	XX11RECQ L5A-x0764	Fresh	12.6	20	CI079584	HIN3-170302-01	F	12	c	0.2M sodium chloride -- 25% PEG3350 -- 0.1M HEPES pH 7.5	NA	NA	NA	NA	NA	0	NA	07/10/2020 19:35	30% DMSO - 15% EG	PEG	-	S7N:L12C	D69Y	no
G2-003	XX21RECQ L5A-x1091	Fresh	30.9	20	CI078932	HIN3-170302-01	G	6	d	0.2M ammonium acetate -- 25% PEG3350 -- 0.1M bis-tris pH 5.5	0	Dimer P 1 2 1	75.17 187.08 100.62 (90.0 107.7 90.0)	P 1 2 1	2.37	9	Xia2-DIALS	17/01/2020 20:05	-	PEG	L12N	S7N	D69Y	no
G2-003	XX21RECQ L5A-x1090	Fresh	30.9	20	CI078932	HIN3-170302-01	G	7	a	0.2M ammonium acetate -- 25% PEG3350 -- 0.1M bis-tris pH 6.5	0	Dimer P 1 2 1	75.88 189.55 100.85 (90.0 107.92 90.0)	P 1 2 1	2.64	9	Xia2-DIALS	17/01/2020 20:03	-	PEG	L12N	S7N	D69Y	no
G2-003	XX21RECQ L5A-x1110	Fresh	30.9	20	CI078932	HIN3-170302-01	D	11	a	28% PEG2000MME -- 0.1M bis-tris pH 6.5	0	Dimer P 1 2 1	75.08 183.39 100.4 (90.0 107.8 90.0)	P 1 21 1	2.7	9	Xia2-DIALS	17/01/2020 23:34	-	PEG	L12N	S7N	D69Y	no
G2-003	XX21RECQ L5A-x1097	Fresh	30.9	20	CI078932	HIN3-170302-01	F	12	c	0.2M sodium chloride -- 25% PEG3350 -- 0.1M HEPES pH 7.5	0	Dimer P 1 2 1	75.59 183.18 100.86 (90.0 108.07 90.0)	P 1 2 1	2.7	9	Xia2-DIALS	17/01/2020 20:21	-	PEG	L12N	S7N	D69Y	no
G2-003	XX21RECQ L5A-x1116	Fresh	30.9	20	CI078932	HIN3-170302-01	D	6	c	25% PEG3350 -- 0.1M bis-tris pH 5.5	0	Dimer P 1 2 1	75.54 185.62 101.09 (90.0 107.88 90.0)	P 1 21 1	2.71	9	Xia2-DIALS	17/01/2020 23:52	-	PEG	L12N	S7N	D69Y	no

Table S4 Diffraction data

Nanobody grouped ID	Mounted Crystal ID	Xtal Plate Protein Frozen/Fresh?	Xtal Plate Protein Concentration (mg/ml)	Xtal Plate Temperature	Xtal Plate Barcode	Screen Batch Name	XTBM Row	XTBM Column	XTBM Subwell	XTBM Condition	Crystal Form Group	Cystal Form Annotation	Unit Cell Dimensions	Space group	Resolution	Number of Successful Autoprocessing Pipelines	Autoprocessing Pipeline Used	Experiment Time	Soaking condition	Xtal Group	Surface Mutations Around Nbinter-1	Key Mutations	Crystal Epitope Mutations	Condition Repitition?
G2-003	XX21RECQ L5A-x1083	Fresh	30.9	20	CI078932	HIN3-170302-01	G	11	a	0.2M magnesium chloride -- 25% PEG3350 -- 0.1M bis-tris pH 6.5	0	Dimer P 1 2 1	75.87 185.61 100.82 (90.0 108.05 90.0)	P 1 21 1	2.76	9	Xia2-DIALS	17/01/2020 21:15	-	PEG	L12N	S7N	D69Y	no
G2-003	XX21RECQ L5A-x1075	Fresh	30.9	20	CI078932	HIN3-170302-01	H	8	d	0.1M magnesium formate -- 15% PEG3350	0	Dimer P 1 2 1	77.01 189.3 101.15 (90.0 108.67 90.0)	P 1 21 1	2.78	8	Xia2-DIALS	17/01/2020 20:51	-	PEG	L12N	S7N	D69Y	no
G2-003	XX21RECQ L5A-x1114	Fresh	30.9	20	CI078932	HIN3-170302-01	D	8	a	25% PEG3350 -- 0.1M HEPES pH 7.5	0	Dimer P 1 2 1	76.26 184.62 100.53 (90.0 108.18 90.0)	P 1 21 1	2.79	7	Xia2-DIALS	17/01/2020 23:47	-	PEG	L12N	S7N	D69Y	no
G2-003	XX21RECQ L5A-x1086	Fresh	30.9	20	CI078932	HIN3-170302-01	G	9	a	0.2M ammonium acetate -- 25% PEG3350 -- 0.1M tris pH 8.5	1	Dimer C 2 2 21	114.93 199.49 174.51 (90.00 90.00 90.00)	C 2 2 21	2.82	5	Xia2-DIALS	17/01/2020 21:24	-	PEG	L12N	S7N	D69Y	no
G2-003	XX21RECQ L5A-x1112	Fresh	30.9	20	CI078932	HIN3-170302-01	D	9	a	25% PEG3350 -- 0.1M tris pH 8.5	0	Dimer P 1 2 1	76.41 182.64 100.41 (90.0 108.42 90.0)	P 1 21 1	2.89	9	Xia2-DIALS	17/01/2020 23:42	-	PEG	L12N	S7N	D69Y	no
G2-003	XX21RECQ L5A-x1101	Fresh	30.9	20	CI078932	HIN3-170302-01	F	10	c	0.2M sodium chloride -- 25% PEG3350 -- 0.1M bis-tris pH 5.5	0	Dimer P 1 2 1	75.42 183.9 100.9 (90.0 108.02 90.0)	P 1 21 1	2.89	9	Xia2-DIALS	18/01/2020 08:41	-	PEG	L12N	S7N	D69Y	no
G2-003	XX21RECQ L5A-x1089	Fresh	30.9	20	CI078932	HIN3-170302-01	G	7	c	0.2M ammonium acetate -- 25% PEG3350 -- 0.1M bis-tris pH 6.5	0	Dimer P 1 2 1	75.43 186.42 100.38 (90.0 107.82 90.0)	P 1 21 1	2.89	9	Xia2-DIALS	17/01/2020 20:00	-	PEG	L12N	S7N	D69Y	no
G2-003	XX21RECQ L5A-x1087	Fresh	30.9	20	CI078932	HIN3-170302-01	G	8	c	0.2M ammonium acetate -- 25% PEG3350 -- 0.1M HEPES pH 7.5	0	Dimer P 1 2 1	75.78 184.41 100.43 (90.0 107.8 90.0)	P 1 21 1	2.89	7	Xia2-DIALS	17/01/2020 19:56	-	PEG	L12N	S7N	D69Y	no
G2-003	XX21RECQ L5A-x1092	Fresh	30.9	20	CI078932	HIN3-170302-01	G	6	c	0.2M ammonium acetate -- 25% PEG3350 -- 0.1M bis-tris pH 5.5	0	Dimer P 1 2 1	75.25 185.84 100.19 (90.0 107.68 90.0)	P 1 21 1	2.91	9	Xia2-DIALS	17/01/2020 20:07	-	PEG	L12N	S7N	D69Y	no
G2-003	XX21RECQ L5A-x1078	Fresh	30.9	20	CI078932	HIN3-170302-01	H	5	d	0.1M succinic acid -- 15% PEG3350	0	Dimer P 1 2 1	76.95 187.64 100.23 (90.0 108.48 90.0)	P 1 21 1	2.91	8	Xia2-DIALS	17/01/2020 21:00	-	PEG	L12N	S7N	D69Y	no
G2-003	XX21RECQ L5A-x1107	Fresh	30.9	20	CI078932	HIN3-170302-01	F	6	a	0.2M ammonium sulfate -- 25% PEG3350 -- 0.1M bis-tris pH 5.5	0	Dimer P 1 2 1	76.64 187.21 98.86 (90.0 108.01 90.0)	P 1 2 1	2.93	9	Xia2-DIALS	17/01/2020 23:27	-	PEG	L12N	S7N	D69Y	no
G2-003	XX21RECQ L5A-x1099	Fresh	30.9	20	CI078932	HIN3-170302-01	F	11	c	0.2M sodium chloride -- 25% PEG3350 -- 0.1M bis-tris pH 6.5	0	Dimer P 1 2 1	75.77 186.52 100.85 (90.0 108.11 90.0)	P 1 2 1	2.94	8	Xia2-DIALS	17/01/2020 20:28	-	PEG	L12N	S7N	D69Y	no
G2-003	XX21RECQ L5A-x1106	Fresh	30.9	20	CI078932	HIN3-170302-01	F	6	c	0.2M ammonium sulfate -- 25% PEG3350 -- 0.1M bis-tris pH 5.5	0	Dimer P 1 2 1	75.86 184.19 99.06 (90.0 107.55 90.0)	P 1 21 1	2.96	9	Xia2-DIALS	17/01/2020 23:25	-	PEG	L12N	S7N	D69Y	no
G2-003	XX21RECQ L5A-x1088	Fresh	30.9	20	CI078932	HIN3-170302-01	G	7	d	0.2M ammonium acetate -- 25% PEG3350 -- 0.1M bis-tris pH 6.5	0	Dimer P 1 2 1	75.13 186.21 100.27 (90.0 107.71 90.0)	P 1 21 1	2.96	9	Xia2-DIALS	17/01/2020 19:58	-	PEG	L12N	S7N	D69Y	no
G2-003	XX21RECQ L5A-x1079	Fresh	30.9	20	CI078932	HIN3-170302-01	H	3	c	0.2M sodium malonate -- 20% PEG3350	-1	NA	173.91 199.17 116.07 (90.0 90.0 90.0)	P 21 21 2	2.98	5	Xia2-DIALS	17/01/2020 21:02	-	PEG	L12N	S7N	D69Y	no

Table S4 Diffraction data

Nanobody grouped ID	Mounted Crystal ID	Xtal Plate Protein Frozen/Fresh?	Xtal Plate Protein Concentration (mg/ml)	Xtal Plate Temperature	Xtal Plate Barcode	Screen Batch Name	XTBM Row	XTBM Column	XTBM Subwell	XTBM Condition	Crystal Form Group	Cystal Form Annotation	Unit Cell Dimensions	Space group	Resolution	Number of Successful Autoprocessing Pipelines	Autoprocessing Pipeline Used	Experiment Time	Soaking condition	Xtal Group	Surface Mutations Around Nbinter-1	Key Mutations	Crystal Epitope Mutations	Condition Repitition?
G2-003	XX21RECQ L5A-x1085	Fresh	30.9	20	CI078932	HIN3-170302-01	G	10	a	0.2M magnesium chloride -- 25% PEG3350 -- 0.1M bis-tris pH 5.5	0	Dimer P 1 2 1	74.68 181.92 99.77 (90.0 107.51 90.0)	P 1 21 1	2.99	9	Xia2-DIALS	17/01/2020 21:22	-	PEG	L12N	S7N	D69Y	no
G2-003	XX21RECQ L5A-x1108	Fresh	30.9	20	CI078932	HIN3-170302-01	F	5	c	0.1M ammonium acetate -- 17%(w/v) PEG10000 -- 0.1M bis-tris pH 5.5	0	Dimer P 1 2 1	76.13 191.0 101.25 (90.0 108.2 90.0)	P 1 2 1	3.06	8	Xia2-DIALS	17/01/2020 23:29	-	PEG	L12N	S7N	D69Y	no
G2-003	XX21RECQ L5A-x1093	Fresh	30.9	20	CI078932	HIN3-170302-01	G	6	a	0.2M ammonium acetate -- 25% PEG3350 -- 0.1M bis-tris pH 5.5	0	Dimer P 1 2 1	75.48 185.0 99.73 (90.0 107.75 90.0)	P 1 2 1	3.08	9	Xia2-DIALS	17/01/2020 20:11	-	PEG	L12N	S7N	D69Y	no
G2-003	XX21RECQ L5A-x1096	Fresh	30.9	20	CI078932	HIN3-170302-01	G	2	a	0.2M lithium sulfate -- 25% PEG3350 -- 0.1M bis-tris pH 5.5	0	Dimer P 1 2 1	76.71 186.44 98.2 (90.0 107.76 90.0)	P 1 2 1	3.1	9	Xia2-DIALS	17/01/2020 20:19	-	PEG	L12N	S7N	D69Y	no
G2-003	XX21RECQ L5A-x1117	Fresh	30.9	20	CI078932	HIN3-170302-01	D	6	a	25% PEG3350 -- 0.1M bis-tris pH 5.5	0	Dimer P 1 2 1	75.78 187.98 100.8 (90.0 108.03 90.0)	P 1 2 1	3.11	9	Xia2-DIALS	17/01/2020 23:55	-	PEG	L12N	S7N	D69Y	no
G2-003	XX21RECQ L5A-x1109	Fresh	30.9	20	CI078932	HIN3-170302-01	F	1	d	0.2M L-Proline -- 10% PEG3350 -- 0.1M HEPES pH 7.5	0	Dimer P 1 2 1	76.5 182.53 100.33 (90.0 108.32 90.0)	P 1 2 1	3.11	9	Xia2-DIALS	17/01/2020 23:32	-	PEG	L12N	S7N	D69Y	no
G2-003	XX21RECQ L5A-x1105	Fresh	30.9	20	CI078932	HIN3-170302-01	F	7	d	0.2M ammonium sulfate -- 25% PEG3350 -- 0.1M bis-tris pH 6.5	0	Dimer P 1 2 1	76.27 183.58 99.27 (90.0 107.75 90.0)	P 1 2 1	3.15	9	Xia2-DIALS	17/01/2020 23:22	-	PEG	L12N	S7N	D69Y	no
G2-003	XX21RECQ L5A-x1103	Fresh	30.9	20	CI078932	HIN3-170302-01	F	9	a	0.2M ammonium sulfate -- 25% PEG3350 -- 0.1M tris pH 8.5	1	Dimer C 2 2 21	116.16 199.41 172.39 (90.00 90.00 90.00)	C 2 2 21	3.22	5	Xia2-DIALS	17/01/2020 23:15	-	PEG	L12N	S7N	D69Y	no
G2-003	XX21RECQ L5A-x1084	Fresh	30.9	20	CI078932	HIN3-170302-01	G	10	c	0.2M magnesium chloride -- 25% PEG3350 -- 0.1M bis-tris pH 5.5	0	Dimer P 1 2 1	74.19 99.21 181.1 (90.05 90.01 72.4)	P 1 2 1	3.23	9	Xia2-DIALS	17/01/2020 21:19	-	PEG	L12N	S7N	D69Y	no
G2-003	XX21RECQ L5A-x1094	Fresh	30.9	20	CI078932	HIN3-170302-01	G	5	d	0.2M lithium sulfate -- 25% PEG3350 -- 0.1M tris pH 8.5	1	Dimer C 2 2 21	114.9 198.0 174.15 (90.0 90.0 90.0)	C 2 2 21	3.25	5	Xia2-DIALS	17/01/2020 20:14	-	PEG	L12N	S7N	D69Y	no
G2-003	XX21RECQ L5A-x1095	Fresh	30.9	20	CI078932	HIN3-170302-01	G	5	a	0.2M lithium sulfate -- 25% PEG3350 -- 0.1M tris pH 8.5	1	Dimer C 2 2 21	115.54 199.62 174.88 (90.0 90.0 90.0)	C 2 2 21	3.3	5	Xia2-DIALS	17/01/2020 20:16	-	PEG	L12N	S7N	D69Y	no
G2-003	XX21RECQ L5A-x1098	Fresh	30.9	20	CI078932	HIN3-170302-01	F	12	a	0.2M sodium chloride -- 25% PEG3350 -- 0.1M HEPES pH 7.5	1	Dimer C 2 2 21	115.18 199.0 175.17 (90.0 90.0 90.0)	C 2 2 21	3.32	6	Xia2-DIALS	17/01/2020 20:24	-	PEG	L12N	S7N	D69Y	no
G2-003	XX21RECQ L5A-x1111	Fresh	30.9	20	CI078932	HIN3-170302-01	D	10	a	20% PEG5000MME -- 0.1M bis-tris pH 6.5	0	Dimer P 1 2 1	75.71 183.52 98.91 (90.0 107.94 90.0)	P 1 2 1	3.37	9	Xia2-DIALS	17/01/2020 23:37	-	PEG	L12N	S7N	D69Y	no
G2-003	XX21RECQ L5A-x1074	Fresh	30.9	20	CI078932	HIN3-170302-01	H	10	c	0.2M sodium citrate tribasic -- 20% PEG3350	1	Dimer C 2 2 21	115.24 198.21 172.96 (90.0 90.0 90.0)	C 2 2 21	3.48	4	Xia2-DIALS	17/01/2020 20:49	-	PEG	L12N	S7N	D69Y	no
G2-003	XX21RECQ L5A-x1102	Fresh	30.9	20	CI078932	HIN3-170302-01	F	9	c	0.2M ammonium sulfate -- 25% PEG3350 -- 0.1M tris pH 8.5	1	Dimer C 2 2 21	115.39 198.29 174.00 (90.00 90.00 90.00)	C 2 2 21	3.51	2	Xia2-DIALS	17/01/2020 20:36	-	PEG	L12N	S7N	D69Y	no

Table S4 Diffraction data

Nanobody grouped ID	Mounted Crystal ID	Xtal Plate Protein Frozen/Fresh?	Xtal Plate Protein Concentration (mg/ml)	Xtal Plate Temperature	Xtal Plate Barcode	Screen Batch Name	XTBM Row	XTBM Column	XTBM Subwell	XTBM Condition	Crystal Form Group	Cystal Form Annotation	Unit Cell Dimensions	Space group	Resolution	Number of Successful Autoprocessing Pipelines	Autoprocessing Pipeline Used	Experiment Time	Soaking condition	Xtal Group	Surface Mutations Around Nbinter-1	Key Mutations	Crystal Epitope Mutations	Condition Repitition?
G2-003	XX21RECQ L5A-x1080	Fresh	30.9	20	CI078932	HIN3-170302-01	H	2	d	0.2M sodium/potassium tartrate -- 20% PEG3350	1	Dimer C 2 2 21	116.57 201.12 173.15 (90.00 90.00 90.00)	C 2 2 21	3.61	7	Xia2-DIALS	17/01/2020 21:06	-	PEG	L12N	S7N	D69Y	no
G2-003	XX21RECQ L5A-x1100	Fresh	30.9	20	CI078932	HIN3-170302-01	F	11	a	0.2M sodium chloride -- 25% PEG3350 -- 0.1M bis-tris pH 6.5	1	Dimer C 2 2 21	115.06 197.52 174.49 (90.0 90.0 90.0)	C 2 2 21	3.62	5	Xia2-DIALS	17/01/2020 20:31	-	PEG	L12N	S7N	D69Y	no
G2-003	XX21RECQ L5A-x1104	Fresh	30.9	20	CI078932	HIN3-170302-01	F	8	a	0.2M ammonium sulfate -- 25% PEG3350 -- 0.1M HEPES pH 7.5	-1	NA	116.08 115.26 171.97 (89.99 89.95 59.75)	P 1	3.63	2	Xia2-DIALS	17/01/2020 23:20	-	PEG	L12N	S7N	D69Y	no
G2-003	XX21RECQ L5A-x1081	Fresh	30.9	20	CI078932	HIN3-170302-01	H	2	c	0.2M sodium/potassium tartrate -- 20% PEG3350	-1	NA	116.58 173.8 200.69 (90.0 90.01 89.96)	P 1	3.63	5	Xia2-DIALS	17/01/2020 21:08	-	PEG	L12N	S7N	D69Y	no
G2-003	XX21RECQ L5A-x1082	Fresh	30.9	20	CI078932	HIN3-170302-01	G	12	a	0.2M magnesium chloride -- 25% PEG3350 -- 0.1M HEPES pH 7.5	0	Dimer P 1 2 1	74.9 178.15 97.31 (90.0 107.5 90.0)	P 1 21 1	3.82	4	Xia2-DIALS	17/01/2020 21:12	-	PEG	L12N	S7N	D69Y	no
G2-003	XX21RECQ L5A-x1115	Fresh	30.9	20	CI078932	HIN3-170302-01	D	7	a	25% PEG3350 -- 0.1M bis-tris pH 6.5	0	Dimer P 1 2 1	75.53 183.92 100.31 (90.0 107.89 90.0)	P 1 2 1	3.84	2	Autoproc	17/01/2020 23:50	-	PEG	L12N	S7N	D69Y	no
G2-003	XX21RECQ L5A-x1113	Fresh	30.9	20	CI078932	HIN3-170302-01	D	8	c	25% PEG3350 -- 0.1M HEPES pH 7.5	0	Dimer P 1 2 1	76.57 194.93 100.61 (90.0 108.43 90.0)	P 1 21 1	4	2	Autoproc	17/01/2020 23:45	-	PEG	L12N	S7N	D69Y	no
G2-003	XX21RECQ L5A-x0385	Fresh	30.9	20	CI078932	HIN3-170302-01	F	7	c	0.2M ammonium sulfate -- 25% PEG3350 -- 0.1M bis-tris pH 6.5	0	Dimer P 1 2 1	78.09 180.8 94.88 (90.0 107.04 90.0)	P 1 2 1	4.02	1	Xia2-DIALS	06/03/2020 08:20	-	PEG	L12N	S7N	D69Y	no
G2-003	XX21RECQ L5A-x0382	Fresh	30.9	20	CI078932	HIN3-170302-01	A	5	c	2M ammonium sulfate -- 0.1M HEPES pH 7.5	-1	NA	116.63 173.74 262.58 (89.95 90.02 89.82)	P 1	5.27	1	Xia2-DIALS	06/03/2020 08:14	-	High Salt	L12N	S7N	D69Y	no
G2-003	XX21RECQ L5A-x1118	Fresh	30.9	20	CI078932	HIN3-170302-01	A	4	c	2M ammonium sulfate -- 0.1M bis-tris pH 6.5	-1	NA	116.42 264.18 176.43 (90.0 89.91 90.0)	P 1 2 1	6.18	3	Xia2-DIALS	17/01/2020 23:59	-	High Salt	L12N	S7N	D69Y	no
G2-003	XX21RECQ L5A-x0384	Fresh	30.9	20	CI078932	HIN3-170302-01	H	6	a	0.2M sodium formate -- 20% PEG3350	-1	NA	115.76 114.06 175.16 (89.94 90.02 119.58)	P 1	NA	1	NA	06/03/2020 08:18	-	PEG	L12N	S7N	D69Y	no
G2-003	XX21RECQ L5A-x1101	Fresh	30.9	20	CI078932	HIN3-170302-01	F	10	c	0.2M sodium chloride -- 25% PEG3350 -- 0.1M bis-tris pH 5.5	NA	NA	NA	NA	NA	0	NA	17/01/2020 20:33	-	PEG	L12N	S7N	D69Y	no
G2-003	XX21RECQ L5A-x1077	Fresh	30.9	20	CI078932	HIN3-170302-01	H	7	c	0.15M DL- malic acid -- 20% PEG3350	NA		NA	NA	NA	0	NA	17/01/2020 20:57	-	PEG	L12N	S7N	D69Y	no
G2-003	XX21RECQ L5A-x1076	Fresh	30.9	20	CI078932	HIN3-170302-01	H	7	d	0.15M DL- malic acid -- 20% PEG3350	NA		NA	NA	NA	0	NA	17/01/2020 20:55	-	PEG	L12N	S7N	D69Y	no
G2-003	XX21RECQ L5A-x0383	Fresh	30.9	20	CI078932	HIN3-170302-01	A	5	c	2M ammonium sulfate -- 0.1M HEPES pH 7.5	NA		NA	NA	NA	0	NA	06/03/2020 08:16	-	High Salt	L12N	S7N	D69Y	no

Table S4 Diffraction data

Nanobody grouped ID	Mounted Crystal ID	Xtal Plate Protein Frozen/Fresh?	Xtal Plate Protein Concentration (mg/ml)	Xtal Plate Temperature	Xtal Plate Barcode	Screen Batch Name	XTBM Row	XTBM Column	XTBM Subwell	XTBM Condition	Crystal Form Group	Cystal Form Annotation	Unit Cell Dimensions	Space group	Resolution	Number of Successful Autoprocessing Pipelines	Autoprocessing Pipeline Used	Experiment Time	Soaking condition	Xtal Group	Surface Mutations Around Nbinter-1	Key Mutations	Crystal Epitope Mutations	Condition Repitition?
G2-003	XX21RECQ L5A-x0381	Fresh	30.9	20	CI078932	HIN3-170302-01	A	5	c	2M ammonium sulfate -- 0.1M HEPES pH 7.5	NA		NA	NA	NA	0	NA	06/03/2020 08:11	-	High Salt	L12N	S7N	D69Y	no
G2-009	XX21RECQ L5A-x0354	Frozen	12.5595	20	CI079712	HIN3-170302-01	F	2	c	0.2M trimethylamine N-oxide -- 20% PEG2000MME -- 0.1M tris pH 8.5	0	Dimer P 1 2 1	75.9 181.88 100.16 (90.0 108.09 90.0)	P 1 21 1	2.86	5	Xia2-DIALS	01/03/2020 02:08	-	PEG	Q90R	S7N:L12C	D69Y	no
G2-009	XX21RECQ L5A-x0360	Frozen	12.5595	20	CI079712	HIN3-170302-01	F	12	c	0.2M sodium chloride -- 25% PEG3350 -- 0.1M HEPES pH 7.5	0	Dimer P 1 2 1	75.4 180.71 99.87 (90.0 107.62 90.0)	P 1 21 1	2.96	5	Xia2-DIALS	01/03/2020 02:21	-	PEG	Q90R	S7N:L12C	D69Y	no
G2-009	XX21RECQ L5A-x0357	Frozen	12.5595	20	CI079712	HIN3-170302-01	F	10	a	0.2M sodium chloride -- 25% PEG3350 -- 0.1M bis-tris pH 5.5	0	Dimer P 1 2 1	75.5 182.9 100.11 (90.0 107.73 90.0)	P 1 21 1	3.04	5	Xia2-DIALS	01/03/2020 02:14	-	PEG	Q90R	S7N:L12C	D69Y	no
G2-009	XX21RECQ L5A-x0359	Frozen	12.5595	20	CI079712	HIN3-170302-01	F	12	a	0.2M sodium chloride -- 25% PEG3350 -- 0.1M HEPES pH 7.5	0	Dimer P 1 2 1	75.72 179.99 99.32 (90.0 107.68 90.0)	P 1 21 1	3.05	5	Xia2-DIALS	01/03/2020 02:19	-	PEG	Q90R	S7N:L12C	D69Y	no
G2-009	XX21RECQ L5A-x0349	Frozen	12.5595	20	CI079712	HIN3-170302-01	D	6	a	25% PEG3350 -- 0.1M bis-tris pH 5.5	0	Dimer P 1 2 1	75.26 184.16 100.64 (90.0 107.65 90.0)	P 1 21 1	3.12	5	Xia2-DIALS	01/03/2020 01:57	-	PEG	Q90R	S7N:L12C	D69Y	no
G2-009	XX21RECQ L5A-x0376	Frozen	12.5595	20	CI079712	HIN3-170302-01	H	5	c	0.1M succinic acid -- 15% PEG3350	0	Dimer P 1 2 1	76.43 185.76 99.77 (90.0 108.11 90.0)	P 1 21 1	3.15	5	Xia2-DIALS	01/03/2020 01:46	-	PEG	Q90R	S7N:L12C	D69Y	no
G2-009	XX21RECQ L5A-x0353	Frozen	12.5595	20	CI079712	HIN3-170302-01	D	11	c	28% PEG2000MME -- 0.1M bis-tris pH 6.5	0	Dimer P 1 2 1	75.39 183.21 100.91 (90.0 107.75 90.0)	P 1 21 1	3.2	5	Xia2-DIALS	01/03/2020 02:06	-	PEG	Q90R	S7N:L12C	D69Y	no
G2-009	XX21RECQ L5A-x0351	Frozen	12.5595	20	CI079712	HIN3-170302-01	D	8	a	25% PEG3350 -- 0.1M HEPES pH 7.5	0	Dimer P 1 2 1	75.6 180.83 99.34 (90.0 107.79 90.0)	P 1 21 1	3.21	5	Xia2-DIALS	01/03/2020 02:01	-	PEG	Q90R	S7N:L12C	D69Y	no
G2-009	XX21RECQ L5A-x0370	Frozen	12.5595	20	CI079712	HIN3-170302-01	G	12	a	0.2M magnesium chloride -- 25% PEG3350 -- 0.1M HEPES pH 7.5	0	Dimer P 1 2 1	75.75 180.91 99.52 (90.0 107.64 90.0)	P 1 21 1	3.25	5	Xia2-DIALS	01/03/2020 01:33	-	PEG	Q90R	S7N:L12C	D69Y	no
G2-009	XX21RECQ L5A-x0364	Frozen	12.5595	20	CI079712	HIN3-170302-01	G	7	a	0.2M ammonium acetate -- 25% PEG3350 -- 0.1M bis-tris pH 6.5	0	Dimer P 1 2 1	75.35 182.74 99.53 (90.0 107.64 90.0)	P 1 21 1	3.29	5	Xia2-DIALS	01/03/2020 01:20	-	PEG	Q90R	S7N:L12C	D69Y	no
G2-009	XX21RECQ L5A-x0372	Frozen	12.5595	20	CI079712	HIN3-170302-01	G	12	d	0.2M magnesium chloride -- 25% PEG3350 -- 0.1M HEPES pH 7.5	0	Dimer P 1 2 1	76.15 181.96 100.5 (90.0 107.9 90.0)	P 1 21 1	3.3	5	Xia2-DIALS	01/03/2020 01:38	-	PEG	Q90R	S7N:L12C	D69Y	no
G2-009	XX21RECQ L5A-x0352	Frozen	12.5595	20	CI079712	HIN3-170302-01	D	11	a	28% PEG2000MME -- 0.1M bis-tris pH 6.5	0	Dimer P 1 2 1	75.36 182.39 99.81 (90.0 107.58 90.0)	P 1 21 1	3.33	5	Xia2-DIALS	01/03/2020 02:03	-	PEG	Q90R	S7N:L12C	D69Y	no
G2-009	XX21RECQ L5A-x0356	Frozen	12.5595	20	CI079712	HIN3-170302-01	F	6	a	0.2M ammonium sulfate -- 25% PEG3350 -- 0.1M bis-tris pH 5.5	0	Dimer P 1 2 1	75.94 186.94 99.34 (90.0 107.38 90.0)	P 1 21 1	3.38	4	Xia2-DIALS	01/03/2020 02:12	-	PEG	Q90R	S7N:L12C	D69Y	no
G2-009	XX21RECQ L5A-x0366	Frozen	12.5595	20	CI079712	HIN3-170302-01	G	9	a	0.2M ammonium acetate -- 25% PEG3350 -- 0.1M tris pH 8.5	0	Dimer P 1 2 1	75.94 181.02 98.9 (90.0 107.52 90.0)	P 1 21 1	3.47	5	Xia2-DIALS	01/03/2020 01:24	-	PEG	Q90R	S7N:L12C	D69Y	no

Table S4 Diffraction data

Nanobody grouped ID	Mounted Crystal ID	Xtal Plate Protein Frozen/Fresh?	Xtal Plate Protein Concentration (mg/ml)	Xtal Plate Temperature	Xtal Plate Barcode	Screen Batch Name	XTBM Row	XTBM Column	XTBM Subwell	XTBM Condition	Crystal Form Group	Cystal Form Annotation	Unit Cell Dimensions	Space group	Resolution	Number of Successful Autoprocessing Pipelines	Autoprocessing Pipeline Used	Experiment Time	Soaking condition	Xtal Group	Surface Mutations Around Nbinter-1	Key Mutations	Crystal Epitope Mutations	Condition Repitition?
G2-009	XX21RECQ L5A-x0358	Frozen	12.5595	20	CI079712	HIN3-170302-01	F	11	a	0.2M sodium chloride -- 25% PEG3350 -- 0.1M bis-tris pH 6.5	0	Dimer P 1 2 1	76.18 183.85 100.22 (90.0 107.87 90.0)	P 1 2 1	3.47	5	Xia2-DIALS	01/03/2020 02:16	-	PEG	Q90R	S7N:L12 C	D69Y	no
G2-009	XX21RECQ L5A-x0361	Frozen	12.5595	20	CI079712	HIN3-170302-01	G	2	a	0.2M lithium sulfate -- 25% PEG3350 -- 0.1M bis-tris pH 5.5	0	Dimer P 1 2 1	75.95 187.64 99.14 (90.0 107.36 90.0)	P 1 21 1	3.59	5	Xia2-DIALS	01/03/2020 02:23	-	PEG	Q90R	S7N:L12 C	D69Y	no
G2-009	XX21RECQ L5A-x0350	Frozen	12.5595	20	CI079712	HIN3-170302-01	D	7	a	25% PEG3350 -- 0.1M bis-tris pH 6.5	0	Dimer P 1 2 1	75.56 182.32 100.02 (90.0 107.72 90.0)	P 1 21 1	3.64	5	Xia2-DIALS	01/03/2020 01:59	-	PEG	Q90R	S7N:L12 C	D69Y	no
G2-009	XX21RECQ L5A-x0363	Frozen	12.5595	20	CI079712	HIN3-170302-01	G	6	c	0.2M ammonium acetate -- 25% PEG3350 -- 0.1M bis-tris pH 5.5	0	Dimer P 1 2 1	75.24 184.07 99.93 (90.0 107.36 90.0)	P 1 2 1	3.75	4	Xia2-DIALS	01/03/2020 01:18	-	PEG	Q90R	S7N:L12 C	D69Y	no
G2-009	XX21RECQ L5A-x0371	Frozen	12.5595	20	CI079712	HIN3-170302-01	G	12	c	0.2M magnesium chloride -- 25% PEG3350 -- 0.1M HEPES pH 7.5	0	Dimer P 1 2 1	75.7 180.01 98.46 (90.0 107.45 90.0)	P 1 21 1	4.02	3	Xia2-DIALS	01/03/2020 01:35	-	PEG	Q90R	S7N:L12 C	D69Y	no
G2-009	XX21RECQ L5A-x0362	Frozen	12.5595	20	CI079712	HIN3-170302-01	G	6	a	0.2M ammonium acetate -- 25% PEG3350 -- 0.1M bis-tris pH 5.5	0	Dimer P 1 2 1	75.44 183.05 99.74 (90.0 107.43 90.0)	P 1 21 1	4.04	3	Xia2-DIALS	01/03/2020 02:25	-	PEG	Q90R	S7N:L12 C	D69Y	no
G2-009	XX21RECQ L5A-x0374	Frozen	12.5595	20	CI079712	HIN3-170302-01	H	2	c	0.2M sodium/potassium tartrate -- 20% PEG3350	-1	NA	77.4 100.58 191.01 (89.8 89.8 71.92)	P 1	5.35	1	Xia2-DIALS	01/03/2020 01:42	-	PEG	Q90R	S7N:L12 C	D69Y	no
G2-009	XX21RECQ L5A-x0380	Frozen	12.5595	20	CI079712	HIN3-170302-01	H	12	a	0.1M potassium bromide -- 30% PEG2000MME	0	Dimer P 1 2 1	75.33 183.48 100.3 (90.0 107.29 90.0)	P 1 21 1	5.43	3	Xia2-3dii	01/03/2020 03:39	-	PEG	Q90R	S7N:L12 C	D69Y	no
G2-009	XX21RECQ L5A-x0365	Frozen	12.5595	20	CI079712	HIN3-170302-01	G	8	c	0.2M ammonium acetate -- 25% PEG3350 -- 0.1M HEPES pH 7.5	0	Dimer P 1 2 1	75.84 179.52 97.09 (90.0 107.34 90.0)	P 1 2 1	6	1	Xia2-DIALS	01/03/2020 01:22	-	PEG	Q90R	S7N:L12 C	D69Y	no
G2-009	XX21RECQ L5A-x0379	Frozen	12.5595	20	CI079712	HIN3-170302-01	H	8	c	0.1M magnesium formate -- 15% PEG3350	NA		NA	NA	NA	0	NA	01/03/2020 03:37	-	PEG	Q90R	S7N:L12 C	D69Y	no
G2-009	XX21RECQ L5A-x0378	Frozen	12.5595	20	CI079712	HIN3-170302-01	H	7	c	0.15M DL- malic acid -- 20% PEG3350	NA		NA	NA	NA	0	NA	01/03/2020 01:51	-	PEG	Q90R	S7N:L12 C	D69Y	no
G2-009	XX21RECQ L5A-x0377	Frozen	12.5595	20	CI079712	HIN3-170302-01	H	5	d	0.1M succinic acid -- 15% PEG3350	NA		NA	NA	NA	0	NA	01/03/2020 01:49	-	PEG	Q90R	S7N:L12 C	D69Y	no
G2-009	XX21RECQ L5A-x0375	Frozen	12.5595	20	CI079712	HIN3-170302-01	H	3	a	0.2M sodium malonate -- 20% PEG3350	NA		NA	NA	NA	0	NA	01/03/2020 01:44	-	PEG	Q90R	S7N:L12 C	D69Y	no
G2-009	XX21RECQ L5A-x0373	Frozen	12.5595	20	CI079712	HIN3-170302-01	H	1	a	0.2M magnesium chloride -- 25% PEG3350 -- 0.1M tris pH 8.5	NA		NA	NA	NA	0	NA	01/03/2020 01:40	-	PEG	Q90R	S7N:L12 C	D69Y	no
G2-009	XX21RECQ L5A-x0369	Frozen	12.5595	20	CI079712	HIN3-170302-01	G	11	c	0.2M magnesium chloride -- 25% PEG3350 -- 0.1M bis-tris pH 6.5	NA		NA	NA	NA	0	NA	01/03/2020 01:31	-	PEG	Q90R	S7N:L12 C	D69Y	no

Table S4 Diffraction data

Nanobody grouped ID	Mounted Crystal ID	Xtal Plate Protein Frozen/Fresh?	Xtal Plate Protein Concentration (mg/ml)	Xtal Plate Temperature	Xtal Plate Barcode	Screen Batch Name	XTBM Row	XTBM Column	XTBM Subwell	XTBM Condition	Crystal Form Group	Cystal Form Annotation	Unit Cell Dimensions	Space group	Resolution	Number of Successful Autoprocessing Pipelines	Autoprocessing Pipeline Used	Experiment Time	Soaking condition	Xtal Group	Surface Mutations Around Nbinter-1	Key Mutations	Crystal Epitope Mutations	Condition Repitition?
G2-009	XX21RECQ L5A-x0368	Frozen	12.5595	20	CI079712	HIN3-170302-01	G	11	a	0.2M magnesium chloride -- 25% PEG3350 -- 0.1M bis-tris pH 6.5	NA		NA	NA	NA	0	NA	01/03/2020 01:29	-	PEG	Q90R	S7N:L12C	D69Y	no
G2-009	XX21RECQ L5A-x0367	Frozen	12.5595	20	CI079712	HIN3-170302-01	G	9	c	0.2M ammonium acetate -- 25% PEG3350 -- 0.1M tris pH 8.5	NA		NA	NA	NA	0	NA	01/03/2020 01:27	-	PEG	Q90R	S7N:L12C	D69Y	no
G2-009	XX21RECQ L5A-x0355	Frozen	12.5595	20	CI079712	HIN3-170302-01	F	3	d	5% tacsimate -- 10% PEG5000MME -- 0.1M HEPES pH 7.0	NA		NA	NA	NA	0	NA	01/03/2020 02:10	-	PEG	Q90R	S7N:L12C	D69Y	no
G2-010	XX21RECQ L5A-x0340	Frozen	12.4989	20	CI079711	HIN3-170302-01	F	10	c	0.2M sodium chloride -- 25% PEG3350 -- 0.1M bis-tris pH 5.5	0	Dimer P 1 2 1	75.01 181.04 100.04 (90.0 107.47 90.0)	P 1 21 1	3.03	5	Xia2-DIALS	01/03/2020 02:47	-	PEG	Q90Y	S7N:L12C	D69Y	no
G2-010	XX21RECQ L5A-x0336	Frozen	12.4989	20	CI079711	HIN3-170302-01	F	6	a	0.2M ammonium sulfate -- 25% PEG3350 -- 0.1M bis-tris pH 5.5	0	Dimer P 1 2 1	75.68 182.16 98.23 (90.0 107.2 90.0)	P 1 2 1	3.63	5	Xia2-DIALS	01/03/2020 02:38	-	PEG	Q90Y	S7N:L12C	D69Y	no
G2-010	XX21RECQ L5A-x0344	Frozen	12.4989	20	CI079711	HIN3-170302-01	G	6	a	0.2M ammonium acetate -- 25% PEG3350 -- 0.1M bis-tris pH 5.5	0	Dimer P 1 2 1	75.51 181.27 99.57 (90.0 107.55 90.0)	P 1 21 1	3.74	5	Xia2-DIALS	01/03/2020 02:55	-	PEG	Q90Y	S7N:L12C	D69Y	no
G2-010	XX21RECQ L5A-x0342	Frozen	12.4989	20	CI079711	HIN3-170302-01	F	12	c	0.2M sodium chloride -- 25% PEG3350 -- 0.1M HEPES pH 7.5	0	Dimer P 1 2 1	77.06 182.25 100.9 (90.0 107.38 90.0)	P 1 21 1	3.87	3	Xia2-DIALS	01/03/2020 02:51	-	PEG	Q90Y	S7N:L12C	D69Y	no
G2-010	XX21RECQ L5A-x0339	Frozen	12.4989	20	CI079711	HIN3-170302-01	F	10	a	0.2M sodium chloride -- 25% PEG3350 -- 0.1M bis-tris pH 5.5	0	Dimer P 1 2 1	75.89 178.6 97.77 (90.0 107.46 90.0)	P 1 21 1	3.88	4	Xia2-DIALS	01/03/2020 02:45	-	PEG	Q90Y	S7N:L12C	D69Y	no
G2-010	XX21RECQ L5A-x0337	Frozen	12.4989	20	CI079711	HIN3-170302-01	F	7	a	0.2M ammonium sulfate -- 25% PEG3350 -- 0.1M bis-tris pH 6.5	0	Dimer P 1 2 1	75.58 182.7 96.91 (90.0 107.39 90.0)	P 1 2 1	5	4	Xia2-DIALS	01/03/2020 02:40	-	PEG	Q90Y	S7N:L12C	D69Y	no
G2-010	XX21RECQ L5A-x0338	Frozen	12.4989	20	CI079711	HIN3-170302-01	F	9	d	0.2M ammonium sulfate -- 25% PEG3350 -- 0.1M tris pH 8.5	0	Dimer P 1 2 1	76.41 183.29 97.95 (90.0 107.62 90.0)	P 1 2 1	5.7	5	Xia2-3dii	01/03/2020 02:43	-	PEG	Q90Y	S7N:L12C	D69Y	no
G2-010	XX21RECQ L5A-x0348	Frozen	12.4989	20	CI079711	HIN3-170302-01	H	10	c	0.2M sodium citrate tribasic -- 20% PEG3350	NA		NA	NA	NA	0	NA	01/03/2020 01:55	-	PEG	Q90Y	S7N:L12C	D69Y	no
G2-010	XX21RECQ L5A-x0347	Frozen	12.4989	20	CI079711	HIN3-170302-01	G	12	c	0.2M magnesium chloride -- 25% PEG3350 -- 0.1M HEPES pH 7.5	NA		NA	NA	NA	0	NA	01/03/2020 01:53	-	PEG	Q90Y	S7N:L12C	D69Y	no
G2-010	XX21RECQ L5A-x0346	Frozen	12.4989	20	CI079711	HIN3-170302-01	G	12	a	0.2M magnesium chloride -- 25% PEG3350 -- 0.1M HEPES pH 7.5	NA		NA	NA	NA	0	NA	01/03/2020 03:00	-	PEG	Q90Y	S7N:L12C	D69Y	no
G2-010	XX21RECQ L5A-x0345	Frozen	12.4989	20	CI079711	HIN3-170302-01	G	9	a	0.2M ammonium acetate -- 25% PEG3350 -- 0.1M tris pH 8.5	NA		NA	NA	NA	0	NA	01/03/2020 02:58	-	PEG	Q90Y	S7N:L12C	D69Y	no
G2-010	XX21RECQ L5A-x0343	Frozen	12.4989	20	CI079711	HIN3-170302-01	G	3	a	0.2M lithium sulfate -- 25% PEG3350 -- 0.1M bis-tris pH 6.5	NA		NA	NA	NA	0	NA	01/03/2020 02:53	-	PEG	Q90Y	S7N:L12C	D69Y	no

Table S4 Diffraction data

Nanobody grouped ID	Mounted Crystal ID	Xtal Plate Protein Frozen/Fresh?	Xtal Plate Protein Concentration (mg/ml)	Xtal Plate Temperature	Xtal Plate Barcode	Screen Batch Name	XTBM Row	XTBM Column	XTBM Subwell	XTBM Condition	Crystal Form Group	Cystal Form Annotation	Unit Cell Dimensions	Space group	Resolution	Number of Successful Autoprocessing Pipelines	Autoprocessing Pipeline Used	Experiment Time	Soaking condition	Xtal Group	Surface Mutations Around Nbinter-1	Key Mutations	Crystal Epitope Mutations	Condition Repitition?
G2-010	XX21RECQ L5A-x0341	Frozen	12.4989	20	CI079711	HIN3-170302-01	F	11	c	0.2M sodium chloride -- 25% PEG3350 -- 0.1M bis-tris pH 6.5	NA		NA	NA	NA	0	NA	01/03/2020 02:49	-	PEG	Q90Y	S7N:L12C	D69Y	no
G2-010	XX21RECQ L5A-x0335	Frozen	12.4989	20	CI079711	HIN3-170302-01	D	10	a	20% PEG5000MME -- 0.1M bis-tris pH 6.5	NA		NA	NA	NA	0	NA	01/03/2020 02:36	-	PEG	Q90Y	S7N:L12C	D69Y	no
G2-010	XX21RECQ L5A-x0334	Frozen	12.4989	20	CI079711	HIN3-170302-01	D	7	a	25% PEG3350 -- 0.1M bis-tris pH 6.5	NA		NA	NA	NA	0	NA	01/03/2020 02:34	-	PEG	Q90Y	S7N:L12C	D69Y	no
G2-011	XX21RECQ L5A-x0258	Frozen	9.2137	20	CI079710	HIN3-170302-01	D	11	c	28% PEG2000MME -- 0.1M bis-tris pH 6.5	0	Dimer P 1 2 1	74.99 181.23 99.8 (90.0 107.6 90.0)	P 1 21 1	2.63	5	Xia2-DIALS	01/03/2020 05:05	-	PEG	Q90H	S7N:L12C	D69Y	no
G2-011	XX21RECQ L5A-x0253	Frozen	9.2137	20	CI079710	HIN3-170302-01	D	3	c	30% jeffamine ED-2003 -- 0.1M HEPES pH 7.0	0	Dimer P 1 2 1	75.57 182.6 100.76 (90.0 107.66 90.0)	P 1 21 1	2.87	5	Xia2-DIALS	01/03/2020 04:54	-	Jeffamine	Q90H	S7N:L12C	D69Y	no
G2-011	XX21RECQ L5A-x0261	Frozen	9.2137	20	CI079710	HIN3-170302-01	F	10	c	0.2M sodium chloride -- 25% PEG3350 -- 0.1M bis-tris pH 5.5	0	Dimer P 1 2 1	75.53 182.37 99.9 (90.0 107.67 90.0)	P 1 21 1	2.9	5	Xia2-DIALS	01/03/2020 05:11	-	PEG	Q90H	S7N:L12C	D69Y	no
G2-011	XX21RECQ L5A-x0266	Frozen	9.2137	20	CI079710	HIN3-170302-01	G	6	a	0.2M ammonium acetate -- 25% PEG3350 -- 0.1M bis-tris pH 5.5	0	Dimer P 1 2 1	75.55 182.86 99.51 (90.0 107.65 90.0)	P 1 21 1	2.94	5	Xia2-DIALS	01/03/2020 02:30	-	PEG	Q90H	S7N:L12C	D69Y	no
G2-011	XX21RECQ L5A-x0257	Frozen	9.2137	20	CI079710	HIN3-170302-01	D	11	a	28% PEG2000MME -- 0.1M bis-tris pH 6.5	0	Dimer P 1 2 1	75.42 182.62 100.12 (90.0 107.88 90.0)	P 1 21 1	2.96	5	Xia2-DIALS	01/03/2020 05:02	-	PEG	Q90H	S7N:L12C	D69Y	no
G2-011	XX21RECQ L5A-x0252	Frozen	9.2137	20	CI079710	HIN3-170302-01	D	3	a	30% jeffamine ED-2003 -- 0.1M HEPES pH 7.0	0	Dimer P 1 2 1	75.72 185.01 100.46 (90.0 107.83 90.0)	P 1 21 1	3.02	4	Xia2-DIALS	01/03/2020 04:52	-	Jeffamine	Q90H	S7N:L12C	D69Y	no
G2-011	XX21RECQ L5A-x0260	Frozen	9.2137	20	CI079710	HIN3-170302-01	F	10	a	0.2M sodium chloride -- 25% PEG3350 -- 0.1M bis-tris pH 5.5	0	Dimer P 1 2 1	76.01 183.24 99.59 (90.0 107.8 90.0)	P 1 21 1	3.06	5	Xia2-DIALS	01/03/2020 05:09	-	PEG	Q90H	S7N:L12C	D69Y	no
G2-011	XX21RECQ L5A-x0263	Frozen	9.2137	20	CI079710	HIN3-170302-01	F	11	c	0.2M sodium chloride -- 25% PEG3350 -- 0.1M bis-tris pH 6.5	0	Dimer P 1 2 1	76.19 181.66 99.71 (90.0 107.76 90.0)	P 1 21 1	3.19	4	Xia2-DIALS	01/03/2020 05:15	-	PEG	Q90H	S7N:L12C	D69Y	no
G2-011	XX21RECQ L5A-x0255	Frozen	9.2137	20	CI079710	HIN3-170302-01	D	8	a	25% PEG3350 -- 0.1M HEPES pH 7.5	0	Dimer P 1 2 1	75.91 181.28 99.44 (90.0 107.96 90.0)	P 1 21 1	3.23	5	Xia2-DIALS	01/03/2020 04:58	-	PEG	Q90H	S7N:L12C	D69Y	no
G2-011	XX21RECQ L5A-x0264	Frozen	9.2137	20	CI079710	HIN3-170302-01	F	12	c	0.2M sodium chloride -- 25% PEG3350 -- 0.1M HEPES pH 7.5	0	Dimer P 1 2 1	75.83 178.91 98.84 (90.0 107.85 90.0)	P 1 21 1	3.34	5	Xia2-DIALS	01/03/2020 05:17	-	PEG	Q90H	S7N:L12C	D69Y	no
G2-011	XX21RECQ L5A-x0250	Frozen	9.2137	20	CI079710	HIN3-170302-01	A	9	a	3M sodium chloride -- 0.1M bis-tris pH 5.5	1	Dimer C 2 2 21	118.23 200.49 175.2 (90.0 90.0 90.0)	C 2 2 21	3.35	5	Xia2-DIALS	01/03/2020 04:47	-	High Salt	Q90H	S7N:L12C	D69Y	no
G2-011	XX21RECQ L5A-x0262	Frozen	9.2137	20	CI079710	HIN3-170302-01	F	11	a	0.2M sodium chloride -- 25% PEG3350 -- 0.1M bis-tris pH 6.5	0	Dimer P 1 2 1	75.97 181.98 99.02 (90.0 107.97 90.0)	P 1 21 1	3.73	3	Xia2-DIALS	01/03/2020 05:13	-	PEG	Q90H	S7N:L12C	D69Y	no

Table S4 Diffraction data

Nanobody grouped ID	Mounted Crystal ID	Xtal Plate Protein Frozen/Fresh?	Xtal Plate Protein Concentration (mg/ml)	Xtal Plate Temperature	Xtal Plate Barcode	Screen Batch Name	XTBM Row	XTBM Column	XTBM Subwell	XTBM Condition	Crystal Form Group	Cystal Form Annotation	Unit Cell Dimensions	Space group	Resolution	Number of Successful Autoprocessing Pipelines	Autoprocessing Pipeline Used	Experiment Time	Soaking condition	Xtal Group	Surface Mutations Around Nbinter-1	Key Mutations	Crystal Epitope Mutations	Condition Repitition?
G2-011	XX21RECQ L5A-x0267	Frozen	9.2137	20	CI079710	HIN3-170302-01	G	12	c	0.2M magnesium chloride -- 25% PEG3350 -- 0.1M HEPES pH 7.5	0	Dimer P 1 2 1	75.74 180.45 99.27 (90.0 107.77 90.0)	P 1 21 1	3.84	3	Xia2-DIALS	01/03/2020 02:32	-	PEG	Q90H	S7N:L12 C	D69Y	no
G2-011	XX21RECQ L5A-x0254	Frozen	9.2137	20	CI079710	HIN3-170302-01	D	7	c	25% PEG3350 -- 0.1M bis-tris pH 6.5	0	Dimer P 1 2 1	75.28 178.89 98.15 (90.0 107.46 90.0)	P 1 21 1	3.92	1	Xia2-DIALS	01/03/2020 04:56	-	PEG	Q90H	S7N:L12 C	D69Y	no
G2-011	XX21RECQ L5A-x0256	Frozen	9.2137	20	CI079710	HIN3-170302-01	D	9	a	25% PEG3350 -- 0.1M tris pH 8.5	-1	NA	77.11 100.85 184.56 (90.31 90.07 108.54)	P 1	4.35	1	Xia2-DIALS	01/03/2020 07:18	-	PEG	Q90H	S7N:L12 C	D69Y	no
G2-011	XX21RECQ L5A-x0251	Frozen	9.2137	20	CI079710	HIN3-170302-01	A	12	a	3M sodium chloride -- 0.1M tris pH 8.5	1	Dimer C 2 2 21	117.46 198.06 174.35 (90.0 90.0 90.0)	C 2 2 21	5.2	2	Xia2-3dii	01/03/2020 04:50	-	High Salt	Q90H	S7N:L12 C	D69Y	no
G2-011	XX21RECQ L5A-x0249	Frozen	9.2137	20	CI079710	HIN3-170302-01	A	3	a	2M ammonium sulfate -- 0.1M bis-tris pH 5.5	-1	NA	117.19 265.29 175.26 (90.0 90.18 90.0)	P 1 2 1	5.26	1	Xia2-DIALS	01/03/2020 04:45	-	High Salt	Q90H	S7N:L12 C	D69Y	no
G2-011	XX21RECQ L5A-x0265	Frozen	9.2137	20	CI079710	HIN3-170302-01	G	3	a	0.2M lithium sulfate -- 25% PEG3350 -- 0.1M bis-tris pH 6.5	NA		NA	NA	NA	0	NA	01/03/2020 07:20	-	PEG	Q90H	S7N:L12 C	D69Y	no
G2-011	XX21RECQ L5A-x0265	Frozen	9.2137	20	CI079710	HIN3-170302-01	G	3	a	0.2M lithium sulfate -- 25% PEG3350 -- 0.1M bis-tris pH 6.5	NA		NA	NA	NA	0	NA	01/03/2020 02:27	-	PEG	Q90H	S7N:L12 C	D69Y	no
G2-011	XX21RECQ L5A-x0259	Frozen	9.2137	20	CI079710	HIN3-170302-01	F	6	d	0.2M ammonium sulfate -- 25% PEG3350 -- 0.1M bis-tris pH 5.5	NA		NA	NA	NA	0	NA	01/03/2020 05:07	-	PEG	Q90H	S7N:L12 C	D69Y	no
G2-011	XX21RECQ L5A-x0256	Frozen	9.2137	20	CI079710	HIN3-170302-01	D	9	a	25% PEG3350 -- 0.1M tris pH 8.5	NA		NA	NA	NA	0	NA	01/03/2020 05:00	-	PEG	Q90H	S7N:L12 C	D69Y	no
G2-013	XX21RECQ L5A-x1132	Fresh	12.6	20	CI078936	HIN3-170302-01	G	6	d	0.2M ammonium acetate -- 25% PEG3350 -- 0.1M bis-tris pH 5.5	0	Dimer P 1 2 1	75.59 184.48 99.73 (90.0 107.7 90.0)	P 1 2 1	2.72	9	Xia2-DIALS	17/01/2020 17:10	-	PEG	-	S7N:L12 C	D69Y	no
G2-013	XX21RECQ L5A-x1127	Fresh	12.6	20	CI078936	HIN3-170302-01	F	10	c	0.2M sodium chloride -- 25% PEG3350 -- 0.1M bis-tris pH 5.5	0	Dimer P 1 2 1	75.83 183.11 99.91 (90.0 107.8 90.0)	P 1 2 1	2.9	9	Xia2-DIALS	17/01/2020 16:54	-	PEG	-	S7N:L12 C	D69Y	no
G2-013	XX21RECQ L5A-x1145	Fresh	12.6	20	CI078936	HIN3-170302-01	H	6	c	0.2M sodium formate -- 20% PEG3350	1	Dimer C 2 2 21	114.85 198.95 174.83 (90.00 90.00 90.00)	C 2 2 21	2.97	4	Xia2-DIALS	17/01/2020 15:34	-	PEG	-	S7N:L12 C	D69Y	no
G2-013	XX21RECQ L5A-x1146	Fresh	12.6	20	CI078936	HIN3-170302-01	H	7	c	0.15M DL- malic acid -- 20% PEG3350	0	Dimer P 1 2 1	77.01 187.24 99.63 (90.0 108.34 90.0)	P 1 2 1	3.14	9	Xia2-DIALS	17/01/2020 15:38	-	PEG	-	S7N:L12 C	D69Y	no
G2-013	XX21RECQ L5A-x1136	Fresh	12.6	20	CI078936	HIN3-170302-01	G	8	a	0.2M ammonium acetate -- 25% PEG3350 -- 0.1M HEPES pH 7.5	0	Dimer P 1 2 1	76.75 184.22 100.19 (90.0 108.05 90.0)	P 1 2 1	3.15	9	Xia2-DIALS	17/01/2020 14:53	-	PEG	-	S7N:L12 C	D69Y	no
G2-013	XX21RECQ L5A-x1140	Fresh	12.6	20	CI078936	HIN3-170302-01	G	10	c	0.2M magnesium chloride -- 25% PEG3350 -- 0.1M bis-tris pH 5.5	0	Dimer P 1 2 1	75.82 181.56 99.32 (90.0 107.77 90.0)	P 1 2 1	3.16	9	Xia2-DIALS	17/01/2020 15:23	-	PEG	-	S7N:L12 C	D69Y	no

Table S4 Diffraction data

Nanobody grouped ID	Mounted Crystal ID	Xtal Plate Protein Frozen/Fresh?	Xtal Plate Protein Concentration (mg/ml)	Xtal Plate Temperature	Xtal Plate Barcode	Screen Batch Name	XTBM Row	XTBM Column	XTBM Subwell	XTBM Condition	Crystal Form Group	Cystal Form Annotation	Unit Cell Dimensions	Space group	Resolution	Number of Successful Autoprocessing Pipelines	Autoprocessing Pipeline Used	Experiment Time	Soaking condition	Xtal Group	Surface Mutations Around Nbinter-1	Key Mutations	Crystal Epitope Mutations	Condition Repitition?
G2-013	XX21RECQ L5A-x1135	Fresh	12.6	20	CI078936	HIN3-170302-01	G	7	d	0.2M ammonium acetate -- 25% PEG3350 -- 0.1M bis-tris pH 6.5	0	Dimer P 1 2 1	75.42 180.99 98.56 (90.0 107.48 90.0)	P 1 2 1	3.17	9	Xia2-DIALS	17/01/2020 14:51	-	PEG	-	S7N:L12 C	D69Y	no
G2-013	XX21RECQ L5A-x1120	Fresh	12.6	20	CI078936	HIN3-170302-01	D	6	c	25% PEG3350 -- 0.1M bis-tris pH 5.5	0	Dimer P 1 2 1	75.45 181.59 100.02 (90.0 107.33 90.0)	P 1 21 1	3.18	6	Xia2-DIALS	17/01/2020 16:34	-	PEG	-	S7N:L12 C	D69Y	no
G2-013	XX21RECQ L5A-x1129	Fresh	12.6	20	CI078936	HIN3-170302-01	F	12	c	0.2M sodium chloride -- 25% PEG3350 -- 0.1M HEPES pH 7.5	0	Dimer P 1 2 1	75.11 177.23 98.27 (90.0 107.77 90.0)	P 1 2 1	3.2	9	Xia2-DIALS	17/01/2020 17:01	-	PEG	-	S7N:L12 C	D69Y	no
G2-013	XX21RECQ L5A-x1143	Fresh	12.6	20	CI078936	HIN3-170302-01	H	1	c	0.2M magnesium chloride -- 25% PEG3350 -- 0.1M tris pH 8.5	0	Dimer P 1 2 1	76.45 182.58 100.23 (90.0 108.45 90.0)	P 1 21 1	3.24	5	Xia2-DIALS	17/01/2020 15:29	-	PEG	-	S7N:L12 C	D69Y	no
G2-013	XX21RECQ L5A-x1125	Fresh	12.6	20	CI078936	HIN3-170302-01	F	6	a	0.2M ammonium sulfate -- 25% PEG3350 -- 0.1M bis-tris pH 5.5	0	Dimer P 1 2 1	76.32 182.61 97.72 (90.0 107.45 90.0)	P 1 2 1	3.24	9	Xia2-DIALS	17/01/2020 16:49	-	PEG	-	S7N:L12 C	D69Y	no
G2-013	XX21RECQ L5A-x1126	Fresh	12.6	20	CI078936	HIN3-170302-01	F	10	a	0.2M sodium chloride -- 25% PEG3350 -- 0.1M bis-tris pH 5.5	0	Dimer P 1 2 1	76.24 182.49 99.25 (90.0 107.75 90.0)	P 1 21 1	3.36	9	Xia2-DIALS	17/01/2020 16:51	-	PEG	-	S7N:L12 C	D69Y	no
G2-013	XX21RECQ L5A-x1137	Fresh	12.6	20	CI078936	HIN3-170302-01	G	8	c	0.2M ammonium acetate -- 25% PEG3350 -- 0.1M HEPES pH 7.5	0	Dimer P 1 2 1	75.87 179.59 98.55 (90.0 107.54 90.0)	P 1 2 1	3.46	9	Xia2-DIALS	17/01/2020 14:55	-	PEG	-	S7N:L12 C	D69Y	no
G2-013	XX21RECQ L5A-x1119	Fresh	12.6	20	CI078936	HIN3-170302-01	D	6	a	25% PEG3350 -- 0.1M bis-tris pH 5.5	0	Dimer P 1 2 1	75.31 181.45 99.08 (90.0 107.41 90.0)	P 1 2 1	3.83	9	Xia2-DIALS	17/01/2020 16:32	-	PEG	-	S7N:L12 C	D69Y	no
G2-013	XX21RECQ L5A-x1122	Fresh	12.6	20	CI078936	HIN3-170302-01	D	8	a	25% PEG3350 -- 0.1M HEPES pH 7.5	0	Dimer P 1 2 1	76.65 182.43 100.03 (90.0 107.94 90.0)	P 1 21 1	3.88	6	Xia2-DIALS	17/01/2020 16:40	-	PEG	-	S7N:L12 C	D69Y	no
G2-013	XX21RECQ L5A-x1121	Fresh	12.6	20	CI078936	HIN3-170302-01	D	7	a	25% PEG3350 -- 0.1M bis-tris pH 6.5	0	Dimer P 1 2 1	75.62 179.78 98.5 (90.0 107.5 90.0)	P 1 21 1	3.9	7	Xia2-DIALS	17/01/2020 16:36	-	PEG	-	S7N:L12 C	D69Y	no
G2-013	XX21RECQ L5A-x1134	Fresh	12.6	20	CI078936	HIN3-170302-01	G	7	c	0.2M ammonium acetate -- 25% PEG3350 -- 0.1M bis-tris pH 6.5	0	Dimer P 1 2 1	75.24 179.76 97.63 (90.0 107.59 90.0)	P 1 21 1	3.93	6	Xia2-DIALS	17/01/2020 17:14	-	PEG	-	S7N:L12 C	D69Y	no
G2-013	XX21RECQ L5A-x1133	Fresh	12.6	20	CI078936	HIN3-170302-01	G	7	a	0.2M ammonium acetate -- 25% PEG3350 -- 0.1M bis-tris pH 6.5	0	Dimer P 1 2 1	76.27 181.92 98.4 (90.0 107.58 90.0)	P 1 21 1	4.1	3	Xia2-DIALS	17/01/2020 17:12	-	PEG	-	S7N:L12 C	D69Y	no
G2-013	XX21RECQ L5A-x1130	Fresh	12.6	20	CI078936	HIN3-170302-01	G	6	a	0.2M ammonium acetate -- 25% PEG3350 -- 0.1M bis-tris pH 5.5	0	Dimer P 1 2 1	75.57 179.97 97.37 (90.0 107.52 90.0)	P 1 2 1	4.36	5	Xia2-DIALS	17/01/2020 17:03	-	PEG	-	S7N:L12 C	D69Y	no
G2-013	XX21RECQ L5A-x1147	Fresh	12.6	20	CI078936	HIN3-170302-01	H	7	d	0.15M DL- malic acid -- 20% PEG3350	0	Dimer P 1 2 1	76.86 180.32 96.14 (90.0 107.79 90.0)	P 1 2 1	4.42	3	Xia2-DIALS	17/01/2020 15:44	-	PEG	-	S7N:L12 C	D69Y	no
G2-013	XX21RECQ L5A-x1124	Fresh	12.6	20	CI078936	HIN3-170302-01	D	11	a	28% PEG2000MME -- 0.1M bis-tris pH 6.5	0	Dimer P 1 2 1	75.9 180.4 98.94 (90.0 107.51 90.0)	P 1 2 1	4.83	6	Xia2-DIALS	17/01/2020 16:46	-	PEG	-	S7N:L12 C	D69Y	no

Table S4 Diffraction data

Nanobody grouped ID	Mounted Crystal ID	Xtal Plate Protein Frozen/Fresh?	Xtal Plate Protein Concentration (mg/ml)	Xtal Plate Temperature	Xtal Plate Barcode	Screen Batch Name	XTBM Row	XTBM Column	XTBM Subwell	XTBM Condition	Crystal Form Group	Cystal Form Annotation	Unit Cell Dimensions	Space group	Resolution	Number of Successful Autoprocessing Pipelines	Autoprocessing Pipeline Used	Experiment Time	Soaking condition	Xtal Group	Surface Mutations Around Nbinter-1	Key Mutations	Crystal Epitope Mutations	Condition Repitition?
G2-013	XX21RECQ L5A-x1148	Fresh	12.6	20	CI078936	HIN3-170302-01	H	8	c	0.1M magnesium formate -- 15% PEG3350	0	Dimer P 1 2 1	78.1 180.1 92.36 (90.0 107.44 90.0)	P 1 2 1	5.32	5	Xia2-DIALS	17/01/2020 15:47	-	PEG	-	S7N:L12 C	D69Y	no
G2-013	XX21RECQ L5A-x1138	Fresh	12.6	20	CI078936	HIN3-170302-01	G	9	a	0.2M ammonium acetate -- 25% PEG3350 -- 0.1M tris pH 8.5	0	Dimer P 1 2 1	75.63 178.63 97.36 (90.0 107.77 90.0)	P 1 2 1	5.33	4	Xia2-DIALS	17/01/2020 14:59	-	PEG	-	S7N:L12 C	D69Y	no
G2-013	XX21RECQ L5A-x1131	Fresh	12.6	20	CI078936	HIN3-170302-01	G	6	c	0.2M ammonium acetate -- 25% PEG3350 -- 0.1M bis-tris pH 5.5	0	Dimer P 1 2 1	76.96 179.6 92.64 (90.0 107.34 90.0)	P 1 2 1	5.39	4	Xia2-DIALS	17/01/2020 17:06	-	PEG	-	S7N:L12 C	D69Y	no
G2-013	XX21RECQ L5A-x1123	Fresh	12.6	20	CI078936	HIN3-170302-01	D	10	a	20% PEG5000MME -- 0.1M bis-tris pH 6.5	0	Dimer P 1 2 1	76.59 183.5 99.69 (90.0 107.89 90.0)	P 1 2 1	5.94	3	Xia2-DIALS	17/01/2020 16:44	-	PEG	-	S7N:L12 C	D69Y	no
G2-013	XX21RECQ L5A-x1144	Fresh	12.6	20	CI078936	HIN3-170302-01	H	5	c	0.1M succinic acid -- 15% PEG3350	NA		NA	NA	NA	0	NA	17/01/2020 15:31	-	PEG	-	S7N:L12 C	D69Y	no
G2-013	XX21RECQ L5A-x1142	Fresh	12.6	20	CI078936	HIN3-170302-01	G	11	c	0.2M magnesium chloride -- 25% PEG3350 -- 0.1M bis-tris pH 6.5	NA		NA	NA	NA	0	NA	17/01/2020 15:27	-	PEG	-	S7N:L12 C	D69Y	no
G2-013	XX21RECQ L5A-x1141	Fresh	12.6	20	CI078936	HIN3-170302-01	G	11	a	0.2M magnesium chloride -- 25% PEG3350 -- 0.1M bis-tris pH 6.5	NA		NA	NA	NA	0	NA	17/01/2020 15:18	-	PEG	-	S7N:L12 C	D69Y	no
G2-013	XX21RECQ L5A-x1139	Fresh	12.6	20	CI078936	HIN3-170302-01	G	10	a	0.2M magnesium chloride -- 25% PEG3350 -- 0.1M bis-tris pH 5.5	NA		NA	NA	NA	0	NA	17/01/2020 15:21	-	PEG	-	S7N:L12 C	D69Y	no
G2-013	XX21RECQ L5A-x1128	Fresh	12.6	20	CI078936	HIN3-170302-01	F	11	c	0.2M sodium chloride -- 25% PEG3350 -- 0.1M bis-tris pH 6.5	NA		NA	NA	NA	0	NA	18/01/2020 09:01	-	PEG	-	S7N:L12 C	D69Y	no
G2-013	XX21RECQ L5A-x1128	Fresh	12.6	20	CI078936	HIN3-170302-01	F	11	c	0.2M sodium chloride -- 25% PEG3350 -- 0.1M bis-tris pH 6.5	NA		NA	NA	NA	0	NA	18/01/2020 08:59	-	PEG	-	S7N:L12 C	D69Y	no
G2-013	XX21RECQ L5A-x1128	Fresh	12.6	20	CI078936	HIN3-170302-01	F	11	c	0.2M sodium chloride -- 25% PEG3350 -- 0.1M bis-tris pH 6.5	NA		NA	NA	NA	0	NA	17/01/2020 16:57	-	PEG	-	S7N:L12 C	D69Y	no
G3*-011=G4-043	XX21RECQ L5A-x0688	Fresh	24.462	20	CI080144	HIN3-170302-01	F	12	d	0.2M sodium chloride -- 25% PEG3350 -- 0.1M HEPES pH 7.5	4	Tetramer I 2 2 2/ C 1 2 1	81.82 90.07 216.39 (90.0 90.0 90.0)	I 2 2 2	2.17	5	Xia2-DIALS	22/10/2020 21:33	-	PEG	G16T	S7N:L12 C:T125 M	Non-D69Y Crystal Epitopes	no
G3*-011=G4-043	XX21RECQ L5A-x0680	Fresh	24.462	20	CI080144	HIN3-170302-01	F	2	c	0.2M trimethylamine N-oxide -- 20% PEG2000MME -- 0.1M tris pH 8.5	4	Tetramer I 2 2 2/ C 1 2 1	81.96 90.21 217.62 (90.0 90.0 90.0)	I 2 2 2	2.48	5	Xia2-DIALS	22/10/2020 20:41	-	PEG	G16T	S7N:L12 C:T125 M	Non-D69Y Crystal Epitopes	no
G3*-011=G4-043	XX21RECQ L5A-x0682	Fresh	24.462	20	CI080144	HIN3-170302-01	F	7	a	0.2M ammonium sulfate -- 25% PEG3350 -- 0.1M bis-tris pH 6.5	4	Tetramer I 2 2 2/ C 1 2 1	81.22 89.62 218.35 (90.0 90.0 90.0)	I 2 2 2	2.5	5	Xia2-DIALS	22/10/2020 21:21	-	PEG	G16T	S7N:L12 C:T125 M	Non-D69Y Crystal Epitopes	no
G3*-011=G4-043	XX21RECQ L5A-x0705	Fresh	24.462	20	CI080144	HIN3-170302-01	H	3	d	0.2M sodium malonate -- 20% PEG3350	4	Tetramer I 2 2 2/ C 1 2 1	227.84 90.12 80.7 (90.0 109.36 90.0)	C 1 2 1	2.56	5	Xia2-DIALS	22/10/2020 17:56	-	PEG	G16T	S7N:L12 C:T125 M	Non-D69Y Crystal Epitopes	no

Table S4 Diffraction data

Nanobody grouped ID	Mounted Crystal ID	Xtal Plate Protein Frozen/Fresh?	Xtal Plate Protein Concentration (mg/ml)	Xtal Plate Temperature	Xtal Plate Barcode	Screen Batch Name	XTBM Row	XTBM Column	XTBM Subwell	XTBM Condition	Crystal Form Group	Cystal Form Annotation	Unit Cell Dimensions	Space group	Resolution	Number of Successful Autoprocessing Pipelines	Autoprocessing Pipeline Used	Experiment Time	Soaking condition	Xtal Group	Surface Mutations Around Nbinter-1	Key Mutations	Crystal Epitope Mutations	Condition Repitition?
G3*-011=G4-043	XX21RECQL5A-x0689	Fresh	24.462	20	CI080144	HIN3-170302-01	F	12	d	0.2M sodium chloride -- 25% PEG3350 -- 0.1M HEPES pH 7.5	4	Tetramer I 2 2 2/ C 1 2 1	224.13 90.19 80.35 (90.0 109.34 90.0)	C 1 2 1	2.79	5	Xia2-DIALS	22/10/2020 21:36	-	PEG	G16T	S7N:L12C:T125M	Non-D69Y Crystal Epitopes	no
G3*-011=G4-043	XX21RECQL5A-x0695	Fresh	24.462	20	CI080144	HIN3-170302-01	G	7	d	0.2M ammonium acetate -- 25% PEG3350 -- 0.1M bis-tris pH 6.5	4	Tetramer I 2 2 2/ C 1 2 1	220.77 90.51 80.38 (90.0 108.22 90.0)	C 1 2 1	2.92	5	Xia2-DIALS	22/10/2020 21:48	-	PEG	G16T	S7N:L12C:T125M	Non-D69Y Crystal Epitopes	no
G3*-011=G4-043	XX21RECQL5A-x0704	Fresh	24.462	20	CI080144	HIN3-170302-01	H	3	d	0.2M sodium malonate -- 20% PEG3350	4	Tetramer I 2 2 2/ C 1 2 1	219.5 90.44 80.06 (90.0 108.61 90.0)	C 1 2 1	3.09	4	Xia2-DIALS	22/10/2020 17:54	-	PEG	G16T	S7N:L12C:T125M	Non-D69Y Crystal Epitopes	no
G3*-011=G4-043	XX21RECQL5A-x0699	Fresh	24.462	20	CI080144	HIN3-170302-01	G	8	d	0.2M ammonium acetate -- 25% PEG3350 -- 0.1M HEPES pH 7.5	4	Tetramer I 2 2 2/ C 1 2 1	82.11 90.44 217.89 (90.0 90.0 90.0)	I 2 2 2	3.37	4	Autoproc	22/10/2020 17:43	-	PEG	G16T	S7N:L12C:T125M	Non-D69Y Crystal Epitopes	no
G3*-011=G4-043	XX21RECQL5A-x0701	Fresh	24.462	20	CI080144	HIN3-170302-01	G	9	c	0.2M ammonium acetate -- 25% PEG3350 -- 0.1M tris pH 8.5	4	Tetramer I 2 2 2/ C 1 2 1	227.19 90.23 80.44 (90.0 109.8 90.0)	C 1 2 1	3.39	3	Xia2-DIALS	22/10/2020 17:47	-	PEG	G16T	S7N:L12C:T125M	Non-D69Y Crystal Epitopes	no
G3*-011=G4-043	XX21RECQL5A-x0706	Fresh	24.462	20	CI080144	HIN3-170302-01	H	7	c	0.15M DL- malic acid -- 20% PEG3350	4	Tetramer I 2 2 2/ C 1 2 1	81.44 90.14 217.06 (90.0 90.0 90.0)	I 2 2 2	3.78	5	Xia2-DIALS	22/10/2020 17:59	-	PEG	G16T	S7N:L12C:T125M	Non-D69Y Crystal Epitopes	no
G3*-011=G4-043	XX21RECQL5A-x0672	Fresh	24.462	20	CI080144	HIN3-170302-01	C	2	a	1.1M ammonium tartrate	2	Tetramer P 2 2 21 Porous	88.57 144.97 268.9 (90.0 90.0 90.0)	P 2 2 2	3.8	3	Xia2-DIALS	22/10/2020 20:23	-	High Salt	G16T	S7N:L12C:T125M	Non-D69Y Crystal Epitopes	no
G3*-011=G4-043	XX21RECQL5A-x0700	Fresh	24.462	20	CI080144	HIN3-170302-01	G	9	a	0.2M ammonium acetate -- 25% PEG3350 -- 0.1M tris pH 8.5	-1	NA	121.14 216.22 80.82 (90.0 131.8 90.0)	C 1 2 1	3.81	3	Xia2-DIALS	22/10/2020 17:45	-	PEG	G16T	S7N:L12C:T125M	Non-D69Y Crystal Epitopes	no
G3*-011=G4-043	XX21RECQL5A-x0679	Fresh	24.462	20	CI080144	HIN3-170302-01	F	2	c	0.2M trimethylamine N-oxide -- 20% PEG2000MME -- 0.1M tris pH 8.5	4	Tetramer I 2 2 2/ C 1 2 1	78.86 88.83 218.35 (90.0 90.0 90.0)	I 2 2 2	4.11	1	Xia2-DIALS	22/10/2020 20:39	-	PEG	G16T	S7N:L12C:T125M	Non-D69Y Crystal Epitopes	no
G3*-011=G4-043	XX21RECQL5A-x0687	Fresh	24.462	20	CI080144	HIN3-170302-01	F	12	c	0.2M sodium chloride -- 25% PEG3350 -- 0.1M HEPES pH 7.5	4	Tetramer I 2 2 2/ C 1 2 1	219.76 90.74 79.99 (90.0 108.4 90.0)	C 1 2 1	4.41	2	Autoproc	22/10/2020 21:31	-	PEG	G16T	S7N:L12C:T125M	Non-D69Y Crystal Epitopes	no
G3*-011=G4-043	XX21RECQL5A-x0673	Fresh	24.462	20	CI080144	HIN3-170302-01	C	6	a	1.5M ammonium sulfate -- 0.1M sodium chloride -- 0.1M bis-tris pH 6.5	2	Tetramer P 2 2 21 Porous	90.58 279.58 150.18 (90.0 92.7 90.0)	P 2 2 21	4.63	2	Xia2-3dii	22/10/2020 20:26	-	High Salt	G16T	S7N:L12C:T125M	Non-D69Y Crystal Epitopes	no
G3*-011=G4-043	XX21RECQL5A-x0681	Fresh	24.462	20	CI080144	HIN3-170302-01	F	7	a	0.2M ammonium sulfate -- 25% PEG3350 -- 0.1M bis-tris pH 6.5	4	Tetramer I 2 2 2/ C 1 2 1	81.37 90.21 218.27 (90.0 90.0 90.0)	I 2 2 2	5.03	4	Xia2-3dii	22/10/2020 20:43	-	PEG	G16T	S7N:L12C:T125M	Non-D69Y Crystal Epitopes	no
G3*-011=G4-043	XX21RECQL5A-x0676	Fresh	24.462	20	CI080144	HIN3-170302-01	C	12	d	15% tacsimate -- 2% PEG3350 -- 0.1M HEPES pH 7.0	2	Tetramer P 2 2 21 Porous	155.25 272.01 90.81 (90.0 90.0 90.0)	P 21 21 2	7.13	1	Xia2-3dii	22/10/2020 20:32	-	High Salt	G16T	S7N:L12C:T125M	Non-D69Y Crystal Epitopes	no
G3*-011=G4-043	XX21RECQL5A-x0666	Fresh	24.462	20	CI080144	HIN3-170302-01	A	10	a	3M sodium chloride -- 0.1M bis-tris pH 6.5	2	Tetramer P 2 2 21 Porous	92.6 158.02 277.05 (90.0 90.0 90.0)	P 2 2 21	8	1	Xia2-3dii	22/10/2020 20:11	-	High Salt	G16T	S7N:L12C:T125M	Non-D69Y Crystal Epitopes	no
G3*-011=G4-043	XX21RECQL5A-x0707	Fresh	24.462	20	CI080144	HIN3-170302-01	H	7	d	0.15M DL- malic acid -- 20% PEG3350	NA		NA	NA	NA	0	NA	22/10/2020 18:00	-	PEG	G16T	S7N:L12C:T125M	Non-D69Y Crystal Epitopes	no

Table S4 Diffraction data

Nanobody grouped ID	Mounted Crystal ID	Xtal Plate Protein Frozen/Fresh?	Xtal Plate Protein Concentration (mg/ml)	Xtal Plate Temperature	Xtal Plate Barcode	Screen Batch Name	XTBM Row	XTBM Column	XTBM Subwell	XTBM Condition	Crystal Form Group	Cystal Form Annotation	Unit Cell Dimensions	Space group	Resolution	Number of Successful Autoprocessing Pipelines	Autoprocessing Pipeline Used	Experiment Time	Soaking condition	Xtal Group	Surface Mutations Around Nbinter-1	Key Mutations	Crystal Epitope Mutations	Condition Repitition?
G3*-011=G4-043	XX21RECQL5A-x0703	Fresh	24.462	20	CI080144	HIN3-170302-01	H	1	c	0.2M magnesium chloride -- 25% PEG3350 -- 0.1M tris pH 8.5	NA		NA	NA	NA	0	NA	22/10/2020 17:52	-	PEG	G16T	S7N:L12C:T125M	Non-D69Y Crystal Epitopes	no
G3*-011=G4-043	XX21RECQL5A-x0702	Fresh	24.462	20	CI080144	HIN3-170302-01	G	9	c	0.2M ammonium acetate -- 25% PEG3350 -- 0.1M tris pH 8.5	NA		NA	NA	NA	0	NA	22/10/2020 17:49	-	PEG	G16T	S7N:L12C:T125M	Non-D69Y Crystal Epitopes	no
G3*-011=G4-043	XX21RECQL5A-x0698	Fresh	24.462	20	CI080144	HIN3-170302-01	G	8	c	0.2M ammonium acetate -- 25% PEG3350 -- 0.1M HEPES pH 7.5	NA		NA	NA	NA	0	NA	22/10/2020 17:41	-	PEG	G16T	S7N:L12C:T125M	Non-D69Y Crystal Epitopes	no
G3*-011=G4-043	XX21RECQL5A-x0697	Fresh	24.462	20	CI080144	HIN3-170302-01	G	8	a	0.2M ammonium acetate -- 25% PEG3350 -- 0.1M HEPES pH 7.5	NA		NA	NA	NA	0	NA	22/10/2020 21:53	-	PEG	G16T	S7N:L12C:T125M	Non-D69Y Crystal Epitopes	no
G3*-011=G4-043	XX21RECQL5A-x0696	Fresh	24.462	20	CI080144	HIN3-170302-01	G	8	a	0.2M ammonium acetate -- 25% PEG3350 -- 0.1M HEPES pH 7.5	NA		NA	NA	NA	0	NA	22/10/2020 21:51	-	PEG	G16T	S7N:L12C:T125M	Non-D69Y Crystal Epitopes	no
G3*-011=G4-043	XX21RECQL5A-x0694	Fresh	24.462	20	CI080144	HIN3-170302-01	G	7	d	0.2M ammonium acetate -- 25% PEG3350 -- 0.1M bis-tris pH 6.5	NA		NA	NA	NA	0	NA	22/10/2020 21:46	-	PEG	G16T	S7N:L12C:T125M	Non-D69Y Crystal Epitopes	no
G3*-011=G4-043	XX21RECQL5A-x0693	Fresh	24.462	20	CI080144	HIN3-170302-01	G	7	c	0.2M ammonium acetate -- 25% PEG3350 -- 0.1M bis-tris pH 6.5	NA		NA	NA	NA	0	NA	22/10/2020 21:44	-	PEG	G16T	S7N:L12C:T125M	Non-D69Y Crystal Epitopes	no
G3*-011=G4-043	XX21RECQL5A-x0692	Fresh	24.462	20	CI080144	HIN3-170302-01	G	3	a	0.2M lithium sulfate -- 25% PEG3350 -- 0.1M bis-tris pH 6.5	NA		NA	NA	NA	0	NA	22/10/2020 21:42	-	PEG	G16T	S7N:L12C:T125M	Non-D69Y Crystal Epitopes	no
G3*-011=G4-043	XX21RECQL5A-x0691	Fresh	24.462	20	CI080144	HIN3-170302-01	G	1	d	0.2M sodium chloride -- 25% PEG3350 -- 0.1M tris pH 8.5	NA		NA	NA	NA	0	NA	22/10/2020 21:40	-	PEG	G16T	S7N:L12C:T125M	Non-D69Y Crystal Epitopes	no
G3*-011=G4-043	XX21RECQL5A-x0690	Fresh	24.462	20	CI080144	HIN3-170302-01	G	1	c	0.2M sodium chloride -- 25% PEG3350 -- 0.1M tris pH 8.5	NA		NA	NA	NA	0	NA	22/10/2020 21:38	-	PEG	G16T	S7N:L12C:T125M	Non-D69Y Crystal Epitopes	no
G3*-011=G4-043	XX21RECQL5A-x0686	Fresh	24.462	20	CI080144	HIN3-170302-01	F	12	a	0.2M sodium chloride -- 25% PEG3350 -- 0.1M HEPES pH 7.5	NA		NA	NA	NA	0	NA	22/10/2020 21:29	-	PEG	G16T	S7N:L12C:T125M	Non-D69Y Crystal Epitopes	no
G3*-011=G4-043	XX21RECQL5A-x0685	Fresh	24.462	20	CI080144	HIN3-170302-01	F	8	c	0.2M ammonium sulfate -- 25% PEG3350 -- 0.1M HEPES pH 7.5	NA		NA	NA	NA	0	NA	22/10/2020 21:27	-	PEG	G16T	S7N:L12C:T125M	Non-D69Y Crystal Epitopes	no
G3*-011=G4-043	XX21RECQL5A-x0684	Fresh	24.462	20	CI080144	HIN3-170302-01	F	7	d	0.2M ammonium sulfate -- 25% PEG3350 -- 0.1M bis-tris pH 6.5	NA		NA	NA	NA	0	NA	22/10/2020 21:25	-	PEG	G16T	S7N:L12C:T125M	Non-D69Y Crystal Epitopes	no
G3*-011=G4-043	XX21RECQL5A-x0683	Fresh	24.462	20	CI080144	HIN3-170302-01	F	7	c	0.2M ammonium sulfate -- 25% PEG3350 -- 0.1M bis-tris pH 6.5	NA		NA	NA	NA	0	NA	22/10/2020 21:23	-	PEG	G16T	S7N:L12C:T125M	Non-D69Y Crystal Epitopes	no
G3*-011=G4-043	XX21RECQL5A-x0678	Fresh	24.462	20	CI080144	HIN3-170302-01	D	9	a	25% PEG3350 -- 0.1M tris pH 8.5	NA		NA	NA	NA	0	NA	22/10/2020 20:37	-	PEG	G16T	S7N:L12C:T125M	Non-D69Y Crystal Epitopes	no

Table S4 Diffraction data

Nanobody grouped ID	Mounted Crystal ID	Xtal Plate Protein Frozen/Fresh?	Xtal Plate Protein Concentration (mg/ml)	Xtal Plate Temperature	Xtal Plate Barcode	Screen Batch Name	XTBM Row	XTBM Column	XTBM Subwell	XTBM Condition	Crystal Form Group	Cystal Form Annotation	Unit Cell Dimensions	Space group	Resolution	Number of Successful Autoprocessing Pipelines	Autoprocessing Pipeline Used	Experiment Time	Soaking condition	Xtal Group	Surface Mutations Around Nbinter-1	Key Mutations	Crystal Epitope Mutations	Condition Repitition?
G3*-011=G4-043	XX21RECQL5A-x0677	Fresh	24.462	20	CI080144	HIN3-170302-01	C	12	d	15% tacsimate -- 2% PEG3350 -- 0.1M HEPES pH 7.0	NA		NA	NA	NA	0	NA	22/10/2020 20:34	-	High Salt	G16T	S7N:L12C:T125M	Non-D69Y Crystal Epitopes	no
G3*-011=G4-043	XX21RECQL5A-x0675	Fresh	24.462	20	CI080144	HIN3-170302-01	C	7	c	0.8M sodium/potassium tartrate -- 0.5% PEG5000MME -- 0.1M tris pH 8.5	NA		NA	NA	NA	0	NA	22/10/2020 20:29	-	High Salt	G16T	S7N:L12C:T125M	Non-D69Y Crystal Epitopes	no
G3*-011=G4-043	XX21RECQL5A-x0674	Fresh	24.462	20	CI080144	HIN3-170302-01	C	6	a	1.5M ammonium sulfate -- 0.1M sodium chloride -- 0.1M bis-tris pH 6.5	NA		NA	NA	NA	0	NA	22/10/2020 20:28	-	High Salt	G16T	S7N:L12C:T125M	Non-D69Y Crystal Epitopes	no
G3*-011=G4-043	XX21RECQL5A-x0671	Fresh	24.462	20	CI080144	HIN3-170302-01	C	2	a	1.1M ammonium tartrate	NA		NA	NA	NA	0	NA	22/10/2020 20:21	-	High Salt	G16T	S7N:L12C:T125M	Non-D69Y Crystal Epitopes	no
G3*-011=G4-043	XX21RECQL5A-x0670	Fresh	24.462	20	CI080144	HIN3-170302-01	A	12	d	3M sodium chloride -- 0.1M tris pH 8.5	NA		NA	NA	NA	0	NA	22/10/2020 20:18	-	High Salt	G16T	S7N:L12C:T125M	Non-D69Y Crystal Epitopes	no
G3*-011=G4-043	XX21RECQL5A-x0669	Fresh	24.462	20	CI080144	HIN3-170302-01	A	12	c	3M sodium chloride -- 0.1M tris pH 8.5	NA		NA	NA	NA	0	NA	22/10/2020 20:17	-	High Salt	G16T	S7N:L12C:T125M	Non-D69Y Crystal Epitopes	no
G3*-011=G4-043	XX21RECQL5A-x0668	Fresh	24.462	20	CI080144	HIN3-170302-01	A	11	d	3M sodium chloride -- 0.1M HEPES pH 7.5	NA		NA	NA	NA	0	NA	22/10/2020 20:15	-	High Salt	G16T	S7N:L12C:T125M	Non-D69Y Crystal Epitopes	no
G3*-011=G4-043	XX21RECQL5A-x0667	Fresh	24.462	20	CI080144	HIN3-170302-01	A	11	d	3M sodium chloride -- 0.1M HEPES pH 7.5	NA		NA	NA	NA	0	NA	22/10/2020 20:13	-	High Salt	G16T	S7N:L12C:T125M	Non-D69Y Crystal Epitopes	no
G3*-049=G4-047	XX21RECQL5A-x0710	Frozen	22.4	20	CI080420	HIN3-170302-01	A	2	d	2M ammonium sulfate -- 0.1M acetate pH 4.5	2	Tetramer P 2 2 21 Porous	90.17 147.23 270.48 (90.0 90.0 90.0)	P 2 2 21	5.16	4	Xia2-3dii	04/12/2020 05:59	-	High Salt	Q90Y	S7N:L12C:T125M	Non-D69Y Crystal Epitopes	no
G3*-049=G4-047	XX21RECQL5A-x0723	Frozen	22.4	20	CI080420	HIN3-170302-01	D	2	d	30% jeffamine M-600 -- 0.1M HEPES pH 7.0	NA		NA	NA	NA	0	NA	04/12/2020 06:56	-	Jeffamine	Q90Y	S7N:L12C:T125M	Non-D69Y Crystal Epitopes	no
G3*-049=G4-047	XX21RECQL5A-x0722	Frozen	22.4	20	CI080420	HIN3-170302-01	D	2	d	30% jeffamine M-600 -- 0.1M HEPES pH 7.0	NA		NA	NA	NA	0	NA	04/12/2020 06:54	-	Jeffamine	Q90Y	S7N:L12C:T125M	Non-D69Y Crystal Epitopes	no
G3*-049=G4-047	XX21RECQL5A-x0721	Frozen	22.4	20	CI080420	HIN3-170302-01	D	2	c	30% jeffamine M-600 -- 0.1M HEPES pH 7.0	NA		NA	NA	NA	0	NA	04/12/2020 06:51	-	Jeffamine	Q90Y	S7N:L12C:T125M	Non-D69Y Crystal Epitopes	no
G3*-049=G4-047	XX21RECQL5A-x0720	Frozen	22.4	20	CI080420	HIN3-170302-01	D	1	d	25% PEG1500	NA		NA	NA	NA	0	NA	04/12/2020 06:48	-	PEG	Q90Y	S7N:L12C:T125M	Non-D69Y Crystal Epitopes	no
G3*-049=G4-047	XX21RECQL5A-x0719	Frozen	22.4	20	CI080420	HIN3-170302-01	D	1	c	25% PEG1500	NA		NA	NA	NA	0	NA	04/12/2020 06:45	-	PEG	Q90Y	S7N:L12C:T125M	Non-D69Y Crystal Epitopes	no
G3*-049=G4-047	XX21RECQL5A-x0718	Frozen	22.4	20	CI080420	HIN3-170302-01	D	1	a	25% PEG1500	NA		NA	NA	NA	0	NA	04/12/2020 06:42	-	PEG	Q90Y	S7N:L12C:T125M	Non-D69Y Crystal Epitopes	no

Table S4 Diffraction data

Nanobody grouped ID	Mounted Crystal ID	Xtal Plate Protein Frozen/Fresh?	Xtal Plate Protein Concentration (mg/ml)	Xtal Plate Temperature	Xtal Plate Barcode	Screen Batch Name	XTBM Row	XTBM Column	XTBM Subwell	XTBM Condition	Crystal Form Group	Cystal Form Annotation	Unit Cell Dimensions	Space group	Resolution	Number of Successful Autoprocessing Pipelines	Autoprocessing Pipeline Used	Experiment Time	Soaking condition	Xtal Group	Surface Mutations Around Nbinter-1	Key Mutations	Crystal Epitope Mutations	Condition Repitition?
G3*-049=G4-047	XX21RECQL5A-x0717	Frozen	22.4	20	CI080420	HIN3-170302-01	C	2	d	1.1M ammonium tartrate	NA		NA	NA	NA	0	NA	04/12/2020 06:39	-	High Salt	Q90Y	S7N:L12C:T125M	Non-D69Y Crystal Epitopes	no
G3*-049=G4-047	XX21RECQL5A-x0716	Frozen	22.4	20	CI080420	HIN3-170302-01	B	2	d	0.5M magnesium formate -- 0.1M bis-tris pH 6.5	NA		NA	NA	NA	0	NA	04/12/2020 06:36	-	High Salt	Q90Y	S7N:L12C:T125M	Non-D69Y Crystal Epitopes	no
G3*-049=G4-047	XX21RECQL5A-x0715	Frozen	22.4	20	CI080420	HIN3-170302-01	B	2	c	0.5M magnesium formate -- 0.1M bis-tris pH 6.5	NA		NA	NA	NA	0	NA	04/12/2020 06:33	-	High Salt	Q90Y	S7N:L12C:T125M	Non-D69Y Crystal Epitopes	no
G3*-049=G4-047	XX21RECQL5A-x0714	Frozen	22.4	20	CI080420	HIN3-170302-01	B	1	d	0.3M magnesium formate -- 0.1M bis-tris pH 5.5	NA		NA	NA	NA	0	NA	04/12/2020 06:30	-	High Salt	Q90Y	S7N:L12C:T125M	Non-D69Y Crystal Epitopes	no
G3*-049=G4-047	XX21RECQL5A-x0713	Frozen	22.4	20	CI080420	HIN3-170302-01	B	1	c	0.3M magnesium formate -- 0.1M bis-tris pH 5.5	NA		NA	NA	NA	0	NA	04/12/2020 06:07	-	High Salt	Q90Y	S7N:L12C:T125M	Non-D69Y Crystal Epitopes	no
G3*-049=G4-047	XX21RECQL5A-x0712	Frozen	22.4	20	CI080420	HIN3-170302-01	B	1	a	0.3M magnesium formate -- 0.1M bis-tris pH 5.5	NA		NA	NA	NA	0	NA	04/12/2020 06:05	-	High Salt	Q90Y	S7N:L12C:T125M	Non-D69Y Crystal Epitopes	no
G3*-049=G4-047	XX21RECQL5A-x0711	Frozen	22.4	20	CI080420	HIN3-170302-01	A	3	a	2M ammonium sulfate -- 0.1M bis-tris pH 5.5	NA		NA	NA	NA	0	NA	04/12/2020 06:02	-	High Salt	Q90Y	S7N:L12C:T125M	Non-D69Y Crystal Epitopes	no
G3*-049=G4-047	XX21RECQL5A-x0709	Frozen	22.4	20	CI080420	HIN3-170302-01	A	2	c	2M ammonium sulfate -- 0.1M acetate pH 4.5	NA		NA	NA	NA	0	NA	04/12/2020 05:56	-	High Salt	Q90Y	S7N:L12C:T125M	Non-D69Y Crystal Epitopes	no
G3*-049=G4-047	XX21RECQL5A-x0708	Frozen	22.4	20	CI080420	HIN3-170302-01	A	2	a	2M ammonium sulfate -- 0.1M acetate pH 4.5	NA		NA	NA	NA	0	NA	04/12/2020 08:15	-	High Salt	Q90Y	S7N:L12C:T125M	Non-D69Y Crystal Epitopes	no
G3*-050	XX21RECQL5A-x0651	Fresh	35.73	20	CI080005	HIN3-170302-01	A	10	a	3M sodium chloride -- 0.1M bis-tris pH 6.5	2	Tetramer P 2 2 21 Porous	89.82 152.95 272.6 (90.0 90.0 90.0)	P 2 2 2	3.41	5	Xia2-DIALS	14/10/2020 08:09	-	High Salt	Q90Y	S7N:L12C	Non-D69Y Crystal Epitopes	no
G3*-050	XX21RECQL5A-x0662	Fresh	35.73	20	CI080005	HIN3-170302-01	C	6	a	1.5M ammonium sulfate -- 0.1M sodium chloride -- 0.1M bis-tris pH 6.5	2	Tetramer P 2 2 21 Porous	90.0 151.84 271.6 (90.0 90.0 90.0)	P 2 2 2	3.97	4	Xia2-3dii	14/10/2020 08:41	-	High Salt	Q90Y	S7N:L12C	Non-D69Y Crystal Epitopes	no
G3*-050	XX21RECQL5A-x0663	Fresh	35.73	20	CI080005	HIN3-170302-01	C	6	a	1.5M ammonium sulfate -- 0.1M sodium chloride -- 0.1M bis-tris pH 6.5	2	Tetramer P 2 2 21 Porous	90.01 152.51 271.14 (90.0 90.0 90.0)	P 2 2 2	4	2	Xia2-3dii	14/10/2020 08:43	-	High Salt	Q90Y	S7N:L12C	Non-D69Y Crystal Epitopes	no
G3*-050	XX21RECQL5A-x0664	Fresh	35.73	20	CI080005	HIN3-170302-01	C	6	c	1.5M ammonium sulfate -- 0.1M sodium chloride -- 0.1M bis-tris pH 6.5	2	Tetramer P 2 2 21 Porous	90.0 151.11 271.14 (90.0 90.0 90.0)	P 2 2 2	4.04	2	Xia2-3dii	14/10/2020 08:46	-	High Salt	Q90Y	S7N:L12C	Non-D69Y Crystal Epitopes	no
G3*-050	XX21RECQL5A-x0657	Fresh	35.73	20	CI080005	HIN3-170302-01	B	10	a	0.8M succinic acid	2	Tetramer P 2 2 21 Porous	90.46 153.12 270.12 (90.0 90.0 90.0)	P 2 2 2	4.3	4	Xia2-3dii	14/10/2020 08:26	-	High Salt	Q90Y	S7N:L12C	Non-D69Y Crystal Epitopes	no
G3*-050	XX21RECQL5A-x0659	Fresh	35.73	20	CI080005	HIN3-170302-01	B	10	c	0.8M succinic acid	2	Tetramer P 2 2 21 Porous	90.36 155.41 268.95 (90.0 90.0 90.0)	P 2 2 21	5.07	4	Xia2-3dii	14/10/2020 08:32	-	High Salt	Q90Y	S7N:L12C	Non-D69Y Crystal Epitopes	no

Table S4 Diffraction data

Nanobody grouped ID	Mounted Crystal ID	Xtal Plate Protein Frozen/Fresh?	Xtal Plate Protein Concentration (mg/ml)	Xtal Plate Temperature	Xtal Plate Barcode	Screen Batch Name	XTBM Row	XTBM Column	XTBM Subwell	XTBM Condition	Crystal Form Group	Cystal Form Annotation	Unit Cell Dimensions	Space group	Resolution	Number of Successful Autoprocessing Pipelines	Autoprocessing Pipeline Used	Experiment Time	Soaking condition	Xtal Group	Surface Mutations Around Nbinter-1	Key Mutations	Crystal Epitope Mutations	Condition Repitition?
G3*-050	XX21RECQ L5A-x0650	Fresh	35.73	20	CI080005	HIN3-170302-01	A	10	a	3M sodium chloride -- 0.1M bis-tris pH 6.5	2	Tetramer P 2 2 21 Porous	152.91 272.23 90.05 (90.0 90.0 90.0)	P 21 21 2	5.84	4	Xia2-3dii	14/10/2020 08:06	-	High Salt	Q90Y	S7N:L12 C	Non-D69Y Crystal Epitopes	no
G3*-050	XX21RECQ L5A-x0656	Fresh	35.73	20	CI080005	HIN3-170302-01	B	10	a	0.8M succinic acid	2	Tetramer P 2 2 21 Porous	85.25 144.3 254.73 (90.0 90.0 90.0)	P 2 2 2	11	1	Xia2-3dii	14/10/2020 08:23	-	High Salt	Q90Y	S7N:L12 C	Non-D69Y Crystal Epitopes	no
G3*-050	XX21RECQ L5A-x0665	Fresh	35.73	20	CI080005	HIN3-170302-01	H	6	a	0.2M sodium formate -- 20% PEG3350	NA		NA	NA	NA	0	NA	14/10/2020 08:49	-	PEG	Q90Y	S7N:L12 C	Non-D69Y Crystal Epitopes	no
G3*-050	XX21RECQ L5A-x0661	Fresh	35.73	20	CI080005	HIN3-170302-01	C	4	a	35% tacsimate	NA		NA	NA	NA	0	NA	14/10/2020 08:38	-	High Salt	Q90Y	S7N:L12 C	Non-D69Y Crystal Epitopes	no
G3*-050	XX21RECQ L5A-x0660	Fresh	35.73	20	CI080005	HIN3-170302-01	B	10	d	0.8M succinic acid	NA		NA	NA	NA	0	NA	14/10/2020 08:35	-	High Salt	Q90Y	S7N:L12 C	Non-D69Y Crystal Epitopes	no
G3*-050	XX21RECQ L5A-x0658	Fresh	35.73	20	CI080005	HIN3-170302-01	B	10	c	0.8M succinic acid	NA		NA	NA	NA	0	NA	14/10/2020 08:29	-	High Salt	Q90Y	S7N:L12 C	Non-D69Y Crystal Epitopes	no
G3*-050	XX21RECQ L5A-x0655	Fresh	35.73	20	CI080005	HIN3-170302-01	B	6	c	0.49M sodium phosphate monobasic -- 0.91M potassium phosphate dibasic	NA		NA	NA	NA	0	NA	14/10/2020 08:20	-	High Salt	Q90Y	S7N:L12 C	Non-D69Y Crystal Epitopes	no
G3*-050	XX21RECQ L5A-x0654	Fresh	35.73	20	CI080005	HIN3-170302-01	B	6	c	0.49M sodium phosphate monobasic -- 0.91M potassium phosphate dibasic	NA		NA	NA	NA	0	NA	14/10/2020 08:18	-	High Salt	Q90Y	S7N:L12 C	Non-D69Y Crystal Epitopes	no
G3*-050	XX21RECQ L5A-x0653	Fresh	35.73	20	CI080005	HIN3-170302-01	B	6	a	0.49M sodium phosphate monobasic -- 0.91M potassium phosphate dibasic	NA		NA	NA	NA	0	NA	14/10/2020 08:15	-	High Salt	Q90Y	S7N:L12 C	Non-D69Y Crystal Epitopes	no
G3*-050	XX21RECQ L5A-x0652	Fresh	35.73	20	CI080005	HIN3-170302-01	B	6	a	0.49M sodium phosphate monobasic -- 0.91M potassium phosphate dibasic	NA		NA	NA	NA	0	NA	14/10/2020 08:12	-	High Salt	Q90Y	S7N:L12 C	Non-D69Y Crystal Epitopes	no
G3-003	XX21RECQ L5A-x0397	Frozen	34.2675	20	CI079715	HIN3-170302-01	A	10	a	3M sodium chloride -- 0.1M bis-tris pH 6.5	2	Tetramer P 2 2 21 Porous	151.57 88.7 275.51 (90.0 90.06 90.0)	P 21 21 2	3.79	5	Xia2-DIALS	14/05/2020 00:00	-	High Salt	Q14K	S7N:L12 C	Non-D69Y Crystal Epitopes	no
G3-003	XX21RECQ L5A-x0404	Frozen	34.2675	20	CI079715	HIN3-170302-01	C	6	a	1.5M ammonium sulfate -- 0.1M sodium chloride -- 0.1M bis-tris pH 6.5	2	Tetramer P 2 2 21 Porous	89.27 144.81 278.84 (90.0 90.0 90.0)	P 2 2 21	4.79	1	Xia2-DIALS	14/05/2020 00:28	-	High Salt	Q14K	S7N:L12 C	Non-D69Y Crystal Epitopes	no
G3-003	XX21RECQ L5A-x0401	Frozen	34.2675	20	CI079715	HIN3-170302-01	B	6	c	0.49M sodium phosphate monobasic -- 0.91M potassium phosphate dibasic	2	Tetramer P 2 2 21 Porous	85.37 142.7 261.96 (90.0 90.0 90.0)	P 2 2 21	9.58	1	Xia2-3dii	14/05/2020 00:16	-	High Salt	Q14K	S7N:L12 C	Non-D69Y Crystal Epitopes	no
G3-003	XX21RECQ L5A-x0406	Frozen	34.2675	20	CI079715	HIN3-170302-01	C	10	a	1M succinic acid -- 1% PEG2000MME -- 0.1M HEPES pH 7.0	NA		NA	NA	NA	0	NA	14/05/2020 00:36	-	High Salt	Q14K	S7N:L12 C	Non-D69Y Crystal Epitopes	no
G3-003	XX21RECQ L5A-x0405	Frozen	34.2675	20	CI079715	HIN3-170302-01	C	6	c	1.5M ammonium sulfate -- 0.1M sodium chloride -- 0.1M bis-tris pH 6.5	NA		NA	NA	NA	0	NA	14/05/2020 00:32	-	High Salt	Q14K	S7N:L12 C	Non-D69Y Crystal Epitopes	no

Table S4 Diffraction data

Nanobody grouped ID	Mounted Crystal ID	Xtal Plate Protein Frozen/Fresh?	Xtal Plate Protein Concentration (mg/ml)	Xtal Plate Temperature	Xtal Plate Barcode	Screen Batch Name	XTBM Row	XTBM Column	XTBM Subwell	XTBM Condition	Crystal Form Group	Cystal Form Annotation	Unit Cell Dimensions	Space group	Resolution	Number of Successful Autoprocessing Pipelines	Autoprocessing Pipeline Used	Experiment Time	Soaking condition	Xtal Group	Surface Mutations Around Nbinter-1	Key Mutations	Crystal Epitope Mutations	Condition Repitition?
G3-003	XX21RECQ L5A-x0403	Frozen	34.2675	20	CI079715	HIN3-170302-01	C	4	a	35% tacsimate	NA		NA	NA	NA	0	NA	14/05/2020 00:24	-	High Salt	Q14K	S7N:L12 C	Non-D69Y Crystal Epitopes	no
G3-003	XX21RECQ L5A-x0402	Frozen	34.2675	20	CI079715	HIN3-170302-01	B	6	d	0.49M sodium phosphate monobasic -- 0.91M potassium phosphate dibasic	NA		NA	NA	NA	0	NA	14/05/2020 00:20	-	High Salt	Q14K	S7N:L12 C	Non-D69Y Crystal Epitopes	no
G3-003	XX21RECQ L5A-x0400	Frozen	34.2675	20	CI079715	HIN3-170302-01	B	6	a	0.49M sodium phosphate monobasic -- 0.91M potassium phosphate dibasic	NA		NA	NA	NA	0	NA	14/05/2020 00:12	-	High Salt	Q14K	S7N:L12 C	Non-D69Y Crystal Epitopes	no
G3-003	XX21RECQ L5A-x0399	Frozen	34.2675	20	CI079715	HIN3-170302-01	A	12	d	3M sodium chloride -- 0.1M tris pH 8.5	NA		NA	NA	NA	0	NA	14/05/2020 00:08	-	High Salt	Q14K	S7N:L12 C	Non-D69Y Crystal Epitopes	no
G3-003	XX21RECQ L5A-x0398	Frozen	34.2675	20	CI079715	HIN3-170302-01	A	10	c	3M sodium chloride -- 0.1M bis-tris pH 6.5	NA		NA	NA	NA	0	NA	14/05/2020 00:04	-	High Salt	Q14K	S7N:L12 C	Non-D69Y Crystal Epitopes	no
G3-003	XX21RECQ L5A-x0396	Frozen	34.2675	20	CI079715	HIN3-170302-01	A	5	a	2M ammonium sulfate -- 0.1M HEPES pH 7.5	NA		NA	NA	NA	0	NA	13/05/2020 23:56	-	High Salt	Q14K	S7N:L12 C	Non-D69Y Crystal Epitopes	no
G3-004	XX21RECQ L5A-x0409	Frozen	29.7	20	CI079716	HIN3-170302-01	A	11	c	3M sodium chloride -- 0.1M HEPES pH 7.5	2	Tetramer P 2 2 21 Porous	89.75 149.55 274.95 (90.0 90.0 90.0)	P 2 2 21	4.13	5	Xia2-DIALS	14/05/2020 00:49	-	High Salt	G16T	S7N:L12 C	Non-D69Y Crystal Epitopes	no
G3-004	XX21RECQ L5A-x0407	Frozen	29.7	20	CI079716	HIN3-170302-01	A	10	a	3M sodium chloride -- 0.1M bis-tris pH 6.5	2	Tetramer P 2 2 21 Porous	89.12 152.46 274.68 (90.0 90.0 90.0)	P 2 2 21	4.18	5	Xia2-DIALS	14/05/2020 00:41	-	High Salt	G16T	S7N:L12 C	Non-D69Y Crystal Epitopes	no
G3-004	XX21RECQ L5A-x0412	Frozen	29.7	20	CI079716	HIN3-170302-01	H	6	a	0.2M sodium formate -- 20% PEG3350	NA		NA	NA	NA	0	NA	14/05/2020 02:05	-	High Salt	G16T	S7N:L12 C	Non-D69Y Crystal Epitopes	no
G3-004	XX21RECQ L5A-x0411	Frozen	29.7	20	CI079716	HIN3-170302-01	A	12	d	3M sodium chloride -- 0.1M tris pH 8.5	NA		NA	NA	NA	0	NA	14/05/2020 02:01	-	High Salt	G16T	S7N:L12 C	Non-D69Y Crystal Epitopes	no
G3-004	XX21RECQ L5A-x0410	Frozen	29.7	20	CI079716	HIN3-170302-01	A	11	d	3M sodium chloride -- 0.1M HEPES pH 7.5	NA		NA	NA	NA	0	NA	14/05/2020 00:53	-	High Salt	G16T	S7N:L12 C	Non-D69Y Crystal Epitopes	no
G3-004	XX21RECQ L5A-x0408	Frozen	29.7	20	CI079716	HIN3-170302-01	A	11	a	3M sodium chloride -- 0.1M HEPES pH 7.5	NA		NA	NA	NA	0	NA	14/05/2020 00:45	-	High Salt	G16T	S7N:L12 C	Non-D69Y Crystal Epitopes	no
G3-006	XX21RECQ L5A-x0424	Frozen	27.9045	20	CI079717	HIN3-170302-01	C	12	a	15% tacsimate -- 2% PEG3350 -- 0.1M HEPES pH 7.0	2	Tetramer P 2 2 21 Porous	89.59 153.01 269.83 (90.0 90.0 90.0)	P 2 2 2	3.2	5	Xia2-DIALS	14/05/2020 02:53	-	High Salt	R20K	S7N:L12 C	Non-D69Y Crystal Epitopes	no
G3-006	XX21RECQ L5A-x0426	Frozen	27.9045	20	CI079717	HIN3-170302-01	C	12	d	15% tacsimate -- 2% PEG3350 -- 0.1M HEPES pH 7.0	2	Tetramer P 2 2 21 Porous	89.61 153.42 270.41 (90.0 90.0 90.0)	P 2 2 2	3.24	5	Xia2-DIALS	14/05/2020 03:02	-	High Salt	R20K	S7N:L12 C	Non-D69Y Crystal Epitopes	no
G3-006	XX21RECQ L5A-x0420	Frozen	27.9045	20	CI079717	HIN3-170302-01	B	3	a	0.5M magnesium formate -- 0.1M HEPES pH 7.5	2	Tetramer P 2 2 21 Porous	89.91 153.26 268.79 (90.0 90.0 90.0)	P 2 2 21	3.34	5	Xia2-DIALS	14/05/2020 02:37	-	High Salt	R20K	S7N:L12 C	Non-D69Y Crystal Epitopes	no

Table S4 Diffraction data

Nanobody grouped ID	Mounted Crystal ID	Xtal Plate Protein Frozen/Fresh?	Xtal Plate Protein Concentration (mg/ml)	Xtal Plate Temperature	Xtal Plate Barcode	Screen Batch Name	XTBM Row	XTBM Column	XTBM Subwell	XTBM Condition	Crystal Form Group	Cystal Form Annotation	Unit Cell Dimensions	Space group	Resolution	Number of Successful Autoprocessing Pipelines	Autoprocessing Pipeline Used	Experiment Time	Soaking condition	Xtal Group	Surface Mutations Around Nbinter-1	Key Mutations	Crystal Epitope Mutations	Condition Repitition?
G3-006	XX21RECQ L5A-x0425	Frozen	27.9045	20	CI079717	HIN3-170302-01	C	12	c	15% tacsimate -- 2% PEG3350 -- 0.1M HEPES pH 7.0	2	Tetramer P 2 2 21 Porous	148.6 271.18 89.46 (90.0 90.0 90.0)	P 21 21 2	3.47	5	Xia2-DIALS	14/05/2020 02:57	-	High Salt	R20K	S7N:L12C	Non-D69Y Crystal Epitopes	no
G3-006	XX21RECQ L5A-x0417	Frozen	27.9045	20	CI079717	HIN3-170302-01	A	12	a	3M sodium chloride -- 0.1M tris pH 8.5	2	Tetramer P 2 2 21 Porous	89.47 151.1 273.25 (90.0 90.0 90.0)	P 2 2 2	3.56	5	Xia2-DIALS	14/05/2020 02:25	-	High Salt	R20K	S7N:L12C	Non-D69Y Crystal Epitopes	no
G3-006	XX21RECQ L5A-x0421	Frozen	27.9045	20	CI079717	HIN3-170302-01	B	3	c	0.5M magnesium formate -- 0.1M HEPES pH 7.5	2	Tetramer P 2 2 21 Porous	89.86 150.68 269.38 (90.0 90.0 90.0)	P 2 2 2	3.94	5	Xia2-DIALS	14/05/2020 02:41	-	High Salt	R20K	S7N:L12C	Non-D69Y Crystal Epitopes	no
G3-006	XX21RECQ L5A-x0413	Frozen	27.9045	20	CI079717	HIN3-170302-01	A	9	a	3M sodium chloride -- 0.1M bis-tris pH 5.5	2	Tetramer P 2 2 21 Porous	89.12 148.68 273.98 (90.0 90.0 90.0)	P 2 2 21	3.99	5	Xia2-DIALS	14/05/2020 02:09	-	High Salt	R20K	S7N:L12C	Non-D69Y Crystal Epitopes	no
G3-006	XX21RECQ L5A-x0418	Frozen	27.9045	20	CI079717	HIN3-170302-01	A	12	c	3M sodium chloride -- 0.1M tris pH 8.5	2	Tetramer P 2 2 21 Porous	151.82 271.79 89.44 (90.0 90.0 90.0)	P 21 21 2	4.18	5	Xia2-DIALS	14/05/2020 02:29	-	High Salt	R20K	S7N:L12C	Non-D69Y Crystal Epitopes	no
G3-006	XX21RECQ L5A-x0414	Frozen	27.9045	20	CI079717	HIN3-170302-01	A	10	a	3M sodium chloride -- 0.1M bis-tris pH 6.5	2	Tetramer P 2 2 21 Porous	146.98 274.73 89.31 (90.0 90.0 90.0)	P 21 21 2	4.22	5	Xia2-DIALS	14/05/2020 02:13	-	High Salt	R20K	S7N:L12C	Non-D69Y Crystal Epitopes	no
G3-006	XX21RECQ L5A-x0416	Frozen	27.9045	20	CI079717	HIN3-170302-01	A	11	a	3M sodium chloride -- 0.1M HEPES pH 7.5	2	Tetramer P 2 2 21 Porous	89.87 153.43 272.3 (90.0 90.0 90.0)	P 2 2 21	4.68	4	Xia2-3dii	14/05/2020 02:21	-	High Salt	R20K	S7N:L12C	Non-D69Y Crystal Epitopes	no
G3-006	XX21RECQ L5A-x0415	Frozen	27.9045	20	CI079717	HIN3-170302-01	A	10	c	3M sodium chloride -- 0.1M bis-tris pH 6.5	2	Tetramer P 2 2 21 Porous	89.24 151.24 273.71 (90.0 90.0 90.0)	P 2 2 21	4.78	5	Xia2-DIALS	14/05/2020 02:17	-	High Salt	R20K	S7N:L12C	Non-D69Y Crystal Epitopes	no
G3-006	XX21RECQ L5A-x0423	Frozen	27.9045	20	CI079717	HIN3-170302-01	C	11	d	1M ammonium sulfate -- 0.5% PEG8000 -- 0.1M HEPES pH 7.0	2	Tetramer P 2 2 21 Porous	89.5 150.22 272.48 (90.01 90.05 90.0)	P 21 21 2	4.91	3	Xia2-DIALS	14/05/2020 02:49	-	High Salt	R20K	S7N:L12C	Non-D69Y Crystal Epitopes	no
G3-006	XX21RECQ L5A-x0422	Frozen	27.9045	20	CI079717	HIN3-170302-01	B	3	d	0.5M magnesium formate -- 0.1M HEPES pH 7.5	2	Tetramer P 2 2 21 Porous	90.4 146.73 272.38 (90.0 90.0 90.0)	P 2 2 21	4.98	1	Xia2-DIALS	14/05/2020 02:45	-	High Salt	R20K	S7N:L12C	Non-D69Y Crystal Epitopes	no
G3-006	XX21RECQ L5A-x0419	Frozen	27.9045	20	CI079717	HIN3-170302-01	A	12	d	3M sodium chloride -- 0.1M tris pH 8.5	2	Tetramer P 2 2 21 Porous	279.55 89.86 155.53 (90.0 90.0 90.0)	P 2 2 21	6.89	1	Xia2-3dii	14/05/2020 02:33	-	High Salt	R20K	S7N:L12C	Non-D69Y Crystal Epitopes	no
G3-010	XX21RECQ L5A-x0443	Frozen	33.7815	20	CI079733	HIN3-170302-01	C	11	a	1M ammonium sulfate -- 0.5% PEG8000 -- 0.1M HEPES pH 7.0	2	Tetramer P 2 2 21 Porous	89.23 150.0 272.73 (90.0 90.0 90.0)	P 2 2 2	3.43	5	Xia2-DIALS	14/05/2020 03:06	-	High Salt	N92D	S7N:L12C	Non-D69Y Crystal Epitopes	no
G3-010	XX21RECQ L5A-x0444	Frozen	33.7815	20	CI079733	HIN3-170302-01	C	12	a	15% tacsimate -- 2% PEG3350 -- 0.1M HEPES pH 7.0	2	Tetramer P 2 2 21 Porous	89.51 152.76 270.66 (90.0 90.0 90.0)	P 2 2 2	3.45	5	Xia2-DIALS	14/05/2020 03:10	-	High Salt	N92D	S7N:L12C	Non-D69Y Crystal Epitopes	no
G3-010	XX21RECQ L5A-x0445	Frozen	33.7815	20	CI079733	HIN3-170302-01	C	12	c	15% tacsimate -- 2% PEG3350 -- 0.1M HEPES pH 7.0	2	Tetramer P 2 2 21 Porous	89.79 152.02 272.07 (90.0 90.0 90.0)	P 2 2 2	3.93	5	Xia2-DIALS	14/05/2020 03:14	-	High Salt	N92D	S7N:L12C	Non-D69Y Crystal Epitopes	no
G3-017	XX21RECQ L5A-x0454	Frozen	30.2985	20	CI079735	HIN3-170302-01	C	6	a	1.5M ammonium sulfate -- 0.1M sodium chloride -- 0.1M bis-tris pH 6.5	2	Tetramer P 2 2 21 Porous	152.52 270.62 90.17 (90.0 90.0 90.0)	P 21 21 2	3.67	5	Xia2-DIALS	14/05/2020 03:50	-	High Salt	R20K	S7N:L12C	Non-D69Y Crystal Epitopes	no

Table S4 Diffraction data

Nanobody grouped ID	Mounted Crystal ID	Xtal Plate Protein Frozen/Fresh?	Xtal Plate Protein Concentration (mg/ml)	Xtal Plate Temperature	Xtal Plate Barcode	Screen Batch Name	XTBM Row	XTBM Column	XTBM Subwell	XTBM Condition	Crystal Form Group	Cystal Form Annotation	Unit Cell Dimensions	Space group	Resolution	Number of Successful Autoprocessing Pipelines	Autoprocessing Pipeline Used	Experiment Time	Soaking condition	Xtal Group	Surface Mutations Around Nbinter-1	Key Mutations	Crystal Epitope Mutations	Condition Repitition?
G3-017	XX21RECQ L5A-x0453	Frozen	30.2985	20	CI079735	HIN3-170302-01	C	4	a	35% tacsimate	2	Tetramer P 2 2 21 Porous	92.24 152.58 272.53 (90.0 90.0 90.0)	P 2 2 21	4.07	2	Xia2-DIALS	14/05/2020 03:46	-	High Salt	R20K	S7N:L12C	Non-D69Y Crystal Epitopes	no
G3-017	XX21RECQ L5A-x0456	Frozen	30.2985	20	CI079735	HIN3-170302-01	C	9	a	1.1M sodium malonate -- 0.5% jeffamine ED-2003 -- 0.1M HEPES pH 7.0	2	Tetramer P 2 2 21 Porous	90.91 150.72 269.29 (89.98 90.08 90.25)	P 2 2 21	4.36	2	Xia2-DIALS	14/05/2020 03:55	-	High Salt	R20K	S7N:L12C	Non-D69Y Crystal Epitopes	no
G3-017	XX21RECQ L5A-x0458	Frozen	30.2985	20	CI079735	HIN3-170302-01	C	10	a	1M succinic acid -- 1% PEG2000MME -- 0.1M HEPES pH 7.0	2	Tetramer P 2 2 21 Porous	156.04 90.19 265.12 (90.0 90.04 90.0)	P 2 2 21	4.38	5	Xia2-DIALS	14/05/2020 04:03	-	High Salt	R20K	S7N:L12C	Non-D69Y Crystal Epitopes	no
G3-017	XX21RECQ L5A-x0451	Frozen	30.2985	20	CI079735	HIN3-170302-01	B	7	c	0.056M sodium phosphate monobasic -- 1.344M potassium phosphate dibasic	2	Tetramer P 2 2 21 Porous	156.75 264.3 90.2 (90.0 90.0 90.0)	P 21 21 2	6	1	Xia2-3dii	14/05/2020 03:38	-	High Salt	R20K	S7N:L12C	Non-D69Y Crystal Epitopes	no
G3-017	XX21RECQ L5A-x0446	Frozen	30.2985	20	CI079735	HIN3-170302-01	A	4	a	2M ammonium sulfate -- 0.1M bis-tris pH 6.5	2	Tetramer P 2 2 21 Porous	91.12 152.17 272.44 (90.0 90.0 90.0)	P 2 2 2	NA	1	NA	14/05/2020 03:18	-	High Salt	R20K	S7N:L12C	Non-D69Y Crystal Epitopes	no
G3-017	XX21RECQ L5A-x0459	Frozen	30.2985	20	CI079735	HIN3-170302-01	C	10	c	1M succinic acid -- 1% PEG2000MME -- 0.1M HEPES pH 7.0	NA		NA	NA	NA	0	NA	13/05/2020 22:47	-	High Salt	R20K	S7N:L12C	Non-D69Y Crystal Epitopes	no
G3-017	XX21RECQ L5A-x0457	Frozen	30.2985	20	CI079735	HIN3-170302-01	C	9	c	1.1M sodium malonate -- 0.5% jeffamine ED-2003 -- 0.1M HEPES pH 7.0	NA		NA	NA	NA	0	NA	14/05/2020 03:59	-	High Salt	R20K	S7N:L12C	Non-D69Y Crystal Epitopes	no
G3-017	XX21RECQ L5A-x0452	Frozen	30.2985	20	CI079735	HIN3-170302-01	B	10	c	0.8M succinic acid	NA		NA	NA	NA	0	NA	14/05/2020 03:42	-	High Salt	R20K	S7N:L12C	Non-D69Y Crystal Epitopes	no
G3-017	XX21RECQ L5A-x0450	Frozen	30.2985	20	CI079735	HIN3-170302-01	B	7	a	0.056M sodium phosphate monobasic -- 1.344M potassium phosphate dibasic	NA		NA	NA	NA	0	NA	14/05/2020 03:34	-	High Salt	R20K	S7N:L12C	Non-D69Y Crystal Epitopes	no
G3-017	XX21RECQ L5A-x0449	Frozen	30.2985	20	CI079735	HIN3-170302-01	B	6	d	0.49M sodium phosphate monobasic -- 0.91M potassium phosphate dibasic	NA		NA	NA	NA	0	NA	14/05/2020 03:31	-	High Salt	R20K	S7N:L12C	Non-D69Y Crystal Epitopes	no
G3-017	XX21RECQ L5A-x0448	Frozen	30.2985	20	CI079735	HIN3-170302-01	A	11	a	3M sodium chloride -- 0.1M HEPES pH 7.5	NA		NA	NA	NA	0	NA	14/05/2020 03:27	-	High Salt	R20K	S7N:L12C	Non-D69Y Crystal Epitopes	no
G3-017	XX21RECQ L5A-x0447	Frozen	30.2985	20	CI079735	HIN3-170302-01	A	5	a	2M ammonium sulfate -- 0.1M HEPES pH 7.5	NA		NA	NA	NA	0	NA	14/05/2020 03:23	-	High Salt	R20K	S7N:L12C	Non-D69Y Crystal Epitopes	no
G3-019	XX21RECQ L5A-x0463	Frozen	26.532	20	CI079736	HIN3-170302-01	C	6	a	1.5M ammonium sulfate -- 0.1M sodium chloride -- 0.1M bis-tris pH 6.5	2	Tetramer P 2 2 21 Porous	89.92 149.81 268.88 (90.0 90.0 90.0)	P 2 2 2	4.43	3	Xia2-DIALS	13/05/2020 23:03	-	High Salt	Q90Y	S7N:L12C	Non-D69Y Crystal Epitopes	no
G3-019	XX21RECQ L5A-x0464	Frozen	26.532	20	CI079736	HIN3-170302-01	C	9	a	1.1M sodium malonate -- 0.5% jeffamine ED-2003 -- 0.1M HEPES pH 7.0	-1	NA	91.17 146.13 269.37 (90.15 90.05 89.97)	P 1	6.47	1	Xia2-DIALS	13/05/2020 23:07	-	High Salt	Q90Y	S7N:L12C	Non-D69Y Crystal Epitopes	no
G3-019	XX21RECQ L5A-x0466	Frozen	26.532	20	CI079736	HIN3-170302-01	C	10	c	1M succinic acid -- 1% PEG2000MME -- 0.1M HEPES pH 7.0	NA		NA	NA	NA	0	NA	13/05/2020 23:14	-	High Salt	Q90Y	S7N:L12C	Non-D69Y Crystal Epitopes	no

Table S4 Diffraction data

Nanobody grouped ID	Mounted Crystal ID	Xtal Plate Protein Frozen/Fresh?	Xtal Plate Protein Concentration (mg/ml)	Xtal Plate Temperature	Xtal Plate Barcode	Screen Batch Name	XTBM Row	XTBM Column	XTBM Subwell	XTBM Condition	Crystal Form Group	Cystal Form Annotation	Unit Cell Dimensions	Space group	Resolution	Number of Successful Autoprocessing Pipelines	Autoprocessing Pipeline Used	Experiment Time	Soaking condition	Xtal Group	Surface Mutations Around Nbinter-1	Key Mutations	Crystal Epitope Mutations	Condition Repitition?
G3-019	XX21RECQL5A-x0465	Frozen	26.532	20	CI079736	HIN3-170302-01	C	10	a	1M succinic acid -- 1% PEG2000MME -- 0.1M HEPES pH 7.0	NA		NA	NA	NA	0	NA	13/05/2020 23:11	-	High Salt	Q90Y	S7N:L12C	Non-D69Y Crystal Epitopes	no
G3-019	XX21RECQL5A-x0462	Frozen	26.532	20	CI079736	HIN3-170302-01	C	4	a	35% tacsimate	NA		NA	NA	NA	0	NA	13/05/2020 22:59	-	High Salt	Q90Y	S7N:L12C	Non-D69Y Crystal Epitopes	no
G3-019	XX21RECQL5A-x0461	Frozen	26.532	20	CI079736	HIN3-170302-01	B	6	c	0.49M sodium phosphate monobasic -- 0.91M potassium phosphate dibasic	NA		NA	NA	NA	0	NA	13/05/2020 22:55	-	High Salt	Q90Y	S7N:L12C	Non-D69Y Crystal Epitopes	no
G3-019	XX21RECQL5A-x0460	Frozen	26.532	20	CI079736	HIN3-170302-01	A	4	a	2M ammonium sulfate -- 0.1M bis-tris pH 6.5	NA		NA	NA	NA	0	NA	13/05/2020 22:51	-	High Salt	Q90Y	S7N:L12C	Non-D69Y Crystal Epitopes	no
G3-020	XX21RECQL5A-x0473	Frozen	29.988	20	CI079737	HIN3-170302-01	C	10	a	1M succinic acid -- 1% PEG2000MME -- 0.1M HEPES pH 7.0	2	Tetramer P 2 2 21 Porous	150.64 268.32 90.58 (90.0 90.0 90.0)	P 21 21 2	3.72	5	Xia2-DIALS	14/05/2020 11:35	-	High Salt	Q90H	S7N:L12C	Non-D69Y Crystal Epitopes	no
G3-020	XX21RECQL5A-x0472	Frozen	29.988	20	CI079737	HIN3-170302-01	C	9	a	1.1M sodium malonate -- 0.5% jeffamine ED-2003 -- 0.1M HEPES pH 7.0	2	Tetramer P 2 2 21 Porous	90.72 149.7 267.23 (90.0 90.0 90.0)	P 2 2 21	3.88	4	Xia2-DIALS	14/05/2020 11:31	-	High Salt	Q90H	S7N:L12C	Non-D69Y Crystal Epitopes	no
G3-020	XX21RECQL5A-x0471	Frozen	29.988	20	CI079737	HIN3-170302-01	C	6	c	1.5M ammonium sulfate -- 0.1M sodium chloride -- 0.1M bis-tris pH 6.5	2	Tetramer P 2 2 21 Porous	90.33 146.52 271.09 (90.0 90.0 90.0)	P 2 2 2	4.13	5	Xia2-DIALS	14/05/2020 11:27	-	High Salt	Q90H	S7N:L12C	Non-D69Y Crystal Epitopes	no
G3-020	XX21RECQL5A-x0470	Frozen	29.988	20	CI079737	HIN3-170302-01	C	6	a	1.5M ammonium sulfate -- 0.1M sodium chloride -- 0.1M bis-tris pH 6.5	NA		NA	NA	NA	0	NA	14/05/2020 11:23	-	High Salt	Q90H	S7N:L12C	Non-D69Y Crystal Epitopes	no
G3-020	XX21RECQL5A-x0469	Frozen	29.988	20	CI079737	HIN3-170302-01	C	4	a	35% tacsimate	NA		NA	NA	NA	0	NA	14/05/2020 11:18	-	High Salt	Q90H	S7N:L12C	Non-D69Y Crystal Epitopes	no
G3-020	XX21RECQL5A-x0468	Frozen	29.988	20	CI079737	HIN3-170302-01	A	5	a	2M ammonium sulfate -- 0.1M HEPES pH 7.5	NA		NA	NA	NA	0	NA	15/05/2020 01:28	-	High Salt	Q90H	S7N:L12C	Non-D69Y Crystal Epitopes	no
G3-020	XX21RECQL5A-x0467	Frozen	29.988	20	CI079737	HIN3-170302-01	A	4	a	2M ammonium sulfate -- 0.1M bis-tris pH 6.5	NA		NA	NA	NA	0	NA	15/05/2020 01:24	-	High Salt	Q90H	S7N:L12C	Non-D69Y Crystal Epitopes	no
G3-025	XX21RECQL5A-x0475	Frozen	35.001	20	CI079759	HIN3-170302-01	C	10	a	1M succinic acid -- 1% PEG2000MME -- 0.1M HEPES pH 7.0	NA		NA	NA	NA	0	NA	14/05/2020 11:42	-	High Salt	Q14K	S7N:L12C	Non-D69Y Crystal Epitopes	no
G3-025	XX21RECQL5A-x0474	Frozen	35.001	20	CI079759	HIN3-170302-01	B	12	a	2.8M sodium acetate	NA		NA	NA	NA	0	NA	14/05/2020 11:38	-	High Salt	Q14K	S7N:L12C	Non-D69Y Crystal Epitopes	no
G3-026	XX21RECQL5A-x0479	Frozen	21.3356	20	CI079753	HIN3-170302-01	B	11	a	2.1M DL- malic acid	-1	NA	168.89 221.55 315.03 (90.0 90.0 90.0)	I 2 2 2	3.55	4	Xia2-DIALS	14/05/2020 11:58	-	High Salt	G16T	S7N:L12C	Non-D69Y Crystal Epitopes	no
G3-026	XX21RECQL5A-x0480	Frozen	21.3356	20	CI079753	HIN3-170302-01	C	3	a	2.4M sodium malonate	NA		NA	NA	NA	0	NA	14/05/2020 12:02	-	High Salt	G16T	S7N:L12C	Non-D69Y Crystal Epitopes	no

Table S4 Diffraction data

Nanobody grouped ID	Mounted Crystal ID	Xtal Plate Protein Frozen/Fresh?	Xtal Plate Protein Concentration (mg/ml)	Xtal Plate Temperature	Xtal Plate Barcode	Screen Batch Name	XTBM Row	XTBM Column	XTBM Subwell	XTBM Condition	Crystal Form Group	Cystal Form Annotation	Unit Cell Dimensions	Space group	Resolution	Number of Successful Autoprocessing Pipelines	Autoprocessing Pipeline Used	Experiment Time	Soaking condition	Xtal Group	Surface Mutations Around Nbinter-1	Key Mutations	Crystal Epitope Mutations	Condition Repitition?
G3-026	XX21RECQL5A-x0478	Frozen	21.3356	20	CI079753	HIN3-170302-01	B	7	a	0.056M sodium phosphate monobasic -- 1.344M potassium phosphate dibasic	NA		NA	NA	NA	0	NA	14/05/2020 11:54	-	High Salt	G16T	S7N:L12C	Non-D69Y Crystal Epitopes	no
G3-026	XX21RECQL5A-x0477	Frozen	21.3356	20	CI079753	HIN3-170302-01	B	6	d	0.49M sodium phosphate monobasic -- 0.91M potassium phosphate dibasic	NA		NA	NA	NA	0	NA	14/05/2020 11:50	-	High Salt	G16T	S7N:L12C	Non-D69Y Crystal Epitopes	no
G3-026	XX21RECQL5A-x0476	Frozen	21.3356	20	CI079753	HIN3-170302-01	A	4	a	2M ammonium sulfate -- 0.1M bis-tris pH 6.5	NA		NA	NA	NA	0	NA	14/05/2020 11:46	-	High Salt	G16T	S7N:L12C	Non-D69Y Crystal Epitopes	no
G3-028	XX21RECQL5A-x0499	Frozen	36.513	20	CI079755	HIN3-170302-01	C	6	d	1.5M ammonium sulfate -- 0.1M sodium chloride -- 0.1M bis-tris pH 6.5	2	Tetramer P 2 2 21 Porous	151.88 268.12 90.24 (90.0 90.0 90.0)	P 21 21 2	3.33	5	Xia2-DIALS	14/05/2020 11:11	-	High Salt	R20K	S7N:L12C	Non-D69Y Crystal Epitopes	no
G3-028	XX21RECQL5A-x0494	Frozen	36.513	20	CI079755	HIN3-170302-01	B	10	c	0.8M succinic acid	2	Tetramer P 2 2 21 Porous	90.23 149.36 268.73 (90.0 90.0 90.0)	P 2 2 21	3.56	5	Xia2-DIALS	14/05/2020 10:50	-	High Salt	R20K	S7N:L12C	Non-D69Y Crystal Epitopes	no
G3-028	XX21RECQL5A-x0496	Frozen	36.513	20	CI079755	HIN3-170302-01	B	12	d	2.8M sodium acetate	2	Tetramer P 2 2 21 Porous	150.34 270.57 89.87 (90.0 90.0 90.0)	P 21 21 2	3.66	5	Xia2-DIALS	14/05/2020 10:58	-	High Salt	R20K	S7N:L12C	Non-D69Y Crystal Epitopes	no
G3-028	XX21RECQL5A-x0487	Frozen	36.513	20	CI079755	HIN3-170302-01	A	12	a	3M sodium chloride -- 0.1M tris pH 8.5	2	Tetramer P 2 2 21 Porous	152.8 270.23 90.86 (90.0 90.0 90.0)	P 21 21 2	3.77	5	Xia2-DIALS	14/05/2020 10:21	-	High Salt	R20K	S7N:L12C	Non-D69Y Crystal Epitopes	no
G3-028	XX21RECQL5A-x0488	Frozen	36.513	20	CI079755	HIN3-170302-01	A	12	c	3M sodium chloride -- 0.1M tris pH 8.5	2	Tetramer P 2 2 21 Porous	270.26 90.42 151.28 (90.0 90.0 90.0)	P 21 21 2	3.92	5	Xia2-DIALS	14/05/2020 10:25	-	High Salt	R20K	S7N:L12C	Non-D69Y Crystal Epitopes	no
G3-028	XX21RECQL5A-x0484	Frozen	36.513	20	CI079755	HIN3-170302-01	A	11	a	3M sodium chloride -- 0.1M HEPES pH 7.5	2	Tetramer P 2 2 21 Porous	89.74 146.58 268.93 (90.0 90.0 90.0)	P 2 2 21	3.94	5	Xia2-DIALS	14/05/2020 12:18	-	High Salt	R20K	S7N:L12C	Non-D69Y Crystal Epitopes	no
G3-028	XX21RECQL5A-x0485	Frozen	36.513	20	CI079755	HIN3-170302-01	A	11	c	3M sodium chloride -- 0.1M HEPES pH 7.5	2	Tetramer P 2 2 21 Porous	146.06 266.64 88.96 (90.0 90.0 90.0)	P 21 21 2	4.17	2	Xia2-DIALS	14/05/2020 10:12	-	High Salt	R20K	S7N:L12C	Non-D69Y Crystal Epitopes	no
G3-028	XX21RECQL5A-x0490	Frozen	36.513	20	CI079755	HIN3-170302-01	B	6	a	0.49M sodium phosphate monobasic -- 0.91M potassium phosphate dibasic	2	Tetramer P 2 2 21 Porous	90.17 146.67 269.66 (90.0 90.0 90.0)	P 2 2 21	4.48	4	Xia2-3dii	14/05/2020 10:34	-	High Salt	R20K	S7N:L12C	Non-D69Y Crystal Epitopes	no
G3-028	XX21RECQL5A-x0497	Frozen	36.513	20	CI079755	HIN3-170302-01	C	6	a	1.5M ammonium sulfate -- 0.1M sodium chloride -- 0.1M bis-tris pH 6.5	2	Tetramer P 2 2 21 Porous	152.5 270.76 90.31 (90.0 90.0 90.0)	P 21 21 2	5.27	2	Xia2-DIALS	14/05/2020 11:02	-	High Salt	R20K	S7N:L12C	Non-D69Y Crystal Epitopes	no
G3-028	XX21RECQL5A-x0500	Frozen	36.513	20	CI079755	HIN3-170302-01	C	10	a	1M succinic acid -- 1% PEG2000MME -- 0.1M HEPES pH 7.0	NA		NA	NA	NA	0	NA	14/05/2020 11:15	-	High Salt	R20K	S7N:L12C	Non-D69Y Crystal Epitopes	no
G3-028	XX21RECQL5A-x0498	Frozen	36.513	20	CI079755	HIN3-170302-01	C	6	c	1.5M ammonium sulfate -- 0.1M sodium chloride -- 0.1M bis-tris pH 6.5	NA		NA	NA	NA	0	NA	14/05/2020 11:06	-	High Salt	R20K	S7N:L12C	Non-D69Y Crystal Epitopes	no
G3-028	XX21RECQL5A-x0495	Frozen	36.513	20	CI079755	HIN3-170302-01	B	10	d	0.8M succinic acid	NA		NA	NA	NA	0	NA	14/05/2020 10:54	-	High Salt	R20K	S7N:L12C	Non-D69Y Crystal Epitopes	no

Table S4 Diffraction data

Nanobody grouped ID	Mounted Crystal ID	Xtal Plate Protein Frozen/Fresh?	Xtal Plate Protein Concentration (mg/ml)	Xtal Plate Temperature	Xtal Plate Barcode	Screen Batch Name	XTBM Row	XTBM Column	XTBM Subwell	XTBM Condition	Crystal Form Group	Cystal Form Annotation	Unit Cell Dimensions	Space group	Resolution	Number of Successful Autoprocessing Pipelines	Autoprocessing Pipeline Used	Experiment Time	Soaking condition	Xtal Group	Surface Mutations Around Nbinter-1	Key Mutations	Crystal Epitope Mutations	Condition Repitition?
G3-028	XX21RECQ L5A-x0493	Frozen	36.513	20	CI079755	HIN3-170302-01	B	7	a	0.056M sodium phosphate monobasic -- 1.344M potassium phosphate dibasic	NA		NA	NA	NA	0	NA	14/05/2020 10:46	-	High Salt	R20K	S7N:L12C	Non-D69Y Crystal Epitopes	no
G3-028	XX21RECQ L5A-x0492	Frozen	36.513	20	CI079755	HIN3-170302-01	B	6	d	0.49M sodium phosphate monobasic -- 0.91M potassium phosphate dibasic	NA		NA	NA	NA	0	NA	14/05/2020 10:42	-	High Salt	R20K	S7N:L12C	Non-D69Y Crystal Epitopes	no
G3-028	XX21RECQ L5A-x0491	Frozen	36.513	20	CI079755	HIN3-170302-01	B	6	c	0.49M sodium phosphate monobasic -- 0.91M potassium phosphate dibasic	NA		NA	NA	NA	0	NA	14/05/2020 10:38	-	High Salt	R20K	S7N:L12C	Non-D69Y Crystal Epitopes	no
G3-028	XX21RECQ L5A-x0489	Frozen	36.513	20	CI079755	HIN3-170302-01	A	12	d	3M sodium chloride -- 0.1M tris pH 8.5	NA		NA	NA	NA	0	NA	14/05/2020 10:29	-	High Salt	R20K	S7N:L12C	Non-D69Y Crystal Epitopes	no
G3-028	XX21RECQ L5A-x0486	Frozen	36.513	20	CI079755	HIN3-170302-01	A	11	d	3M sodium chloride -- 0.1M HEPES pH 7.5	NA		NA	NA	NA	0	NA	14/05/2020 10:16	-	High Salt	R20K	S7N:L12C	Non-D69Y Crystal Epitopes	no
G3-028	XX21RECQ L5A-x0483	Frozen	36.513	20	CI079755	HIN3-170302-01	A	5	a	2M ammonium sulfate -- 0.1M HEPES pH 7.5	NA		NA	NA	NA	0	NA	14/05/2020 12:14	-	High Salt	R20K	S7N:L12C	Non-D69Y Crystal Epitopes	no
G3-028	XX21RECQ L5A-x0482	Frozen	36.513	20	CI079755	HIN3-170302-01	A	4	c	2M ammonium sulfate -- 0.1M bis-tris pH 6.5	NA		NA	NA	NA	0	NA	14/05/2020 12:10	-	High Salt	R20K	S7N:L12C	Non-D69Y Crystal Epitopes	no
G3-028	XX21RECQ L5A-x0481	Frozen	36.513	20	CI079755	HIN3-170302-01	A	4	a	2M ammonium sulfate -- 0.1M bis-tris pH 6.5	NA		NA	NA	NA	0	NA	14/05/2020 12:06	-	High Salt	R20K	S7N:L12C	Non-D69Y Crystal Epitopes	no
G3-029	XX21RECQ L5A-x0502	Frozen	34.245	20	CI079756	HIN3-170302-01	A	5	a	2M ammonium sulfate -- 0.1M HEPES pH 7.5	3	Tetramer P 2 2 21 Compact	80.15 90.31 261.34 (90.0 90.0 90.0)	P 2 2 21	3.39	4	Xia2-3dii	14/05/2020 09:12	-	High Salt	Q90R	S7N:L12C	Non-D69Y Crystal Epitopes	no
G3-029	XX21RECQ L5A-x0509	Frozen	34.245	20	CI079756	HIN3-170302-01	B	6	c	0.49M sodium phosphate monobasic -- 0.91M potassium phosphate dibasic	2	Tetramer P 2 2 21 Porous	150.35 268.66 90.26 (90.0 90.0 90.0)	P 21 21 2	3.8	5	Xia2-DIALS	14/05/2020 09:40	-	High Salt	Q90R	S7N:L12C	Non-D69Y Crystal Epitopes	no
G3-029	XX21RECQ L5A-x0511	Frozen	34.245	20	CI079756	HIN3-170302-01	C	6	a	1.5M ammonium sulfate -- 0.1M sodium chloride -- 0.1M bis-tris pH 6.5	2	Tetramer P 2 2 21 Porous	151.36 272.31 90.32 (90.0 90.0 90.0)	P 21 21 2	3.86	5	Xia2-DIALS	14/05/2020 09:48	-	High Salt	Q90R	S7N:L12C	Non-D69Y Crystal Epitopes	no
G3-029	XX21RECQ L5A-x0506	Frozen	34.245	20	CI079756	HIN3-170302-01	A	11	c	3M sodium chloride -- 0.1M HEPES pH 7.5	2	Tetramer P 2 2 21 Porous	90.11 147.56 271.03 (90.0 90.0 90.0)	P 2 2 21	3.94	5	Xia2-DIALS	14/05/2020 09:28	-	High Salt	Q90R	S7N:L12C	Non-D69Y Crystal Epitopes	no
G3-029	XX21RECQ L5A-x0508	Frozen	34.245	20	CI079756	HIN3-170302-01	B	6	a	0.49M sodium phosphate monobasic -- 0.91M potassium phosphate dibasic	2	Tetramer P 2 2 21 Porous	90.48 149.73 269.16 (90.0 90.0 90.0)	P 2 2 2	3.97	3	Xia2-DIALS	14/05/2020 09:36	-	High Salt	Q90R	S7N:L12C	Non-D69Y Crystal Epitopes	no
G3-029	XX21RECQ L5A-x0512	Frozen	34.245	20	CI079756	HIN3-170302-01	C	6	c	1.5M ammonium sulfate -- 0.1M sodium chloride -- 0.1M bis-tris pH 6.5	2	Tetramer P 2 2 21 Porous	90.08 151.78 270.48 (90.0 90.0 90.0)	P 2 2 21	4.18	5	Xia2-DIALS	14/05/2020 09:52	-	High Salt	Q90R	S7N:L12C	Non-D69Y Crystal Epitopes	no
G3-029	XX21RECQ L5A-x0504	Frozen	34.245	20	CI079756	HIN3-170302-01	A	10	a	3M sodium chloride -- 0.1M bis-tris pH 6.5	2	Tetramer P 2 2 21 Porous	144.29 267.01 88.53 (90.0 90.0 90.0)	P 21 21 2	6.15	1	Xia2-3dii	14/05/2020 09:20	-	High Salt	Q90R	S7N:L12C	Non-D69Y Crystal Epitopes	no

Table S4 Diffraction data

Nanobody grouped ID	Mounted Crystal ID	Xtal Plate Protein Frozen/Fresh?	Xtal Plate Protein Concentration (mg/ml)	Xtal Plate Temperature	Xtal Plate Barcode	Screen Batch Name	XTBM Row	XTBM Column	XTBM Subwell	XTBM Condition	Crystal Form Group	Cystal Form Annotation	Unit Cell Dimensions	Space group	Resolution	Number of Successful Autoprocessing Pipelines	Autoprocessing Pipeline Used	Experiment Time	Soaking condition	Xtal Group	Surface Mutations Around Nbinter-1	Key Mutations	Crystal Epitope Mutations	Condition Repitition?
G3-029	XX21RECQ L5A-x0510	Frozen	34.245	20	CI079756	HIN3-170302-01	C	4	a	35% tacsimate	NA		NA	NA	NA	0	NA	14/05/2020 09:44	-	High Salt	Q90R	S7N:L12C	Non-D69Y Crystal Epitopes	no
G3-029	XX21RECQ L5A-x0507	Frozen	34.245	20	CI079756	HIN3-170302-01	A	12	c	3M sodium chloride -- 0.1M tris pH 8.5	NA		NA	NA	NA	0	NA	14/05/2020 09:32	-	High Salt	Q90R	S7N:L12C	Non-D69Y Crystal Epitopes	no
G3-029	XX21RECQ L5A-x0505	Frozen	34.245	20	CI079756	HIN3-170302-01	A	11	a	3M sodium chloride -- 0.1M HEPES pH 7.5	NA		NA	NA	NA	0	NA	14/05/2020 09:24	-	High Salt	Q90R	S7N:L12C	Non-D69Y Crystal Epitopes	no
G3-029	XX21RECQ L5A-x0503	Frozen	34.245	20	CI079756	HIN3-170302-01	A	6	a	2M ammonium sulfate -- 0.1M tris pH 8.5	NA		NA	NA	NA	0	NA	14/05/2020 09:16	-	High Salt	Q90R	S7N:L12C	Non-D69Y Crystal Epitopes	no
G3-029	XX21RECQ L5A-x0501	Frozen	34.245	20	CI079756	HIN3-170302-01	A	3	a	2M ammonium sulfate -- 0.1M bis-tris pH 5.5	NA		NA	NA	NA	0	NA	14/05/2020 09:08	-	High Salt	Q90R	S7N:L12C	Non-D69Y Crystal Epitopes	no
G3-030	XX21RECQ L5A-x0520	Frozen	32.58	20	CI079757	HIN3-170302-01	B	6	a	0.49M sodium phosphate monobasic -- 0.91M potassium phosphate dibasic	2	Tetramer P 2 2 21 Porous	90.12 150.3 270.54 (90.0 90.0 90.0)	P 2 2 2	3.52	5	Xia2-DIALS	14/05/2020 08:14	-	High Salt	Q90Y	S7N:L12C	Non-D69Y Crystal Epitopes	no
G3-030	XX21RECQ L5A-x0523	Frozen	32.58	20	CI079757	HIN3-170302-01	B	12	c	2.8M sodium acetate	2	Tetramer P 2 2 21 Porous	150.06 264.96 90.26 (90.0 90.0 90.0)	P 21 21 2	3.57	4	Xia2-3dii	14/05/2020 08:28	-	High Salt	Q90Y	S7N:L12C	Non-D69Y Crystal Epitopes	no
G3-030	XX21RECQ L5A-x0519	Frozen	32.58	20	CI079757	HIN3-170302-01	A	12	c	3M sodium chloride -- 0.1M tris pH 8.5	2	Tetramer P 2 2 21 Porous	90.02 149.89 271.5 (90.0 90.0 90.0)	P 2 2 2	3.76	5	Xia2-DIALS	14/05/2020 08:10	-	High Salt	Q90Y	S7N:L12C	Non-D69Y Crystal Epitopes	no
G3-030	XX21RECQ L5A-x0525	Frozen	32.58	20	CI079757	HIN3-170302-01	C	4	a	35% tacsimate	2	Tetramer P 2 2 21 Porous	152.2 267.48 90.0 (90.0 90.0 90.0)	P 21 21 2	3.87	5	Xia2-DIALS	14/05/2020 08:36	-	High Salt	Q90Y	S7N:L12C	Non-D69Y Crystal Epitopes	no
G3-030	XX21RECQ L5A-x0518	Frozen	32.58	20	CI079757	HIN3-170302-01	A	12	a	3M sodium chloride -- 0.1M tris pH 8.5	2	Tetramer P 2 2 21 Porous	153.5 271.73 90.18 (90.0 90.0 90.0)	P 21 21 2	4.03	5	Xia2-DIALS	14/05/2020 08:06	-	High Salt	Q90Y	S7N:L12C	Non-D69Y Crystal Epitopes	no
G3-030	XX21RECQ L5A-x0526	Frozen	32.58	20	CI079757	HIN3-170302-01	C	6	a	1.5M ammonium sulfate -- 0.1M sodium chloride -- 0.1M bis-tris pH 6.5	2	Tetramer P 2 2 21 Porous	150.66 273.62 89.7 (90.0 90.0 90.0)	P 21 21 2	4.35	5	Xia2-DIALS	14/05/2020 08:40	-	High Salt	Q90Y	S7N:L12C	Non-D69Y Crystal Epitopes	no
G3-030	XX21RECQ L5A-x0515	Frozen	32.58	20	CI079757	HIN3-170302-01	A	10	a	3M sodium chloride -- 0.1M bis-tris pH 6.5	2	Tetramer P 2 2 21 Porous	89.91 150.42 274.29 (90.0 90.0 90.0)	P 2 2 21	4.37	5	Xia2-DIALS	14/05/2020 10:04	-	High Salt	Q90Y	S7N:L12C	Non-D69Y Crystal Epitopes	no
G3-030	XX21RECQ L5A-x0521	Frozen	32.58	20	CI079757	HIN3-170302-01	B	6	d	0.49M sodium phosphate monobasic -- 0.91M potassium phosphate dibasic	2	Tetramer P 2 2 21 Porous	151.84 268.15 90.08 (90.0 90.0 90.0)	P 21 21 2	4.71	2	Xia2-DIALS	14/05/2020 08:19	-	High Salt	Q90Y	S7N:L12C	Non-D69Y Crystal Epitopes	no
G3-030	XX21RECQ L5A-x0514	Frozen	32.58	20	CI079757	HIN3-170302-01	A	4	a	2M ammonium sulfate -- 0.1M bis-tris pH 6.5	2	Tetramer P 2 2 21 Porous	89.35 148.71 270.78 (90.0 90.0 90.0)	P 2 2 21	5.41	4	Xia2-3dii	14/05/2020 10:00	-	High Salt	Q90Y	S7N:L12C	Non-D69Y Crystal Epitopes	no
G3-030	XX21RECQ L5A-x0513	Frozen	32.58	20	CI079757	HIN3-170302-01	A	3	a	2M ammonium sulfate -- 0.1M bis-tris pH 5.5	2	Tetramer P 2 2 21 Porous	89.75 151.85 271.96 (90.0 90.0 90.0)	P 21 21 21	5.43	5	Xia2-DIALS	14/05/2020 09:56	-	High Salt	Q90Y	S7N:L12C	Non-D69Y Crystal Epitopes	no

Table S4 Diffraction data

Nanobody grouped ID	Mounted Crystal ID	Xtal Plate Protein Frozen/Fresh?	Xtal Plate Protein Concentration (mg/ml)	Xtal Plate Temperature	Xtal Plate Barcode	Screen Batch Name	XTBM Row	XTBM Column	XTBM Subwell	XTBM Condition	Crystal Form Group	Cystal Form Annotation	Unit Cell Dimensions	Space group	Resolution	Number of Successful Autoprocessing Pipelines	Autoprocessing Pipeline Used	Experiment Time	Soaking condition	Xtal Group	Surface Mutations Around Nbinter-1	Key Mutations	Crystal Epitope Mutations	Condition Repitition?
G3-030	XX21RECQ L5A-x0516	Frozen	32.58	20	CI079757	HIN3-170302-01	A	11	a	3M sodium chloride -- 0.1M HEPES pH 7.5	2	Tetramer P 2 2 21 Porous	154.83 276.06 91.27 (90.0 90.0 90.0)	P 21 21 2	6.82	1	Xia2-3dii	14/05/2020 10:08	-	High Salt	Q90Y	S7N:L12C	Non-D69Y Crystal Epitopes	no
G3-030	XX21RECQ L5A-x0527	Frozen	32.58	20	CI079757	HIN3-170302-01	C	6	d	1.5M ammonium sulfate -- 0.1M sodium chloride -- 0.1M bis-tris pH 6.5	NA		NA	NA	NA	0	NA	14/05/2020 08:44	-	High Salt	Q90Y	S7N:L12C	Non-D69Y Crystal Epitopes	no
G3-030	XX21RECQ L5A-x0524	Frozen	32.58	20	CI079757	HIN3-170302-01	C	1	a	3.5M sodium formate	NA		NA	NA	NA	0	NA	14/05/2020 08:32	-	High Salt	Q90Y	S7N:L12C	Non-D69Y Crystal Epitopes	no
G3-030	XX21RECQ L5A-x0522	Frozen	32.58	20	CI079757	HIN3-170302-01	B	10	a	0.8M succinic acid	NA		NA	NA	NA	0	NA	14/05/2020 08:24	-	High Salt	Q90Y	S7N:L12C	Non-D69Y Crystal Epitopes	no
G3-030	XX21RECQ L5A-x0517	Frozen	32.58	20	CI079757	HIN3-170302-01	A	11	c	3M sodium chloride -- 0.1M HEPES pH 7.5	NA		NA	NA	NA	0	NA	14/05/2020 08:02	-	High Salt	Q90Y	S7N:L12C	Non-D69Y Crystal Epitopes	no
G3-031	XX21RECQ L5A-x0272	Frozen	30.888	20	CI079296	HIN3-170302-01	B	10	c	0.8M succinic acid	2	Tetramer P 2 2 21 Porous	90.46 153.3 266.01 (89.98 90.03 89.97)	P 2 21 21	3.2	5	Xia2-DIALS	01/03/2020 05:53	-	High Salt	Q90H	S7N:L12C	Non-D69Y Crystal Epitopes	no
G3-031	XX21RECQ L5A-x0268	Frozen	30.888	20	CI079296	HIN3-170302-01	A	11	a	3M sodium chloride -- 0.1M HEPES pH 7.5	2	Tetramer P 2 2 21 Porous	90.19 153.69 266.73 (90.0 90.0 90.0)	P 2 2 2	3.79	4	Xia2-DIALS	01/03/2020 05:44	-	High Salt	Q90H	S7N:L12C	Non-D69Y Crystal Epitopes	no
G3-031	XX21RECQ L5A-x0274	Frozen	30.888	20	CI079296	HIN3-170302-01	C	6	a	1.5M ammonium sulfate -- 0.1M sodium chloride -- 0.1M bis-tris pH 6.5	2	Tetramer P 2 2 21 Porous	89.66 149.73 269.4 (90.0 90.0 90.0)	P 2 2 2	3.86	2	Xia2-DIALS	01/03/2020 06:45	-	High Salt	Q90H	S7N:L12C	Non-D69Y Crystal Epitopes	no
G3-031	XX21RECQ L5A-x0269	Frozen	30.888	20	CI079296	HIN3-170302-01	A	11	c	3M sodium chloride -- 0.1M HEPES pH 7.5	2	Tetramer P 2 2 21 Porous	90.12 151.14 268.59 (90.0 90.0 90.0)	P 2 21 21	4.06	1	Xia2-DIALS	01/03/2020 05:46	-	High Salt	Q90H	S7N:L12C	Non-D69Y Crystal Epitopes	no
G3-031	XX21RECQ L5A-x0271	Frozen	30.888	20	CI079296	HIN3-170302-01	B	6	c	0.49M sodium phosphate monobasic -- 0.91M potassium phosphate dibasic	2	Tetramer P 2 2 21 Porous	89.82 150.78 266.27 (90.0 90.0 90.0)	P 2 2 2	5.85	2	Autoproc	01/03/2020 05:50	-	High Salt	Q90H	S7N:L12C	Non-D69Y Crystal Epitopes	no
G3-031	XX21RECQ L5A-x0273	Frozen	30.888	20	CI079296	HIN3-170302-01	B	10	d	0.8M succinic acid	2	Tetramer P 2 2 21 Porous	85.75 139.99 254.04 (90.0 90.0 90.0)	P 2 2 2	NA	1	NA	01/03/2020 06:43	-	High Salt	Q90H	S7N:L12C	Non-D69Y Crystal Epitopes	no
G3-031	XX21RECQ L5A-x0270	Frozen	30.888	20	CI079296	HIN3-170302-01	B	6	a	0.49M sodium phosphate monobasic -- 0.91M potassium phosphate dibasic	NA		NA	NA	NA	0	NA	01/03/2020 05:48	-	High Salt	Q90H	S7N:L12C	Non-D69Y Crystal Epitopes	no
G3-046	XX21RECQ L5A-x0276	Frozen	35.01	20	CI079298	HIN3-170302-01	C	3	c	2.4M sodium malonate	-1	NA	89.16 158.6 218.24 (90.0 90.0 90.0)	P 2 2 2	5.47	1	Xia2-3dii	01/03/2020 06:50	-	High Salt	Q14D	S7N:L12C	Non-D69Y Crystal Epitopes	no
G3-046	XX21RECQ L5A-x0275	Frozen	35.01	20	CI079298	HIN3-170302-01	A	4	d	2M ammonium sulfate -- 0.1M bis-tris pH 6.5	NA		NA	NA	NA	0	NA	01/03/2020 06:48	-	High Salt	Q14D	S7N:L12C	Non-D69Y Crystal Epitopes	no
G3-048	XX21RECQ L5A-x0278	Frozen	21.87	20	CI079205	HIN3-170302-01	A	5	c	2M ammonium sulfate -- 0.1M HEPES pH 7.5	3	Tetramer P 2 2 21 Compact	89.58 259.32 79.44 (90.0 90.0 90.0)	P 21 21 2	2.74	5	Xia2-DIALS	01/03/2020 03:48	-	High Salt	G16T	S7N:L12C	Non-D69Y Crystal Epitopes	no

Table S4 Diffraction data

Nanobody grouped ID	Mounted Crystal ID	Xtal Plate Protein Frozen/Fresh?	Xtal Plate Protein Concentration (mg/ml)	Xtal Plate Temperature	Xtal Plate Barcode	Screen Batch Name	XTBM Row	XTBM Column	XTBM Subwell	XTBM Condition	Crystal Form Group	Cystal Form Annotation	Unit Cell Dimensions	Space group	Resolution	Number of Successful Autoprocessing Pipelines	Autoprocessing Pipeline Used	Experiment Time	Soaking condition	Xtal Group	Surface Mutations Around Nbinter-1	Key Mutations	Crystal Epitope Mutations	Condition Repitition?
G3-048	XX21RECQ L5A-x0282	Frozen	21.87	20	CI079205	HIN3-170302-01	B	12	d	2.8M sodium acetate	-1	NA	90.52 150.07 264.7 (89.94 89.85 90.0)	P 1	3.19	2	Xia2-DIALS	01/03/2020 03:57	-	High Salt	G16T	S7N:L12C	Non-D69Y Crystal Epitopes	no
G3-048	XX21RECQ L5A-x0280	Frozen	21.87	20	CI079205	HIN3-170302-01	B	10	c	0.8M succinic acid	-1	NA	151.89 151.89 92.59 (90.0 90.0 120.0)	P 6 2 2	3.33	3	Xia2-DIALS	01/03/2020 03:53	-	High Salt	G16T	S7N:L12C	Non-D69Y Crystal Epitopes	no
G3-048	XX21RECQ L5A-x0277	Frozen	21.87	20	CI079205	HIN3-170302-01	A	4	c	2M ammonium sulfate -- 0.1M bis-tris pH 6.5	-1	NA	90.06 154.89 265.99 (90.02 90.02 90.04)	P 1	5.35	1	Xia2-DIALS	01/03/2020 03:46	-	High Salt	G16T	S7N:L12C	Non-D69Y Crystal Epitopes	no
G3-048	XX21RECQ L5A-x0283	Frozen	21.87	20	CI079205	HIN3-170302-01	C	6	c	1.5M ammonium sulfate -- 0.1M sodium chloride -- 0.1M bis-tris pH 6.5	NA		NA	NA	NA	0	NA	01/03/2020 03:59	-	High Salt	G16T	S7N:L12C	Non-D69Y Crystal Epitopes	no
G3-048	XX21RECQ L5A-x0281	Frozen	21.87	20	CI079205	HIN3-170302-01	B	10	d	0.8M succinic acid	NA		NA	NA	NA	0	NA	01/03/2020 03:55	-	High Salt	G16T	S7N:L12C	Non-D69Y Crystal Epitopes	no
G3-048	XX21RECQ L5A-x0279	Frozen	21.87	20	CI079205	HIN3-170302-01	B	6	a	0.49M sodium phosphate monobasic -- 0.91M potassium phosphate dibasic	NA		NA	NA	NA	0	NA	01/03/2020 03:50	-	High Salt	G16T	S7N:L12C	Non-D69Y Crystal Epitopes	no
G3-050	XX21RECQ L5A-x0037	Frozen	44.82	20	CI079290	HIN3-170302-01	A	11	c	3M sodium chloride -- 0.1M HEPES pH 7.5	2	Tetramer P 2 2 21 Porous	90.15 151.05 268.52 (90.0 90.0 90.0)	P 2 2 21	3.61	3	Xia2-DIALS	01/03/2020 04:25	-	High Salt	R20K	S7N:L12C	Non-D69Y Crystal Epitopes	no
G3-050	XX21RECQ L5A-x0287	Frozen	44.82	20	CI079290	HIN3-170302-01	C	6	c	1.5M ammonium sulfate -- 0.1M sodium chloride -- 0.1M bis-tris pH 6.5	2	Tetramer P 2 2 21 Porous	90.21 149.88 267.94 (90.0 90.0 90.0)	P 2 2 21	5.16	3	Xia2-DIALS	01/03/2020 04:29	-	High Salt	R20K	S7N:L12C	Non-D69Y Crystal Epitopes	no
G3-050	XX21RECQ L5A-x0288	Frozen	44.82	20	CI079290	HIN3-170302-01	G	1	d	0.2M sodium chloride -- 25% PEG3350 -- 0.1M tris pH 8.5	NA		NA	NA	NA	0	NA	01/03/2020 04:32	-	High Salt	R20K	S7N:L12C	Non-D69Y Crystal Epitopes	no
G3-050	XX21RECQ L5A-x0286	Frozen	44.82	20	CI079290	HIN3-170302-01	C	6	a	1.5M ammonium sulfate -- 0.1M sodium chloride -- 0.1M bis-tris pH 6.5	NA		NA	NA	NA	0	NA	01/03/2020 04:27	-	High Salt	R20K	S7N:L12C	Non-D69Y Crystal Epitopes	no
G3-051	XX21RECQ L5A-x0290	Frozen	29.52	20	CI079291	HIN3-170302-01	B	6	a	0.49M sodium phosphate monobasic -- 0.91M potassium phosphate dibasic	-1	NA	90.25 151.07 266.82 (89.99 89.98 90.01)	P 1	4.64	2	Xia2-DIALS	01/03/2020 04:36	-	High Salt	Q90R	S7N:L12C	Non-D69Y Crystal Epitopes	no
G3-051	XX21RECQ L5A-x0289	Frozen	29.52	20	CI079291	HIN3-170302-01	A	10	a	3M sodium chloride -- 0.1M bis-tris pH 6.5	NA		NA	NA	NA	0	NA	01/03/2020 04:34	-	High Salt	Q90R	S7N:L12C	Non-D69Y Crystal Epitopes	no
G3-052	XX21RECQ L5A-x0298	Frozen	35.01	20	CI079292	HIN3-170302-01	C	11	a	1M ammonium sulfate -- 0.5% PEG8000 -- 0.1M HEPES pH 7.0	2	Tetramer P 2 2 21 Porous	150.69 89.46 269.12 (90.0 90.02 90.0)	P 2 21 21	3.03	4	Xia2-DIALS	01/03/2020 05:29	-	High Salt	Q90Y	S7N:L12C	Non-D69Y Crystal Epitopes	no
G3-052	XX21RECQ L5A-x0300	Frozen	35.01	20	CI079292	HIN3-170302-01	C	11	d	1M ammonium sulfate -- 0.5% PEG8000 -- 0.1M HEPES pH 7.0	2	Tetramer P 2 2 21 Porous	151.49 269.4 89.2 (90.0 90.0 90.0)	P 21 21 2	3.52	4	Xia2-DIALS	01/03/2020 05:34	-	High Salt	Q90Y	S7N:L12C	Non-D69Y Crystal Epitopes	no
G3-052	XX21RECQ L5A-x0295	Frozen	35.01	20	CI079292	HIN3-170302-01	C	2	d	1.1M ammonium tartrate	2	Tetramer P 2 2 21 Porous	153.15 269.05 90.18 (90.0 90.0 90.0)	P 21 21 2	3.64	4	Xia2-DIALS	01/03/2020 05:23	-	High Salt	Q90Y	S7N:L12C	Non-D69Y Crystal Epitopes	no

Table S4 Diffraction data

Nanobody grouped ID	Mounted Crystal ID	Xtal Plate Protein Frozen/Fresh?	Xtal Plate Protein Concentration (mg/ml)	Xtal Plate Temperature	Xtal Plate Barcode	Screen Batch Name	XTBM Row	XTBM Column	XTBM Subwell	XTBM Condition	Crystal Form Group	Cystal Form Annotation	Unit Cell Dimensions	Space group	Resolution	Number of Successful Autoprocessing Pipelines	Autoprocessing Pipeline Used	Experiment Time	Soaking condition	Xtal Group	Surface Mutations Around Nbinter-1	Key Mutations	Crystal Epitope Mutations	Condition Repitition?
G3-052	XX21RECQL5A-x0291	Frozen	35.01	20	CI079292	HIN3-170302-01	A	10	a	3M sodium chloride -- 0.1M bis-tris pH 6.5	2	Tetramer P 2 2 21 Porous	151.16 269.88 89.64 (90.0 90.0 90.0)	P 21 21 2	4.11	3	Xia2-DIALS	01/03/2020 04:38	-	High Salt	Q90Y	S7N:L12C	Non-D69Y Crystal Epitopes	no
G3-052	XX21RECQL5A-x0297	Frozen	35.01	20	CI079292	HIN3-170302-01	C	6	a	1.5M ammonium sulfate -- 0.1M sodium chloride -- 0.1M bis-tris pH 6.5	2	Tetramer P 2 2 21 Porous	89.83 151.36 268.68 (90.0 90.0 90.0)	P 2 21 21	4.14	1	Xia2-DIALS	01/03/2020 05:27	-	High Salt	Q90Y	S7N:L12C	Non-D69Y Crystal Epitopes	no
G3-052	XX21RECQL5A-x0293	Frozen	35.01	20	CI079292	HIN3-170302-01	B	6	a	0.49M sodium phosphate monobasic -- 0.91M potassium phosphate dibasic	2	Tetramer P 2 2 21 Porous	268.88 89.89 147.75 (90.0 90.0 90.0)	P 2 2 21	4.43	3	Xia2-DIALS	01/03/2020 04:43	-	High Salt	Q90Y	S7N:L12C	Non-D69Y Crystal Epitopes	no
G3-052	XX21RECQL5A-x0294	Frozen	35.01	20	CI079292	HIN3-170302-01	B	11	a	2.1M DL- malic acid	2	Tetramer P 2 2 21 Porous	148.11 267.42 90.33 (90.0 90.0 90.0)	P 21 21 2	5.12	4	Xia2-DIALS	01/03/2020 05:21	-	High Salt	Q90Y	S7N:L12C	Non-D69Y Crystal Epitopes	no
G3-052	XX21RECQL5A-x0301	Frozen	35.01	20	CI079292	HIN3-170302-01	G	3	d	0.2M lithium sulfate -- 25% PEG3350 -- 0.1M bis-tris pH 6.5	NA		NA	NA	NA	0	NA	01/03/2020 05:36	-	High Salt	Q90Y	S7N:L12C	Non-D69Y Crystal Epitopes	no
G3-052	XX21RECQL5A-x0299	Frozen	35.01	20	CI079292	HIN3-170302-01	C	11	c	1M ammonium sulfate -- 0.5% PEG8000 -- 0.1M HEPES pH 7.0	NA		NA	NA	NA	0	NA	01/03/2020 05:32	-	High Salt	Q90Y	S7N:L12C	Non-D69Y Crystal Epitopes	no
G3-052	XX21RECQL5A-x0296	Frozen	35.01	20	CI079292	HIN3-170302-01	C	4	a	35% tacsimate	NA		NA	NA	NA	0	NA	01/03/2020 05:25	-	High Salt	Q90Y	S7N:L12C	Non-D69Y Crystal Epitopes	no
G3-052	XX21RECQL5A-x0292	Frozen	35.01	20	CI079292	HIN3-170302-01	B	3	a	0.5M magnesium formate -- 0.1M HEPES pH 7.5	NA		NA	NA	NA	0	NA	01/03/2020 04:40	-	High Salt	Q90Y	S7N:L12C	Non-D69Y Crystal Epitopes	no
G3-053	XX21RECQL5A-x0302	Frozen	33.03	20	CI079293	HIN3-170302-01	B	6	a	0.49M sodium phosphate monobasic -- 0.91M potassium phosphate dibasic	2	Tetramer P 2 2 21 Porous	90.21 152.11 266.85 (90.0 90.0 90.0)	P 2 21 21	3.6	4	Xia2-DIALS	01/03/2020 05:38	-	High Salt	Q90H	S7N:L12C	Non-D69Y Crystal Epitopes	no
G3-053	XX21RECQL5A-x0304	Frozen	33.03	20	CI079293	HIN3-170302-01	C	6	a	1.5M ammonium sulfate -- 0.1M sodium chloride -- 0.1M bis-tris pH 6.5	NA		NA	NA	NA	0	NA	01/03/2020 05:42	-	High Salt	Q90H	S7N:L12C	Non-D69Y Crystal Epitopes	no
G3-053	XX21RECQL5A-x0303	Frozen	33.03	20	CI079293	HIN3-170302-01	C	1	d	3.5M sodium formate	NA		NA	NA	NA	0	NA	01/03/2020 05:40	-	High Salt	Q90H	S7N:L12C	Non-D69Y Crystal Epitopes	no
G3-054	XX21RECQL5A-x0306	Frozen	34.29	20	CI079218	HIN3-170302-01	C	2	a	1.1M ammonium tartrate	2	Tetramer P 2 2 21 Porous	143.43 268.82 89.69 (90.0 90.0 90.0)	P 21 21 2	3.19	2	Xia2-DIALS	01/03/2020 04:03	-	High Salt	N92D	S7N:L12C	Non-D69Y Crystal Epitopes	no
G3-054	XX21RECQL5A-x0310	Frozen	34.29	20	CI079218	HIN3-170302-01	C	11	a	1M ammonium sulfate -- 0.5% PEG8000 -- 0.1M HEPES pH 7.0	2	Tetramer P 2 2 21 Porous	89.78 149.15 269.87 (90.0 90.0 90.0)	P 2 2 21	3.63	4	Xia2-DIALS	01/03/2020 03:02	-	High Salt	N92D	S7N:L12C	Non-D69Y Crystal Epitopes	no
G3-054	XX21RECQL5A-x0307	Frozen	34.29	20	CI079218	HIN3-170302-01	C	2	d	1.1M ammonium tartrate	2	Tetramer P 2 2 21 Porous	89.82 150.13 268.29 (90.0 90.0 90.0)	P 2 21 21	3.97	1	Xia2-DIALS	01/03/2020 04:06	-	High Salt	N92D	S7N:L12C	Non-D69Y Crystal Epitopes	no
G3-054	XX21RECQL5A-x0308	Frozen	34.29	20	CI079218	HIN3-170302-01	C	6	a	1.5M ammonium sulfate -- 0.1M sodium chloride -- 0.1M bis-tris pH 6.5	2	Tetramer P 2 2 21 Porous	90.02 143.84 271.23 (90.0 90.0 90.0)	P 2 21 21	4.04	1	Xia2-DIALS	01/03/2020 04:08	-	High Salt	N92D	S7N:L12C	Non-D69Y Crystal Epitopes	no

Table S4 Diffraction data

Nanobody grouped ID	Mounted Crystal ID	Xtal Plate Protein Frozen/Fresh?	Xtal Plate Protein Concentration (mg/ml)	Xtal Plate Temperature	Xtal Plate Barcode	Screen Batch Name	XTBM Row	XTBM Column	XTBM Subwell	XTBM Condition	Crystal Form Group	Cystal Form Annotation	Unit Cell Dimensions	Space group	Resolution	Number of Successful Autoprocessing Pipelines	Autoprocessing Pipeline Used	Experiment Time	Soaking condition	Xtal Group	Surface Mutations Around Nbinter-1	Key Mutations	Crystal Epitope Mutations	Condition Repitition?
G3-054	XX21RECQ L5A-x0309	Frozen	34.29	20	CI079218	HIN3-170302-01	C	7	a	0.8M sodium/potassium tartrate -- 0.5% PEG5000MME -- 0.1M tris pH 8.5	-1	NA	90.06 148.56 265.59 (90.09 89.98 90.04)	P 1	6.03	1	Xia2-DIALS	01/03/2020 04:10	-	High Salt	N92D	S7N:L12C	Non-D69Y Crystal Epitopes	no
G3-054	XX21RECQ L5A-x0311	Frozen	34.29	20	CI079218	HIN3-170302-01	C	11	c	1M ammonium sulfate -- 0.5% PEG8000 -- 0.1M HEPES pH 7.0	NA		NA	NA	NA	0	NA	01/03/2020 03:05	-	High Salt	N92D	S7N:L12C	Non-D69Y Crystal Epitopes	no
G3-054	XX21RECQ L5A-x0305	Frozen	34.29	20	CI079218	HIN3-170302-01	A	11	a	3M sodium chloride -- 0.1M HEPES pH 7.5	NA		NA	NA	NA	0	NA	01/03/2020 04:01	-	High Salt	N92D	S7N:L12C	Non-D69Y Crystal Epitopes	no
G3-055	XX21RECQ L5A-x0313	Frozen	36.63	20	CI079204	HIN3-170302-01	C	3	a	2.4M sodium malonate	-1	NA	89.49 159.88 220.97 (90.0 90.0 90.0)	P 2 2 21	3.63	5	Xia2-DIALS	01/03/2020 03:44	-	High Salt	L12N	S7N	Non-D69Y Crystal Epitopes	no
G3-055	XX21RECQ L5A-x0312	Frozen	36.63	20	CI079204	HIN3-170302-01	B	12	d	2.8M sodium acetate	NA		NA	NA	NA	0	NA	01/03/2020 03:41	-	High Salt	L12N	S7N	Non-D69Y Crystal Epitopes	no
G3-061	XX21RECQ L5A-x0316	Frozen	36	20	CI079240	HIN3-170302-01	A	5	c	2M ammonium sulfate -- 0.1M HEPES pH 7.5	3	Tetramer P 2 2 21 Compact	79.27 89.71 260.27 (90.0 90.0 90.0)	P 2 2 21	2.95	3	Xia2-DIALS	01/03/2020 03:11	-	High Salt	R20K	S7N:L12C	Non-D69Y Crystal Epitopes	no
G3-061	XX21RECQ L5A-x0315	Frozen	36	20	CI079240	HIN3-170302-01	A	5	a	2M ammonium sulfate -- 0.1M HEPES pH 7.5	3	Tetramer P 2 2 21 Compact	79.28 89.58 259.61 (90.0 90.0 90.0)	P 2 2 21	3.82	3	Xia2-DIALS	01/03/2020 03:09	-	High Salt	R20K	S7N:L12C	Non-D69Y Crystal Epitopes	no
G3-061	XX21RECQ L5A-x0317	Frozen	36	20	CI079240	HIN3-170302-01	A	11	a	3M sodium chloride -- 0.1M HEPES pH 7.5	-1	NA	89.85 150.76 268.48 (89.99 89.97 89.96)	P 1	5.1	1	Xia2-DIALS	01/03/2020 03:13	-	High Salt	R20K	S7N:L12C	Non-D69Y Crystal Epitopes	no
G3-061	XX21RECQ L5A-x0320	Frozen	36	20	CI079240	HIN3-170302-01	C	6	c	1.5M ammonium sulfate -- 0.1M sodium chloride -- 0.1M bis-tris pH 6.5	NA		NA	NA	NA	0	NA	01/03/2020 03:20	-	High Salt	R20K	S7N:L12C	Non-D69Y Crystal Epitopes	no
G3-061	XX21RECQ L5A-x0319	Frozen	36	20	CI079240	HIN3-170302-01	C	6	a	1.5M ammonium sulfate -- 0.1M sodium chloride -- 0.1M bis-tris pH 6.5	NA		NA	NA	NA	0	NA	01/03/2020 03:18	-	High Salt	R20K	S7N:L12C	Non-D69Y Crystal Epitopes	no
G3-061	XX21RECQ L5A-x0318	Frozen	36	20	CI079240	HIN3-170302-01	A	12	a	3M sodium chloride -- 0.1M tris pH 8.5	NA		NA	NA	NA	0	NA	01/03/2020 03:16	-	High Salt	R20K	S7N:L12C	Non-D69Y Crystal Epitopes	no
G3-061	XX21RECQ L5A-x0314	Frozen	36	20	CI079240	HIN3-170302-01	A	4	a	2M ammonium sulfate -- 0.1M bis-tris pH 6.5	NA		NA	NA	NA	0	NA	01/03/2020 03:07	-	High Salt	R20K	S7N:L12C	Non-D69Y Crystal Epitopes	no
G3-062	XX21RECQ L5A-x0325	Frozen	30.6	20	CI079241	HIN3-170302-01	B	6	a	0.49M sodium phosphate monobasic -- 0.91M potassium phosphate dibasic	2	Tetramer P 2 2 21 Porous	152.65 268.72 89.83 (90.0 90.0 90.0)	P 21 21 2	3.57	3	Xia2-DIALS	01/03/2020 03:31	-	High Salt	Q90R	S7N:L12C	Non-D69Y Crystal Epitopes	no
G3-062	XX21RECQ L5A-x0321	Frozen	30.6	20	CI079241	HIN3-170302-01	A	10	a	3M sodium chloride -- 0.1M bis-tris pH 6.5	2	Tetramer P 2 2 21 Porous	90.14 152.79 270.84 (90.0 90.0 90.0)	P 2 2 2	4.29	5	Xia2-DIALS	01/03/2020 03:22	-	High Salt	Q90R	S7N:L12C	Non-D69Y Crystal Epitopes	no
G3-062	XX21RECQ L5A-x0324	Frozen	30.6	20	CI079241	HIN3-170302-01	A	11	d	3M sodium chloride -- 0.1M HEPES pH 7.5	2	Tetramer P 2 2 21 Porous	89.97 150.01 269.51 (90.0 90.0 90.0)	P 2 2 2	4.98	2	Xia2-3dii	01/03/2020 03:29	-	High Salt	Q90R	S7N:L12C	Non-D69Y Crystal Epitopes	no

Table S4 Diffraction data

Nanobody grouped ID	Mounted Crystal ID	Xtal Plate Protein Frozen/Fresh?	Xtal Plate Protein Concentration (mg/ml)	Xtal Plate Temperature	Xtal Plate Barcode	Screen Batch Name	XTBM Row	XTBM Column	XTBM Subwell	XTBM Condition	Crystal Form Group	Cystal Form Annotation	Unit Cell Dimensions	Space group	Resolution	Number of Successful Autoprocessing Pipelines	Autoprocessing Pipeline Used	Experiment Time	Soaking condition	Xtal Group	Surface Mutations Around Nbinter-1	Key Mutations	Crystal Epitope Mutations	Condition Repitition?
G3-062	XX21RECQ L5A-x0326	Frozen	30.6	20	CI079241	HIN3-170302-01	H	6	c	0.2M sodium formate -- 20% PEG3350	NA		NA	NA	NA	0	NA	01/03/2020 03:33	-	PEG	Q90R	S7N:L12C	Non-D69Y Crystal Epitopes	no
G3-062	XX21RECQ L5A-x0323	Frozen	30.6	20	CI079241	HIN3-170302-01	A	11	c	3M sodium chloride -- 0.1M HEPES pH 7.5	NA		NA	NA	NA	0	NA	01/03/2020 03:26	-	High Salt	Q90R	S7N:L12C	Non-D69Y Crystal Epitopes	no
G3-062	XX21RECQ L5A-x0322	Frozen	30.6	20	CI079241	HIN3-170302-01	A	11	a	3M sodium chloride -- 0.1M HEPES pH 7.5	NA		NA	NA	NA	0	NA	01/03/2020 03:24	-	High Salt	Q90R	S7N:L12C	Non-D69Y Crystal Epitopes	no
G3-063	XX21RECQ L5A-x0328	Frozen	40.5	20	CI079242	HIN3-170302-01	A	11	c	3M sodium chloride -- 0.1M HEPES pH 7.5	-1	NA	157.16 157.16 91.0 (90.0 90.0 120.0)	P 6 2 2	3.19	3	Xia2-DIALS	01/03/2020 04:12	-	High Salt	Q90Y	S7N:L12C	Non-D69Y Crystal Epitopes	no
G3-063	XX21RECQ L5A-x0330	Frozen	40.5	20	CI079242	HIN3-170302-01	B	6	a	0.49M sodium phosphate monobasic -- 0.91M potassium phosphate dibasic	2	Tetramer P 2 2 21 Porous	150.94 268.13 89.8 (90.0 90.0 90.0)	P 21 21 2	3.79	5	Xia2-DIALS	01/03/2020 04:17	-	High Salt	Q90Y	S7N:L12C	Non-D69Y Crystal Epitopes	no
G3-063	XX21RECQ L5A-x0332	Frozen	40.5	20	CI079242	HIN3-170302-01	C	6	a	1.5M ammonium sulfate -- 0.1M sodium chloride -- 0.1M bis-tris pH 6.5	2	Tetramer P 2 2 21 Porous	150.85 270.45 89.56 (90.0 90.0 90.0)	P 21 21 2	3.82	3	Xia2-DIALS	01/03/2020 04:21	-	High Salt	Q90Y	S7N:L12C	Non-D69Y Crystal Epitopes	no
G3-063	XX21RECQ L5A-x0327	Frozen	40.5	20	CI079242	HIN3-170302-01	A	11	a	3M sodium chloride -- 0.1M HEPES pH 7.5	2	Tetramer P 2 2 21 Porous	89.71 150.96 269.6 (90.0 90.0 90.0)	P 2 21 21	3.91	1	Xia2-DIALS	01/03/2020 03:35	-	High Salt	Q90Y	S7N:L12C	Non-D69Y Crystal Epitopes	no
G3-063	XX21RECQ L5A-x0331	Frozen	40.5	20	CI079242	HIN3-170302-01	B	6	c	0.49M sodium phosphate monobasic -- 0.91M potassium phosphate dibasic	-1	NA	90.16 151.31 269.08 (89.93 90.02 89.9)	P 1	4.33	2	Xia2-DIALS	01/03/2020 04:19	-	High Salt	Q90Y	S7N:L12C	Non-D69Y Crystal Epitopes	no
G3-063	XX21RECQ L5A-x0333	Frozen	40.5	20	CI079242	HIN3-170302-01	F	8	a	0.2M ammonium sulfate -- 25% PEG3350 -- 0.1M HEPES pH 7.5	NA		NA	NA	NA	0	NA	01/03/2020 04:23	-	PEG	Q90Y	S7N:L12C	Non-D69Y Crystal Epitopes	no
G3-063	XX21RECQ L5A-x0329	Frozen	40.5	20	CI079242	HIN3-170302-01	A	11	d	3M sodium chloride -- 0.1M HEPES pH 7.5	NA		NA	NA	NA	0	NA	01/03/2020 04:15	-	High Salt	Q90Y	S7N:L12C	Non-D69Y Crystal Epitopes	no
G3-070	XX21RECQ L5A-x0532	Frozen	21.6	20	CI079703	HIN3-170302-01	B	6	a	0.49M sodium phosphate monobasic -- 0.91M potassium phosphate dibasic	2	Tetramer P 2 2 21 Porous	90.15 152.38 268.64 (90.0 90.0 90.0)	P 2 21 21	4.74	1	Xia2-DIALS	15/05/2020 00:18	-	High Salt	G16T	S7N:L12C	Non-D69Y Crystal Epitopes	no
G3-070	XX21RECQ L5A-x0533	Frozen	21.6	20	CI079703	HIN3-170302-01	B	6	c	0.49M sodium phosphate monobasic -- 0.91M potassium phosphate dibasic	2	Tetramer P 2 2 21 Porous	90.53 150.92 268.8 (90.0 90.0 90.0)	P 2 21 21	5.48	1	Xia2-DIALS	15/05/2020 00:22	-	High Salt	G16T	S7N:L12C	Non-D69Y Crystal Epitopes	no
G3-070	XX21RECQ L5A-x0540	Frozen	21.6	20	CI079703	HIN3-170302-01	C	10	a	1M succinic acid -- 1% PEG2000MME -- 0.1M HEPES pH 7.0	NA		NA	NA	NA	0	NA	14/05/2020 12:37	-	High Salt	G16T	S7N:L12C	Non-D69Y Crystal Epitopes	no
G3-070	XX21RECQ L5A-x0539	Frozen	21.6	20	CI079703	HIN3-170302-01	C	6	c	1.5M ammonium sulfate -- 0.1M sodium chloride -- 0.1M bis-tris pH 6.5	NA		NA	NA	NA	0	NA	14/05/2020 12:33	-	High Salt	G16T	S7N:L12C	Non-D69Y Crystal Epitopes	no
G3-070	XX21RECQ L5A-x0538	Frozen	21.6	20	CI079703	HIN3-170302-01	C	6	a	1.5M ammonium sulfate -- 0.1M sodium chloride -- 0.1M bis-tris pH 6.5	NA		NA	NA	NA	0	NA	14/05/2020 12:29	-	High Salt	G16T	S7N:L12C	Non-D69Y Crystal Epitopes	no

Table S4 Diffraction data

Nanobody grouped ID	Mounted Crystal ID	Xtal Plate Protein Frozen/Fresh?	Xtal Plate Protein Concentration (mg/ml)	Xtal Plate Temperature	Xtal Plate Barcode	Screen Batch Name	XTBM Row	XTBM Column	XTBM Subwell	XTBM Condition	Crystal Form Group	Cystal Form Annotation	Unit Cell Dimensions	Space group	Resolution	Number of Successful Autoprocessing Pipelines	Autoprocessing Pipeline Used	Experiment Time	Soaking condition	Xtal Group	Surface Mutations Around Nbinter-1	Key Mutations	Crystal Epitope Mutations	Condition Repitition?
G3-070	XX21RECQ L5A-x0537	Frozen	21.6	20	CI079703	HIN3-170302-01	C	4	a	35% tacsimate	NA		NA	NA	NA	0	NA	14/05/2020 12:26	-	High Salt	G16T	S7N:L12C	Non-D69Y Crystal Epitopes	no
G3-070	XX21RECQ L5A-x0536	Frozen	21.6	20	CI079703	HIN3-170302-01	B	10	d	0.8M succinic acid	NA		NA	NA	NA	0	NA	14/05/2020 12:22	-	High Salt	G16T	S7N:L12C	Non-D69Y Crystal Epitopes	no
G3-070	XX21RECQ L5A-x0535	Frozen	21.6	20	CI079703	HIN3-170302-01	B	10	c	0.8M succinic acid	NA		NA	NA	NA	0	NA	15/05/2020 00:30	-	High Salt	G16T	S7N:L12C	Non-D69Y Crystal Epitopes	no
G3-070	XX21RECQ L5A-x0534	Frozen	21.6	20	CI079703	HIN3-170302-01	B	7	d	0.056M sodium phosphate monobasic -- 1.344M potassium phosphate dibasic	NA		NA	NA	NA	0	NA	15/05/2020 00:26	-	High Salt	G16T	S7N:L12C	Non-D69Y Crystal Epitopes	no
G3-070	XX21RECQ L5A-x0531	Frozen	21.6	20	CI079703	HIN3-170302-01	A	12	a	3M sodium chloride -- 0.1M tris pH 8.5	NA		NA	NA	NA	0	NA	15/05/2020 00:14	-	High Salt	G16T	S7N:L12C	Non-D69Y Crystal Epitopes	no
G3-070	XX21RECQ L5A-x0530	Frozen	21.6	20	CI079703	HIN3-170302-01	A	11	a	3M sodium chloride -- 0.1M HEPES pH 7.5	NA		NA	NA	NA	0	NA	15/05/2020 00:10	-	High Salt	G16T	S7N:L12C	Non-D69Y Crystal Epitopes	no
G3-070	XX21RECQ L5A-x0529	Frozen	21.6	20	CI079703	HIN3-170302-01	A	5	a	2M ammonium sulfate -- 0.1M HEPES pH 7.5	NA		NA	NA	NA	0	NA	15/05/2020 00:07	-	High Salt	G16T	S7N:L12C	Non-D69Y Crystal Epitopes	no
G3-070	XX21RECQ L5A-x0528	Frozen	21.6	20	CI079703	HIN3-170302-01	A	4	a	2M ammonium sulfate -- 0.1M bis-tris pH 6.5	NA		NA	NA	NA	0	NA	15/05/2020 00:03	-	High Salt	G16T	S7N:L12C	Non-D69Y Crystal Epitopes	no
G3-072	XX21RECQ L5A-x0541	Frozen	29.151	20	CI079705	HIN3-170302-01	A	10	a	3M sodium chloride -- 0.1M bis-tris pH 6.5	2	Tetramer P 2 2 21 Porous	152.29 272.31 90.01 (90.0 90.0 90.0)	P 21 21 2	3.94	5	Xia2-DIALS	14/05/2020 12:41	-	High Salt	R20K	S7N:L12C	Non-D69Y Crystal Epitopes	no
G3-072	XX21RECQ L5A-x0542	Frozen	29.151	20	CI079705	HIN3-170302-01	A	10	c	3M sodium chloride -- 0.1M bis-tris pH 6.5	2	Tetramer P 2 2 21 Porous	89.97 153.19 271.13 (90.0 90.0 90.0)	P 2 2 2	3.99	5	Xia2-DIALS	14/05/2020 12:46	-	High Salt	R20K	S7N:L12C	Non-D69Y Crystal Epitopes	no
G3-072	XX21RECQ L5A-x0547	Frozen	29.151	20	CI079705	HIN3-170302-01	A	12	d	3M sodium chloride -- 0.1M tris pH 8.5	2	Tetramer P 2 2 21 Porous	149.22 272.36 90.22 (90.0 90.0 90.0)	P 21 21 2	4.07	5	Xia2-DIALS	14/05/2020 13:05	-	High Salt	R20K	S7N:L12C	Non-D69Y Crystal Epitopes	no
G3-072	XX21RECQ L5A-x0546	Frozen	29.151	20	CI079705	HIN3-170302-01	A	12	c	3M sodium chloride -- 0.1M tris pH 8.5	2	Tetramer P 2 2 21 Porous	90.69 148.92 273.62 (90.0 90.0 90.0)	P 2 2 21	4.44	2	Xia2-DIALS	14/05/2020 13:01	-	High Salt	R20K	S7N:L12C	Non-D69Y Crystal Epitopes	no
G3-072	XX21RECQ L5A-x0551	Frozen	29.151	20	CI079705	HIN3-170302-01	B	12	d	2.8M sodium acetate	NA		NA	NA	NA	0	NA	14/05/2020 13:21	-	High Salt	R20K	S7N:L12C	Non-D69Y Crystal Epitopes	no
G3-072	XX21RECQ L5A-x0550	Frozen	29.151	20	CI079705	HIN3-170302-01	B	6	d	0.49M sodium phosphate monobasic -- 0.91M potassium phosphate dibasic	NA		NA	NA	NA	0	NA	14/05/2020 13:17	-	High Salt	R20K	S7N:L12C	Non-D69Y Crystal Epitopes	no
G3-072	XX21RECQ L5A-x0549	Frozen	29.151	20	CI079705	HIN3-170302-01	B	6	c	0.49M sodium phosphate monobasic -- 0.91M potassium phosphate dibasic	NA		NA	NA	NA	0	NA	14/05/2020 13:13	-	High Salt	R20K	S7N:L12C	Non-D69Y Crystal Epitopes	no

Table S4 Diffraction data

Nanobody grouped ID	Mounted Crystal ID	Xtal Plate Protein Frozen/Fresh?	Xtal Plate Protein Concentration (mg/ml)	Xtal Plate Temperature	Xtal Plate Barcode	Screen Batch Name	XTBM Row	XTBM Column	XTBM Subwell	XTBM Condition	Crystal Form Group	Cystal Form Annotation	Unit Cell Dimensions	Space group	Resolution	Number of Successful Autoprocessing Pipelines	Autoprocessing Pipeline Used	Experiment Time	Soaking condition	Xtal Group	Surface Mutations Around Nbinter-1	Key Mutations	Crystal Epitope Mutations	Condition Repitition?
G3-072	XX21RECQL5A-x0548	Frozen	29.151	20	CI079705	HIN3-170302-01	B	6	a	0.49M sodium phosphate monobasic -- 0.91M potassium phosphate dibasic	NA		NA	NA	NA	0	NA	14/05/2020 13:09	-	High Salt	R20K	S7N:L12C	Non-D69Y Crystal Epitopes	no
G3-072	XX21RECQL5A-x0545	Frozen	29.151	20	CI079705	HIN3-170302-01	A	11	d	3M sodium chloride -- 0.1M HEPES pH 7.5	NA		NA	NA	NA	0	NA	14/05/2020 12:57	-	High Salt	R20K	S7N:L12C	Non-D69Y Crystal Epitopes	no
G3-072	XX21RECQL5A-x0544	Frozen	29.151	20	CI079705	HIN3-170302-01	A	11	c	3M sodium chloride -- 0.1M HEPES pH 7.5	NA		NA	NA	NA	0	NA	14/05/2020 12:53	-	High Salt	R20K	S7N:L12C	Non-D69Y Crystal Epitopes	no
G3-072	XX21RECQL5A-x0543	Frozen	29.151	20	CI079705	HIN3-170302-01	A	11	a	3M sodium chloride -- 0.1M HEPES pH 7.5	NA		NA	NA	NA	0	NA	14/05/2020 12:49	-	High Salt	R20K	S7N:L12C	Non-D69Y Crystal Epitopes	no
G3-073	XX21RECQL5A-x0553	Frozen	32.9445	20	CI079706	HIN3-170302-01	A	10	a	3M sodium chloride -- 0.1M bis-tris pH 6.5	2	Tetramer P 2 2 21 Porous	153.23 273.28 90.09 (90.0 90.0 90.0)	P 21 21 2	4.12	5	Xia2-DIALS	14/05/2020 06:04	-	High Salt	Q90R	S7N:L12C	Non-D69Y Crystal Epitopes	no
G3-073	XX21RECQL5A-x0554	Frozen	32.9445	20	CI079706	HIN3-170302-01	A	10	c	3M sodium chloride -- 0.1M bis-tris pH 6.5	2	Tetramer P 2 2 21 Porous	89.5 152.26 270.71 (90.0 90.0 90.0)	P 2 2 2	4.39	3	Xia2-DIALS	14/05/2020 06:08	-	High Salt	Q90R	S7N:L12C	Non-D69Y Crystal Epitopes	no
G3-073	XX21RECQL5A-x0556	Frozen	32.9445	20	CI079706	HIN3-170302-01	A	11	c	3M sodium chloride -- 0.1M HEPES pH 7.5	NA		NA	NA	NA	0	NA	14/05/2020 06:16	-	High Salt	Q90R	S7N:L12C	Non-D69Y Crystal Epitopes	no
G3-073	XX21RECQL5A-x0555	Frozen	32.9445	20	CI079706	HIN3-170302-01	A	11	a	3M sodium chloride -- 0.1M HEPES pH 7.5	NA		NA	NA	NA	0	NA	14/05/2020 06:13	-	High Salt	Q90R	S7N:L12C	Non-D69Y Crystal Epitopes	no
G3-073	XX21RECQL5A-x0552	Frozen	32.9445	20	CI079706	HIN3-170302-01	A	9	a	3M sodium chloride -- 0.1M bis-tris pH 5.5	NA		NA	NA	NA	0	NA	14/05/2020 06:00	-	High Salt	Q90R	S7N:L12C	Non-D69Y Crystal Epitopes	no
G3-075	XX21RECQL5A-x0557	Frozen	30.6135	20	CI079707	HIN3-170302-01	B	6	a	0.49M sodium phosphate monobasic -- 0.91M potassium phosphate dibasic	2	Tetramer P 2 2 21 Porous	90.59 150.48 269.18 (90.0 90.0 90.0)	P 2 2 2	3.38	5	Xia2-DIALS	14/05/2020 06:21	-	High Salt	Q90H	S7N:L12C	Non-D69Y Crystal Epitopes	no
G3-075	XX21RECQL5A-x0558	Frozen	30.6135	20	CI079707	HIN3-170302-01	B	6	c	0.49M sodium phosphate monobasic -- 0.91M potassium phosphate dibasic	2	Tetramer P 2 2 21 Porous	151.97 268.75 90.88 (90.0 90.0 90.0)	P 21 21 2	3.58	4	Xia2-DIALS	14/05/2020 06:25	-	High Salt	Q90H	S7N:L12C	Non-D69Y Crystal Epitopes	no
G3-075	XX21RECQL5A-x0562	Frozen	30.6135	20	CI079707	HIN3-170302-01	C	6	a	1.5M ammonium sulfate -- 0.1M sodium chloride -- 0.1M bis-tris pH 6.5	2	Tetramer P 2 2 21 Porous	89.63 150.13 273.73 (90.0 90.0 90.0)	P 2 2 21	5.91	4	Xia2-DIALS	14/05/2020 06:40	-	High Salt	Q90H	S7N:L12C	Non-D69Y Crystal Epitopes	no
G3-075	XX21RECQL5A-x0564	Frozen	30.6135	20	CI079707	HIN3-170302-01	F	7	d	0.2M ammonium sulfate -- 25% PEG3350 -- 0.1M bis-tris pH 6.5	NA		NA	NA	NA	0	NA	14/05/2020 06:48	-	PEG	Q90H	S7N:L12C	Non-D69Y Crystal Epitopes	no
G3-075	XX21RECQL5A-x0563	Frozen	30.6135	20	CI079707	HIN3-170302-01	C	6	c	1.5M ammonium sulfate -- 0.1M sodium chloride -- 0.1M bis-tris pH 6.5	NA		NA	NA	NA	0	NA	14/05/2020 06:44	-	High Salt	Q90H	S7N:L12C	Non-D69Y Crystal Epitopes	no
G3-075	XX21RECQL5A-x0561	Frozen	30.6135	20	CI079707	HIN3-170302-01	C	4	a	35% tacsimate	NA		NA	NA	NA	0	NA	14/05/2020 06:36	-	High Salt	Q90H	S7N:L12C	Non-D69Y Crystal Epitopes	no

Table S4 Diffraction data

Nanobody grouped ID	Mounted Crystal ID	Xtal Plate Protein Frozen/Fresh?	Xtal Plate Protein Concentration (mg/ml)	Xtal Plate Temperature	Xtal Plate Barcode	Screen Batch Name	XTBM Row	XTBM Column	XTBM Subwell	XTBM Condition	Crystal Form Group	Cystal Form Annotation	Unit Cell Dimensions	Space group	Resolution	Number of Successful Autoprocessing Pipelines	Autoprocessing Pipeline Used	Experiment Time	Soaking condition	Xtal Group	Surface Mutations Around Nbinter-1	Key Mutations	Crystal Epitope Mutations	Condition Repitition?
G3-075	XX21RECQL5A-x0560	Frozen	30.6135	20	CI079707	HIN3-170302-01	B	10	a	0.8M succinic acid	NA		NA	NA	NA	0	NA	14/05/2020 06:32	-	High Salt	Q90H	S7N:L12C	Non-D69Y Crystal Epitopes	no
G3-075	XX21RECQL5A-x0559	Frozen	30.6135	20	CI079707	HIN3-170302-01	B	6	d	0.49M sodium phosphate monobasic -- 0.91M potassium phosphate dibasic	NA		NA	NA	NA	0	NA	14/05/2020 06:28	-	High Salt	Q90H	S7N:L12C	Non-D69Y Crystal Epitopes	no
G3-076	XX21RECQL5A-x0566	Frozen	38.412	20	CI079708	HIN3-170302-01	A	10	c	3M sodium chloride -- 0.1M bis-tris pH 6.5	2	Tetramer P 2 2 21 Porous	152.71 271.81 90.01 (90.0 90.0 90.0)	P 21 21 2	3.94	5	Xia2-DIALS	14/05/2020 06:56	-	High Salt	N92D	S7N:L12C	Non-D69Y Crystal Epitopes	no
G3-076	XX21RECQL5A-x0565	Frozen	38.412	20	CI079708	HIN3-170302-01	A	10	a	3M sodium chloride -- 0.1M bis-tris pH 6.5	2	Tetramer P 2 2 21 Porous	89.81 147.65 271.53 (90.0 90.0 90.0)	P 2 2 21	5.88	1	Xia2-3dii	14/05/2020 06:52	-	High Salt	N92D	S7N:L12C	Non-D69Y Crystal Epitopes	no
G3-076	XX21RECQL5A-x0569	Frozen	38.412	20	CI079708	HIN3-170302-01	A	12	d	3M sodium chloride -- 0.1M tris pH 8.5	2	Tetramer P 2 2 21 Porous	89.92 144.93 273.69 (90.02 89.89 90.13)	P 21 21 2	7.82	4	Xia2-DIALS	14/05/2020 04:07	-	High Salt	N92D	S7N:L12C	Non-D69Y Crystal Epitopes	no
G3-076	XX21RECQL5A-x0572	Frozen	38.412	20	CI079708	HIN3-170302-01	H	6	a	0.2M sodium formate -- 20% PEG3350	NA		NA	NA	NA	0	NA	14/05/2020 04:19	-	High Salt	N92D	S7N:L12C	Non-D69Y Crystal Epitopes	no
G3-076	XX21RECQL5A-x0571	Frozen	38.412	20	CI079708	HIN3-170302-01	C	12	d	15% tacsimate -- 2% PEG3350 -- 0.1M HEPES pH 7.0	NA		NA	NA	NA	0	NA	14/05/2020 04:14	-	High Salt	N92D	S7N:L12C	Non-D69Y Crystal Epitopes	no
G3-076	XX21RECQL5A-x0570	Frozen	38.412	20	CI079708	HIN3-170302-01	C	6	d	1.5M ammonium sulfate -- 0.1M sodium chloride -- 0.1M bis-tris pH 6.5	NA		NA	NA	NA	0	NA	14/05/2020 04:11	-	High Salt	N92D	S7N:L12C	Non-D69Y Crystal Epitopes	no
G3-076	XX21RECQL5A-x0567	Frozen	38.412	20	CI079708	HIN3-170302-01	A	11	d	3M sodium chloride -- 0.1M HEPES pH 7.5	NA		NA	NA	NA	0	NA	14/05/2020 07:00	-	High Salt	N92D	S7N:L12C	Non-D69Y Crystal Epitopes	no
G3-077	XX21RECQL5A-x0573	Frozen	36.3465	20	CI079709	HIN3-170302-01	B	7	c	0.056M sodium phosphate monobasic -- 1.344M potassium phosphate dibasic	-1	NA	116.26 256.63 179.04 (90.0 90.0 90.0)	C 2 2 21	6.24	5	Xia2-DIALS	13/05/2020 23:51	-	High Salt	G8D	S7N:L12C	Non-D69Y Crystal Epitopes	no
G3-082	XX21RECQL5A-x0580	Frozen	31.671	20	CI079702	HIN3-170302-01	B	6	c	0.49M sodium phosphate monobasic -- 0.91M potassium phosphate dibasic	2	Tetramer P 2 2 21 Porous	90.1 152.68 269.0 (90.0 90.0 90.0)	P 2 2 2	3.26	5	Xia2-DIALS	13/05/2020 23:43	-	High Salt	R20K	S7N:L12C	Non-D69Y Crystal Epitopes	no
G3-082	XX21RECQL5A-x0586	Frozen	31.671	20	CI079702	HIN3-170302-01	C	1	a	3.5M sodium formate	2	Tetramer P 2 2 21 Porous	154.19 265.58 90.32 (90.0 90.0 90.0)	P 21 21 2	3.8	4	Xia2-DIALS	14/05/2020 01:13	-	High Salt	R20K	S7N:L12C	Non-D69Y Crystal Epitopes	no
G3-082	XX21RECQL5A-x0587	Frozen	31.671	20	CI079702	HIN3-170302-01	C	6	a	1.5M ammonium sulfate -- 0.1M sodium chloride -- 0.1M bis-tris pH 6.5	2	Tetramer P 2 2 21 Porous	151.21 272.41 89.87 (90.0 90.0 90.0)	P 21 21 2	3.92	5	Xia2-DIALS	14/05/2020 01:17	-	High Salt	R20K	S7N:L12C	Non-D69Y Crystal Epitopes	no
G3-082	XX21RECQL5A-x0584	Frozen	31.671	20	CI079702	HIN3-170302-01	B	10	c	0.8M succinic acid	2	Tetramer P 2 2 21 Porous	146.77 270.12 90.14 (90.0 90.0 90.0)	P 21 21 2	3.94	5	Xia2-DIALS	14/05/2020 01:05	-	High Salt	R20K	S7N:L12C	Non-D69Y Crystal Epitopes	no
G3-082	XX21RECQL5A-x0585	Frozen	31.671	20	CI079702	HIN3-170302-01	B	12	d	2.8M sodium acetate	2	Tetramer P 2 2 21 Porous	90.47 150.14 266.82 (90.0 90.0 90.0)	P 2 2 21	4.83	3	Xia2-3dii	14/05/2020 01:09	-	High Salt	R20K	S7N:L12C	Non-D69Y Crystal Epitopes	no

Table S4 Diffraction data

Nanobody grouped ID	Mounted Crystal ID	Xtal Plate Protein Frozen/Fresh?	Xtal Plate Protein Concentration (mg/ml)	Xtal Plate Temperature	Xtal Plate Barcode	Screen Batch Name	XTBM Row	XTBM Column	XTBM Subwell	XTBM Condition	Crystal Form Group	Cystal Form Annotation	Unit Cell Dimensions	Space group	Resolution	Number of Successful Autoprocessing Pipelines	Autoprocessing Pipeline Used	Experiment Time	Soaking condition	Xtal Group	Surface Mutations Around Nbinter-1	Key Mutations	Crystal Epitope Mutations	Condition Repitition?
G3-082	XX21RECQ L5A-x0574	Frozen	31.671	20	CI079702	HIN3-170302-01	A	3	a	2M ammonium sulfate -- 0.1M bis-tris pH 5.5	2	Tetramer P 2 2 21 Porous	89.83 152.49 270.02 (90.0 90.0 90.0)	P 2 2 2	5.54	3	Xia2-DIALS	13/05/2020 23:19	-	High Salt	R20K	S7N:L12C	Non-D69Y Crystal Epitopes	no
G3-082	XX21RECQ L5A-x0588	Frozen	31.671	20	CI079702	HIN3-170302-01	H	6	a	0.2M sodium formate -- 20% PEG3350	2	Tetramer P 2 2 21 Porous	89.99 144.61 267.2 (90.0 90.0 90.0)	P 2 2 21	6.56	1	Xia2-3dii	14/05/2020 01:21	-	PEG	R20K	S7N:L12C	Non-D69Y Crystal Epitopes	no
G3-082	XX21RECQ L5A-x0583	Frozen	31.671	20	CI079702	HIN3-170302-01	B	7	c	0.056M sodium phosphate monobasic -- 1.344M potassium phosphate dibasic	-1	NA	151.37 90.58 272.72 (90.0 90.61 90.0)	P 1 2 1	7.04	1	Xia2-3dii	14/05/2020 01:01	-	High Salt	R20K	S7N:L12C	Non-D69Y Crystal Epitopes	no
G3-082	XX21RECQ L5A-x0579	Frozen	31.671	20	CI079702	HIN3-170302-01	A	12	c	3M sodium chloride -- 0.1M tris pH 8.5	2	Tetramer P 2 2 21 Porous	90.23 149.21 271.25 (90.0 90.0 90.0)	P 2 2 2	NA	1	NA	13/05/2020 23:39	-	High Salt	R20K	S7N:L12C	Non-D69Y Crystal Epitopes	no
G3-082	XX21RECQ L5A-x0582	Frozen	31.671	20	CI079702	HIN3-170302-01	B	7	a	0.056M sodium phosphate monobasic -- 1.344M potassium phosphate dibasic	NA		NA	NA	NA	0	NA	14/05/2020 00:57	-	High Salt	R20K	S7N:L12C	Non-D69Y Crystal Epitopes	no
G3-082	XX21RECQ L5A-x0581	Frozen	31.671	20	CI079702	HIN3-170302-01	B	6	d	0.49M sodium phosphate monobasic -- 0.91M potassium phosphate dibasic	NA		NA	NA	NA	0	NA	13/05/2020 23:47	-	High Salt	R20K	S7N:L12C	Non-D69Y Crystal Epitopes	no
G3-082	XX21RECQ L5A-x0578	Frozen	31.671	20	CI079702	HIN3-170302-01	A	12	a	3M sodium chloride -- 0.1M tris pH 8.5	NA		NA	NA	NA	0	NA	13/05/2020 23:35	-	High Salt	R20K	S7N:L12C	Non-D69Y Crystal Epitopes	no
G3-082	XX21RECQ L5A-x0577	Frozen	31.671	20	CI079702	HIN3-170302-01	A	11	c	3M sodium chloride -- 0.1M HEPES pH 7.5	NA		NA	NA	NA	0	NA	13/05/2020 23:31	-	High Salt	R20K	S7N:L12C	Non-D69Y Crystal Epitopes	no
G3-082	XX21RECQ L5A-x0576	Frozen	31.671	20	CI079702	HIN3-170302-01	A	11	a	3M sodium chloride -- 0.1M HEPES pH 7.5	NA		NA	NA	NA	0	NA	13/05/2020 23:27	-	High Salt	R20K	S7N:L12C	Non-D69Y Crystal Epitopes	no
G3-082	XX21RECQ L5A-x0575	Frozen	31.671	20	CI079702	HIN3-170302-01	A	4	a	2M ammonium sulfate -- 0.1M bis-tris pH 6.5	NA		NA	NA	NA	0	NA	13/05/2020 23:23	-	High Salt	R20K	S7N:L12C	Non-D69Y Crystal Epitopes	no
G3-084	XX21RECQ L5A-x0592	Frozen	28.1655	20	CI079719	HIN3-170302-01	C	6	a	1.5M ammonium sulfate -- 0.1M sodium chloride -- 0.1M bis-tris pH 6.5	2	Tetramer P 2 2 21 Porous	90.51 149.87 271.05 (90.0 90.0 90.0)	P 2 2 21	3.76	5	Xia2-DIALS	15/05/2020 01:13	-	High Salt	Q90Y	S7N:L12C	Non-D69Y Crystal Epitopes	no
G3-084	XX21RECQ L5A-x0593	Frozen	28.1655	20	CI079719	HIN3-170302-01	C	6	c	1.5M ammonium sulfate -- 0.1M sodium chloride -- 0.1M bis-tris pH 6.5	2	Tetramer P 2 2 21 Porous	90.08 145.31 271.36 (90.0 90.0 90.0)	P 2 2 2	4.29	2	Xia2-DIALS	15/05/2020 01:17	-	High Salt	Q90Y	S7N:L12C	Non-D69Y Crystal Epitopes	no
G3-084	XX21RECQ L5A-x0594	Frozen	28.1655	20	CI079719	HIN3-170302-01	C	9	a	1.1M sodium malonate -- 0.5% jeffamine ED-2003 -- 0.1M HEPES pH 7.0	-1	NA	91.31 147.8 270.68 (90.0 90.03 90.12)	P 1	4.99	1	Xia2-DIALS	15/05/2020 01:21	-	High Salt	Q90Y	S7N:L12C	Non-D69Y Crystal Epitopes	no
G3-084	XX21RECQ L5A-x0591	Frozen	28.1655	20	CI079719	HIN3-170302-01	A	5	a	2M ammonium sulfate -- 0.1M HEPES pH 7.5	NA		NA	NA	NA	0	NA	15/05/2020 01:09	-	High Salt	Q90Y	S7N:L12C	Non-D69Y Crystal Epitopes	no
G3-084	XX21RECQ L5A-x0590	Frozen	28.1655	20	CI079719	HIN3-170302-01	A	4	c	2M ammonium sulfate -- 0.1M bis-tris pH 6.5	NA		NA	NA	NA	0	NA	15/05/2020 01:05	-	High Salt	Q90Y	S7N:L12C	Non-D69Y Crystal Epitopes	no

Table S4 Diffraction data

Nanobody grouped ID	Mounted Crystal ID	Xtal Plate Protein Frozen/Fresh?	Xtal Plate Protein Concentration (mg/ml)	Xtal Plate Temperature	Xtal Plate Barcode	Screen Batch Name	XTBM Row	XTBM Column	XTBM Subwell	XTBM Condition	Crystal Form Group	Cystal Form Annotation	Unit Cell Dimensions	Space group	Resolution	Number of Successful Autoprocessing Pipelines	Autoprocessing Pipeline Used	Experiment Time	Soaking condition	Xtal Group	Surface Mutations Around Nbinter-1	Key Mutations	Crystal Epitope Mutations	Condition Repitition?
G3-084	XX21RECQ L5A-x0589	Frozen	28.1655	20	CI079719	HIN3-170302-01	A	4	a	2M ammonium sulfate -- 0.1M bis-tris pH 6.5	NA		NA	NA	NA	0	NA	15/05/2020 01:01	-	High Salt	Q90Y	S7N:L12C	Non-D69Y Crystal Epitopes	no
G3-085	XX21RECQ L5A-x0602	Frozen	35.748	20	CI079721	HIN3-170302-01	B	6	c	0.49M sodium phosphate monobasic -- 0.91M potassium phosphate dibasic	2	Tetramer P 2 2 21 Porous	152.2 268.75 90.45 (90.0 90.0 90.0)	P 21 21 2	3.3	5	Xia2-DIALS	14/05/2020 01:53	-	High Salt	Q90H	S7N:L12C	Non-D69Y Crystal Epitopes	no
G3-085	XX21RECQ L5A-x0598	Frozen	35.748	20	CI079721	HIN3-170302-01	A	11	a	3M sodium chloride -- 0.1M HEPES pH 7.5	2	Tetramer P 2 2 21 Porous	152.83 268.56 90.58 (90.0 90.0 90.0)	P 21 21 2	3.47	5	Xia2-DIALS	14/05/2020 01:37	-	High Salt	Q90H	S7N:L12C	Non-D69Y Crystal Epitopes	no
G3-085	XX21RECQ L5A-x0599	Frozen	35.748	20	CI079721	HIN3-170302-01	A	11	c	3M sodium chloride -- 0.1M HEPES pH 7.5	2	Tetramer P 2 2 21 Porous	151.3 269.29 90.47 (90.0 90.0 90.0)	P 21 21 2	3.54	5	Xia2-DIALS	14/05/2020 01:41	-	High Salt	Q90H	S7N:L12C	Non-D69Y Crystal Epitopes	no
G3-085	XX21RECQ L5A-x0609	Frozen	35.748	20	CI079721	HIN3-170302-01	C	6	a	1.5M ammonium sulfate -- 0.1M sodium chloride -- 0.1M bis-tris pH 6.5	2	Tetramer P 2 2 21 Porous	147.6 272.55 90.2 (90.0 90.0 90.0)	P 21 21 2	3.55	5	Xia2-DIALS	15/05/2020 00:49	-	High Salt	Q90H	S7N:L12C	Non-D69Y Crystal Epitopes	no
G3-085	XX21RECQ L5A-x0600	Frozen	35.748	20	CI079721	HIN3-170302-01	A	12	a	3M sodium chloride -- 0.1M tris pH 8.5	2	Tetramer P 2 2 21 Porous	154.82 268.16 90.49 (90.0 90.0 90.0)	P 21 21 2	3.66	5	Xia2-DIALS	14/05/2020 01:45	-	High Salt	Q90H	S7N:L12C	Non-D69Y Crystal Epitopes	no
G3-085	XX21RECQ L5A-x0608	Frozen	35.748	20	CI079721	HIN3-170302-01	C	4	a	35% tacsimate	2	Tetramer P 2 2 21 Porous	151.78 268.23 90.74 (90.0 90.0 90.0)	P 21 21 2	3.7	5	Xia2-DIALS	15/05/2020 00:46	-	High Salt	Q90H	S7N:L12C	Non-D69Y Crystal Epitopes	no
G3-085	XX21RECQ L5A-x0611	Frozen	35.748	20	CI079721	HIN3-170302-01	C	10	a	1M succinic acid -- 1% PEG2000MME -- 0.1M HEPES pH 7.0	2	Tetramer P 2 2 21 Porous	152.46 267.56 90.44 (90.0 90.0 90.0)	P 21 21 2	3.88	5	Xia2-DIALS	15/05/2020 00:57	-	High Salt	Q90H	S7N:L12C	Non-D69Y Crystal Epitopes	no
G3-085	XX21RECQ L5A-x0605	Frozen	35.748	20	CI079721	HIN3-170302-01	B	10	c	0.8M succinic acid	-1	NA	90.67 152.6 268.12 (89.9 89.93 89.87)	P 1	4.26	1	Xia2-DIALS	15/05/2020 00:34	-	High Salt	Q90H	S7N:L12C	Non-D69Y Crystal Epitopes	no
G3-085	XX21RECQ L5A-x0595	Frozen	35.748	20	CI079721	HIN3-170302-01	A	3	a	2M ammonium sulfate -- 0.1M bis-tris pH 5.5	2	Tetramer P 2 2 21 Porous	90.25 145.32 273.46 (90.0 90.0 90.0)	P 2 2 21	5.5	1	Xia2-DIALS	14/05/2020 01:25	-	High Salt	Q90H	S7N:L12C	Non-D69Y Crystal Epitopes	no
G3-085	XX21RECQ L5A-x0596	Frozen	35.748	20	CI079721	HIN3-170302-01	A	4	a	2M ammonium sulfate -- 0.1M bis-tris pH 6.5	2	Tetramer P 2 2 21 Porous	274.25 91.17 149.48 (90.0 90.0 90.0)	P 2 2 21	7.28	1	Xia2-3dii	14/05/2020 01:29	-	High Salt	Q90H	S7N:L12C	Non-D69Y Crystal Epitopes	no
G3-085	XX21RECQ L5A-x0607	Frozen	35.748	20	CI079721	HIN3-170302-01	B	12	d	2.8M sodium acetate	-1	NA	90.79 150.94 265.52 (90.17 90.0 89.75)	P 1	7.33	1	Xia2-DIALS	15/05/2020 00:42	-	High Salt	Q90H	S7N:L12C	Non-D69Y Crystal Epitopes	no
G3-085	XX21RECQ L5A-x0610	Frozen	35.748	20	CI079721	HIN3-170302-01	C	6	c	1.5M ammonium sulfate -- 0.1M sodium chloride -- 0.1M bis-tris pH 6.5	NA		NA	NA	NA	0	NA	15/05/2020 00:53	-	High Salt	Q90H	S7N:L12C	Non-D69Y Crystal Epitopes	no
G3-085	XX21RECQ L5A-x0606	Frozen	35.748	20	CI079721	HIN3-170302-01	B	10	d	0.8M succinic acid	NA		NA	NA	NA	0	NA	15/05/2020 00:38	-	High Salt	Q90H	S7N:L12C	Non-D69Y Crystal Epitopes	no
G3-085	XX21RECQ L5A-x0603	Frozen	35.748	20	CI079721	HIN3-170302-01	B	6	d	0.49M sodium phosphate monobasic -- 0.91M potassium phosphate dibasic	NA		NA	NA	NA	0	NA	14/05/2020 01:57	-	High Salt	Q90H	S7N:L12C	Non-D69Y Crystal Epitopes	no

Table S4 Diffraction data

Nanobody grouped ID	Mounted Crystal ID	Xtal Plate Protein Frozen/Fresh?	Xtal Plate Protein Concentration (mg/ml)	Xtal Plate Temperature	Xtal Plate Barcode	Screen Batch Name	XTBM Row	XTBM Column	XTBM Subwell	XTBM Condition	Crystal Form Group	Cystal Form Annotation	Unit Cell Dimensions	Space group	Resolution	Number of Successful Autoprocessing Pipelines	Autoprocessing Pipeline Used	Experiment Time	Soaking condition	Xtal Group	Surface Mutations Around Nbinter-1	Key Mutations	Crystal Epitope Mutations	Condition Repitition?
G3-085	XX21RECQ L5A-x0601	Frozen	35.748	20	CI079721	HIN3-170302-01	A	12	c	3M sodium chloride -- 0.1M tris pH 8.5	NA		NA	NA	NA	0	NA	14/05/2020 01:49	-	High Salt	Q90H	S7N:L12C	Non-D69Y Crystal Epitopes	no
G3-085	XX21RECQ L5A-x0597	Frozen	35.748	20	CI079721	HIN3-170302-01	A	6	a	2M ammonium sulfate -- 0.1M tris pH 8.5	NA		NA	NA	NA	0	NA	14/05/2020 01:33	-	High Salt	Q90H	S7N:L12C	Non-D69Y Crystal Epitopes	no
G3-086	XX21RECQ L5A-x0617	Frozen	35.2215	20	CI079428	HIN3-170302-01	C	10	c	1M succinic acid -- 1% PEG2000MME -- 0.1M HEPES pH 7.0	2	Tetramer P 2 2 21 Porous	90.12 146.9 268.98 (90.0 90.0 90.0)	P 2 2 2	3.31	3	Xia2-DIALS	14/05/2020 04:44	-	High Salt	N92D	S7N:L12C	Non-D69Y Crystal Epitopes	no
G3-086	XX21RECQ L5A-x0615	Frozen	35.2215	20	CI079428	HIN3-170302-01	C	9	d	1.1M sodium malonate -- 0.5% jeffamine ED-2003 -- 0.1M HEPES pH 7.0	2	Tetramer P 2 2 21 Porous	90.25 145.38 268.47 (90.0 90.0 90.0)	P 2 2 21	3.48	5	Xia2-DIALS	14/05/2020 04:36	-	High Salt	N92D	S7N:L12C	Non-D69Y Crystal Epitopes	no
G3-086	XX21RECQ L5A-x0614	Frozen	35.2215	20	CI079428	HIN3-170302-01	C	9	c	1.1M sodium malonate -- 0.5% jeffamine ED-2003 -- 0.1M HEPES pH 7.0	2	Tetramer P 2 2 21 Porous	90.69 148.29 268.92 (90.0 90.0 90.0)	P 2 2 2	3.67	5	Xia2-DIALS	14/05/2020 04:32	-	High Salt	N92D	S7N:L12C	Non-D69Y Crystal Epitopes	no
G3-086	XX21RECQ L5A-x0613	Frozen	35.2215	20	CI079428	HIN3-170302-01	C	9	a	1.1M sodium malonate -- 0.5% jeffamine ED-2003 -- 0.1M HEPES pH 7.0	2	Tetramer P 2 2 21 Porous	90.36 144.08 269.25 (90.0 90.0 90.0)	P 2 2 21	3.94	2	Xia2-DIALS	14/05/2020 04:27	-	High Salt	N92D	S7N:L12C	Non-D69Y Crystal Epitopes	no
G3-086	XX21RECQ L5A-x0620	Frozen	35.2215	20	CI079428	HIN3-170302-01	E	8	c	0.2M potassium chloride -- 35% pentaerythritol propoxylate 5/4 -- 0.1M HEPES pH 7.5	NA		NA	NA	NA	0	NA	14/05/2020 04:56	-	High Salt	N92D	S7N:L12C	Non-D69Y Crystal Epitopes	no
G3-086	XX21RECQ L5A-x0619	Frozen	35.2215	20	CI079428	HIN3-170302-01	E	8	a	0.2M potassium chloride -- 35% pentaerythritol propoxylate 5/4 -- 0.1M HEPES pH 7.5	NA		NA	NA	NA	0	NA	14/05/2020 04:52	-	High Salt	N92D	S7N:L12C	Non-D69Y Crystal Epitopes	no
G3-086	XX21RECQ L5A-x0618	Frozen	35.2215	20	CI079428	HIN3-170302-01	C	10	d	1M succinic acid -- 1% PEG2000MME -- 0.1M HEPES pH 7.0	NA		NA	NA	NA	0	NA	14/05/2020 04:48	-	High Salt	N92D	S7N:L12C	Non-D69Y Crystal Epitopes	no
G3-086	XX21RECQ L5A-x0616	Frozen	35.2215	20	CI079428	HIN3-170302-01	C	10	a	1M succinic acid -- 1% PEG2000MME -- 0.1M HEPES pH 7.0	NA		NA	NA	NA	0	NA	14/05/2020 04:40	-	High Salt	N92D	S7N:L12C	Non-D69Y Crystal Epitopes	no
G3-086	XX21RECQ L5A-x0612	Frozen	35.2215	20	CI079428	HIN3-170302-01	B	9	d	1.8M ammonium citrate	NA		NA	NA	NA	0	NA	14/05/2020 04:23	-	High Salt	N92D	S7N:L12C	Non-D69Y Crystal Epitopes	no
G3-089	XX21RECQ L5A-x0622	Frozen	35.6535	20	CI079738	HIN3-170302-01	A	11	d	3M sodium chloride -- 0.1M HEPES pH 7.5	-1	NA	89.89 153.65 273.07 (90.0 90.06 90.0)	P 1 21 1	4.74	1	Xia2-DIALS	14/05/2020 08:52	-	High Salt	Q14K	S7N:L12C	Non-D69Y Crystal Epitopes	no
G3-089	XX21RECQ L5A-x0624	Frozen	35.6535	20	CI079738	HIN3-170302-01	B	12	a	2.8M sodium acetate	2	Tetramer P 2 2 21 Porous	158.09 277.74 95.13 (90.0 90.0 90.0)	P 21 21 2	7.23	1	Xia2-3dii	14/05/2020 09:00	-	High Salt	Q14K	S7N:L12C	Non-D69Y Crystal Epitopes	no
G3-089	XX21RECQ L5A-x0623	Frozen	35.6535	20	CI079738	HIN3-170302-01	A	12	a	3M sodium chloride -- 0.1M tris pH 8.5	NA		NA	NA	NA	0	NA	14/05/2020 08:56	-	High Salt	Q14K	S7N:L12C	Non-D69Y Crystal Epitopes	no
G3-089	XX21RECQ L5A-x0621	Frozen	35.6535	20	CI079738	HIN3-170302-01	A	5	a	2M ammonium sulfate -- 0.1M HEPES pH 7.5	NA		NA	NA	NA	0	NA	14/05/2020 08:48	-	High Salt	Q14K	S7N:L12C	Non-D69Y Crystal Epitopes	no

Table S4 Diffraction data

Nanobody grouped ID	Mounted Crystal ID	Xtal Plate Protein Frozen/Fresh?	Xtal Plate Protein Concentration (mg/ml)	Xtal Plate Temperature	Xtal Plate Barcode	Screen Batch Name	XTBM Row	XTBM Column	XTBM Subwell	XTBM Condition	Crystal Form Group	Cystal Form Annotation	Unit Cell Dimensions	Space group	Resolution	Number of Successful Autoprocessing Pipelines	Autoprocessing Pipeline Used	Experiment Time	Soaking condition	Xtal Group	Surface Mutations Around Nbinter-1	Key Mutations	Crystal Epitope Mutations	Condition Repitition?
G3-092	XX21RECQ L5A-x0625	Frozen	33.4845	20	CI079739	HIN3-170302-01	A	10	a	3M sodium chloride -- 0.1M bis-tris pH 6.5	2	Tetramer P 2 2 21 Porous	89.76 150.94 271.05 (90.0 90.0 90.0)	P 2 2 2	4.03	5	Xia2-DIALS	14/05/2020 09:04	-	High Salt	R20K	S7N:L12 C	Non-D69Y Crystal Epitopes	no
G3-092	XX21RECQ L5A-x0632	Frozen	33.4845	20	CI079739	HIN3-170302-01	C	11	a	1M ammonium sulfate -- 0.5% PEG8000 -- 0.1M HEPES pH 7.0	2	Tetramer P 2 2 21 Porous	90.03 151.88 270.36 (90.0 90.0 90.0)	P 2 2 2	4.12	3	Xia2-DIALS	14/05/2020 05:20	-	High Salt	R20K	S7N:L12 C	Non-D69Y Crystal Epitopes	no
G3-092	XX21RECQ L5A-x0635	Frozen	33.4845	20	CI079739	HIN3-170302-01	C	12	c	15% tacsimate -- 2% PEG3350 -- 0.1M HEPES pH 7.0	2	Tetramer P 2 2 21 Porous	142.76 264.44 88.34 (90.0 90.0 90.0)	P 21 21 2	4.66	1	Xia2-3dii	14/05/2020 05:31	-	High Salt	R20K	S7N:L12 C	Non-D69Y Crystal Epitopes	no
G3-092	XX21RECQ L5A-x0634	Frozen	33.4845	20	CI079739	HIN3-170302-01	C	12	a	15% tacsimate -- 2% PEG3350 -- 0.1M HEPES pH 7.0	NA		NA	NA	NA	0	NA	14/05/2020 05:27	-	High Salt	R20K	S7N:L12 C	Non-D69Y Crystal Epitopes	no
G3-092	XX21RECQ L5A-x0633	Frozen	33.4845	20	CI079739	HIN3-170302-01	C	11	c	1M ammonium sulfate -- 0.5% PEG8000 -- 0.1M HEPES pH 7.0	NA		NA	NA	NA	0	NA	14/05/2020 05:24	-	High Salt	R20K	S7N:L12 C	Non-D69Y Crystal Epitopes	no
G3-092	XX21RECQ L5A-x0630	Frozen	33.4845	20	CI079739	HIN3-170302-01	A	12	c	3M sodium chloride -- 0.1M tris pH 8.5	NA		NA	NA	NA	0	NA	14/05/2020 05:15	-	High Salt	R20K	S7N:L12 C	Non-D69Y Crystal Epitopes	no
G3-092	XX21RECQ L5A-x0629	Frozen	33.4845	20	CI079739	HIN3-170302-01	A	12	a	3M sodium chloride -- 0.1M tris pH 8.5	NA		NA	NA	NA	0	NA	14/05/2020 05:12	-	High Salt	R20K	S7N:L12 C	Non-D69Y Crystal Epitopes	no
G3-092	XX21RECQ L5A-x0628	Frozen	33.4845	20	CI079739	HIN3-170302-01	A	11	d	3M sodium chloride -- 0.1M HEPES pH 7.5	NA		NA	NA	NA	0	NA	14/05/2020 05:08	-	High Salt	R20K	S7N:L12 C	Non-D69Y Crystal Epitopes	no
G3-092	XX21RECQ L5A-x0627	Frozen	33.4845	20	CI079739	HIN3-170302-01	A	11	a	3M sodium chloride -- 0.1M HEPES pH 7.5	NA		NA	NA	NA	0	NA	14/05/2020 05:04	-	High Salt	R20K	S7N:L12 C	Non-D69Y Crystal Epitopes	no
G3-092	XX21RECQ L5A-x0626	Frozen	33.4845	20	CI079739	HIN3-170302-01	A	10	c	3M sodium chloride -- 0.1M bis-tris pH 6.5	NA		NA	NA	NA	0	NA	14/05/2020 05:00	-	High Salt	R20K	S7N:L12 C	Non-D69Y Crystal Epitopes	no
G3-093	XX21RECQ L5A-x0637	Frozen	34.569	20	CI079720	HIN3-170302-01	A	12	d	3M sodium chloride -- 0.1M tris pH 8.5	NA		NA	NA	NA	0	NA	14/05/2020 05:40	-	High Salt	Q90R	S7N:L12 C	Non-D69Y Crystal Epitopes	no
G3-093	XX21RECQ L5A-x0636	Frozen	34.569	20	CI079720	HIN3-170302-01	A	11	c	3M sodium chloride -- 0.1M HEPES pH 7.5	NA		NA	NA	NA	0	NA	14/05/2020 05:36	-	High Salt	Q90R	S7N:L12 C	Non-D69Y Crystal Epitopes	no
G3-095	XX21RECQ L5A-x0639	Frozen	33.3945	20	CI079427	HIN3-170302-01	A	10	a	3M sodium chloride -- 0.1M bis-tris pH 6.5	2	Tetramer P 2 2 21 Porous	90.11 152.45 270.5 (90.0 90.0 90.0)	P 2 21 21	4.26	1	Xia2-DIALS	14/05/2020 14:22	-	High Salt	Q90H	S7N:L12 C	Non-D69Y Crystal Epitopes	no
G3-095	XX21RECQ L5A-x0638	Frozen	33.3945	20	CI079427	HIN3-170302-01	A	9	a	3M sodium chloride -- 0.1M bis-tris pH 5.5	2	Tetramer P 2 2 21 Porous	89.84 153.28 272.65 (90.0 90.0 90.0)	P 2 2 21	4.5	5	Xia2-DIALS	14/05/2020 05:56	-	High Salt	Q90H	S7N:L12 C	Non-D69Y Crystal Epitopes	no
G3-095	XX21RECQ L5A-x0643	Frozen	33.3945	20	CI079427	HIN3-170302-01	A	12	a	3M sodium chloride -- 0.1M tris pH 8.5	2	Tetramer P 2 2 21 Porous	90.37 152.31 271.6 (90.0 90.0 90.0)	P 2 21 21	4.93	1	Xia2-DIALS	14/05/2020 23:47	-	High Salt	Q90H	S7N:L12 C	Non-D69Y Crystal Epitopes	no

Table S4 Diffraction data

Nanobody grouped ID	Mounted Crystal ID	Xtal Plate Protein Frozen/Fresh?	Xtal Plate Protein Concentration (mg/ml)	Xtal Plate Temperature	Xtal Plate Barcode	Screen Batch Name	XTBM Row	XTBM Column	XTBM Subwell	XTBM Condition	Crystal Form Group	Cystal Form Annotation	Unit Cell Dimensions	Space group	Resolution	Number of Successful Autoprocessing Pipelines	Autoprocessing Pipeline Used	Experiment Time	Soaking condition	Xtal Group	Surface Mutations Around Nbinter-1	Key Mutations	Crystal Epitope Mutations	Condition Repitition?
G3-095	XX21RECQ L5A-x0646	Frozen	33.3945	20	CI079427	HIN3-170302-01	C	2	c	1.1M ammonium tartrate	NA		NA	NA	NA	0	NA	14/05/2020 23:59	-	High Salt	Q90H	S7N:L12 C	Non-D69Y Crystal Epitopes	no
G3-095	XX21RECQ L5A-x0645	Frozen	33.3945	20	CI079427	HIN3-170302-01	A	12	d	3M sodium chloride -- 0.1M tris pH 8.5	NA		NA	NA	NA	0	NA	14/05/2020 23:55	-	High Salt	Q90H	S7N:L12 C	Non-D69Y Crystal Epitopes	no
G3-095	XX21RECQ L5A-x0644	Frozen	33.3945	20	CI079427	HIN3-170302-01	A	12	c	3M sodium chloride -- 0.1M tris pH 8.5	NA		NA	NA	NA	0	NA	14/05/2020 23:51	-	High Salt	Q90H	S7N:L12 C	Non-D69Y Crystal Epitopes	no
G3-095	XX21RECQ L5A-x0642	Frozen	33.3945	20	CI079427	HIN3-170302-01	A	11	a	3M sodium chloride -- 0.1M HEPES pH 7.5	NA		NA	NA	NA	0	NA	14/05/2020 23:44	-	High Salt	Q90H	S7N:L12 C	Non-D69Y Crystal Epitopes	no
G3-095	XX21RECQ L5A-x0641	Frozen	33.3945	20	CI079427	HIN3-170302-01	A	10	d	3M sodium chloride -- 0.1M bis-tris pH 6.5	NA		NA	NA	NA	0	NA	14/05/2020 23:39	-	High Salt	Q90H	S7N:L12 C	Non-D69Y Crystal Epitopes	no
G3-095	XX21RECQ L5A-x0640	Frozen	33.3945	20	CI079427	HIN3-170302-01	A	10	c	3M sodium chloride -- 0.1M bis-tris pH 6.5	NA		NA	NA	NA	0	NA	14/05/2020 23:36	-	High Salt	Q90H	S7N:L12 C	Non-D69Y Crystal Epitopes	no
G3-096	XX21RECQ L5A-x0649	Frozen	28.287	20	CI079758	HIN3-170302-01	F	3	c	5% tacsimate -- 10% PEG5000MME -- 0.1M HEPES pH 7.0	NA		NA	NA	NA	0	NA	14/05/2020 05:52	-	PEG	N92D	S7N:L12 C	Non-D69Y Crystal Epitopes	no
G3-096	XX21RECQ L5A-x0648	Frozen	28.287	20	CI079758	HIN3-170302-01	E	9	a	0.05M ammonium sulfate -- 30% pentaerythritol ethoxylate 15/4 -- 0.1M bis-tris pH 6.5	NA		NA	NA	NA	0	NA	14/05/2020 05:48	-	High Salt	N92D	S7N:L12 C	Non-D69Y Crystal Epitopes	no
G3-096	XX21RECQ L5A-x0647	Frozen	28.287	20	CI079758	HIN3-170302-01	A	10	d	3M sodium chloride -- 0.1M bis-tris pH 6.5	NA		NA	NA	NA	0	NA	14/05/2020 05:44	-	High Salt	N92D	S7N:L12 C	Non-D69Y Crystal Epitopes	no
G4-025	XX21RECQ L5A-x0727	Fresh	22.02	20	CI080557	HIN3-170302-01	G	1	a	0.2M sodium chloride -- 25% PEG3350 -- 0.1M tris pH 8.5	4	Tetramer I 2 2 2/ C 1 2 1	227.13 90.97 81.08 (90.0 108.47 90.0)	C 1 2 1	2.61	5	Xia2-DIALS	02/12/2020 19:05	-	PEG	R20K	S7N:L12 C:T125 M	Non-D69Y Crystal Epitopes	no
G4-025	XX21RECQ L5A-x0728	Fresh	22.02	20	CI080557	HIN3-170302-01	G	1	d	0.2M sodium chloride -- 25% PEG3350 -- 0.1M tris pH 8.5	4	Tetramer I 2 2 2/ C 1 2 1	82.23 90.29 216.18 (90.0 90.0 90.0)	I 2 2 2	2.63	5	Xia2-DIALS	02/12/2020 19:08	-	PEG	R20K	S7N:L12 C:T125 M	Non-D69Y Crystal Epitopes	no
G4-025	XX21RECQ L5A-x0725	Fresh	22.02	20	CI080557	HIN3-170302-01	A	12	a	3M sodium chloride -- 0.1M tris pH 8.5	2	Tetramer P 2 2 21 Porous	90.29 152.18 268.99 (90.0 90.0 90.0)	P 2 21 21	5.02	5	Xia2-DIALS	02/12/2020 19:00	-	High Salt	R20K	S7N:L12 C:T125 M	Non-D69Y Crystal Epitopes	no
G4-025	XX21RECQ L5A-x0726	Fresh	22.02	20	CI080557	HIN3-170302-01	B	11	a	2.1M DL- malic acid	NA		NA	NA	NA	0	NA	02/12/2020 19:03	-	High Salt	R20K	S7N:L12 C:T125 M	Non-D69Y Crystal Epitopes	no
G4-025	XX21RECQ L5A-x0724	Fresh	22.02	20	CI080557	HIN3-170302-01	A	5	d	2M ammonium sulfate -- 0.1M HEPES pH 7.5	NA		NA	NA	NA	0	NA	02/12/2020 18:57	-	High Salt	R20K	S7N:L12 C:T125 M	Non-D69Y Crystal Epitopes	no
G4-027	XX21RECQ L5A-x0752	Fresh	19.88	20	CI080559	HIN3-170302-01	G	1	a	0.2M sodium chloride -- 25% PEG3350 -- 0.1M tris pH 8.5	4	Tetramer I 2 2 2/ C 1 2 1	81.33 90.1 214.45 (90.0 90.0 90.0)	I 2 2 2	2.68	5	Xia2-DIALS	03/12/2020 23:41	-	PEG	Q90Y	S7N:L12 C:T125 M	Non-D69Y Crystal Epitopes	no

Table S4 Diffraction data

Nanobody grouped ID	Mounted Crystal ID	Xtal Plate Protein Frozen/Fresh?	Xtal Plate Protein Concentration (mg/ml)	Xtal Plate Temperature	Xtal Plate Barcode	Screen Batch Name	XTBM Row	XTBM Column	XTBM Subwell	XTBM Condition	Crystal Form Group	Cystal Form Annotation	Unit Cell Dimensions	Space group	Resolution	Number of Successful Autoprocessing Pipelines	Autoprocessing Pipeline Used	Experiment Time	Soaking condition	Xtal Group	Surface Mutations Around Nbinter-1	Key Mutations	Crystal Epitope Mutations	Condition Repitition?
G4-027	XX21RECQ L5A-x0751	Fresh	19.88	20	CI080559	HIN3-170302-01	F	7	c	0.2M ammonium sulfate -- 25% PEG3350 -- 0.1M bis-tris pH 6.5	NA		NA	NA	NA	0	NA	03/12/2020 23:37	-	PEG	Q90Y	S7N:L12 C:T125 M	Non-D69Y Crystal Epitopes	no
G4-027	XX21RECQ L5A-x0750	Fresh	19.88	20	CI080559	HIN3-170302-01	A	12	a	3M sodium chloride -- 0.1M tris pH 8.5	NA		NA	NA	NA	0	NA	03/12/2020 23:34	-	High Salt	Q90Y	S7N:L12 C:T125 M	Non-D69Y Crystal Epitopes	no
G4-027	XX21RECQ L5A-x0749	Fresh	19.88	20	CI080559	HIN3-170302-01	A	5	c	2M ammonium sulfate -- 0.1M HEPES pH 7.5	NA		NA	NA	NA	0	NA	03/12/2020 23:31	-	High Salt	Q90Y	S7N:L12 C:T125 M	Non-D69Y Crystal Epitopes	no
G4-029	XX21RECQ L5A-x0786	Fresh	20.12	20	CI080536	HIN3-170302-01	F	10	c	0.2M sodium chloride -- 25% PEG3350 -- 0.1M bis-tris pH 5.5	4	Tetramer I 2 2 2/ C 1 2 1	82.23 90.92 218.37 (90.0 90.0 90.0)	I 2 2 2	2.66	4	Xia2-DIALS	03/12/2020 22:54	-	PEG	N92D	S7N:L12 C:T125 M	Non-D69Y Crystal Epitopes	no
G4-029	XX21RECQ L5A-x0789	Fresh	20.12	20	CI080536	HIN3-170302-01	G	1	d	0.2M sodium chloride -- 25% PEG3350 -- 0.1M tris pH 8.5	4	Tetramer I 2 2 2/ C 1 2 1	80.88 90.81 216.41 (90.0 90.0 90.0)	I 2 2 2	3.12	2	Xia2-DIALS	02/12/2020 18:47	-	PEG	N92D	S7N:L12 C:T125 M	Non-D69Y Crystal Epitopes	no
G4-029	XX21RECQ L5A-x0790	Fresh	20.12	20	CI080536	HIN3-170302-01	G	12	c	0.2M magnesium chloride -- 25% PEG3350 -- 0.1M HEPES pH 7.5	4	Tetramer I 2 2 2/ C 1 2 1	219.72 89.98 79.75 (90.0 108.16 90.0)	C 1 2 1	3.44	2	Xia2-DIALS	02/12/2020 18:51	-	PEG	N92D	S7N:L12 C:T125 M	Non-D69Y Crystal Epitopes	no
G4-029	XX21RECQ L5A-x0791	Fresh	20.12	20	CI080536	HIN3-170302-01	H	11	c	0.1M potassium thiocyanate -- 30% PEG2000MME	NA		NA	NA	NA	0	NA	02/12/2020 18:54	-	PEG	N92D	S7N:L12 C:T125 M	Non-D69Y Crystal Epitopes	no
G4-029	XX21RECQ L5A-x0788	Fresh	20.12	20	CI080536	HIN3-170302-01	G	1	c	0.2M sodium chloride -- 25% PEG3350 -- 0.1M tris pH 8.5	NA		NA	NA	NA	0	NA	02/12/2020 18:44	-	PEG	N92D	S7N:L12 C:T125 M	Non-D69Y Crystal Epitopes	no
G4-029	XX21RECQ L5A-x0787	Fresh	20.12	20	CI080536	HIN3-170302-01	F	11	c	0.2M sodium chloride -- 25% PEG3350 -- 0.1M bis-tris pH 6.5	NA		NA	NA	NA	0	NA	03/12/2020 22:57	-	PEG	N92D	S7N:L12 C:T125 M	Non-D69Y Crystal Epitopes	no
G4-029	XX21RECQ L5A-x0785	Fresh	20.12	20	CI080536	HIN3-170302-01	F	2	c	0.2M trimethylamine N-oxide -- 20% PEG2000MME -- 0.1M tris pH 8.5	NA		NA	NA	NA	0	NA	03/12/2020 22:51	-	PEG	N92D	S7N:L12 C:T125 M	Non-D69Y Crystal Epitopes	no
G4-029	XX21RECQ L5A-x0784	Fresh	20.12	20	CI080536	HIN3-170302-01	D	3	a	30% jeffamine ED-2003 -- 0.1M HEPES pH 7.0	NA		NA	NA	NA	0	NA	03/12/2020 22:47	-	Jeffa mine	N92D	S7N:L12 C:T125 M	Non-D69Y Crystal Epitopes	no
G4-029	XX21RECQ L5A-x0783	Fresh	20.12	20	CI080536	HIN3-170302-01	A	10	a	3M sodium chloride -- 0.1M bis-tris pH 6.5	NA		NA	NA	NA	0	NA	03/12/2020 22:44	-	High Salt	N92D	S7N:L12 C:T125 M	Non-D69Y Crystal Epitopes	no
G5-001	XX21RECQ L5A-x0748	Fresh	22.89	20	CI080551	HIN3-170302-01	H	6	d	0.2M sodium formate -- 20% PEG3350	4	Tetramer I 2 2 2/ C 1 2 1	82.0 89.56 219.08 (90.0 90.0 90.0)	I 2 2 2	2.11	5	Xia2-DIALS	02/12/2020 19:26	-	PEG	Q14K	S7N:L12 C:T125 M	Non-D69Y Crystal Epitopes	no
G5-001	XX21RECQ L5A-x0746	Fresh	22.89	20	CI080551	HIN3-170302-01	G	1	d	0.2M sodium chloride -- 25% PEG3350 -- 0.1M tris pH 8.5	4	Tetramer I 2 2 2/ C 1 2 1	232.56 89.54 81.63 (90.0 109.55 90.0)	C 1 2 1	2.4	5	Xia2-DIALS	02/12/2020 19:21	-	PEG	Q14K	S7N:L12 C:T125 M	Non-D69Y Crystal Epitopes	no
G5-001	XX21RECQ L5A-x0745	Fresh	22.89	20	CI080551	HIN3-170302-01	G	1	a	0.2M sodium chloride -- 25% PEG3350 -- 0.1M tris pH 8.5	4	Tetramer I 2 2 2/ C 1 2 1	81.06 89.05 215.12 (90.0 90.0 90.0)	I 2 2 2	3.5	3	Xia2-3dii	02/12/2020 19:18	-	PEG	Q14K	S7N:L12 C:T125 M	Non-D69Y Crystal Epitopes	no

Table S4 Diffraction data

Nanobody grouped ID	Mounted Crystal ID	Xtal Plate Protein Frozen/Fresh?	Xtal Plate Protein Concentration (mg/ml)	Xtal Plate Temperature	Xtal Plate Barcode	Screen Batch Name	XTBM Row	XTBM Column	XTBM Subwell	XTBM Condition	Crystal Form Group	Cystal Form Annotation	Unit Cell Dimensions	Space group	Resolution	Number of Successful Autoprocessing Pipelines	Autoprocessing Pipeline Used	Experiment Time	Soaking condition	Xtal Group	Surface Mutations Around Nbinter-1	Key Mutations	Crystal Epitope Mutations	Condition Repitition?
G5-001	XX21RECQ L5A-x0743	Fresh	22.89	20	CI080551	HIN3-170302-01	A	5	c	2M ammonium sulfate -- 0.1M HEPES pH 7.5	2	Tetramer P 2 2 21 Porous	88.85 151.71 277.42 (90.0 90.0 90.0)	P 2 2 21	6.51	4	Xia2-3dii	02/12/2020 19:11	-	High Salt	Q14K	S7N:L12 C:T125 M	Non-D69Y Crystal Epitopes	no
G5-001	XX21RECQ L5A-x0747	Fresh	22.89	20	CI080551	HIN3-170302-01	G	3	d	0.2M lithium sulfate -- 25% PEG3350 -- 0.1M bis-tris pH 6.5	NA		NA	NA	NA	0	NA	02/12/2020 19:24	-	PEG	Q14K	S7N:L12 C:T125 M	Non-D69Y Crystal Epitopes	no
G5-001	XX21RECQ L5A-x0744	Fresh	22.89	20	CI080551	HIN3-170302-01	F	9	c	0.2M ammonium sulfate -- 25% PEG3350 -- 0.1M tris pH 8.5	NA		NA	NA	NA	0	NA	02/12/2020 19:15	-	PEG	Q14K	S7N:L12 C:T125 M	Non-D69Y Crystal Epitopes	no
G5-003	XX21RECQ L5A-x0736	Fresh	18.19	20	CI080556	HIN3-170302-01	G	1	a	0.2M sodium chloride -- 25% PEG3350 -- 0.1M tris pH 8.5	4	Tetramer I 2 2 2/ C 1 2 1	82.6 90.82 221.88 (90.0 90.0 90.0)	I 2 2 2	2.11	5	Xia2-DIALS	03/12/2020 23:06	-	PEG	Q14K	S7N:L12 C:T125 M	Non-D69Y Crystal Epitopes	no
G5-003	XX21RECQ L5A-x0740	Fresh	18.19	20	CI080556	HIN3-170302-01	G	5	a	0.2M lithium sulfate -- 25% PEG3350 -- 0.1M tris pH 8.5	4	Tetramer I 2 2 2/ C 1 2 1	230.64 90.14 80.85 (90.0 109.01 90.0)	C 1 2 1	2.49	5	Xia2-DIALS	03/12/2020 23:22	-	PEG	Q14K	S7N:L12 C:T125 M	Non-D69Y Crystal Epitopes	no
G5-003	XX21RECQ L5A-x0737	Fresh	18.19	20	CI080556	HIN3-170302-01	G	1	c	0.2M sodium chloride -- 25% PEG3350 -- 0.1M tris pH 8.5	4	Tetramer I 2 2 2/ C 1 2 1	82.52 90.65 218.06 (90.0 90.0 90.0)	I 2 2 2	2.57	5	Xia2-3dii	03/12/2020 23:09	-	PEG	Q14K	S7N:L12 C:T125 M	Non-D69Y Crystal Epitopes	no
G5-003	XX21RECQ L5A-x0741	Fresh	18.19	20	CI080556	HIN3-170302-01	G	5	c	0.2M lithium sulfate -- 25% PEG3350 -- 0.1M tris pH 8.5	4	Tetramer I 2 2 2/ C 1 2 1	219.31 90.28 80.02 (90.0 108.21 90.0)	C 1 2 1	2.87	5	Xia2-DIALS	03/12/2020 23:25	-	PEG	Q14K	S7N:L12 C:T125 M	Non-D69Y Crystal Epitopes	no
G5-003	XX21RECQ L5A-x0735	Fresh	18.19	20	CI080556	HIN3-170302-01	F	9	d	0.2M ammonium sulfate -- 25% PEG3350 -- 0.1M tris pH 8.5	4	Tetramer I 2 2 2/ C 1 2 1	79.94 88.67 215.86 (90.0 90.0 90.0)	I 2 2 2	3.18	2	Xia2-DIALS	03/12/2020 23:03	-	PEG	Q14K	S7N:L12 C:T125 M	Non-D69Y Crystal Epitopes	no
G5-003	XX21RECQ L5A-x0734	Fresh	18.19	20	CI080556	HIN3-170302-01	F	8	d	0.2M ammonium sulfate -- 25% PEG3350 -- 0.1M HEPES pH 7.5	4	Tetramer I 2 2 2/ C 1 2 1	218.18 90.21 80.0 (90.0 108.43 90.0)	C 1 2 1	3.35	1	Xia2-DIALS	03/12/2020 23:00	-	PEG	Q14K	S7N:L12 C:T125 M	Non-D69Y Crystal Epitopes	no
G5-003	XX21RECQ L5A-x0729	Fresh	18.19	20	CI080556	HIN3-170302-01	B	7	a	0.056M sodium phosphate monobasic -- 1.344M potassium phosphate dibasic	2	Tetramer P 2 2 21 Porous	149.84 270.76 89.15 (90.0 90.0 90.0)	P 21 21 2	6.24	2	Xia2-3dii	02/12/2020 19:29	-	High Salt	Q14K	S7N:L12 C:T125 M	Non-D69Y Crystal Epitopes	no
G5-003	XX21RECQ L5A-x0733	Fresh	18.19	20	CI080556	HIN3-170302-01	C	1	a	3.5M sodium formate	2	Tetramer P 2 2 21 Porous	89.98 150.49 267.22 (90.0 90.0 90.0)	P 2 2 2	7.02	3	Xia2-3dii	02/12/2020 19:52	-	High Salt	Q14K	S7N:L12 C:T125 M	Non-D69Y Crystal Epitopes	no
G5-003	XX21RECQ L5A-x0742	Fresh	18.19	20	CI080556	HIN3-170302-01	H	6	d	0.2M sodium formate -- 20% PEG3350	NA		NA	NA	NA	0	NA	03/12/2020 23:28	-	PEG	Q14K	S7N:L12 C:T125 M	Non-D69Y Crystal Epitopes	no
G5-003	XX21RECQ L5A-x0739	Fresh	18.19	20	CI080556	HIN3-170302-01	G	4	d	0.2M lithium sulfate -- 25% PEG3350 -- 0.1M HEPES pH 7.5	NA		NA	NA	NA	0	NA	03/12/2020 23:15	-	PEG	Q14K	S7N:L12 C:T125 M	Non-D69Y Crystal Epitopes	no
G5-003	XX21RECQ L5A-x0738	Fresh	18.19	20	CI080556	HIN3-170302-01	G	1	d	0.2M sodium chloride -- 25% PEG3350 -- 0.1M tris pH 8.5	NA		NA	NA	NA	0	NA	03/12/2020 23:12	-	PEG	Q14K	S7N:L12 C:T125 M	Non-D69Y Crystal Epitopes	no
G5-003	XX21RECQ L5A-x0732	Fresh	18.19	20	CI080556	HIN3-170302-01	B	12	c	2.8M sodium acetate	NA		NA	NA	NA	0	NA	02/12/2020 19:45	-	High Salt	Q14K	S7N:L12 C:T125 M	Non-D69Y Crystal Epitopes	no

Table S4 Diffraction data

Nanobody grouped ID	Mounted Crystal ID	Xtal Plate Protein Frozen/Fresh?	Xtal Plate Protein Concentration (mg/ml)	Xtal Plate Temperature	Xtal Plate Barcode	Screen Batch Name	XTBM Row	XTBM Column	XTBM Subwell	XTBM Condition	Crystal Form Group	Cystal Form Annotation	Unit Cell Dimensions	Space group	Resolution	Number of Successful Autoprocessing Pipelines	Autoprocessing Pipeline Used	Experiment Time	Soaking condition	Xtal Group	Surface Mutations Around Nbinter-1	Key Mutations	Crystal Epitope Mutations	Condition Repitition?
G5-003	XX21RECQ L5A-x0731	Fresh	18.19	20	CI080556	HIN3-170302-01	B	12	a	2.8M sodium acetate	NA		NA	NA	NA	0	NA	02/12/2020 19:36	-	High Salt	Q14K	S7N:L12 C:T125 M	Non-D69Y Crystal Epitopes	no
G5-003	XX21RECQ L5A-x0730	Fresh	18.19	20	CI080556	HIN3-170302-01	B	7	c	0.056M sodium phosphate monobasic -- 1.344M potassium phosphate dibasic	NA		NA	NA	NA	0	NA	02/12/2020 19:33	-	High Salt	Q14K	S7N:L12 C:T125 M	Non-D69Y Crystal Epitopes	no
G5-006	XX21RECQ L5A-x1378	Fresh	40	20	CI072128	HIN3-170302-01	F	8	c	0.2M ammonium sulfate -- 25% PEG3350 -- 0.1M HEPES pH 7.5	4	Tetramer I 2 2 2/ C 1 2 1	232.44 89.9 164.05 (90.0 110.09 90.0)	C 1 2 1	2	5	Xia2-DIALS	12/01/2021 14:16	30% DMSO - 0% EG	PEG	Q14K	S7N:L12 C:T125 M	Non-D69Y Crystal Epitopes	no
G5-006	XX21RECQ L5A-x1391	Fresh	40	20	CI072128	HIN3-170302-01	F	8	c	0.2M ammonium sulfate -- 25% PEG3350 -- 0.1M HEPES pH 7.5	4	Tetramer I 2 2 2/ C 1 2 1	232.71 89.84 164.14 (90.0 110.17 90.0)	C 1 2 1	2.03	5	Xia2-DIALS	12/01/2021 14:51	15% DMSO - 15% EG	PEG	Q14K	S7N:L12 C:T125 M	Non-D69Y Crystal Epitopes	no
G5-006	XX21RECQ L5A-x1355	Fresh	40	20	CI072128	HIN3-170302-01	F	8	c	0.2M ammonium sulfate -- 25% PEG3350 -- 0.1M HEPES pH 7.5	4	Tetramer I 2 2 2/ C 1 2 1	231.58 89.81 163.25 (90.0 109.99 90.0)	C 1 2 1	2.04	5	Xia2-DIALS	12/01/2021 13:11	0% DMSO - 15% EG	PEG	Q14K	S7N:L12 C:T125 M	Non-D69Y Crystal Epitopes	no
G5-006	XX21RECQ L5A-x1366	Fresh	40	20	CI072128	HIN3-170302-01	F	8	c	0.2M ammonium sulfate -- 25% PEG3350 -- 0.1M HEPES pH 7.5	4	Tetramer I 2 2 2/ C 1 2 1	232.8 90.01 164.34 (90.0 109.97 90.0)	C 1 2 1	2.05	5	Xia2-DIALS	12/01/2021 13:42	10% DMSO - 15% EG	PEG	Q14K	S7N:L12 C:T125 M	Non-D69Y Crystal Epitopes	no
G5-006	XX21RECQ L5A-x1371	Fresh	40	20	CI072128	HIN3-170302-01	F	8	c	0.2M ammonium sulfate -- 25% PEG3350 -- 0.1M HEPES pH 7.5	4	Tetramer I 2 2 2/ C 1 2 1	233.01 90.33 164.46 (90.0 110.08 90.0)	C 1 2 1	2.11	5	Xia2-DIALS	12/01/2021 13:56	20% DMSO - 0% EG	PEG	Q14K	S7N:L12 C:T125 M	Non-D69Y Crystal Epitopes	no
G5-006	XX21RECQ L5A-x0766	Fresh	20.04	20	CI080566	HIN3-170302-01	H	6	a	0.2M sodium formate -- 20% PEG3350	4	Tetramer I 2 2 2/ C 1 2 1	230.89 90.43 82.02 (90.0 110.07 90.0)	C 1 2 1	2.13	5	Xia2-DIALS	04/12/2020 07:28	-	PEG	Q14K	S7N:L12 C:T125 M	Non-D69Y Crystal Epitopes	no
G5-006	XX21RECQ L5A-x1390	Fresh	40	20	CI072128	HIN3-170302-01	F	8	c	0.2M ammonium sulfate -- 25% PEG3350 -- 0.1M HEPES pH 7.5	4	Tetramer I 2 2 2/ C 1 2 1	232.08 89.5 163.48 (90.0 109.95 90.0)	C 1 2 1	2.13	5	Xia2-DIALS	12/01/2021 14:48	15% DMSO - 15% EG	PEG	Q14K	S7N:L12 C:T125 M	Non-D69Y Crystal Epitopes	no
G5-006	XX21RECQ L5A-x1357	Fresh	40	20	CI072128	HIN3-170302-01	F	8	c	0.2M ammonium sulfate -- 25% PEG3350 -- 0.1M HEPES pH 7.5	4	Tetramer I 2 2 2/ C 1 2 1	231.61 89.83 163.6 (90.0 109.81 90.0)	C 1 2 1	2.16	5	Xia2-DIALS	12/01/2021 13:16	0% DMSO - 15% EG	PEG	Q14K	S7N:L12 C:T125 M	Non-D69Y Crystal Epitopes	no
G5-006	XX21RECQ L5A-x1382	Fresh	40	20	CI072128	HIN3-170302-01	F	8	c	0.2M ammonium sulfate -- 25% PEG3350 -- 0.1M HEPES pH 7.5	4	Tetramer I 2 2 2/ C 1 2 1	232.54 89.97 165.04 (90.0 110.17 90.0)	C 1 2 1	2.19	4	Xia2-DIALS	12/01/2021 14:26	30% DMSO - 0% EG	PEG	Q14K	S7N:L12 C:T125 M	Non-D69Y Crystal Epitopes	no
G5-006	XX21RECQ L5A-x1376	Fresh	40	20	CI072128	HIN3-170302-01	F	8	c	0.2M ammonium sulfate -- 25% PEG3350 -- 0.1M HEPES pH 7.5	4	Tetramer I 2 2 2/ C 1 2 1	232.72 89.66 163.77 (90.0 109.97 90.0)	C 1 2 1	2.23	5	Xia2-DIALS	12/01/2021 14:10	20% DMSO - 15% EG	PEG	Q14K	S7N:L12 C:T125 M	Non-D69Y Crystal Epitopes	no
G5-006	XX21RECQ L5A-x1384	Fresh	40	20	CI072128	HIN3-170302-01	F	8	c	0.2M ammonium sulfate -- 25% PEG3350 -- 0.1M HEPES pH 7.5	4	Tetramer I 2 2 2/ C 1 2 1	233.55 89.63 164.12 (90.0 110.05 90.0)	C 1 2 1	2.24	5	Xia2-DIALS	12/01/2021 14:32	30% DMSO - 15% EG	PEG	Q14K	S7N:L12 C:T125 M	Non-D69Y Crystal Epitopes	no
G5-006	XX21RECQ L5A-x1370	Fresh	40	20	CI072128	HIN3-170302-01	F	8	c	0.2M ammonium sulfate -- 25% PEG3350 -- 0.1M HEPES pH 7.5	4	Tetramer I 2 2 2/ C 1 2 1	230.8 89.75 163.47 (90.0 110.15 90.0)	C 1 2 1	2.26	5	Xia2-DIALS	12/01/2021 13:54	20% DMSO - 0% EG	PEG	Q14K	S7N:L12 C:T125 M	Non-D69Y Crystal Epitopes	no
G5-006	XX21RECQ L5A-x1362	Fresh	40	20	CI072128	HIN3-170302-01	F	8	c	0.2M ammonium sulfate -- 25% PEG3350 -- 0.1M HEPES pH 7.5	4	Tetramer I 2 2 2/ C 1 2 1	232.99 89.82 164.15 (90.0 110.0 90.0)	C 1 2 1	2.27	5	Xia2-DIALS	12/01/2021 13:29	10% DMSO - 0% EG	PEG	Q14K	S7N:L12 C:T125 M	Non-D69Y Crystal Epitopes	no

Table S4 Diffraction data

Nanobody grouped ID	Mounted Crystal ID	Xtal Plate Protein Frozen/Fresh?	Xtal Plate Protein Concentration (mg/ml)	Xtal Plate Temperature	Xtal Plate Barcode	Screen Batch Name	XTBM Row	XTBM Column	XTBM Subwell	XTBM Condition	Crystal Form Group	Cystal Form Annotation	Unit Cell Dimensions	Space group	Resolution	Number of Successful Autoprocessing Pipelines	Autoprocessing Pipeline Used	Experiment Time	Soaking condition	Xtal Group	Surface Mutations Around Nbinter-1	Key Mutations	Crystal Epitope Mutations	Condition Repitition?
G5-006	XX21RECQ L5A-x1372	Fresh	40	20	CI072128	HIN3-170302-01	F	8	c	0.2M ammonium sulfate -- 25% PEG3350 -- 0.1M HEPES pH 7.5	4	Tetramer I 2 2 2/ C 1 2 1	232.68 89.63 164.1 (90.0 109.91 90.0)	C 1 2 1	2.27	7	Xia2-DIALS	12/01/2021 13:59	20% DMSO - 0% EG	PEG	Q14K	S7N:L12 C:T125 M	Non-D69Y Crystal Epitopes	no
G5-006	XX21RECQ L5A-x1369	Fresh	40	20	CI072128	HIN3-170302-01	F	8	c	0.2M ammonium sulfate -- 25% PEG3350 -- 0.1M HEPES pH 7.5	4	Tetramer I 2 2 2/ C 1 2 1	233.1 89.55 164.03 (90.0 110.04 90.0)	C 1 2 1	2.27	5	Xia2-DIALS	12/01/2021 13:51	20% DMSO - 0% EG	PEG	Q14K	S7N:L12 C:T125 M	Non-D69Y Crystal Epitopes	no
G5-006	XX21RECQ L5A-x1379	Fresh	40	20	CI072128	HIN3-170302-01	F	8	c	0.2M ammonium sulfate -- 25% PEG3350 -- 0.1M HEPES pH 7.5	4	Tetramer I 2 2 2/ C 1 2 1	232.84 89.73 164.08 (90.0 110.04 90.0)	C 1 2 1	2.27	5	Xia2-DIALS	12/01/2021 14:18	30% DMSO - 0% EG	PEG	Q14K	S7N:L12 C:T125 M	Non-D69Y Crystal Epitopes	no
G5-006	XX21RECQ L5A-x1367	Fresh	40	20	CI072128	HIN3-170302-01	F	8	c	0.2M ammonium sulfate -- 25% PEG3350 -- 0.1M HEPES pH 7.5	4	Tetramer I 2 2 2/ C 1 2 1	234.3 89.55 164.02 (90.0 109.99 90.0)	C 1 2 1	2.31	5	Xia2-DIALS	12/01/2021 13:45	10% DMSO - 15% EG	PEG	Q14K	S7N:L12 C:T125 M	Non-D69Y Crystal Epitopes	no
G5-006	XX21RECQ L5A-x0850	Frozen	22	20	CI080726	HIN3-170302-01	F	8	c	0.2M ammonium sulfate -- 25% PEG3350 -- 0.1M HEPES pH 7.5	4	Tetramer I 2 2 2/ C 1 2 1	216.13 89.94 79.84 (90.0 107.66 90.0)	C 1 2 1	2.32	5	Xia2-DIALS	17/12/2020 06:25	-	PEG	Q14K	S7N:L12 C:T125 M	Non-D69Y Crystal Epitopes	yes
G5-006	XX21RECQ L5A-x0765	Fresh	20.04	20	CI080566	HIN3-170302-01	H	3	d	0.2M sodium malonate -- 20% PEG3350	4	Tetramer I 2 2 2/ C 1 2 1	229.38 90.42 81.13 (90.0 109.71 90.0)	C 1 2 1	2.33	5	Xia2-DIALS	04/12/2020 07:25	-	PEG	Q14K	S7N:L12 C:T125 M	Non-D69Y Crystal Epitopes	no
G5-006	XX21RECQ L5A-x1374	Fresh	40	20	CI072128	HIN3-170302-01	F	8	c	0.2M ammonium sulfate -- 25% PEG3350 -- 0.1M HEPES pH 7.5	4	Tetramer I 2 2 2/ C 1 2 1	234.52 89.38 164.3 (90.0 110.01 90.0)	C 1 2 1	2.33	5	Xia2-DIALS	12/01/2021 14:05	20% DMSO - 15% EG	PEG	Q14K	S7N:L12 C:T125 M	Non-D69Y Crystal Epitopes	no
G5-006	XX21RECQ L5A-x1373	Fresh	40	20	CI072128	HIN3-170302-01	F	8	c	0.2M ammonium sulfate -- 25% PEG3350 -- 0.1M HEPES pH 7.5	4	Tetramer I 2 2 2/ C 1 2 1	233.71 89.88 164.62 (90.0 109.96 90.0)	C 1 2 1	2.34	5	Xia2-DIALS	12/01/2021 14:02	20% DMSO - 15% EG	PEG	Q14K	S7N:L12 C:T125 M	Non-D69Y Crystal Epitopes	no
G5-006	XX21RECQ L5A-x1352	Fresh	10.63	20	CI072128	HIN3-170302-01	F	8	c	0.2M ammonium sulfate -- 25% PEG3350 -- 0.1M HEPES pH 7.5	4	Tetramer I 2 2 2/ C 1 2 1	82.1 89.75 220.11 (90.0 90.0 90.0)	I 2 2 2	2.36	7	Autoproc	12/01/2021 13:02	0% DMSO - 0% EG	PEG	Q14K	S7N:L12 C:T125 M	Non-D69Y Crystal Epitopes	no
G5-006	XX21RECQ L5A-x0842	Frozen	22	20	CI080726	HIN3-170302-01	F	8	c	0.2M ammonium sulfate -- 25% PEG3350 -- 0.1M HEPES pH 7.5	4	Tetramer I 2 2 2/ C 1 2 1	216.65 90.03 79.84 (90.0 107.33 90.0)	C 1 2 1	2.38	5	Xia2-DIALS	17/12/2020 06:03	-	PEG	Q14K	S7N:L12 C:T125 M	Non-D69Y Crystal Epitopes	yes
G5-006	XX21RECQ L5A-x1356	Fresh	40	20	CI072128	HIN3-170302-01	F	8	c	0.2M ammonium sulfate -- 25% PEG3350 -- 0.1M HEPES pH 7.5	4	Tetramer I 2 2 2/ C 1 2 1	231.28 89.76 163.62 (90.0 109.84 90.0)	C 1 2 1	2.38	7	Xia2-DIALS	12/01/2021 13:13	0% DMSO - 15% EG	PEG	Q14K	S7N:L12 C:T125 M	Non-D69Y Crystal Epitopes	no
G5-006	XX21RECQ L5A-x1381	Fresh	40	20	CI072128	HIN3-170302-01	F	8	c	0.2M ammonium sulfate -- 25% PEG3350 -- 0.1M HEPES pH 7.5	4	Tetramer I 2 2 2/ C 1 2 1	232.03 89.35 163.38 (90.0 110.06 90.0)	C 1 2 1	2.38	5	Xia2-DIALS	12/01/2021 14:23	30% DMSO - 0% EG	PEG	Q14K	S7N:L12 C:T125 M	Non-D69Y Crystal Epitopes	no
G5-006	XX21RECQ L5A-x1354	Fresh	40	20	CI072128	HIN3-170302-01	F	8	c	0.2M ammonium sulfate -- 25% PEG3350 -- 0.1M HEPES pH 7.5	4	Tetramer I 2 2 2/ C 1 2 1	232.49 89.83 163.9 (90.0 110.15 90.0)	C 1 2 1	2.39	5	Xia2-DIALS	12/01/2021 13:08	0% DMSO - 15% EG	PEG	Q14K	S7N:L12 C:T125 M	Non-D69Y Crystal Epitopes	no
G5-006	XX21RECQ L5A-x1364	Fresh	10.63	20	CI072128	HIN3-170302-01	F	8	c	0.2M ammonium sulfate -- 25% PEG3350 -- 0.1M HEPES pH 7.5	-1	NA	90.13 124.1 162.98 (108.57 90.02 111.0)	P 1	2.39	7	Xia2-DIALS	12/01/2021 13:37	10% DMSO - 15% EG	PEG	Q14K	S7N:L12 C:T125 M	Non-D69Y Crystal Epitopes	no
G5-006	XX21RECQ L5A-x1385	Fresh	40	20	CI072128	HIN3-170302-01	F	8	c	0.2M ammonium sulfate -- 25% PEG3350 -- 0.1M HEPES pH 7.5	4	Tetramer I 2 2 2/ C 1 2 1	236.08 89.88 165.04 (90.0 109.98 90.0)	C 1 2 1	2.39	5	Xia2-DIALS	12/01/2021 14:35	30% DMSO - 15% EG	PEG	Q14K	S7N:L12 C:T125 M	Non-D69Y Crystal Epitopes	no

Table S4 Diffraction data

Nanobody grouped ID	Mounted Crystal ID	Xtal Plate Protein Frozen/Fresh?	Xtal Plate Protein Concentration (mg/ml)	Xtal Plate Temperature	Xtal Plate Barcode	Screen Batch Name	XTBM Row	XTBM Column	XTBM Subwell	XTBM Condition	Crystal Form Group	Cystal Form Annotation	Unit Cell Dimensions	Space group	Resolution	Number of Successful Autoprocessing Pipelines	Autoprocessing Pipeline Used	Experiment Time	Soaking condition	Xtal Group	Surface Mutations Around Nbinter-1	Key Mutations	Crystal Epitope Mutations	Condition Repitition?
G5-006	XX21RECQ L5A-x1368	Fresh	10.63	20	CI072128	HIN3-170302-01	F	8	c	0.2M ammonium sulfate -- 25% PEG3350 -- 0.1M HEPES pH 7.5	4	Tetramer I 2 2 2/ C 1 2 1	82.38 90.04 220.08 (90.0 90.0 90.0)	I 2 2 2	2.42	5	Xia2-DIALS	12/01/2021 13:48	20% DMSO - 0% EG	PEG	Q14K	S7N:L12 C:T125 M	Non-D69Y Crystal Epitopes	no
G5-006	XX21RECQ L5A-x1353	Fresh	40	20	CI072128	HIN3-170302-01	F	8	c	0.2M ammonium sulfate -- 25% PEG3350 -- 0.1M HEPES pH 7.5	4	Tetramer I 2 2 2/ C 1 2 1	234.71 89.58 164.74 (90.0 110.11 90.0)	C 1 2 1	2.43	7	Xia2-DIALS	12/01/2021 13:05	0% DMSO - 15% EG	PEG	Q14K	S7N:L12 C:T125 M	Non-D69Y Crystal Epitopes	no
G5-006	XX21RECQ L5A-x1363	Fresh	40	20	CI072128	HIN3-170302-01	F	8	c	0.2M ammonium sulfate -- 25% PEG3350 -- 0.1M HEPES pH 7.5	4	Tetramer I 2 2 2/ C 1 2 1	233.73 90.24 164.51 (90.0 110.05 90.0)	C 1 2 1	2.43	7	Xia2-DIALS	12/01/2021 13:33	10% DMSO - 15% EG	PEG	Q14K	S7N:L12 C:T125 M	Non-D69Y Crystal Epitopes	no
G5-006	XX21RECQ L5A-x1380	Fresh	10.63	20	CI072128	HIN3-170302-01	F	8	c	0.2M ammonium sulfate -- 25% PEG3350 -- 0.1M HEPES pH 7.5	4	Tetramer I 2 2 2/ C 1 2 1	227.48 89.46 79.95 (90.0 108.75 90.0)	C 1 2 1	2.44	5	Xia2-DIALS	12/01/2021 14:21	30% DMSO - 0% EG	PEG	Q14K	S7N:L12 C:T125 M	Non-D69Y Crystal Epitopes	no
G5-006	XX21RECQ L5A-x1349	Fresh	40	20	CI072128	HIN3-170302-01	F	8	c	0.2M ammonium sulfate -- 25% PEG3350 -- 0.1M HEPES pH 7.5	4	Tetramer I 2 2 2/ C 1 2 1	233.32 89.76 164.37 (90.0 109.93 90.0)	C 1 2 1	2.45	5	Xia2-DIALS	12/01/2021 12:54	0% DMSO - 0% EG	PEG	Q14K	S7N:L12 C:T125 M	Non-D69Y Crystal Epitopes	no
G5-006	XX21RECQ L5A-x1360	Fresh	40	20	CI072128	HIN3-170302-01	F	8	c	0.2M ammonium sulfate -- 25% PEG3350 -- 0.1M HEPES pH 7.5	4	Tetramer I 2 2 2/ C 1 2 1	236.1 89.8 164.97 (90.0 109.99 90.0)	C 1 2 1	2.46	5	Xia2-DIALS	12/01/2021 13:23	10% DMSO - 0% EG	PEG	Q14K	S7N:L12 C:T125 M	Non-D69Y Crystal Epitopes	no
G5-006	XX21RECQ L5A-x1365	Fresh	40	20	CI072128	HIN3-170302-01	F	8	c	0.2M ammonium sulfate -- 25% PEG3350 -- 0.1M HEPES pH 7.5	4	Tetramer I 2 2 2/ C 1 2 1	232.75 89.95 164.29 (90.0 110.22 90.0)	C 1 2 1	2.47	7	Xia2-DIALS	12/01/2021 13:39	10% DMSO - 15% EG	PEG	Q14K	S7N:L12 C:T125 M	Non-D69Y Crystal Epitopes	no
G5-006	XX21RECQ L5A-x0844	Frozen	22	20	CI080726	HIN3-170302-01	F	8	c	0.2M ammonium sulfate -- 25% PEG3350 -- 0.1M HEPES pH 7.5	4	Tetramer I 2 2 2/ C 1 2 1	215.35 90.03 79.74 (90.0 107.52 90.0)	C 1 2 1	2.48	5	Xia2-DIALS	17/12/2020 06:08	-	PEG	Q14K	S7N:L12 C:T125 M	Non-D69Y Crystal Epitopes	yes
G5-006	XX21RECQ L5A-x0849	Frozen	22	20	CI080726	HIN3-170302-01	F	8	c	0.2M ammonium sulfate -- 25% PEG3350 -- 0.1M HEPES pH 7.5	4	Tetramer I 2 2 2/ C 1 2 1	217.53 90.06 80.04 (90.0 107.38 90.0)	C 1 2 1	2.51	4	Xia2-DIALS	17/12/2020 06:23	-	PEG	Q14K	S7N:L12 C:T125 M	Non-D69Y Crystal Epitopes	yes
G5-006	XX21RECQ L5A-x1351	Fresh	40	20	CI072128	HIN3-170302-01	F	8	c	0.2M ammonium sulfate -- 25% PEG3350 -- 0.1M HEPES pH 7.5	4	Tetramer I 2 2 2/ C 1 2 1	234.15 89.55 163.2 (90.0 109.63 90.0)	C 1 2 1	2.51	5	Xia2-DIALS	12/01/2021 12:59	0% DMSO - 0% EG	PEG	Q14K	S7N:L12 C:T125 M	Non-D69Y Crystal Epitopes	no
G5-006	XX21RECQ L5A-x1389	Fresh	10.63	20	CI072128	HIN3-170302-01	F	8	c	0.2M ammonium sulfate -- 25% PEG3350 -- 0.1M HEPES pH 7.5	4	Tetramer I 2 2 2/ C 1 2 1	230.7 90.27 79.9 (90.0 109.01 90.0)	C 1 2 1	2.51	5	Xia2-DIALS	12/01/2021 14:46	15% DMSO - 0% EG	PEG	Q14K	S7N:L12 C:T125 M	Non-D69Y Crystal Epitopes	no
G5-006	XX21RECQ L5A-x1387	Fresh	10.63	20	CI072128	HIN3-170302-01	F	8	c	0.2M ammonium sulfate -- 25% PEG3350 -- 0.1M HEPES pH 7.5	-1	NA	229.58 79.17 89.89 (90.0 90.0 90.0)	P 21 21 2	2.51	7	Xia2-DIALS	12/01/2021 14:40	30% DMSO - 15% EG	PEG	Q14K	S7N:L12 C:T125 M	Non-D69Y Crystal Epitopes	no
G5-006	XX21RECQ L5A-x1361	Fresh	40	20	CI072128	HIN3-170302-01	F	8	c	0.2M ammonium sulfate -- 25% PEG3350 -- 0.1M HEPES pH 7.5	4	Tetramer I 2 2 2/ C 1 2 1	234.45 89.73 164.16 (90.0 109.82 90.0)	C 1 2 1	2.52	7	Xia2-DIALS	12/01/2021 13:26	10% DMSO - 0% EG	PEG	Q14K	S7N:L12 C:T125 M	Non-D69Y Crystal Epitopes	no
G5-006	XX21RECQ L5A-x1375	Fresh	10.63	20	CI072128	HIN3-170302-01	F	8	c	0.2M ammonium sulfate -- 25% PEG3350 -- 0.1M HEPES pH 7.5	4	Tetramer I 2 2 2/ C 1 2 1	228.61 89.55 80.09 (90.0 108.8 90.0)	C 1 2 1	2.52	4	Xia2-DIALS	12/01/2021 14:07	20% DMSO - 15% EG	PEG	Q14K	S7N:L12 C:T125 M	Non-D69Y Crystal Epitopes	no
G5-006	XX21RECQ L5A-x0760	Fresh	20.04	20	CI080566	HIN3-170302-01	F	8	c	0.2M ammonium sulfate -- 25% PEG3350 -- 0.1M HEPES pH 7.5	4	Tetramer I 2 2 2/ C 1 2 1	234.4 89.79 80.84 (90.0 109.46 90.0)	C 1 2 1	2.53	5	Xia2-DIALS	04/12/2020 07:12	-	PEG	Q14K	S7N:L12 C:T125 M	Non-D69Y Crystal Epitopes	no

Table S4 Diffraction data

Nanobody grouped ID	Mounted Crystal ID	Xtal Plate Protein Frozen/Fresh?	Xtal Plate Protein Concentration (mg/ml)	Xtal Plate Temperature	Xtal Plate Barcode	Screen Batch Name	XTBM Row	XTBM Column	XTBM Subwell	XTBM Condition	Crystal Form Group	Cystal Form Annotation	Unit Cell Dimensions	Space group	Resolution	Number of Successful Autoprocessing Pipelines	Autoprocessing Pipeline Used	Experiment Time	Soaking condition	Xtal Group	Surface Mutations Around Nbinter-1	Key Mutations	Crystal Epitope Mutations	Condition Repitition?
G5-006	XX21RECQ L5A-x1386	Fresh	40	20	CI072128	HIN3-170302-01	F	8	c	0.2M ammonium sulfate -- 25% PEG3350 -- 0.1M HEPES pH 7.5	4	Tetramer I 2 2 2/ C 1 2 1	235.02 89.86 165.66 (90.0 110.16 90.0)	C 1 2 1	2.55	5	Xia2-DIALS	12/01/2021 14:38	30% DMSO - 15% EG	PEG	Q14K	S7N:L12 C:T125 M	Non-D69Y Crystal Epitopes	no
G5-006	XX21RECQ L5A-x0851	Frozen	22	20	CI080726	HIN3-170302-01	F	8	c	0.2M ammonium sulfate -- 25% PEG3350 -- 0.1M HEPES pH 7.5	4	Tetramer I 2 2 2/ C 1 2 1	216.35 89.74 79.75 (90.0 107.46 90.0)	C 1 2 1	2.56	5	Xia2-DIALS	17/12/2020 06:28	-	PEG	Q14K	S7N:L12 C:T125 M	Non-D69Y Crystal Epitopes	yes
G5-006	XX21RECQ L5A-x1350	Fresh	40	20	CI072128	HIN3-170302-01	F	8	c	0.2M ammonium sulfate -- 25% PEG3350 -- 0.1M HEPES pH 7.5	4	Tetramer I 2 2 2/ C 1 2 1	235.64 90.24 165.26 (90.0 110.1 90.0)	C 1 2 1	2.56	5	Xia2-DIALS	12/01/2021 12:57	0% DMSO - 0% EG	PEG	Q14K	S7N:L12 C:T125 M	Non-D69Y Crystal Epitopes	no
G5-006	XX21RECQ L5A-x1377	Fresh	10.63	20	CI072128	HIN3-170302-01	F	8	c	0.2M ammonium sulfate -- 25% PEG3350 -- 0.1M HEPES pH 7.5	4	Tetramer I 2 2 2/ C 1 2 1	229.34 89.78 80.54 (90.0 109.69 90.0)	C 1 2 1	2.58	7	Xia2-DIALS	12/01/2021 14:13	20% DMSO - 15% EG	PEG	Q14K	S7N:L12 C:T125 M	Non-D69Y Crystal Epitopes	no
G5-006	XX21RECQ L5A-x1383	Fresh	10.63	20	CI072128	HIN3-170302-01	F	8	c	0.2M ammonium sulfate -- 25% PEG3350 -- 0.1M HEPES pH 7.5	4	Tetramer I 2 2 2/ C 1 2 1	229.89 89.52 80.27 (90.0 108.91 90.0)	C 1 2 1	2.62	7	Xia2-3dii	12/01/2021 14:29	30% DMSO - 15% EG	PEG	Q14K	S7N:L12 C:T125 M	Non-D69Y Crystal Epitopes	no
G5-006	XX21RECQ L5A-x0764	Fresh	20.04	20	CI080566	HIN3-170302-01	H	2	d	0.2M sodium/potassium tartrate -- 20% PEG3350	4	Tetramer I 2 2 2/ C 1 2 1	231.8 90.2 81.02 (90.0 109.55 90.0)	C 1 2 1	2.68	4	Xia2-DIALS	04/12/2020 07:22	-	PEG	Q14K	S7N:L12 C:T125 M	Non-D69Y Crystal Epitopes	no
G5-006	XX21RECQ L5A-x1388	Fresh	10.63	20	CI072128	HIN3-170302-01	F	8	c	0.2M ammonium sulfate -- 25% PEG3350 -- 0.1M HEPES pH 7.5	-1	NA	78.98 89.66 224.14 (90.0 90.09 90.0)	P 1 2 1	2.68	5	Xia2-DIALS	12/01/2021 14:43	15% DMSO - 0% EG	PEG	Q14K	S7N:L12 C:T125 M	Non-D69Y Crystal Epitopes	no
G5-006	XX21RECQ L5A-x0847	Frozen	22	20	CI080726	HIN3-170302-01	F	8	c	0.2M ammonium sulfate -- 25% PEG3350 -- 0.1M HEPES pH 7.5	4	Tetramer I 2 2 2/ C 1 2 1	215.99 90.14 79.75 (90.0 107.41 90.0)	C 1 2 1	2.71	5	Xia2-DIALS	17/12/2020 06:16	-	PEG	Q14K	S7N:L12 C:T125 M	Non-D69Y Crystal Epitopes	yes
G5-006	XX21RECQ L5A-x1348	Fresh	40	20	CI072128	HIN3-170302-01	F	8	c	0.2M ammonium sulfate -- 25% PEG3350 -- 0.1M HEPES pH 7.5	4	Tetramer I 2 2 2/ C 1 2 1	233.57 89.54 164.51 (90.0 109.97 90.0)	C 1 2 1	2.74	5	Xia2-DIALS	12/01/2021 12:52	0% DMSO - 0% EG	PEG	Q14K	S7N:L12 C:T125 M	Non-D69Y Crystal Epitopes	no
G5-006	XX21RECQ L5A-x0762	Fresh	20.04	20	CI080566	HIN3-170302-01	G	1	d	0.2M sodium chloride -- 25% PEG3350 -- 0.1M tris pH 8.5	4	Tetramer I 2 2 2/ C 1 2 1	217.72 90.69 79.96 (90.0 108.06 90.0)	C 1 2 1	2.78	5	Xia2-DIALS	04/12/2020 07:19	-	PEG	Q14K	S7N:L12 C:T125 M	Non-D69Y Crystal Epitopes	no
G5-006	XX21RECQ L5A-x0846	Frozen	22	20	CI080726	HIN3-170302-01	F	8	c	0.2M ammonium sulfate -- 25% PEG3350 -- 0.1M HEPES pH 7.5	4	Tetramer I 2 2 2/ C 1 2 1	216.53 90.2 79.92 (90.0 107.45 90.0)	C 1 2 1	2.81	5	Xia2-DIALS	17/12/2020 06:13	-	PEG	Q14K	S7N:L12 C:T125 M	Non-D69Y Crystal Epitopes	yes
G5-006	XX21RECQ L5A-x1359	Fresh	10.63	20	CI072128	HIN3-170302-01	F	8	c	0.2M ammonium sulfate -- 25% PEG3350 -- 0.1M HEPES pH 7.5	4	Tetramer I 2 2 2/ C 1 2 1	82.47 89.82 220.0 (90.0 90.0 90.0)	I 2 2 2	2.94	3	Xia2-DIALS	12/01/2021 13:21	10% DMSO - 0% EG	PEG	Q14K	S7N:L12 C:T125 M	Non-D69Y Crystal Epitopes	no
G5-006	XX21RECQ L5A-x0761	Fresh	20.04	20	CI080566	HIN3-170302-01	F	9	c	0.2M ammonium sulfate -- 25% PEG3350 -- 0.1M tris pH 8.5	4	Tetramer I 2 2 2/ C 1 2 1	220.25 90.0 80.34 (90.0 107.94 90.0)	C 1 2 1	2.98	4	Xia2-DIALS	04/12/2020 07:16	-	PEG	Q14K	S7N:L12 C:T125 M	Non-D69Y Crystal Epitopes	no
G5-006	XX21RECQ L5A-x0855	Frozen	22	20	CI080726	HIN3-170302-01	F	9	a	0.2M ammonium sulfate -- 25% PEG3350 -- 0.1M tris pH 8.5	4	Tetramer I 2 2 2/ C 1 2 1	79.91 89.85 222.18 (90.0 90.0 90.0)	I 2 2 2	3.14	5	Xia2-DIALS	17/12/2020 06:38	-	PEG	Q14K	S7N:L12 C:T125 M	Non-D69Y Crystal Epitopes	yes
G5-006	XX21RECQ L5A-x0858	Frozen	22	20	CI080726	HIN3-170302-01	F	9	a	0.2M ammonium sulfate -- 25% PEG3350 -- 0.1M tris pH 8.5	4	Tetramer I 2 2 2/ C 1 2 1	219.29 90.24 80.06 (90.0 108.02 90.0)	C 1 2 1	3.24	2	Xia2-DIALS	17/12/2020 06:47	-	PEG	Q14K	S7N:L12 C:T125 M	Non-D69Y Crystal Epitopes	yes

Table S4 Diffraction data

Nanobody grouped ID	Mounted Crystal ID	Xtal Plate Protein Frozen/Fresh?	Xtal Plate Protein Concentration (mg/ml)	Xtal Plate Temperature	Xtal Plate Barcode	Screen Batch Name	XTBM Row	XTBM Column	XTBM Subwell	XTBM Condition	Crystal Form Group	Cystal Form Annotation	Unit Cell Dimensions	Space group	Resolution	Number of Successful Autoprocessing Pipelines	Autoprocessing Pipeline Used	Experiment Time	Soaking condition	Xtal Group	Surface Mutations Around Nbinter-1	Key Mutations	Crystal Epitope Mutations	Condition Repitition?
G5-006	XX21RECQ L5A-x0845	Frozen	22	20	CI080726	HIN3-170302-01	F	8	c	0.2M ammonium sulfate -- 25% PEG3350 -- 0.1M HEPES pH 7.5	4	Tetramer I 2 2 2/ C 1 2 1	217.12 90.26 79.59 (90.0 107.02 90.0)	C 1 2 1	3.26	5	Xia2-DIALS	17/12/2020 06:11	-	PEG	Q14K	S7N:L12 C:T125 M	Non-D69Y Crystal Epitopes	yes
G5-006	XX21RECQ L5A-x0853	Frozen	22	20	CI080726	HIN3-170302-01	F	9	a	0.2M ammonium sulfate -- 25% PEG3350 -- 0.1M tris pH 8.5	4	Tetramer I 2 2 2/ C 1 2 1	80.1 89.93 222.82 (90.0 90.0 90.0)	I 2 2 2	3.33	2	Xia2-DIALS	17/12/2020 06:33	-	PEG	Q14K	S7N:L12 C:T125 M	Non-D69Y Crystal Epitopes	yes
G5-006	XX21RECQ L5A-x0857	Frozen	22	20	CI080726	HIN3-170302-01	F	9	a	0.2M ammonium sulfate -- 25% PEG3350 -- 0.1M tris pH 8.5	4	Tetramer I 2 2 2/ C 1 2 1	220.47 90.48 80.31 (90.0 107.75 90.0)	C 1 2 1	3.34	4	Xia2-3dii	17/12/2020 06:44	-	PEG	Q14K	S7N:L12 C:T125 M	Non-D69Y Crystal Epitopes	yes
G5-006	XX21RECQ L5A-x0861	Frozen	22	20	CI080726	HIN3-170302-01	F	9	a	0.2M ammonium sulfate -- 25% PEG3350 -- 0.1M tris pH 8.5	4	Tetramer I 2 2 2/ C 1 2 1	81.23 91.22 224.67 (90.0 90.0 90.0)	I 2 2 2	3.37	4	Xia2-DIALS	17/12/2020 06:55	-	PEG	Q14K	S7N:L12 C:T125 M	Non-D69Y Crystal Epitopes	yes
G5-006	XX21RECQ L5A-x0755	Fresh	20.04	20	CI080566	HIN3-170302-01	B	7	a	0.056M sodium phosphate monobasic -- 1.344M potassium phosphate dibasic	2	Tetramer P 2 2 21 Porous	90.11 152.16 271.24 (90.0 90.0 90.0)	P 2 2 2	4	5	Xia2-DIALS	03/12/2020 23:51	-	High Salt	Q14K	S7N:L12 C:T125 M	Non-D69Y Crystal Epitopes	no
G5-006	XX21RECQ L5A-x0854	Frozen	22	20	CI080726	HIN3-170302-01	F	9	a	0.2M ammonium sulfate -- 25% PEG3350 -- 0.1M tris pH 8.5	-1	NA	90.23 223.0 80.25 (90.0 90.0 90.0)	P 2 2 21	4.02	3	Autoproc	17/12/2020 06:36	-	PEG	Q14K	S7N:L12 C:T125 M	Non-D69Y Crystal Epitopes	yes
G5-006	XX21RECQ L5A-x0859	Frozen	22	20	CI080726	HIN3-170302-01	F	9	a	0.2M ammonium sulfate -- 25% PEG3350 -- 0.1M tris pH 8.5	4	Tetramer I 2 2 2/ C 1 2 1	217.91 90.17 79.86 (90.0 108.15 90.0)	C 1 2 1	4.03	4	Xia2-3dii	17/12/2020 06:50	-	PEG	Q14K	S7N:L12 C:T125 M	Non-D69Y Crystal Epitopes	yes
G5-006	XX21RECQ L5A-x0865	Frozen	22	20	CI080726	HIN3-170302-01	G	1	c	0.2M sodium chloride -- 25% PEG3350 -- 0.1M tris pH 8.5	4	Tetramer I 2 2 2/ C 1 2 1	79.59 90.31 220.16 (90.0 90.0 90.0)	I 2 2 2	4.09	2	Xia2-DIALS	17/12/2020 07:07	-	PEG	Q14K	S7N:L12 C:T125 M	Non-D69Y Crystal Epitopes	yes
G5-006	XX21RECQ L5A-x0753	Fresh	20.04	20	CI080566	HIN3-170302-01	A	12	a	3M sodium chloride -- 0.1M tris pH 8.5	2	Tetramer P 2 2 21 Porous	89.58 152.99 271.84 (90.0 90.0 90.0)	P 2 21 21	4.9	5	Xia2-DIALS	03/12/2020 23:44	-	High Salt	Q14K	S7N:L12 C:T125 M	Non-D69Y Crystal Epitopes	no
G5-006	XX21RECQ L5A-x0856	Frozen	22	20	CI080726	HIN3-170302-01	F	9	a	0.2M ammonium sulfate -- 25% PEG3350 -- 0.1M tris pH 8.5	4	Tetramer I 2 2 2/ C 1 2 1	82.69 89.21 201.74 (90.0 90.0 90.0)	I 2 2 2	5.4	1	Xia2-3dii	17/12/2020 06:41	-	PEG	Q14K	S7N:L12 C:T125 M	Non-D69Y Crystal Epitopes	yes
G5-006	XX21RECQ L5A-x0754	Fresh	20.04	20	CI080566	HIN3-170302-01	A	12	c	3M sodium chloride -- 0.1M tris pH 8.5	2	Tetramer P 2 2 21 Porous	90.02 150.75 274.79 (90.0 90.0 90.0)	P 2 21 21	5.56	1	Xia2-DIALS	03/12/2020 23:47	-	High Salt	Q14K	S7N:L12 C:T125 M	Non-D69Y Crystal Epitopes	no
G5-006	XX21RECQ L5A-x0767	Fresh	20.04	20	CI080566	HIN3-170302-01	H	6	d	0.2M sodium formate -- 20% PEG3350	4	Tetramer I 2 2 2/ C 1 2 1	80.76 90.87 216.53 (90.0 90.0 90.0)	I 2 2 2	5.92	2	Xia2-3dii	04/12/2020 07:31	-	PEG	Q14K	S7N:L12 C:T125 M	Non-D69Y Crystal Epitopes	no
G5-006	XX21RECQ L5A-x0758	Fresh	20.04	20	CI080566	HIN3-170302-01	C	3	a	2.4M sodium malonate	2	Tetramer P 2 2 21 Porous	155.98 272.43 90.57 (90.0 90.0 90.0)	P 21 21 2	6.35	1	Xia2-3dii	04/12/2020 07:06	-	High Salt	Q14K	S7N:L12 C:T125 M	Non-D69Y Crystal Epitopes	no
G5-006	XX21RECQ L5A-x0757	Fresh	20.04	20	CI080566	HIN3-170302-01	C	1	a	3.5M sodium formate	2	Tetramer P 2 2 21 Porous	90.16 149.49 269.81 (90.0 90.0 90.0)	P 2 21 21	6.36	5	Xia2-DIALS	04/12/2020 07:03	-	High Salt	Q14K	S7N:L12 C:T125 M	Non-D69Y Crystal Epitopes	no
G5-006	XX21RECQ L5A-x0756	Fresh	20.04	20	CI080566	HIN3-170302-01	B	7	c	0.056M sodium phosphate monobasic -- 1.344M potassium phosphate dibasic	2	Tetramer P 2 2 21 Porous	89.94 144.19 272.6 (90.0 90.0 90.0)	P 2 2 2	6.38	3	Xia2-3dii	04/12/2020 06:59	-	High Salt	Q14K	S7N:L12 C:T125 M	Non-D69Y Crystal Epitopes	no

Table S4 Diffraction data

Nanobody grouped ID	Mounted Crystal ID	Xtal Plate Protein Frozen/Fresh?	Xtal Plate Protein Concentration (mg/ml)	Xtal Plate Temperature	Xtal Plate Barcode	Screen Batch Name	XTBM Row	XTBM Column	XTBM Subwell	XTBM Condition	Crystal Form Group	Cystal Form Annotation	Unit Cell Dimensions	Space group	Resolution	Number of Successful Autoprocessing Pipelines	Autoprocessing Pipeline Used	Experiment Time	Soaking condition	Xtal Group	Surface Mutations Around Nbinter-1	Key Mutations	Crystal Epitope Mutations	Condition Repitition?
G5-006	XX21RECQL5A-x0759	Fresh	20.04	20	CI080566	HIN3-170302-01	C	9	c	1.1M sodium malonate -- 0.5% jeffamine ED-2003 -- 0.1M HEPES pH 7.0	NA		NA	NA	NA	0	NA	04/12/2020 07:09	-	High Salt	Q14K	S7N:L12C:T125M	Non-D69Y Crystal Epitopes	no
G5-006	XX21RECQL5A-x0872	Frozen	22	20	CI080726	HIN3-170302-01	G	1	c	0.2M sodium chloride -- 25% PEG3350 -- 0.1M tris pH 8.5	NA		NA	NA	NA	0	NA	17/12/2020 07:25	-	PEG	Q14K	S7N:L12C:T125M	Non-D69Y Crystal Epitopes	yes
G5-006	XX21RECQL5A-x0871	Frozen	22	20	CI080726	HIN3-170302-01	G	1	c	0.2M sodium chloride -- 25% PEG3350 -- 0.1M tris pH 8.5	NA		NA	NA	NA	0	NA	17/12/2020 07:22	-	PEG	Q14K	S7N:L12C:T125M	Non-D69Y Crystal Epitopes	yes
G5-006	XX21RECQL5A-x0870	Frozen	22	20	CI080726	HIN3-170302-01	G	1	c	0.2M sodium chloride -- 25% PEG3350 -- 0.1M tris pH 8.5	NA		NA	NA	NA	0	NA	17/12/2020 07:19	-	PEG	Q14K	S7N:L12C:T125M	Non-D69Y Crystal Epitopes	yes
G5-006	XX21RECQL5A-x0869	Frozen	22	20	CI080726	HIN3-170302-01	G	1	c	0.2M sodium chloride -- 25% PEG3350 -- 0.1M tris pH 8.5	NA		NA	NA	NA	0	NA	17/12/2020 07:17	-	PEG	Q14K	S7N:L12C:T125M	Non-D69Y Crystal Epitopes	yes
G5-006	XX21RECQL5A-x0868	Frozen	22	20	CI080726	HIN3-170302-01	G	1	c	0.2M sodium chloride -- 25% PEG3350 -- 0.1M tris pH 8.5	NA		NA	NA	NA	0	NA	17/12/2020 07:14	-	PEG	Q14K	S7N:L12C:T125M	Non-D69Y Crystal Epitopes	yes
G5-006	XX21RECQL5A-x0867	Frozen	22	20	CI080726	HIN3-170302-01	G	1	c	0.2M sodium chloride -- 25% PEG3350 -- 0.1M tris pH 8.5	NA		NA	NA	NA	0	NA	17/12/2020 07:12	-	PEG	Q14K	S7N:L12C:T125M	Non-D69Y Crystal Epitopes	yes
G5-006	XX21RECQL5A-x0866	Frozen	22	20	CI080726	HIN3-170302-01	G	1	c	0.2M sodium chloride -- 25% PEG3350 -- 0.1M tris pH 8.5	NA		NA	NA	NA	0	NA	17/12/2020 07:09	-	PEG	Q14K	S7N:L12C:T125M	Non-D69Y Crystal Epitopes	yes
G5-006	XX21RECQL5A-x0864	Frozen	22	20	CI080726	HIN3-170302-01	G	1	c	0.2M sodium chloride -- 25% PEG3350 -- 0.1M tris pH 8.5	NA		NA	NA	NA	0	NA	17/12/2020 07:03	-	PEG	Q14K	S7N:L12C:T125M	Non-D69Y Crystal Epitopes	yes
G5-006	XX21RECQL5A-x0863	Frozen	22	20	CI080726	HIN3-170302-01	G	1	c	0.2M sodium chloride -- 25% PEG3350 -- 0.1M tris pH 8.5	NA		NA	NA	NA	0	NA	17/12/2020 07:00	-	PEG	Q14K	S7N:L12C:T125M	Non-D69Y Crystal Epitopes	yes
G5-006	XX21RECQL5A-x0862	Frozen	22	20	CI080726	HIN3-170302-01	F	9	a	0.2M ammonium sulfate -- 25% PEG3350 -- 0.1M tris pH 8.5	NA		NA	NA	NA	0	NA	17/12/2020 06:57	-	PEG	Q14K	S7N:L12C:T125M	Non-D69Y Crystal Epitopes	yes
G5-006	XX21RECQL5A-x0860	Frozen	22	20	CI080726	HIN3-170302-01	F	9	a	0.2M ammonium sulfate -- 25% PEG3350 -- 0.1M tris pH 8.5	NA		NA	NA	NA	0	NA	17/12/2020 06:52	-	PEG	Q14K	S7N:L12C:T125M	Non-D69Y Crystal Epitopes	yes
G5-006	XX21RECQL5A-x0852	Frozen	22	20	CI080726	HIN3-170302-01	F	9	a	0.2M ammonium sulfate -- 25% PEG3350 -- 0.1M tris pH 8.5	NA		NA	NA	NA	0	NA	17/12/2020 06:30	-	PEG	Q14K	S7N:L12C:T125M	Non-D69Y Crystal Epitopes	yes
G5-006	XX21RECQL5A-x0848	Frozen	22	20	CI080726	HIN3-170302-01	F	8	c	0.2M ammonium sulfate -- 25% PEG3350 -- 0.1M HEPES pH 7.5	NA		NA	NA	NA	0	NA	17/12/2020 06:19	-	PEG	Q14K	S7N:L12C:T125M	Non-D69Y Crystal Epitopes	yes
G5-006	XX21RECQL5A-x0843	Frozen	22	20	CI080726	HIN3-170302-01	F	8	c	0.2M ammonium sulfate -- 25% PEG3350 -- 0.1M HEPES pH 7.5	NA		NA	NA	NA	0	NA	17/12/2020 06:05	-	PEG	Q14K	S7N:L12C:T125M	Non-D69Y Crystal Epitopes	yes

Table S4 Diffraction data

Nanobody grouped ID	Mounted Crystal ID	Xtal Plate Protein Frozen/Fresh?	Xtal Plate Protein Concentration (mg/ml)	Xtal Plate Temperature	Xtal Plate Barcode	Screen Batch Name	XTBM Row	XTBM Column	XTBM Subwell	XTBM Condition	Crystal Form Group	Cystal Form Annotation	Unit Cell Dimensions	Space group	Resolution	Number of Successful Autoprocessing Pipelines	Autoprocessing Pipeline Used	Experiment Time	Soaking condition	Xtal Group	Surface Mutations Around Nbinter-1	Key Mutations	Crystal Epitope Mutations	Condition Repitition?
G5-006	XX21RECQL5A-x0841	Frozen	22	20	CI080726	HIN3-170302-01	F	8	c	0.2M ammonium sulfate -- 25% PEG3350 -- 0.1M HEPES pH 7.5	NA		NA	NA	NA	0	NA	17/12/2020 05:59	-	PEG	Q14K	S7N:L12C:T125M	Non-D69Y Crystal Epitopes	yes
G5-006	XX21RECQL5A-x0840	Frozen	22	20	CI080726	HIN3-170302-01	F	8	c	0.2M ammonium sulfate -- 25% PEG3350 -- 0.1M HEPES pH 7.5	NA		NA	NA	NA	0	NA	17/12/2020 05:55	-	PEG	Q14K	S7N:L12C:T125M	Non-D69Y Crystal Epitopes	yes
G5-006	XX21RECQL5A-x1358	Fresh	10.63	20	CI072128	HIN3-170302-01	F	8	c	0.2M ammonium sulfate -- 25% PEG3350 -- 0.1M HEPES pH 7.5	NA		NA	NA	NA	0	NA	12/01/2021 13:18	10% DMSO - 0% EG	PEG	-	S7N:L12C:T125M	Non-D69Y Crystal Epitopes	no
G5-007	XX21RECQL5A-x0774	Fresh	21.61	20	CI080535	HIN3-170302-01	F	8	c	0.2M ammonium sulfate -- 25% PEG3350 -- 0.1M HEPES pH 7.5	4	Tetramer I 2 2 2/ C 1 2 1	226.87 90.17 80.59 (90.0 108.98 90.0)	C 1 2 1	2.58	4	Xia2-DIALS	02/12/2020 20:01	-	PEG	Q14K	S7N:L12C:T125M	Non-D69Y Crystal Epitopes	no
G5-007	XX21RECQL5A-x0772	Fresh	21.61	20	CI080535	HIN3-170302-01	F	7	d	0.2M ammonium sulfate -- 25% PEG3350 -- 0.1M bis-tris pH 6.5	4	Tetramer I 2 2 2/ C 1 2 1	229.04 90.07 80.56 (90.0 109.21 90.0)	C 1 2 1	3.04	4	Xia2-DIALS	02/12/2020 19:55	-	PEG	Q14K	S7N:L12C:T125M	Non-D69Y Crystal Epitopes	no
G5-007	XX21RECQL5A-x0780	Fresh	21.61	20	CI080535	HIN3-170302-01	G	4	c	0.2M lithium sulfate -- 25% PEG3350 -- 0.1M HEPES pH 7.5	4	Tetramer I 2 2 2/ C 1 2 1	218.93 90.61 80.18 (90.0 108.66 90.0)	C 1 2 1	3.15	4	Xia2-DIALS	04/12/2020 08:12	-	PEG	Q14K	S7N:L12C:T125M	Non-D69Y Crystal Epitopes	no
G5-007	XX21RECQL5A-x0782	Fresh	21.61	20	CI080535	HIN3-170302-01	H	6	c	0.2M sodium formate -- 20% PEG3350	4	Tetramer I 2 2 2/ C 1 2 1	80.39 90.63 216.04 (90.0 90.0 90.0)	I 2 2 2	3.41	2	Xia2-DIALS	03/12/2020 09:00	-	PEG	Q14K	S7N:L12C:T125M	Non-D69Y Crystal Epitopes	no
G5-007	XX21RECQL5A-x0769	Fresh	21.61	20	CI080535	HIN3-170302-01	C	1	a	3.5M sodium formate	2	Tetramer P 2 2 21 Porous	90.68 151.47 267.59 (90.0 90.0 90.0)	P 2 2 2	3.76	5	Xia2-DIALS	04/12/2020 07:37	-	High Salt	Q14K	S7N:L12C:T125M	Non-D69Y Crystal Epitopes	no
G5-007	XX21RECQL5A-x0775	Fresh	21.61	20	CI080535	HIN3-170302-01	F	8	d	0.2M ammonium sulfate -- 25% PEG3350 -- 0.1M HEPES pH 7.5	4	Tetramer I 2 2 2/ C 1 2 1	218.5 89.99 79.83 (90.0 108.93 90.0)	C 1 2 1	3.99	4	Xia2-DIALS	02/12/2020 20:04	-	PEG	Q14K	S7N:L12C:T125M	Non-D69Y Crystal Epitopes	no
G5-007	XX21RECQL5A-x0781	Fresh	21.61	20	CI080535	HIN3-170302-01	H	6	a	0.2M sodium formate -- 20% PEG3350	-1	NA	121.5 216.73 81.27 (90.0 131.83 90.0)	C 1 2 1	4.07	2	Xia2-DIALS	03/12/2020 08:57	-	PEG	Q14K	S7N:L12C:T125M	Non-D69Y Crystal Epitopes	no
G5-007	XX21RECQL5A-x0770	Fresh	21.61	20	CI080535	HIN3-170302-01	C	4	a	35% tacsimate	2	Tetramer P 2 2 21 Porous	90.19 148.04 269.29 (90.0 90.0 90.0)	P 2 2 21	4.11	5	Xia2-DIALS	04/12/2020 07:41	-	High Salt	Q14K	S7N:L12C:T125M	Non-D69Y Crystal Epitopes	no
G5-007	XX21RECQL5A-x0779	Fresh	21.61	20	CI080535	HIN3-170302-01	G	4	a	0.2M lithium sulfate -- 25% PEG3350 -- 0.1M HEPES pH 7.5	NA		NA	NA	NA	0	NA	02/12/2020 20:29	-	PEG	Q14K	S7N:L12C:T125M	Non-D69Y Crystal Epitopes	no
G5-007	XX21RECQL5A-x0778	Fresh	21.61	20	CI080535	HIN3-170302-01	G	1	c	0.2M sodium chloride -- 25% PEG3350 -- 0.1M tris pH 8.5	NA		NA	NA	NA	0	NA	02/12/2020 20:13	-	PEG	Q14K	S7N:L12C:T125M	Non-D69Y Crystal Epitopes	no
G5-007	XX21RECQL5A-x0777	Fresh	21.61	20	CI080535	HIN3-170302-01	F	9	d	0.2M ammonium sulfate -- 25% PEG3350 -- 0.1M tris pH 8.5	NA		NA	NA	NA	0	NA	02/12/2020 20:11	-	PEG	Q14K	S7N:L12C:T125M	Non-D69Y Crystal Epitopes	no
G5-007	XX21RECQL5A-x0776	Fresh	21.61	20	CI080535	HIN3-170302-01	F	9	c	0.2M ammonium sulfate -- 25% PEG3350 -- 0.1M tris pH 8.5	NA		NA	NA	NA	0	NA	02/12/2020 20:07	-	PEG	Q14K	S7N:L12C:T125M	Non-D69Y Crystal Epitopes	no

Table S4 Diffraction data

Nanobody grouped ID	Mounted Crystal ID	Xtal Plate Protein Frozen/Fresh?	Xtal Plate Protein Concentration (mg/ml)	Xtal Plate Temperature	Xtal Plate Barcode	Screen Batch Name	XTBM Row	XTBM Column	XTBM Subwell	XTBM Condition	Crystal Form Group	Cystal Form Annotation	Unit Cell Dimensions	Space group	Resolution	Number of Successful Autoprocessing Pipelines	Autoprocessing Pipeline Used	Experiment Time	Soaking condition	Xtal Group	Surface Mutations Around Nbinter-1	Key Mutations	Crystal Epitope Mutations	Condition Repitition?
G5-007	XX21RECQL5A-x0773	Fresh	21.61	20	CI080535	HIN3-170302-01	F	8	a	0.2M ammonium sulfate -- 25% PEG3350 -- 0.1M HEPES pH 7.5	NA		NA	NA	NA	0	NA	02/12/2020 19:58	-	PEG	Q14K	S7N:L12C:T125M	Non-D69Y Crystal Epitopes	no
G5-007	XX21RECQL5A-x0771	Fresh	21.61	20	CI080535	HIN3-170302-01	F	7	c	0.2M ammonium sulfate -- 25% PEG3350 -- 0.1M bis-tris pH 6.5	NA		NA	NA	NA	0	NA	04/12/2020 07:44	-	PEG	Q14K	S7N:L12C:T125M	Non-D69Y Crystal Epitopes	no
G5-007	XX21RECQL5A-x0768	Fresh	21.61	20	CI080535	HIN3-170302-01	B	6	c	0.49M sodium phosphate monobasic -- 0.91M potassium phosphate dibasic	NA		NA	NA	NA	0	NA	04/12/2020 07:34	-	High Salt	Q14K	S7N:L12C:T125M	Non-D69Y Crystal Epitopes	no
G5-009	XX21RECQL5A-x11128	Fresh	22.6	20	CI080525	HIN3-170302-01	F	8	c	0.2M ammonium sulfate -- 25% PEG3350 -- 0.1M HEPES pH 7.5	4	Tetramer I 2 2 2/ C 1 2 1	81.02 89.65 218.72(90.0 90.0 90.0)	I 2 2 2	2.18	1	Xia2-DIALS	02/12/2020 02:44	-	PEG	Q14K	S7N:L12C:T125M	Non-D69Y Crystal Epitopes	no
G5-009	XX21RECQL5A-x11134	Fresh	22.6	20	CI080525	HIN3-170302-01	G	1	c	0.2M sodium chloride -- 25% PEG3350 -- 0.1M tris pH 8.5	4	Tetramer I 2 2 2/ C 1 2 1	82.0 90.10 216.10 (90.0 90.0 90.0)	I 2 2 2	2.2	8	Xia2-DIALS	02/12/2020 12:20	-	PEG	Q14K	S7N:L12C:T125M	Non-D69Y Crystal Epitopes	no
G5-009	XX21RECQL5A-x11127	Fresh	22.6	20	CI080525	HIN3-170302-01	F	8	a	0.2M ammonium sulfate -- 25% PEG3350 -- 0.1M HEPES pH 7.5	4	Tetramer I 2 2 2/ C 1 2 1	80.91 89.38 218.54(90.0 90.0 90.0)	I 2 2 2	2.42	5	Xia2-DIALS	02/12/2020 02:39	-	PEG	Q14K	S7N:L12C:T125M	Non-D69Y Crystal Epitopes	no
G5-009	XX21RECQL5A-x11133	Fresh	22.6	20	CI080525	HIN3-170302-01	G	1	a	0.2M sodium chloride -- 25% PEG3350 -- 0.1M tris pH 8.5	4	Tetramer I 2 2 2/ C 1 2 1	81.65 90.10 216.17(90.0 90.0 90.0)	I 2 2 2	2.48	5	Xia2-DIALS	02/12/2020 03:08	-	PEG	Q14K	S7N:L12C:T125M	Non-D69Y Crystal Epitopes	no
G5-009	XX21RECQL5A-x11117	Fresh	22.6	20	CI080525	HIN3-170302-01	A	4	a	2M ammonium sulfate -- 0.1M bis-tris pH 6.5	4	Tetramer I 2 2 2/ C 1 2 1	79.90 89.85 222.25 (90.0 90.0 90.0)	I 2 2 2	2.9	7	Xia2-DIALS	02/12/2020 10:09	-	High Salt	Q14K	S7N:L12C:T125M	Non-D69Y Crystal Epitopes	no
G5-009	XX21RECQL5A-x11137	Fresh	22.6	20	CI080525	HIN3-170302-01	H	6	d	0.2M sodium formate -- 20% PEG3350	4	Tetramer I 2 2 2/ C 1 2 1	81.07 89.32 215.72(90.0 90.0 90.0)	I 2 2 2	3.11	4	Xia2-DIALS	02/12/2020 10:05	-	PEG	Q14K	S7N:L12C:T125M	Non-D69Y Crystal Epitopes	no
G5-009	XX21RECQL5A-x0839	Frozen	22	20	CI080726	HIN3-170302-01	F	9	a	0.2M ammonium sulfate -- 25% PEG3350 -- 0.1M tris pH 8.5	4	Tetramer I 2 2 2/ C 1 2 1	215.76 89.37 79.19 (90.0 108.19 90.0)	C 1 2 1	3.17	5	Xia2-DIALS	17/12/2020 05:51	-	PEG	Q14K	S7N:L12C:T125M	Non-D69Y Crystal Epitopes	yes
G5-009	XX21RECQL5A-x0834	Frozen	22	20	CI080726	HIN3-170302-01	F	9	a	0.2M ammonium sulfate -- 25% PEG3350 -- 0.1M tris pH 8.5	4	Tetramer I 2 2 2/ C 1 2 1	218.6 90.62 80.21 (90.0 108.16 90.0)	C 1 2 1	3.25	1	Xia2-DIALS	17/12/2020 05:37	-	PEG	Q14K	S7N:L12C:T125M	Non-D69Y Crystal Epitopes	yes
G5-009	XX21RECQL5A-x0809	Frozen	22	20	CI080726	HIN3-170302-01	A	5	c	2M ammonium sulfate -- 0.1M HEPES pH 7.5	3	Tetramer P 2 2 21 Compact	88.73 266.17 78.95 (90.0 90.0 90.0)	P 21 21 2	3.48	4	Xia2-DIALS	17/12/2020 04:29	-	High Salt	Q14K	S7N:L12C:T125M	Non-D69Y Crystal Epitopes	yes
G5-009	XX21RECQL5A-x0838	Frozen	22	20	CI080726	HIN3-170302-01	F	9	a	0.2M ammonium sulfate -- 25% PEG3350 -- 0.1M tris pH 8.5	4	Tetramer I 2 2 2/ C 1 2 1	218.84 89.65 79.57 (90.0 107.99 90.0)	C 1 2 1	3.74	4	Xia2-DIALS	17/12/2020 05:48	-	PEG	Q14K	S7N:L12C:T125M	Non-D69Y Crystal Epitopes	yes
G5-009	XX21RECQL5A-x0818	Frozen	22	20	CI080726	HIN3-170302-01	B	7	c	0.056M sodium phosphate monobasic -- 1.344M potassium phosphate dibasic	2	Tetramer P 2 2 21 Porous	89.47 148.88 271.07 (90.0 90.0 90.0)	P 2 2 2	3.92	3	Xia2-DIALS	17/12/2020 04:53	-	High Salt	Q14K	S7N:L12C:T125M	Non-D69Y Crystal Epitopes	yes
G5-009	XX21RECQL5A-x11124	Fresh	22.6	20	CI080525	HIN3-170302-01	B	7	c	0.056M sodium phosphate monobasic -- 1.344M potassium phosphate dibasic	2	Tetramer P 2 2 21 Porous	89.41 151.66 268.97(90.0 90.0 90.0)	P 2 21 21	4.04	2	Xia2-DIALS	02/12/2020 02:24	-	High Salt	Q14K	S7N:L12C:T125M	Non-D69Y Crystal Epitopes	no

Table S4 Diffraction data

Nanobody grouped ID	Mounted Crystal ID	Xtal Plate Protein Frozen/Fresh?	Xtal Plate Protein Concentration (mg/ml)	Xtal Plate Temperature	Xtal Plate Barcode	Screen Batch Name	XTBM Row	XTBM Column	XTBM Subwell	XTBM Condition	Crystal Form Group	Cystal Form Annotation	Unit Cell Dimensions	Space group	Resolution	Number of Successful Autoprocessing Pipelines	Autoprocessing Pipeline Used	Experiment Time	Soaking condition	Xtal Group	Surface Mutations Around Nbinter-1	Key Mutations	Crystal Epitope Mutations	Condition Repitition?
G5-009	XX21RECQ L5A-x0815	Frozen	22	20	CI080726	HIN3-170302-01	A	5	c	2M ammonium sulfate -- 0.1M HEPES pH 7.5	3	Tetramer P 2 2 21 Compact	79.01 89.2 267.2 (90.0 90.0 90.0)	P 2 2 21	4.12	3	Autoproc	17/12/2020 04:46	-	High Salt	Q14K	S7N:L12 C:T125 M	Non-D69Y Crystal Epitopes	yes
G5-009	XX21RECQ L5A-x0805	Frozen	22	20	CI080726	HIN3-170302-01	A	5	c	2M ammonium sulfate -- 0.1M HEPES pH 7.5	-1	NA	88.69 107.86 135.18 (70.23 77.49 74.0)	P 1	4.52	2	Xia2-3dii	17/12/2020 04:10	-	High Salt	Q14K	S7N:L12 C:T125 M	Non-D69Y Crystal Epitopes	yes
G5-009	XX21RECQ L5A-x0831	Frozen	22	20	CI080726	HIN3-170302-01	F	9	a	0.2M ammonium sulfate -- 25% PEG3350 -- 0.1M tris pH 8.5	4	Tetramer I 2 2 2/ C 1 2 1	223.79 90.08 80.17 (90.0 107.91 90.0)	C 1 2 1	4.54	4	Xia2-DIALS	17/12/2020 05:28	-	PEG	Q14K	S7N:L12 C:T125 M	Non-D69Y Crystal Epitopes	yes
G5-009	XX21RECQ L5A-x0798	Frozen	22	20	CI080726	HIN3-170302-01	A	4	a	2M ammonium sulfate -- 0.1M bis-tris pH 6.5	2	Tetramer P 2 2 21 Porous	143.38 274.78 88.88 (90.0 90.0 90.0)	P 21 21 2	4.69	2	Xia2-3dii	17/12/2020 03:48	-	High Salt	Q14K	S7N:L12 C:T125 M	Non-D69Y Crystal Epitopes	yes
G5-009	XX21RECQ L5A-x11122	Fresh	22.6	20	CI080525	HIN3-170302-01	A	5	c	2M ammonium sulfate -- 0.1M HEPES pH 7.5	2	Tetramer P 2 2 21 Porous	88.77 150.17 274.24 (90.0 90.0 90.0)	P 2 21 21	4.92	2	Xia2-DIALS	02/12/2020 12:17	-	High Salt	Q14K	S7N:L12 C:T125 M	Non-D69Y Crystal Epitopes	no
G5-009	XX21RECQ L5A-x0792	Frozen	22	20	CI080726	HIN3-170302-01	A	4	a	2M ammonium sulfate -- 0.1M bis-tris pH 6.5	2	Tetramer P 2 2 21 Porous	89.3 143.26 273.65 (90.0 90.0 90.0)	P 2 21 21	5.03	2	Xia2-DIALS	17/12/2020 03:32	-	High Salt	Q14K	S7N:L12 C:T125 M	Non-D69Y Crystal Epitopes	yes
G5-009	XX21RECQ L5A-x0806	Frozen	22	20	CI080726	HIN3-170302-01	A	5	c	2M ammonium sulfate -- 0.1M HEPES pH 7.5	2	Tetramer P 2 2 21 Porous	88.90 143.21 277.0 (89.99 89.92 89.96)	P 21 21 2	5.36	3	Xia2-DIALS	17/12/2020 04:14	-	High Salt	Q14K	S7N:L12 C:T125 M	Non-D69Y Crystal Epitopes	yes
G5-009	XX21RECQ L5A-x0820	Frozen	22	20	CI080726	HIN3-170302-01	B	7	c	0.056M sodium phosphate monobasic -- 1.344M potassium phosphate dibasic	2	Tetramer P 2 2 21 Porous	89.39 149.94 269.71 (90.0 90.0 90.0)	P 2 2 2	5.37	3	Autoproc	17/12/2020 04:59	-	High Salt	Q14K	S7N:L12 C:T125 M	Non-D69Y Crystal Epitopes	yes
G5-009	XX21RECQ L5A-x0804	Frozen	22	20	CI080726	HIN3-170302-01	A	5	c	2M ammonium sulfate -- 0.1M HEPES pH 7.5	2	Tetramer P 2 2 21 Porous	149.15 276.28 89.13 (90.0 90.0 90.0)	P 21 21 2	5.62	2	Xia2-3dii	17/12/2020 04:07	-	High Salt	Q14K	S7N:L12 C:T125 M	Non-D69Y Crystal Epitopes	yes
G5-009	XX21RECQ L5A-x0810	Frozen	22	20	CI080726	HIN3-170302-01	A	5	c	2M ammonium sulfate -- 0.1M HEPES pH 7.5	2	Tetramer P 2 2 21 Porous	88.99 146.13 275.93 (90.0 90.0 90.0)	P 2 2 2	5.98	3	Autoproc	17/12/2020 04:32	-	High Salt	Q14K	S7N:L12 C:T125 M	Non-D69Y Crystal Epitopes	yes
G5-009	XX21RECQ L5A-x0807	Frozen	22	20	CI080726	HIN3-170302-01	A	5	c	2M ammonium sulfate -- 0.1M HEPES pH 7.5	3	Tetramer P 2 2 21 Compact	78.92 89.06 267.05 (90.0 90.0 90.0)	P 2 2 21	6.12	2	Autoproc	17/12/2020 04:19	-	High Salt	Q14K	S7N:L12 C:T125 M	Non-D69Y Crystal Epitopes	yes
G5-009	XX21RECQ L5A-x0811	Frozen	22	20	CI080726	HIN3-170302-01	A	5	c	2M ammonium sulfate -- 0.1M HEPES pH 7.5	-1	NA	291.07 539.89 175.49 (90.0 90.0 90.0)	P 2 2 21	7.6	1	Xia2-3dii	17/12/2020 04:35	-	High Salt	Q14K	S7N:L12 C:T125 M	Non-D69Y Crystal Epitopes	yes
G5-009	XX21RECQ L5A-x11132	Fresh	22.6	20	CI080525	HIN3-170302-01	F	9	d	0.2M ammonium sulfate -- 25% PEG3350 -- 0.1M tris pH 8.5	NA	NA	NA	NA	NA	NA	NA	02/12/2020 03:05	-	PEG	Q14K	S7N:L12 C:T125 M	Non-D69Y Crystal Epitopes	no
G5-009	XX21RECQ L5A-x11131	Fresh	22.6	20	CI080525	HIN3-170302-01	F	9	c	0.2M ammonium sulfate -- 25% PEG3350 -- 0.1M tris pH 8.5	NA	NA	NA	NA	NA	NA	NA	02/12/2020 02:59	-	PEG	Q14K	S7N:L12 C:T125 M	Non-D69Y Crystal Epitopes	no
G5-009	XX21RECQ L5A-x11130	Fresh	22.6	20	CI080525	HIN3-170302-01	F	9	a	0.2M ammonium sulfate -- 25% PEG3350 -- 0.1M tris pH 8.5	NA	NA	NA	NA	NA	NA	NA	02/12/2020 02:55	-	PEG	Q14K	S7N:L12 C:T125 M	Non-D69Y Crystal Epitopes	no

Table S4 Diffraction data

Nanobody grouped ID	Mounted Crystal ID	Xtal Plate Protein Frozen/Fresh?	Xtal Plate Protein Concentration (mg/ml)	Xtal Plate Temperature	Xtal Plate Barcode	Screen Batch Name	XTBM Row	XTBM Column	XTBM Subwell	XTBM Condition	Crystal Form Group	Cystal Form Annotation	Unit Cell Dimensions	Space group	Resolution	Number of Successful Autoprocessing Pipelines	Autoprocessing Pipeline Used	Experiment Time	Soaking condition	Xtal Group	Surface Mutations Around Nbinter-1	Key Mutations	Crystal Epitope Mutations	Condition Repitition?
G5-009	XX21RECQ L5A-x11129	Fresh	22.6	20	CI080525	HIN3-170302-01	F	8	d	0.2M ammonium sulfate -- 25% PEG3350 -- 0.1M HEPES pH 7.5	NA	NA	NA	NA	NA	NA	NA	02/12/2020 02:49	-	PEG	Q14K	S7N:L12 C:T125 M	Non-D69Y Crystal Epitopes	no
G5-009	XX21RECQ L5A-x11126	Fresh	22.6	20	CI080525	HIN3-170302-01	F	7	c	0.2M ammonium sulfate -- 25% PEG3350 -- 0.1M bis-tris pH 6.5	NA	NA	NA	NA	NA	NA	NA	02/12/2020 02:34	-	PEG	Q14K	S7N:L12 C:T125 M	Non-D69Y Crystal Epitopes	no
G5-009	XX21RECQ L5A-x11125	Fresh	22.6	20	CI080525	HIN3-170302-01	C	1	c	3.5M sodium formate	NA	NA	NA	NA	NA	NA	NA	02/12/2020 02:29	-	High Salt	Q14K	S7N:L12 C:T125 M	Non-D69Y Crystal Epitopes	no
G5-009	XX21RECQ L5A-x11123	Fresh	22.6	20	CI080525	HIN3-170302-01	A	12	a	3M sodium chloride -- 0.1M tris pH 8.5	NA	NA	NA	NA	NA	NA	NA	02/12/2020 02:19	-	High Salt	Q14K	S7N:L12 C:T125 M	Non-D69Y Crystal Epitopes	no
G5-009	XX21RECQ L5A-x11122	Fresh	22.6	20	CI080525	HIN3-170302-01	A	5	c	2M ammonium sulfate -- 0.1M HEPES pH 7.5	NA	NA	NA	NA	NA	NA	NA	02/12/2020 02:14	-	High Salt	Q14K	S7N:L12 C:T125 M	Non-D69Y Crystal Epitopes	no
G5-009	XX21RECQ L5A-x11121	Fresh	22.6	20	CI080525	HIN3-170302-01	A	4	c	2M ammonium sulfate -- 0.1M bis-tris pH 6.5	NA	NA	NA	NA	NA	NA	NA	02/12/2020 02:09	-	High Salt	Q14K	S7N:L12 C:T125 M	Non-D69Y Crystal Epitopes	no
G5-009	XX21RECQ L5A-x0837	Frozen	22	20	CI080726	HIN3-170302-01	F	9	a	0.2M ammonium sulfate -- 25% PEG3350 -- 0.1M tris pH 8.5	NA		NA	NA	NA	0	NA	17/12/2020 05:45	-	PEG	Q14K	S7N:L12 C:T125 M	Non-D69Y Crystal Epitopes	yes
G5-009	XX21RECQ L5A-x0836	Frozen	22	20	CI080726	HIN3-170302-01	F	9	a	0.2M ammonium sulfate -- 25% PEG3350 -- 0.1M tris pH 8.5	NA		NA	NA	NA	0	NA	17/12/2020 05:43	-	PEG	Q14K	S7N:L12 C:T125 M	Non-D69Y Crystal Epitopes	yes
G5-009	XX21RECQ L5A-x0835	Frozen	22	20	CI080726	HIN3-170302-01	F	9	a	0.2M ammonium sulfate -- 25% PEG3350 -- 0.1M tris pH 8.5	NA		NA	NA	NA	0	NA	17/12/2020 05:40	-	PEG	Q14K	S7N:L12 C:T125 M	Non-D69Y Crystal Epitopes	yes
G5-009	XX21RECQ L5A-x0833	Frozen	22	20	CI080726	HIN3-170302-01	F	9	a	0.2M ammonium sulfate -- 25% PEG3350 -- 0.1M tris pH 8.5	NA		NA	NA	NA	0	NA	17/12/2020 05:34	-	PEG	Q14K	S7N:L12 C:T125 M	Non-D69Y Crystal Epitopes	yes
G5-009	XX21RECQ L5A-x0832	Frozen	22	20	CI080726	HIN3-170302-01	F	9	a	0.2M ammonium sulfate -- 25% PEG3350 -- 0.1M tris pH 8.5	NA		NA	NA	NA	0	NA	17/12/2020 05:31	-	PEG	Q14K	S7N:L12 C:T125 M	Non-D69Y Crystal Epitopes	yes
G5-009	XX21RECQ L5A-x0830	Frozen	22	20	CI080726	HIN3-170302-01	F	9	a	0.2M ammonium sulfate -- 25% PEG3350 -- 0.1M tris pH 8.5	NA		NA	NA	NA	0	NA	17/12/2020 05:26	-	PEG	Q14K	S7N:L12 C:T125 M	Non-D69Y Crystal Epitopes	yes
G5-009	XX21RECQ L5A-x0829	Frozen	22	20	CI080726	HIN3-170302-01	F	9	a	0.2M ammonium sulfate -- 25% PEG3350 -- 0.1M tris pH 8.5	NA		NA	NA	NA	0	NA	17/12/2020 05:23	-	PEG	Q14K	S7N:L12 C:T125 M	Non-D69Y Crystal Epitopes	yes
G5-009	XX21RECQ L5A-x0828	Frozen	22	20	CI080726	HIN3-170302-01	F	9	a	0.2M ammonium sulfate -- 25% PEG3350 -- 0.1M tris pH 8.5	NA		NA	NA	NA	0	NA	17/12/2020 05:21	-	PEG	Q14K	S7N:L12 C:T125 M	Non-D69Y Crystal Epitopes	yes
G5-009	XX21RECQ L5A-x0827	Frozen	22	20	CI080726	HIN3-170302-01	B	7	c	0.056M sodium phosphate monobasic -- 1.344M potassium phosphate dibasic	NA		NA	NA	NA	0	NA	17/12/2020 05:18	-	High Salt	Q14K	S7N:L12 C:T125 M	Non-D69Y Crystal Epitopes	yes

Table S4 Diffraction data

Nanobody grouped ID	Mounted Crystal ID	Xtal Plate Protein Frozen/Fresh?	Xtal Plate Protein Concentration (mg/ml)	Xtal Plate Temperature	Xtal Plate Barcode	Screen Batch Name	XTBM Row	XTBM Column	XTBM Subwell	XTBM Condition	Crystal Form Group	Cystal Form Annotation	Unit Cell Dimensions	Space group	Resolution	Number of Successful Autoprocessing Pipelines	Autoprocessing Pipeline Used	Experiment Time	Soaking condition	Xtal Group	Surface Mutations Around Nbinter-1	Key Mutations	Crystal Epitope Mutations	Condition Repitition?
G5-009	XX21RECQL5A-x0826	Frozen	22	20	CI080726	HIN3-170302-01	B	7	c	0.056M sodium phosphate monobasic -- 1.344M potassium phosphate dibasic	NA		NA	NA	NA	0	NA	17/12/2020 05:15	-	High Salt	Q14K	S7N:L12C:T125M	Non-D69Y Crystal Epitopes	yes
G5-009	XX21RECQL5A-x0825	Frozen	22	20	CI080726	HIN3-170302-01	B	7	c	0.056M sodium phosphate monobasic -- 1.344M potassium phosphate dibasic	NA		NA	NA	NA	0	NA	17/12/2020 05:13	-	High Salt	Q14K	S7N:L12C:T125M	Non-D69Y Crystal Epitopes	yes
G5-009	XX21RECQL5A-x0824	Frozen	22	20	CI080726	HIN3-170302-01	B	7	c	0.056M sodium phosphate monobasic -- 1.344M potassium phosphate dibasic	NA		NA	NA	NA	0	NA	17/12/2020 05:11	-	High Salt	Q14K	S7N:L12C:T125M	Non-D69Y Crystal Epitopes	yes
G5-009	XX21RECQL5A-x0823	Frozen	22	20	CI080726	HIN3-170302-01	B	7	c	0.056M sodium phosphate monobasic -- 1.344M potassium phosphate dibasic	NA		NA	NA	NA	0	NA	17/12/2020 05:08	-	High Salt	Q14K	S7N:L12C:T125M	Non-D69Y Crystal Epitopes	yes
G5-009	XX21RECQL5A-x0822	Frozen	22	20	CI080726	HIN3-170302-01	B	7	c	0.056M sodium phosphate monobasic -- 1.344M potassium phosphate dibasic	NA		NA	NA	NA	0	NA	17/12/2020 05:05	-	High Salt	Q14K	S7N:L12C:T125M	Non-D69Y Crystal Epitopes	yes
G5-009	XX21RECQL5A-x0821	Frozen	22	20	CI080726	HIN3-170302-01	B	7	c	0.056M sodium phosphate monobasic -- 1.344M potassium phosphate dibasic	NA		NA	NA	NA	0	NA	17/12/2020 05:02	-	High Salt	Q14K	S7N:L12C:T125M	Non-D69Y Crystal Epitopes	yes
G5-009	XX21RECQL5A-x0819	Frozen	22	20	CI080726	HIN3-170302-01	B	7	c	0.056M sodium phosphate monobasic -- 1.344M potassium phosphate dibasic	NA		NA	NA	NA	0	NA	17/12/2020 04:56	-	High Salt	Q14K	S7N:L12C:T125M	Non-D69Y Crystal Epitopes	yes
G5-009	XX21RECQL5A-x0817	Frozen	22	20	CI080726	HIN3-170302-01	B	7	c	0.056M sodium phosphate monobasic -- 1.344M potassium phosphate dibasic	NA		NA	NA	NA	0	NA	17/12/2020 04:51	-	High Salt	Q14K	S7N:L12C:T125M	Non-D69Y Crystal Epitopes	yes
G5-009	XX21RECQL5A-x0816	Frozen	22	20	CI080726	HIN3-170302-01	B	7	c	0.056M sodium phosphate monobasic -- 1.344M potassium phosphate dibasic	NA		NA	NA	NA	0	NA	17/12/2020 04:48	-	High Salt	Q14K	S7N:L12C:T125M	Non-D69Y Crystal Epitopes	yes
G5-009	XX21RECQL5A-x0814	Frozen	22	20	CI080726	HIN3-170302-01	A	5	c	2M ammonium sulfate -- 0.1M HEPES pH 7.5	NA		NA	NA	NA	0	NA	17/12/2020 04:43	-	High Salt	Q14K	S7N:L12C:T125M	Non-D69Y Crystal Epitopes	yes
G5-009	XX21RECQL5A-x0813	Frozen	22	20	CI080726	HIN3-170302-01	A	5	c	2M ammonium sulfate -- 0.1M HEPES pH 7.5	NA		NA	NA	NA	0	NA	17/12/2020 04:40	-	High Salt	Q14K	S7N:L12C:T125M	Non-D69Y Crystal Epitopes	yes
G5-009	XX21RECQL5A-x0812	Frozen	22	20	CI080726	HIN3-170302-01	A	5	c	2M ammonium sulfate -- 0.1M HEPES pH 7.5	NA		NA	NA	NA	0	NA	17/12/2020 04:38	-	High Salt	Q14K	S7N:L12C:T125M	Non-D69Y Crystal Epitopes	yes
G5-009	XX21RECQL5A-x0808	Frozen	22	20	CI080726	HIN3-170302-01	A	5	c	2M ammonium sulfate -- 0.1M HEPES pH 7.5	NA		NA	NA	NA	0	NA	17/12/2020 04:25	-	High Salt	Q14K	S7N:L12C:T125M	Non-D69Y Crystal Epitopes	yes
G5-009	XX21RECQL5A-x0803	Frozen	22	20	CI080726	HIN3-170302-01	A	4	a	2M ammonium sulfate -- 0.1M bis-tris pH 6.5	NA		NA	NA	NA	0	NA	17/12/2020 04:03	-	High Salt	Q14K	S7N:L12C:T125M	Non-D69Y Crystal Epitopes	yes
G5-009	XX21RECQL5A-x0802	Frozen	22	20	CI080726	HIN3-170302-01	A	4	a	2M ammonium sulfate -- 0.1M bis-tris pH 6.5	NA		NA	NA	NA	0	NA	17/12/2020 04:00	-	High Salt	Q14K	S7N:L12C:T125M	Non-D69Y Crystal Epitopes	yes

Table S4 Diffraction data

Nanobody grouped ID	Mounted Crystal ID	Xtal Plate Protein Frozen/Fresh?	Xtal Plate Protein Concentration (mg/ml)	Xtal Plate Temperature	Xtal Plate Barcode	Screen Batch Name	XTBM Row	XTBM Column	XTBM Subwell	XTBM Condition	Crystal Form Group	Cystal Form Annotation	Unit Cell Dimensions	Space group	Resolution	Number of Successful Autoprocessing Pipelines	Autoprocessing Pipeline Used	Experiment Time	Soaking condition	Xtal Group	Surface Mutations Around Nbinter-1	Key Mutations	Crystal Epitope Mutations	Condition Repitition?
G5-009	XX21RECQ L5A-x0801	Frozen	22	20	CI080726	HIN3-170302-01	A	4	a	2M ammonium sulfate -- 0.1M bis-tris pH 6.5	NA		NA	NA	NA	0	NA	17/12/2020 03:57	-	High Salt	Q14K	S7N:L12 C:T125 M	Non-D69Y Crystal Epitopes	yes
G5-009	XX21RECQ L5A-x0800	Frozen	22	20	CI080726	HIN3-170302-01	A	4	a	2M ammonium sulfate -- 0.1M bis-tris pH 6.5	NA		NA	NA	NA	0	NA	17/12/2020 03:54	-	High Salt	Q14K	S7N:L12 C:T125 M	Non-D69Y Crystal Epitopes	yes
G5-009	XX21RECQ L5A-x0799	Frozen	22	20	CI080726	HIN3-170302-01	A	4	a	2M ammonium sulfate -- 0.1M bis-tris pH 6.5	NA		NA	NA	NA	0	NA	17/12/2020 03:51	-	High Salt	Q14K	S7N:L12 C:T125 M	Non-D69Y Crystal Epitopes	yes
G5-009	XX21RECQ L5A-x0797	Frozen	22	20	CI080726	HIN3-170302-01	A	4	a	2M ammonium sulfate -- 0.1M bis-tris pH 6.5	NA		NA	NA	NA	0	NA	17/12/2020 03:45	-	High Salt	Q14K	S7N:L12 C:T125 M	Non-D69Y Crystal Epitopes	yes
G5-009	XX21RECQ L5A-x0796	Frozen	22	20	CI080726	HIN3-170302-01	A	4	a	2M ammonium sulfate -- 0.1M bis-tris pH 6.5	NA		NA	NA	NA	0	NA	17/12/2020 03:43	-	High Salt	Q14K	S7N:L12 C:T125 M	Non-D69Y Crystal Epitopes	yes
G5-009	XX21RECQ L5A-x0795	Frozen	22	20	CI080726	HIN3-170302-01	A	4	a	2M ammonium sulfate -- 0.1M bis-tris pH 6.5	NA		NA	NA	NA	0	NA	17/12/2020 03:40	-	High Salt	Q14K	S7N:L12 C:T125 M	Non-D69Y Crystal Epitopes	yes
G5-009	XX21RECQ L5A-x0794	Frozen	22	20	CI080726	HIN3-170302-01	A	4	a	2M ammonium sulfate -- 0.1M bis-tris pH 6.5	NA		NA	NA	NA	0	NA	17/12/2020 03:37	-	High Salt	Q14K	S7N:L12 C:T125 M	Non-D69Y Crystal Epitopes	yes
G5-009	XX21RECQ L5A-x0793	Frozen	22	20	CI080726	HIN3-170302-01	A	4	a	2M ammonium sulfate -- 0.1M bis-tris pH 6.5	NA		NA	NA	NA	0	NA	17/12/2020 03:35	-	High Salt	Q14K	S7N:L12 C:T125 M	Non-D69Y Crystal Epitopes	yes

Table S5 Detailed information with numeric values of each cell in Figure 5A

Table S5 Crystal hit conditions

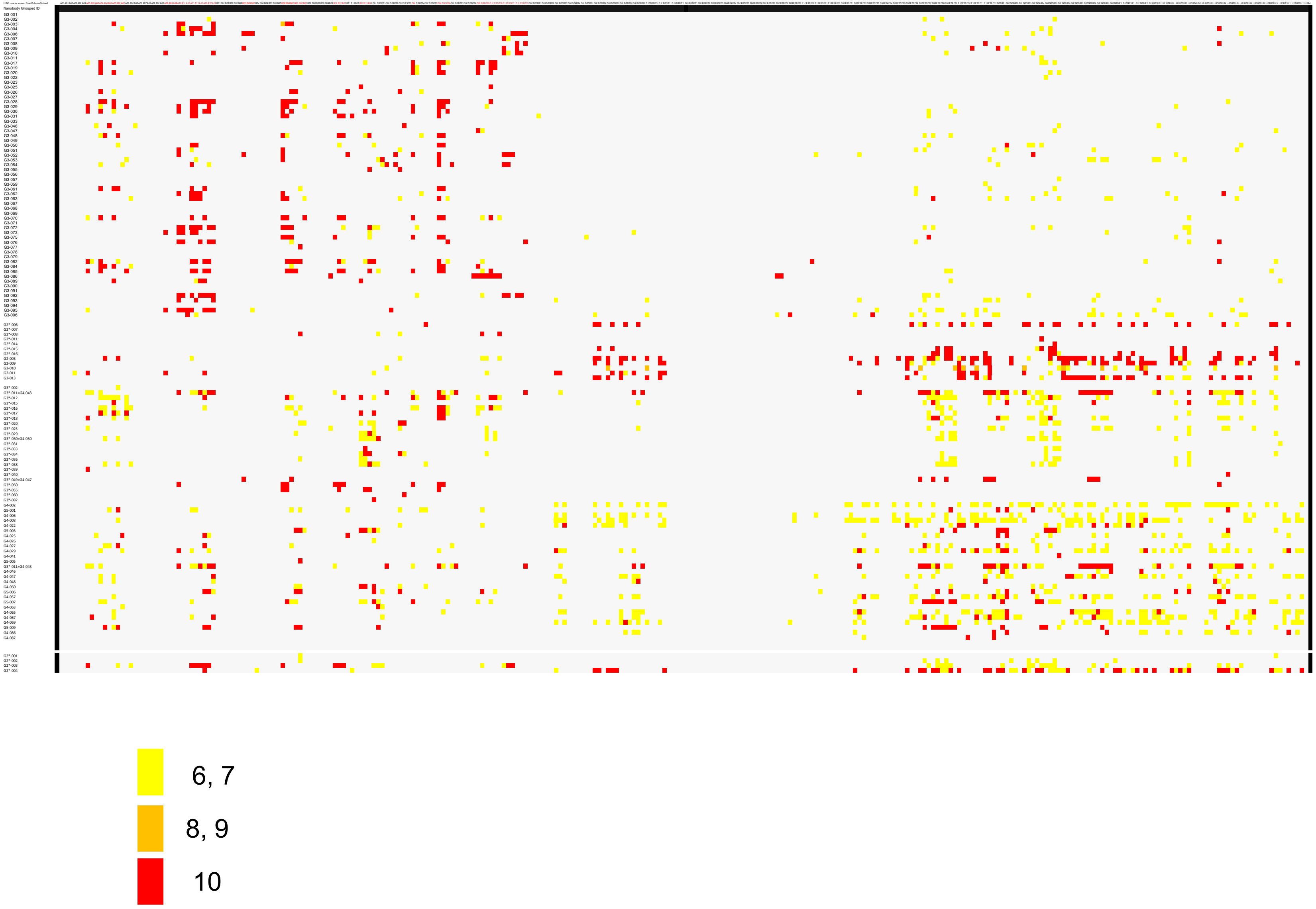

Table S6 Data collection and refinement statistics of MPP8 in complex with the Gluebody

	**mpp8 chromodomain in complex with 3A02 Gluebody**
**Wavelength**	0.99986
**Resolution range**	59.1 - 2.01 (2.04 - 2.01)
**Space group**	P 21 21 21
**Unit cell**	52.933 73.454 99.527 90 90 90
**Total reflections**	565911
**Unique reflections**	25877 (1310)
**Multiplicity**	21.3 (13.39)
**Completeness (%)**	100 (90.12)
**Mean I/sigma(I)**	3.6 (0.5)
**Wilson B-factor**	28.39
**R-merge**	0.545 (3.192)
**R-meas**	0.559 (3.322)
**R-pim**	0.121
**CC1/2**	0.993 (0.220)
**Reflections used in refinement**	25877
**Reflections used for R-free**	1904
**R-work**	0.2523
**R-free**	0.2802
**Number of non-hydrogen atoms**	2831
**macromolecules**	2696
**ligands**	0
**solvent**	135
**Protein residues**	354

**RMS(bonds)**	0.002
**RMS(angles)**	0.43
**Ramachandran favored (%)**	99.71
**Ramachandran allowed (%)**	0.29
**Ramachandran outliers (%)**	0.00
**Rotamer outliers (%)**	0.00
**Clashscore**	2.48
**Average B-factor**	32.97
**macromolecules**	32.79
**solvent**	36.55

Statistics for the highest-resolution shell are shown in parentheses.
