# Supplementary material for: Gluebodies Offer a Route To Improve Crystal Reliability and Diversity through Transferable Nanobody Mutations That Introduce Constitutive Close Contacts
Source: ACS Cent Sci. 2025 Oct 27;11(12):2385–99. doi: 10.1021/acscentsci.5c00937 (PMC12746149; doi:10.1021/acscentsci.5c00937)
Supplement: Supplementary file 2 [file oc5c00937_si_002.pdf]

oc-2025-00937g.R1

Name: Peer Review Information for "Gluebodies offer a route to improve crystal reliability and diversity through transferable nanobody mutations that introduce constitutive close contacts"

First Round of Reviewer Comments

Reviewer: 1

Comments to the Author

In the manuscript “Gluebodies improve crystal reliability and diversity through transferable nanobody mutations that introduce constitutive close contacts”, Ye et al. address a significant bottleneck in high-resolution protein structure determination. Many proteins are difficult or impossible to crystallize for X-ray diffraction studies. However, the ongoing need to visualize protein-ligand complexes, especially for therapeutic development, makes this work broadly significant. Through a large structure-mining approach, the authors identify crucial interactions that enhance nanobody-nanobody interfaces, facilitating protein crystallization. Multiple generations of mutational studies and extensive crystal screening then result in a construct, dubbed a Gluebody, which enables high-resolution structure determination from various crystal forms obtained under numerous crystallization conditions.

The manuscript is well-written and follows a logical progression. The experimental approach is both innovative and sound. However, a few concerns arise regarding the presentation of the data and the interpretation of the results.

1. The nomenclature of nanobody constructs and their evolution should be more clearly explained to facilitate better understanding for the reader.

2. One significant open question is the general applicability of the developed Gluebody. Table 1 indicates that for proteins that are difficult to crystallize, even the new approach does not result in diffracting crystals or only yields low-resolution structures. The authors should discuss the limitations in greater detail. Furthermore, they might consider adjusting

the title of the manuscript to avoid misleading the reader. As it stands, the general applicability appears to be lacking.

3. The entire manuscript focuses on X-ray crystallography. Therefore, the application of the Gluebody approach to cryo-EM seems out of place in the discussion and distracting. If this is meant as an additional application of Gluebodies, then a more detailed discussion is necessary.

Minor points:

P9, line 53: typo ‘futher’

P12, line 52: typo ‘from’

P14, line 57: typo ‘submit’

Reviewer: 2

Comments to the Author

This manuscript introduces the concept of “Gluebodies” — nanobody variants engineered to enhance protein crystallization by introducing transferable crystal contact interfaces. The central idea is creative and has clear potential, particularly in structural biology, where obtaining high-quality crystals remains a major hurdle. The authors present a well-executed engineering effort using the RECQL5 system and offer preliminary insights into the method’s applicability to other targets.   However, despite the clear effort and ambition, the current version does not yet meet the standards of generality, reproducibility, and mechanistic rigor expected for publication in ACS Central Science. The conclusions are interesting, but are primarily drawn from a single protein system, with limited validation across other targets. Substantial revision is required to demonstrate the broad claims and practicality of the Gluebody platform. If this is not possible, the reviewer encourages submission to Crystal Growth & Design.

Major Points

1. Although this paper suggests that Gluebodies may be widely adopted, the selection of Gluebodies for target proteins and high-throughput crystallization involving the purification of a large number of Gluebody mutants have not been demonstrated. Therefore, the current workflow, which involves extensive structural screening, may be beyond the reach of many laboratories. How can we address this lack of experimental versatility?

2. The Gluebody approach is rigorously developed and validated for RECQL5, but among six additional targets, only one (MPP8) yielded a structure with improved diffraction. The remaining cases resulted in low-resolution, unsolvable, or absent crystals. The authors should clearly define the scope of applicability. Which target properties (e.g., molecular weight, surface charge, conformational flexibility) are most likely to influence Gluebody performance? Providing a set of guidelines or a predictive framework would greatly enhance the practical value of the work.

Author's Response to Peer Review Comments:

Dear Editor,

Thank you very much for your patience, and we have now uploaded the point-by-point response to reviewers and the revised manuscript on the portal. We thank the reviewers for the valuable advice and attention on our work. In summary, we have the following changes to the manuscript:

- We have addressed the general applicability of the Gluebody approach by reviewing our tone throughout.
- We have introduced a simplified Gluebody testing strategy, which could be implemented in standard laboratory settings.
- We have introduced more descriptive paragraphs and references to clarify our nomenclature.
- We have corrected the manuscript throughout according to the formatting needs.

We thank the reviewers again for their instructive input and we look forward to further communication.

Kindest regards,

Mingda

We again thank them for their constructive input. We have adjusted the manuscript as follows; changes that address reviewers' comments are highlighted in the text.

Formatting Needs:

Abstract: Please make sure the word count of your Abstract does not exceed 200 words.

Now 190 words

Author Affiliations: Please include postal codes/country in the author affiliations in the publication file(s).

Done

Graphics: If a figure has parts labeled (i.e. a, b, etc.), each part must be mentioned in the figure caption.

Done

Supporting Information: If the manuscript is accompanied by any Supporting Information for Publication, a brief description of the supplementary material is required in the manuscript, before the reference list. The appropriate format is: Supporting Information. Brief statement in non-sentence format listing the contents of the material supplied as Supporting Information. Please list each supporting item individually.

**Examples of sufficient descriptions: "Supporting Information: 1H NMR spectra for all compounds" or "Additional experimental details, materials, and methods, including photographs of experimental setup."*

**Examples of insufficient descriptions: "Supporting Information: Figures S1-S3" or "Additional figures as mentioned in the text."*

Acknowledged.

Supporting Information: Please remove the supplementary figures from the main manuscript and upload in a separate file. Please add a full header to the top of the file designated "Supporting Information for Publication." Provide the title (in title case), authors' names, and affiliations on the top of the first page, matching those of the manuscript file exactly.

Acknowledged.

Supporting Information: Please label all graphics/tables in the following format: "Figure S1, S2…", "Scheme S1, S2…." or "Table S1, S2…", etc.

Acknowledged.

Supporting Information: Please number all pages in the following format: S1, S2, S3, etc.

Acknowledged.

Synopsis: ACS Central Science requires a brief synopsis. The synopsis should be no more than 200 characters (including spaces) and should reasonably correlate with the Table of Contents (TOC) graphic. The synopsis is intended to explain the importance of the article to a broader readership across the sciences. Please place your synopsis in the manuscript file after the TOC graphic and label as "Synopsis."

Now supplied, on page 2 of the manuscript.

TOC Graphic: Include a TOC graphic illustrating the significance of the paper. The TOC graphic should be something that is representative of your entire work. Color schemes or illustrations typically make good choices. The TOC graphic must be original and free from any copyright issues. Confirm that all text is legible. Present the TOC graphic on the last page of the manuscript by itself. Please label the TOC as "TOC Graphic". A caption describing the TOC is not needed. Please see more information/guidelines for TOC Graphics at the following link: http://pubsapp.acs.org/paragonplus/submission/toc_abstract_graphics_guidelines.pdf?

Now supplied, on page 2 of the manuscript.

Reviewer(s)' Comments to Author:
Reviewer: 1

Recommendation: Publish in ACS Central Science after minor revisions noted.
Comments:

In the manuscript "Gluebodies improve crystal reliability and diversity through transferable nanobody mutations that introduce constitutive close contacts", Ye et al. address a significant bottleneck in high-resolution protein structure determination. Many proteins are difficult or impossible to crystallize for X-ray diffraction studies. However, the ongoing need to visualize protein-ligand complexes, especially for therapeutic development, makes this work broadly significant. Through a large structure-mining approach, the authors identify crucial interactions that enhance nanobody-nanobody interfaces, facilitating protein crystallization. Multiple generations of mutational studies and extensive crystal screening then result in a construct, dubbed a Gluebody, which enables high-resolution structure determination from various crystal forms obtained under numerous crystallization conditions.
The manuscript is well-written and follows a logical progression. The experimental approach is both innovative and sound. However, a few concerns arise regarding the presentation of the data and the interpretation of the results.

1. The nomenclature of nanobody constructs and their evolution should be more clearly explained to facilitate better understanding for the reader.

> We have addressed this by: (a) clarifying the nomenclature in a new, descriptive paragraph at the end of the 4th results section (highlighted), which outlines the progression of the Gluebody generations and their associated mutations; and (b) referencing a supplementary figure that summarizes the structural logic behind each generation.

2. One significant open question is the general applicability of the developed Gluebody. Table 1 indicates that for proteins that are difficult to crystallize, even the new approach does not result in diffracting crystals or only yields low-resolution structures. The authors should discuss the limitations in greater detail. Furthermore, they might consider adjusting the title of the manuscript to avoid misleading the reader. As it stands, the general applicability appears to be lacking.

> We have addressed this important point, that the claims must be properly qualified, by reviewing the tone throughout. We have adjusted the following, with changes highlighted in the text:
>
> - Manuscript title and abstract
> - Heading of the final results section

- Expanded the 6th and concluding section of Results, and the 2nd section of the Discussion, to note that the Gluebody are not universally effective, especially for targets intrinsically reluctant to crystallize; and to spell out guidelines for users to assess likely applicability based on protein characteristics.
- Added Tables 1, 2 and 3 to make the subtleties fully transparent.

3. The entire manuscript focuses on X-ray crystallography. Therefore, the application of the Gluebody approach to cryo-EM seems out of place in the discussion and distracting. If this is meant as an additional application of Gluebodies, then a more detailed discussion is necessary.

We have now tidied up this point, which we had indeed managed to garble thoroughly, by properly contextualising it with our crystallographic observations of covalent interactions. We now properly reference our study, now published, that builds on this observation. We followed the reviewer's advice not to distract from the manuscript's crystallography focus, and thus kept this to a short paragraph at the very end.

Minor points:
P9, line 53: typo 'futher'
P12, line 52: typo 'from'
P14, line 57: typo 'submit'

Now corrected.

Additional Questions:
Quality of experimental data, technical rigor: Top 10%

Significance to chemistry researchers in this and related fields: Top 10%

Broad interest to other researchers: Top 1%

Novelty: Top 1%

Is this research study suitable for media coverage or a First Reactions (a News & Views piece in the journal)?: No

Reviewer: 2

Recommendation: Major revisions required.

Comments:
This manuscript introduces the concept of "Gluebodies" — nanobody variants engineered to enhance protein crystallization by introducing transferable crystal contact interfaces. The central idea is creative and has clear potential, particularly in structural biology, where obtaining high-quality crystals remains a major hurdle. The authors present a well-executed engineering effort using the RECQL5 system and offer preliminary insights into the method's applicability to other targets.
However, despite the clear effort and ambition, the current version does not yet meet the standards of generality, reproducibility, and mechanistic rigor expected for publication in ACS Central Science.

The conclusions are interesting, but are primarily drawn from a single protein system, with limited validation across other targets. Substantial revision is required to demonstrate the broad claims and practicality of the Gluebody platform. If this is not possible, the reviewer encourages submission to Crystal Growth & Design.

Major Points
1. Although this paper suggests that Gluebodies may be widely adopted, the selection of Gluebodies for target proteins and high-throughput crystallization involving the purification of a large number of Gluebody mutants have not been demonstrated. Therefore, the current workflow, which involves extensive structural screening, may be beyond the reach of many laboratories. How can we address this lack of experimental versatility?

> A paragraph has been added to the Discussion (paragraph 5), spelling out that a simplified Gluebody testing strategy may be implemented in standard laboratory settings.

2. The Gluebody approach is rigorously developed and validated for RECQL5, but among six additional targets, only one (MPP8) yielded a structure with improved diffraction. The remaining cases resulted in low-resolution, unsolvable, or absent crystals. The authors should clearly define the scope of applicability. Which target properties (e.g., molecular weight, surface charge, conformational flexibility) are most likely to influence Gluebody performance? Providing a set of guidelines or a predictive framework would greatly enhance the practical value of the work.

> This point is the same as point 2 of Reviewer 1, and is thus addressed by the changes described above.

Additional Questions:
Quality of experimental data, technical rigor: Moderate

Significance to chemistry researchers in this and related fields: Moderate

Broad interest to other researchers: Moderate

Novelty: Moderate

Is this research study suitable for media coverage or a First Reactions (a News & Views piece in the journal)?: No

oc-2025-00937g.R2

Name: Peer Review Information for "Gluebodies offer a route to improve crystal reliability and diversity through transferable nanobody mutations that introduce constitutive close contacts"

Second Round of Reviewer Comments

Reviewer: 1

Comments to the Author

All of my original comments have been satisfactorily addressed.

Reviewer: 2

Comments to the Author

The authors have responded sincerely to the reviewer's comments. However, these explanations merely clarify that this Gluebody is not versatile for crystallizing various proteins and does not offer any advantages over conventional methods, such as efficiency, in the crystallization process. Therefore, this manuscript is not worthy of interest to the broad readership of ACS Central Science. The reviewer recommends resubmitting the manuscript to a specialized journal in crystal engineering or protein science.

Reviewer: 3

Comments to the Author

The authors have appropriately addressed prior reviewer comments.

Author's Response to Peer Review Comments:

Dear Editor,

Thanks very much for your decision letter and we are very glad to proceed through with this revision. Therefore, we have revised the manuscript according to the formatting needs set out as follows:

Formatting Needs:

1. Author Affiliations: Please include postal codes in the author affiliations in the publication file(s).

We have included post codes for every affiliation.

2. Ethics Statement: Your experimental section appears to detail the use of human or animal samples/participants. As per ACS's ethical guidelines section B15, "The American Chemical Society Publications rules and ethical guidelines provide mandatory standards of practice in experimental studies performed using biological samples obtained from animals or human subjects. Studies submitted for publication approval must present evidence that the described experimental activities have undergone local institutional review assessing safety and humane usage of study subject animals. In the case of human subjects, authors must also provide a statement that study samples were obtained through the informed consent of the donors, or in lieu of that evidence, by the authority of the institutional board that licensed the use of such material. Authors are requested to declare the identification or case number of institution approval as well as the name of the licensing committee in a statement placed in the section describing the studies' Material and Methods."

Please add a statement to your methods section detailing your ethics approval process for the use of animal/human participants. The statement should provide the identification or case number of institutional approval as well as the name of the licensing committee that

approved the experiments. For research involving human participants/samples, please also include a statement detailing your informed consent protocol.

We have moved the ‘Ethics’ paragraph with the identification of institution approval and licensing committee to the section describing the studies ‘Materials and Methods’.

3. Highlighting: Please submit your publication files without any markups. Any copies that contain highlights, colored text, or tracked changes should be submitted as "Supporting Information for Review Only."

We have removed all the markups throughout the manuscript.

4. Supporting Information: Please remove the Supporting Information material from the manuscript file and upload it as a “Supporting Information for Publication” file. Please ensure this file is formatted as follows:

a.) Add a full header (at top of page 1) to the Supporting Information file, which includes the heading “Supporting Information” at the top followed by the Manuscript Title, Full Author List and Author Affiliations (exactly as they appear in the manuscript).

We have consolidated all the supporting documents into one file with a full header page according to this requirement.

b.) Label all graphics/tables in the Supporting Information file in the following format: “Figure S1, S2...”, “Scheme S1, S2....” or “Table S1, S2...”, etc.

We have changed the titles of each supporting item as per requirements.

c.) Number all pages in the Supporting Information file in the following format: S1, S2, S3, etc.

We have numbered all pages in this supporting information file as S1, S2, S3 etc.

d.) Supporting Information Statement: A brief, non-sentence description of the actual contents of each Supporting Information file is required. Please provide the brief description at the end of the manuscript file before the Acknowledgments and References sections with the heading "Supporting Information". Please avoid long paragraphs or lists of supplemental figure captions. Examples of sufficient and insufficient descriptions are as follows:

We have added a section of supporting information statement in the main manuscript file.

We sincerely appreciate the editing and revision work from you and the reviewers, and thank you again for the valuable advice to improve this manuscript. We look forward to hearing back from you again!

Yours sincerely,

Mingda Ye
